# Genome-wide investigation and expression analysis of AP2-ERF gene family in salt tolerant common bean

**DOI:** 10.17179/excli2015-600

**Published:** 2015-11-27

**Authors:** Musa Kavas, Aslihan Kizildogan, Gökhan Gökdemir, Mehmet Cengiz Baloglu

**Affiliations:** 1 Ondokuz Mayis University, Faculty of Agriculture, Department of Agricultural Biotechnology, Samsun, Turkey; 2 Kastamonu University, Faculty of Engineering and Architecture, Department of Genetics and Bioengineering, Kastamonu, Turkey

**Keywords:** AP2-ERF family, miRNA, interactome, salt resistance, transcriptome analyses

## Abstract

Apetala2-ethylene-responsive element binding factor (AP2-ERF) superfamily with common AP2-DNA binding domain have developmentally and physiologically important roles in plants. Since common bean genome project has been completed recently, it is possible to identify all of the *AP2-ERF* genes in the common bean genome. In this study, a comprehensive genome-wide in silico analysis identified 180 AP2-ERF superfamily genes in common bean (*Phaseolus vulgaris*). Based on the amino acid alignment and phylogenetic analyses, superfamily members were classified into four subfamilies: DREB (54), ERF (95), AP2 (27) and RAV (3), as well as one soloist. The physical and chemical characteristics of amino acids, interaction between AP2-ERF proteins, cis elements of promoter region of *AP2-ERF* genes and phylogenetic trees were predicted and analyzed. Additionally, expression levels of *AP2-ERF* genes were evaluated by in silico and qRT-PCR analyses. In silico micro-RNA target transcript analyses identified nearly all *PvAP2-ERF* genes as targets of by 44 different plant species' miRNAs were identified in this study. The most abundant target genes were *PvAP2/ERF-20-25-62-78-113-173*. miR156, miR172 and miR838 were the most important miRNAs found in targeting and BLAST analyses. Interactome analysis revealed that the transcription factor *PvAP2-ERF78*, an ortholog of Arabidopsis *At2G28550*, was potentially interacted with at least 15 proteins, indicating that it was very important in transcriptional regulation. Here we present the first study to identify and characterize the AP2-ERF transcription factors in common bean using whole-genome analysis, and the findings may serve as a references for future functional research on the transcription factors in common bean.

## Introduction

Pulse crops are very well known species with their nitrogen fixation capacity via nodulation in symbiosis with rhizobia. Common bean, *Phaseolus vulgaris* L., is the most important leguminous crop in human diet as a natural nutritional source with its high protein, complex carbohydrate and micronutrients content. It is marketed in different forms such as canned beans, green beans or dried beans and it is consumed mostly in grain form worldwide (Broughton et al., 2003[6]). As a crucial nutritional source, the global production of common bean is estimated to be 23 million metric tons and India is in first rank with 3.3 million tons annual bean production (FAO, 2014[17]). Due to its economic value especially for developing countries, studies have focused on improvement of agronomic and nutritional traits of this legume. Analysis of gene expression upon exposure to biotic and abiotic stresses (Liu et al., 2012[35]; Hiz et al., 2014[21]) or post-transcriptional regulation of gene expression (Contreras-Cubas et al., 2012[12]) are among the common approaches performed. Meanwhile, the complete genome sequence of common bean has been recently published (Schmutz et al., 2014[51]). Its release further extended genome-wide identification and expression analyses of genes and their regulatory networks to improve knowledge about common bean (Formey et al., 2015[18]). High-salt content in soil is an adverse environmental factor that threatens growth and productivity of plants thereby reducing agricultural sustainability in affected lands (Munns, 2002[43]; Qin et al., 2011[47]). Soil salinity exerts its effect on plants by causing ionic and osmotic stresses, which disturb plant's main metabolic processes such as photosynthesis, lipid metabolism and protein synthesis (Parida and Das, 2005[46]). Being a glycophyte, *P. vulgaris* is negatively influenced even in the presence of 1 dS/m salinity level (Chinnusamy et al., 2005[10]). In order to overcome hazardous effects of abiotic conditions, plants have evolved different response mechanisms at molecular, physiological and biochemical levels (Zhou et al., 2010[68]). The stress response leads to a diverse set of gene expression regulated by specific transcription factors (TFs) (Bouaziz et al., 2015[5]). Analysis of the function of such TFs provides useful insights to understand molecular mechanisms that govern plant abiotic stress tolerance and elucidates development of genetic manipulation strategies to maintain crop productivity (Kalavacharla et al., 2011[27]).

Apetala2/ethylene-responsive element binding factor (AP2/ERF) family is among central plant specific transcription factors that have crucial roles in the regulation of stress-responsive gene expression upon exposure to abiotic stresses such as salinity, drought, temperature fluctuation, disease resistance, and under floral development (Yamaguchi-Shinozaki and Shinozaki, 2006[65]; Mizoi et al., 2012[40]). By the presence of conserved AP2-ERF DNA-binding domain composed of 57-70 amino acid residue, AP2-ERF binds to specific *cis*-acting elements located at promoter regions of related stress-responsive target genes. These *cis*-acting elements are named as GCC box and/or *cis*-acting dehydration responsive element/C-repeat element (DRE/CRT) (Wessler, 2005[61]; Zhou et al., 2010[68]). AP2-ERF family is divided into five subgroups according to the sequence similarities and the number of AP2 domains they possess: 

(i) AP2 (APETALA2), 

(ii) RAV (related to ABI3/VP1), 

(iii) ERF, 

(iv) DREB (dehydration-responsive element-binding protein) and 

(v) other ones (Sakuma et al., 2002[50]). 

AP2 protein contains two tandem AP2 domains (Nakano et al., 2006[44]) while RAV family includes a B3 domain in addition to a single AP2 domain (Hu and Liu, 2011[24]). Since ERF and DREB proteins contain only a single AP2, they are distinguished from each other by the presence of different amino acid residues. The conserved amino acids in positions 14 and 19 are alanine and aspartic acid for ERF, and valine and glutamine for DREB, respectively (Sharma et al., 2010[52]).

Up to now, several AP2/ERF-related proteins have been identified in different plant species; that of 147 in Arabidopsis (Sakuma et al., 2002[50]; Nakano et al., 2006[44]), 200 in poplar (Zhuang et al., 2008[69]), 117 in wheat (Zhuang et al., 2011[70]), 58 in apple (Zhuang et al., 2011[71]), 149 in grape (Licausi et al., 2010[34]), 119 in castor bean (Xu et al., 2013[64]), 210 in potato (Massa et al., 2011[39]) and 164 in rice (Riano-Pachon et al., 2007[49]). In *P. vulgaris*, AP2-ERF transcription factor (AP2-ERF TF) family has not been analyzed at the genome-wide scale. Thus, herein we report for the first time a comprehensive genome-wide analysis of AP2-ERF TFs in common bean under salt stress conditions. In addition, the characterization of genomic structures, chromosomal locations, promoter analyses, interactome analyses, sequence homologies of all common bean *AP2-ERF* TF genes were also undertaken in this study. The expression analyses of selected *AP2-ERF* genes were conducted using qRT-PCR and previously uploaded RNAseq data in root and leaf tissues exposed to salt stress.

## Materials and Methods

### Identification of AP2-ERF sequences and construction of the phylogeny

Comprehensive identification of AP2-ERF amino acid sequences was accomplished by using two approaches. In first step, AP2-ERF encoding amino acid sequences belonging to *Arabidopsis thaliana, Brachypodium distachyon, Carica papaya, Cucumis sativus, Glycine max, Lotus japonicus, Medicago truncatula, Oryza sativa subsp. japonica, Physcomitrella patens, Populus trichocarpa, Sorghum bicolor, Vitis vinifera, and Zea mays *were obtained from plant transcription factor database 3.0 <http://planttfdb.cbi.pku.edu.cn> (Jin et al., 2014[26]) in order to create local blast query sequences. A total of 2422 amino acid sequences was used for BLASTP search at PHYTOZOME v10.1 database <www.phytozome.net> with default parameters (Goodstein et al., 2012[20]) in order to identify genes encode homolog peptides from *P. vulgaris*. In addition to the Blast search, keyword search was also performed in PHYTOZOME v10.3 database <www.phytozome.net>. All the obtained sequences with expected values less than 1.0 were downloaded as FASTA files. Different decrease redundancy tools <web.expasy.org/decrease_redundancy> and elimdupes <http://hcv.lanl.gov/content/sequence/elimdupes/elimdupes.html> were utilized for removing of redundant sequences. All of non-redundant sequences were analyzed for the confirmation of conserved AP2-ERF domain by using InterProScan Sequence Search http://www.ebi.ac.uk/Tools/pfa/iprscan. The miRNA locations on the MtARFs were searched using PMRD database http://bioinformatics.cau.edu.cn/PMRD/. 

The multiple alignments of the final amino acid sequences of PvAP2-ERF proteins were performed with Muscle by using CLC Genomics Workbench 8.0 software (CLC bio, Aarhus, Denmark) with default parameters. Furthermore, alignments of amino acid sequences of AP2-ERF domains from *P. vulgaris and Arabidopsis thaliana *were carried out in the same way. Then, the phylogenetic trees of aligned AP2-ERF proteins were constructed using CLC Genomics Workbench via the Neighbor-Joining (NJ) method with the following parameters: WAG protein substitution model, gamma distribution, and bootstrap (1,000). The constructed phylogenetic trees were visualized with ITOL (Letunic and Bork, 2007[32]). Amino acid composition of the AP2-ERF proteins was calculated by using MEGA 6.0.1. 

### Intron/Exon structure, genome distribution, and motif prediction

In order to illustrate the intron exon structures of *AP2-ERF* genes, DNA and cDNA sequences corresponding to each predicted gene from the *P. vulgaris* genome and the intron distribution pattern information of *PvAP2*-*ERFs* were downloaded from the http://phytozome.jgi.doe.gov/pz/portal.html#!info?alias=Org_Pvulgaris. The gene schematic structure was drawn by the Gene Structure Display Server (GSDS) <http://gsds.cbi.pku.edu.cn/index.php> (Hu et al., 2015[23]). The nucleotide sequences of all *PvAP2-ERF* genes were used as query sequences for BLASTN search of the *P. vulgaris* sequences against the PHYTOZOME v10.1 database using default settings www.phytozome.net for determination of location of these genes on bean chromosomes. Genes were distributed onto bean chromosomes according to their order of physical position (bp), from the first chromosome to eleventh chromosome and finally visualized with a software called MapChart v2.2 <www.wageningenur.nl/en/show/Mapchart.htm>. 

Gene duplications were also studied based on Plant Genome Duplication Database (Tang et al., 2008[57]). *PvAP2-ERF* genes found within the same chromosome were characterized as tandem duplication (Shiu and Bleecker, 2003[53]; Du et al., 2014[14]). For segmental duplications, BLASTP search was performed against all identified peptide sequences of identified PvAP2-ERF in common bean. As potential anchors, top five matches with an e-value (≤ 1e-05) was evaluated and then, MCScan was utilized for the determination of collinear blocks. Finally, the alignment was performed and e-value with ≤ 1e-10 was selected as significant matches (Tang et al., 2008[57]; Du et al., 2014[14]).

Conserved motifs of AP2-ERF transcription factors were detected with web-based sequence analysis tool called as MEME (4.10.1) with the following parameters: optimum width 80-100 amino acids, any number of repetitions of a motif, and maximum number of motifs set at 10. The BLAST search for the resulting motifs in the NCBI and SMART databases was performed to determine their biological context (Sharma et al., 2010[52]).

### Prediction of 3D protein homology, molecular and physicochemical parameters

The isoelectric point (pI), amino acid composition, molecular weight of common bean AP2-ERF deduced proteins and instability indexes were determined using the ProtParam tool http://us.expasy.org/tools/protparam.html. The sub-cellular localization was investigated using the mGOASVM (V2) <http://bioinfo.eie.polyu.edu.hk/mGoaSvmServer2/mGOASVM_v2.html>. The PROSOII program http://mips.helmholtz-muenchen.de/prosoII/prosoII.seam was used to predict the sequence-based solubility of proteins (Smialowski et al., 2012[54]).

For prediction of 3D protein structures of PvAP2-ERF proteins, Phyre2 server (Protein Homology/Analogy Recognition Engine) (Kelley and Sternberg, 2009[29]) was used. Firstly, all PvAP2-ERF protein sequences were searched in the Protein Data Bank (PDB) (Berman et al., 2000[3]) with the default parameters. Then, the data was analyzed in Phyre2 server with 'intensive' mode for identification of the best template having similar sequence and known three-dimensional structure. Finally, predicted protein structures of bean PvAP2-ERF was evaluated in terms of confidence level (> 90 %) and percentage residue level (80 to 100).

### Analyses of the putative PvAP2-ERF promoter region and miRNAs targeting

A total of 180 common bean *AP2-ERF* genes were analyzed in LegumeIP <http://plantgrn.noble.org/LegumeIP/v2> in order to obtain promoter sequences of identified genes. The 2000 bp fragment upstream of the transcriptional start site of *PvAP2-ERFs* was determined as promoter region to identify the *cis*-elements. The identified *cis*-elements of promoter sequences were analyzed by using the PLACE http://www.dna.affrc.go.jp/PLACE/ database. 

Detection of miRNA target genes plays important role for understanding the functions of miRNAs. Bioinformatics and prediction analyses of miRNAs and their target *PvAP2-ERF* genes were performed in the web-based psRNA Target Server <http://plantgrn.noble.org/psRNATarget> with default parameters. Alignment of identified genes with all known plant miRNAs was conducted based on Zhang (2005[67]). Furthermore, miRNA targets were also checked with BLASTX searches with ≤ 1e-10 against *P. vulgaris* EST sequences at NCBI database for confirmation of putative gene homologous.

### Gene ontology (GO) and functional annotation

The functional characterization and annotation of *AP2-ERF* sequences were performed using Blast2GO, which is a sequence-based tool to assign GO terms and annotation for each BLAST hit (Conesa et al., 2005[11]). The GO terms for each of the three main categories (biological process-BP, molecular function-MF and cellular component-CC) were obtained from sequence similarity using the default parameters. Firstly, the common bean amino acid sequences were used as queries in a BLASTP search launched from CLC Genomics Workbench. The BLAST search was performed by using non-redundant (NR) database at NCBI with an e-value of 1xE-5 and top 100 alignments for each sequence was used for further analysis. The resulting Multi Blast data collection was then converted into a Blast2GO Project. GO annotation was carried out by applying the Blast2GO annotation rule, which computes an annotation score for each candidate GO term.

### Prediction of protein-protein interaction network

Since there were no references for common bean interactome data, the protein-protein interaction data from Arabidopsis was used for predicting protein-protein interaction network of PvAP2-ERF members. First, PvAP2-ERFs sequences were used to search Arabidopsis homologous sequences in Inparanoid <http://inparanoid.sbc.su.se/cgi-bin/index.cgi> with default parameters. Secondly, the edge information file (querynw.sif) of Arabidopsis orthologous (*AtAP2-ERFs*) were created via AraNet http://www.functionalnet.org/aranet/, and placed to PvAP2-ERF data to create an edge information file of PvAP2-ERF members. Finally, the protein-protein interaction network of PvAP2-ERF was illustrated with Cytoscape_v3.2.1 (National Institute of General Medical Sciences, MD, USA).

### In silico expression analysis of PvAP2-ERF genes 

To obtain insight into the global expression pattern of common bean* PvAP2-ERF *genes in root and leaf tissues under high salt concentration, Illumina RNA-seq data that was reported previously by Hiz et al. (2014[21]) were utilized. Sequencing reads were obtained from Sequence Read Archive (SRA), following accession numbers of SRR957668 (salt-treated leaf), SRR957667 (control leaf), SRR958472 (salt-treated root) and SRR958469 (control root). The low-quality reads (Phred quality (Q) score < 20) determined by FastQC were trimmed with CLC Genomics Workbench 8.0. To evaluate the expression pattern of root and leaf tissues, high quality reads were mapped to *P. vulgaris* genome (v1.0), downloaded from PHYTOZOME V10.3 database (<http://www.phytozome.net>), by using CLC Genomics Workbench with default parameters. Normalization of the gene expression values were carried out by the reads per kilobase of exon model per million mapped reads (RPKM) algorithm (Mortazavi et al., 2008[41]). To identify differentially expressed *PvAP2-ERF *genes, a FDR-value ≤ 0.001, fold change (RPKM-tr/RPKM-cont) ≥ 2 and the absolute ratio of log2 (RPKM-tr/RPKM-cont) ≥ 1 were used as threshold values. Finally, the heat maps of hierarchical clustering were visualized with PermutMatrix (Caraux and Pinloche, 2005[8]).

### Plant materials, growth conditions, and salt stress applications

The seeds of Ispir, a salt tolerant local common bean variety, were germinated in vermiculite containing plug trays. Seedlings were incubated in growth chamber at 24/ 20 °C cycle under a 16h/8h photoperiod with 350 μmol m^−2^ s^−1^ light intensity, and 50-60 % relative humidity (Dasgan and Koc, 2009[13]) . Hoagland`s solution (Hoagland and Arnon, 1950[22]) was applied to the seedlings in a growth chamber up to trifoliate leaf stage. Salt stress was applied with Hoagland`s solution including 150 mM NaCl for 9 days (Kavas et al., 2015[28]). After this period, root and leaf tissues were collected from salt treated and control plants for gene expression analysis with qRT-PCR. Three separate biological replicates were used.

### RNA extraction and quantitative real-time PCR analysis

RNeasy Plant Mini Kit (Qiagen, Valencia, California, USA) was used to isolate total RNAs of leaf and root tissues collected after 150 mM salt treatment. Isolated total RNAs were quantified by using NanoDrop 2000 UV-VIS spectrophotometer (NanoDrop Technologies, Wilmington, DE, USA) and quality-analyzed using Agilent 2100 Bioanalyzer (Agilent Technologies, Palo Alto, CA, USA). The cDNA was synthesized from 1 µg of DNAse I treated total RNA in a 20 µl reaction volume with RevertAid™ First Strand cDNA Synthesis Kit (Thermo Scientific, USA). 

Tissue-specific expression levels of *PvAP2-ERF* TF family genes that showed differentially upregulated expression pattern in RNA-seq analysis upon salt stress were evaluated with qRT-PCR. qRT-PCR ampliﬁcations were performed by BioRad CFX96 machine (BioRad, Hercules, CA) with default parameters. Primers for the genes of *PvAP2-ERF100, PvAP2-ERF111, PvAP2-ERF119, PvAP2-ERF177, PvAP2-ERF69, PvAP2-ERF70, PvAP2-ERF72, PvAP2-ERF150 *and* PvAP2-ERF53* genes were designed for both leaf and root tissues since they showed higher expression level in RNA-seq analysis. The Skip16 gene was used as an internal standard to calculate relative fold differences based on the comparative C_T_ method (Borges et al., 2012[4]). The primer sequences are listed in Supplementary Table S1. PCR reactions were carried out in 96-well optical reaction plates by using SsoAdvance Universal SYBR Green Supermix (Biorad) according to the manufacturer's protocol. The reaction was set up using 20 ng of cDNA sample as a template. All reactions were repeated three times with triple biological replicates. The data were analyzed using the 2^−ΔΔ^C_T_ method (Livak and Schmittgen, 2001[37]). 

## Results and Discussion

### Identification and chromosomal distribution of PvAP2-ERF genes in P. vulgaris

Completed whole genome sequences of *P. vulgaris* provided an opportunity for the identification of *AP2-ERF* family member genes in common bean for the first time. AP2-ERF family protein sequences from different plant species including *Arabidopsis thaliana, Cucumis sativus, Glycine max, Hordeum vulgare, Medicago truncatula, Nicotiana tabacum, Oryza sativa, Physcomitrella patens, Ricinus cummunis, Solanum lycopersicum, Sorghum bicolor, Triticum aestivum, Vigna radiate, Vitis vinifera *and *Zea mays* were utilized as queries for performing BLAST searches in relevant databases. The putative proteins were examined for the validation of presence of AP2 domain (PF00847; E value < 0.001) using the Pfam database found in CLC Genomics Workbench 8.0. Sequences with insigniﬁcant matches were discarded from further analysis. As a result of this analysis, a total of 180 genes with AP2-ERF domain were identified in common bean (Table 1(Tab. 1), Supplementary Table S2). Accordingly, *P. vulgaris* was found to possess more AP2 and ERF domains as compared to *Cucumis melo, Arabidopdsis thaliana, Glycine max* and *Solanum tuberosum* thereby indicating a variation in number, function and structure among AP2-ERF members belonging to different plant species. *AP2-ERF* gene family members were named as *PvAP2-ERF* according to their order on the chromosomes. Only one gene located in scaffold_91 was called as* PvAP2-ERF-180. *

The ORFs and gene lengths, protein MWs, pI values, stability index, solubility and subcellular localization of these putative genes were analyzed, and results are listed in Table S2. Gene lengths ranged from 363 (*PvAP2*-*ERF155*) to 7445 bp (*PvAP2-ERF128*), MWs from 13.33 (*PvAP2-ERF155*) to 75.70 kDa (*PvAP2-ERF125*) and pI values from 4.56 (PvAP2-ERF94) to 10.06 (PvAP2-ERF73). Cellular localization is often an important factor in determining the protein function (Thamilarasan et al., 2014[58]). Subcellular localization prediction made by mGOASVM suggested that 177 proteins were located in nucleus. It was suggested that TFs are located only in the nucleus in previous studies (Liu et al., 2013[36]). According to an instability index (II), most of the PvAP2-ERF proteins (95 %) were estimated as unstable in a test tube. Solubility of PvAP2-ERF proteins in *Escherichia coli* was evaluated according to their amino acid sequences. According to this evaluation, almost half of the PvAP2-ERF proteins were found as soluble. Based on the average amino acid composition of AP2-ERF proteins, the most abundant amino acid was Ser (S) with the value of 11.80. The average abundance of important salt responsive amino acids, Gly (G) and Pro (P) were found to be 6.28 and 6.00, respectively (Table S3).

**Figure 1:**
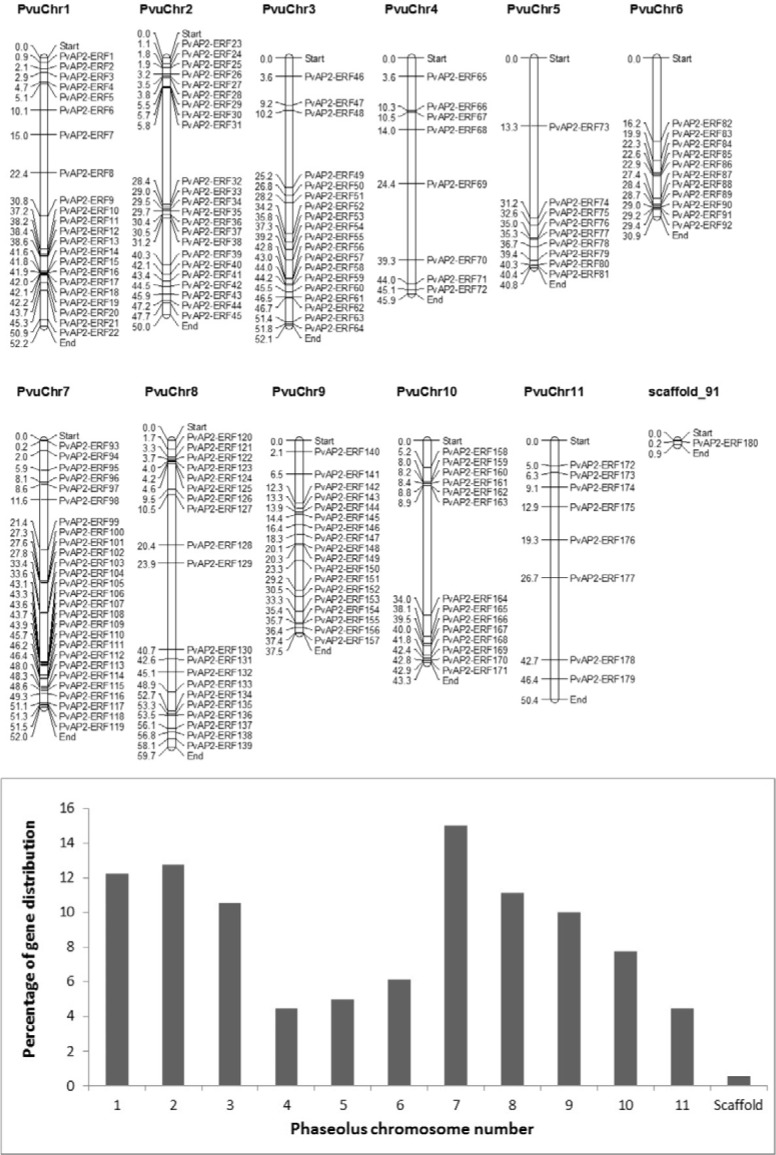
Chromosomal distribution of 180 *PvAP2-ERF* genes. The chromosome number is indicated above each chromosome. Numbers on the left site of each chromosome indicate the physical position (in Mb).The graph below represents percentage of *PvAP2-ERF* gene distribution among 11 Phaseolus chromosome and a scaffold.

It was reported that Glycine-rich proteins have important roles in plants under different abiotic and biotic stress conditions by regulating the gene expression post-transcriptionally (Mousavi and Hotta, 2005[42]).The average abundance of Trp and Cys amino acids in AP2-ERF proteins were 1.67 and 1.29, respectively.Except *PvAP2/ERF-180*, all other *PvAP2-ERF *genes were successfully mapped to eleven chromosomes. The exact position (in kp) of each *PvAP2-ERF *on bean chromosome is shown in Figure 1(Fig. 1). The highest number of *AP2-ERF *genes were found in chromosome 7 with 15 %. Following this, chromosome 2, 1 and 8 contained 23/180 (12.8 %), 22/180 (12.2 %) and 20/180 (11.1 %) *PvAP2-ERF *genes, respectively. Among all, the least gene distribution was observed in chromosome 4 and 11 with 4.4 % (Figure 1(Fig. 1)). It was also realized that different distribution pattern of the *PvAP2-ERF *genes on chromosomes has occurred and gene clusters were accumulated on lower or upper end of the chromosome arms. For example, *PvAP2-ERF *genes located on chromosomes 1, 2, 7, 8 and 10 were distributed on both arms, whereas genes found on chromosomes 3, 5, 6 and 9 appear to be congregated at only lower end of the arm (Figure 1(Fig. 1)).

The AP2-ERF transcription factor genes are one of the most identified gene family members in different plant genomes including 122 genes in *Arabidopsis thaliana *(Nakano et al., 2006[44]), 226 genes in *Brassica oleracea* (Thamilarasan et al., 2014[58]), 248 genes in *Brassica rapa* (Song et al., 2013[56]), 108 genes in *Citrus sinensis* (Ito et al., 2014[25]), 136 genes in *Cucumis melo *(Ma et al., 2015[38]), 103 genes in *Cucumis sativus* (Hu and Liu, 2011[24]), 267 genes in *Daucus carota* (Li et al., 2015[33]), 209 genes in *Eucalyptus grandis* (Cao et al., 2015[7]), 98 genes in *Glycine max* (Zhuang et al., 2008[69]), 209 genes in *Malus domestica* (Girardi et al., 2013[19]), 139 genes in *Oryza sativa* (Nakano et al., 2006[44]), 116 genes in *Phyllostachys edulis* (Wu et al., 2015[63]), 200 genes in *Populus trichocarpa* (Zhuang et al., 2008[69]), 114 genes in *Ricinuscommunis* (Xu et al., 2013[64]), 173 genes in *Salix arbutifolia* (Rao et al., 2015[48]), 171 genes in *Setaria italica* (Lata et al., 2014[30]), 112 genes in *Solanum lypersicon* (Sharma et al., 2010[52]), 155 genes in *Solanum tuberosum* (Charfeddine et al., 2015[9]), 117 genes in *Triticum aestivum* (Zhuang et al., 2011[70]), 132 genes in *Vitis vinifera* (Licausi et al., 2010[34]), 184 genes in *Zea mays* (Du et al., 2014[14]). The first discovery of these family genes at genomic level was accomplished in *Arabidopsis *and *Oryza sativa *in 2006 (Nakano et al., 2006[44]). From that time, characterization of *AP2-ERF *gene family members from different organisms has continued. The density of *PvAP2-ERF *is about 0.3066 which is higher than most of the analyzed plants. *Arabidopsis thaliana* (0.9037) and *Triticum aestivum *(0.0069) had the highest and lowest *AP2-ERF *gene density, respectively (Table S4). We also found similar gene numbers in *P. vulgaris* (180) with *Zea mays* (184) and *Setaria italica* (171). Advent of omics technologies and availability of draft genomes of organisms provide valuable sources for detection of new genes and determination of their function (Baloglu, 2014[2]). Although determination of *AP2-ERF *gene members at genome level are common for different plant species, there is a limited data for genome-wide identification and their characterizations in the common bean genome (Hiz et al., 2014[21]; Kavas et al., 2015[28]). Therefore, it can be concluded that we firstly found 180* AP2-ERF *genes in Phaseolus genome was found in this study for the first time on the basis of Pfam and SMART domain searches.

### Phylogenetic analysis of the AP2-ERF family

**Figure 2:**
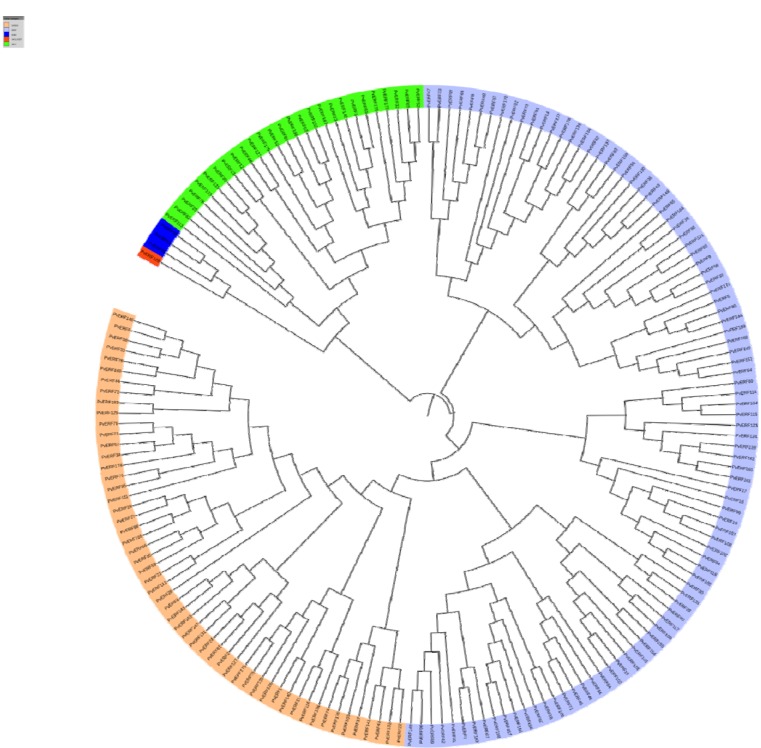
Phylogenetic tree of aligned 180 AP2-ERF subfamily proteins constructed using CLC Genomics Workbench via the Neighbor-Joining (NJ) method. The different classes of PvAP2-ERF proteins are highlighted in different colors.

The classification method of Sakuma et al. (2002[50]) used for AtAP2/ERFs was followed for further classification of PvAP2/ERFs. According to this classification, a total of 149 PvAP2/ERFs with only one domain was grouped into two subfamilies (ERF and DREB) based on the similarity of their amino acid sequences. Like other plants such as Arabidopsis, melon, sweet orange and potato, the ERF and DREB subfamilies are the most dominant form of AP2-ERF transcription factors in common bean. The ERF subfamily factors were divided into six subgroups: B1-B6. The DREB subfamily factors were also subdivided into six groups: A1-A6. A total of 27 factors carrying double AP2-ERF domains were grouped into the AP2 subfamily. Three factors that were identified with a single AP2-ERF domain and a B3 domain were clustered to the RAV subfamily. There was only one soloist gene namely *PvAP2-ERF128* which is ortholog of AT4G13040. Based on the multiple sequence alignment and motif analysis, unrooted tree with 180 PvAP2-ERF domain sequences was constructed (Figure 2(Fig. 2), Supplementary Figure S1). 

Compared with *Arabidopsis thaliana* (122 members), *Cucumis melo* (136 members), *Cucumis sativus* (103 members), *Solanum tuberosum* (155 members), *Solanum lypersicon* (112 members) and *Glycine max* (98 members), the AP2-ERF family seems to have relatively higher number of members in common bean. It is expected result that the number of the AP2-ERF members varied among different species (Table 1(Tab. 1) References in Table 1: Ma et al., 2015[38]; Sharma et al., 2010[52]; Zhang et al., 2008[67]; Nakano et al., 2006[44]). For instance, the number of members in the ERF subfamily ranges from 64 (in melon) to 95 (in common bean), and the number of DREB members ranges from 25 (in potato) to 54 (in common bean). 

### Gene structure and conserved motifs analysis of the AP2-ERF gene family

To gain further information of the structural diversity of common bean *AP2-ERF* genes, we analyzed exon/intron organization within PvAP2-ERF family members (Figure S2). A total of 42 intronless *AP2-ERF* genes were identified, which accounted for 23 % of total *PvAP2-ERFs*. Number of ERF subfamily genes (23 genes) without intron was higher than that of DREB subfamily genes (16). Intron organization and numbers of *AP2-ERF* genes in common bean showed different variation and distribution into different subgroups. AP2 subfamily members had 5-9 introns and almost half of the groups members had seven introns. In contrast to other members of AP2 subfamily, only *PvAP2-ERF179* had five introns (Figure S2). 

The amino acid sequences of the AP2-ERF family members were aligned with multiple sequence alignment tools to determine the phylogenetic relationships between the genes in the common bean AP2-ERF family. A total of 20 consensus amino acids, 3G, 4V, 11G, 16E, 17I, 33R, 35W, 36L, 37G, 45A, 46A, 48A, 49Y, 50D, 51A, 52A, 57G, 60A, 63N and 64F were more than 80 % conserved among the 189 proteins in the PvAP2-ERF family (Figure S3). The alignment indicated that 95 of these genes were located in the ERF subfamily, 27 were in the AP2 subfamily, three were in the RAV subfamily and 54 were in the DREB subfamily. The differences within the subfamilies were further analyzed by examining the conserved motifs using MEME. The members in each subfamily had high sequence similarities (Figure S2). All members of the DREB subfamily and the ERF family contained a WLG motif; however, only a few ERF factors possessed WIG. Most of the members of AP2 subfamily contained YLG motif. In addition to these residues, most of members (more than 85 %) in common bean AP2-ERF family possessed the two featured conserved elements YRG and AYD elements within the AP2-ERF domain region.

### Gene duplications rate of the PvAP2-ERF genes

Segmental and tandem gene duplications play important role for expansion and evolution of large gene families in plants (Baloglu et al., 2014[2]). We have also indicated gene duplication events of *AP2-ERF *genes in common bean. It was observed that segmental gene duplication mainly occurred in the Phaseolus genome rather than tandem duplication event (Table S5). Totally, 103 segmental duplicated *AP2-ERF *genes have been found which accounts for about 57 % (103/180) of total *PvAP2-ERF *genes. In contrast to segmental duplication rate, tandem duplication event has a limited contribution to the gene family expansion. A total of 14 (8 %) tandem duplicate *PvAP2-ERF *genes were detected. Similar results have been observed for in the sorghum, rice, Arabidopsis (Wang et al., 2011[59]), cucumber (Baloglu et al., 2014[2]) and common bean (Kavas et al., 2015[28]) genomes. Our observation might support the idea that segmental duplication of *AP2-ERF *genes in common bean has a major contribution to the expansion of this gene family in Phaseolus genome.

### 3D Protein Homology Modeling 

**Figure 3:**
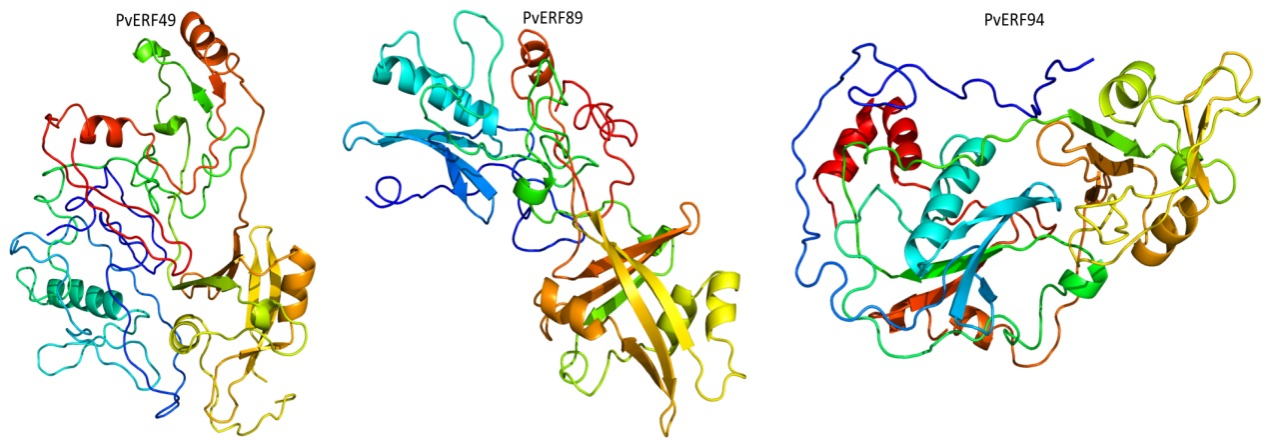
Predicted 3D structures of three selected PvAP2-ERF proteins namely, PvERF49, PvERF89 and PvERF94, modelled at >90 % confidence level by using Phyre2 server.

Firstly, BLASTP search was conducted in the PDB for construction of 3D homology model for PvAP2-ERF proteins. Only three of PvAP2-ERF proteins (PvAP2/ERF-49-89-94) showed higher homology rate. Then, intensive mode of Phyre2 was used for enhancing higher accuracy. The alignment of hidden Markov models is used in this web-based software (Soding, 2005[55]). In addition, Poing which has a new folding simulation mode in Phyre2 has been improved for modelling of proteins regions with no significant homology for known structures. Finally, 3D protein homology modeling of PvAP2/ERF-49-89-94 proteins are predicted at above 90 % confidence and the percentage residue varied from 80 to 100 (Figure 3(Fig. 3)). These results are consistent with the findings of the previous studies in which small number of predicted 3D homology of bHLH proteins (Kavas et al., 2015[28]) and bZIP ones (Baloglu et al., 2014[2]) were found.

The secondary structures were mainly composed of β strand and with small rate of α helices. Thus, all the predicted PvAP2-ERF proteins are considered highly reliable providing preliminary information for understanding the molecular function of AP2-ERF proteins in common bean, as well.

### Identifying miRNAs for PvAP2-ERF gene targets

miRU database was used for identification of *PvAP2-ERF *gene targets with the consideration of two important parameters (Zhang, 2005[67]). The first one is the maximum expectation, which is the threshold of the score. The default cut-off threshold was adjusted to 3.0. If this score is greater than the threshold, a miRNA/target site pair has been omitted. UPE is the second one, which is defined as maximum energy to unpair the target site. Nearly all of *PvAP2-ERF *genes targeted by 44 different plant species` miRNAs were identified in this study. The most abundant target genes are *PvAP2/ERF*-20-25-62-78-113-173. Furthermore, miR156, miR172 and miR838 are the most important miRNAs found by targeting and BLAST analysis (Table S6).

MicroRNAs (abbreviated miRNA) are a small non-protein coding RNA molecule (aprox. 21 nucleotides) found in plants, animals, and some viruses, which regulate gene expression at post-transcriptional level (Eldem et al., 2013[15]). They play functional roles in plant development and stress response to biotic and abiotic environmental factors. Based on *in silico* analysis of *PvAP2-ERF *gene targets, miR172 appeared in all selected plant species miRNAs. The crucial role of miR172 has been described for soybean (Yan et al., 2013[66]), common bean-rhizobia symbiosis (Wang et al., 2014[60]) and common bean (Nova-Franco et al., 2015[45]). In common bean, miR172c indirectly regulates the expression of the transcription factors NF-YA1, NSP2 and CYCLOPS as well as the gene FLOT2, all of which are essential regulators of early stages of the symbiosis (Nova-Franco et al., 2015[45]). There is a close relationship between miR156 and miR172, which coordinated developmental timing in Arabidopsis. miR156 represses the expression of SPL transcription factors whereas miR172 acts downstream of miR156 to promote adult epidermal identity in Arabidopsis (Wu et al., 2009[62]). Additionally, miR156 is responsible for regulation of miR172 expression via SPL9 and SPL10, which immediately promotes the transcription of miR172b (Wu et al., 2009[62]). Therefore, identification of miRNAs and their *PvAP2-ERF *target genes might provide valuable information for determination of possible involvement of miRNAs in diverse physiological mechanisms. 

### The interaction network of AP2/ERF genes in common bean

**Figure 4:**
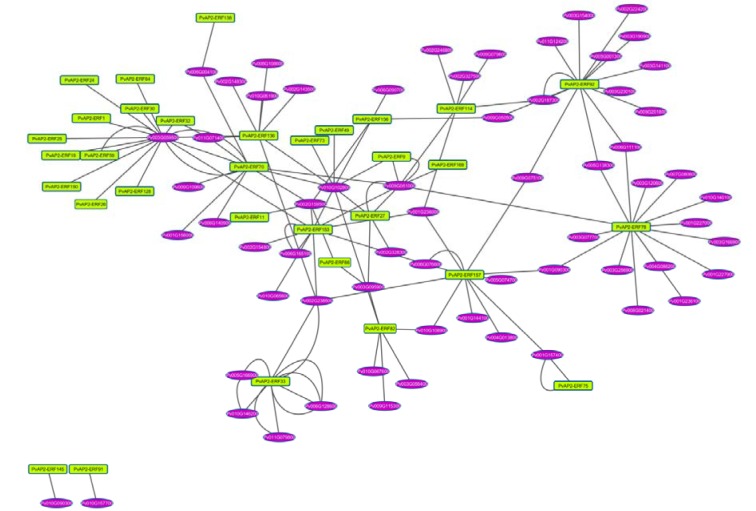
Figure 4: The interaction network of *AP2-ERF* genes in common bean according to the orthologs in Arabidopsis. Ellipse in purple represent common bean genes and rectangle in yellow color *PvAP2-ERFs*.

Plant growth is a developmental process that multiple genes and their interaction genes are involved in. The Arabidopsis Interactions Viewer is a database that provides complex biomolecular and pathway information of thousands of interactions between proteins. In order to further understand the interactions between *AP2/ERFs* and other genes in common bean, an interaction network was constructed according to the orthologs in Arabidopsis (Figure 4(Fig. 4)). There were 32 *AP2/ERF* genes that showed interactions with other genes in common genome. A total of 71 gene pairs with the value of Pearson correlation coefficient (PCC) over than zero were positive correlations, whereas 33 gene pairs with the value of PCC less than zero were negative correlations. Moreover, 27 gene pairs could not be calculated. The transcription factor PvAP2-ERF78, an ortholog of At2G28550, was potentially interacted with at least 15 proteins, indicating that it has very important role in transcriptional level regulation (Figure 4(Fig. 4)). However, twelve AP2-ERF factors were regulated by a common bean protein Phvul.003G059500, an important protein for response to osmotic and oxidative stress. 

### Analysis of the putative promoter regions and GO terms of the AP2-ERF gene subfamily 

DNA sequences located at upstream of genes in the promoter region and served as transcription factor (TF)-binding sites are called as *cis*-regulatory elements. It was reported that they have important roles in controlling tissue-speciﬁc and stress-responsive gene expression (Le et al., 2012[31]). There are many studies showing that multi-stimulated genes are closely correlated with *cis*-regulatory elements in their promoter sequences (Fang et al., 2008[16]). To increase knowledge about the transcriptional control and potential roles of *AP2-ERFs*, cis-elements in their promoter sequences were predicted using plant promoter database (PLACE). By searching the *PvAP2-ERF* promoter sequences in the plant promoter PLACE database, a number of potential regulatory sequences corresponding to *cis*-acting elements related to tissue speciﬁc gene expression, abiotic and biotic stress responses and hormone response were predicted. According to this putative promoter analysis, promoter region of AP2-ERFs was found to contain MYB, MYC, WBOX, and WRKY binding elements (Table S7). These interesting results reveal that MYB, MYC and WRKY transcription factors can regulate the expression of *AP2-ERF* genes. The fact that many cis-elements related to various developmental process suggests that these transcription factors are controlled by different signaling pathways involved in response to several stress conditions. In addition to these elements, many abiotic stress related cis-elements such as S000176, S000028, S000144, S000408 and S000415 for drought stress, S000453 for salt stress, S00030 for heat stress, S000407 for cold stress and S000457 for wound stress were found widely in the promoter regions of *AP2-ERFS* in common bean. In this context, *PvAP2-ERF1* had up to 23 light-responsive and tissue-specific activation of phenylpropanoid biosynthesis genes elements (S000144). This clearly showed that AP2-ERFS superfamily transcription factors might respond to abiotic stress and have potential functions in enhancing abiotic stress resistance. 

**Figure 5:**
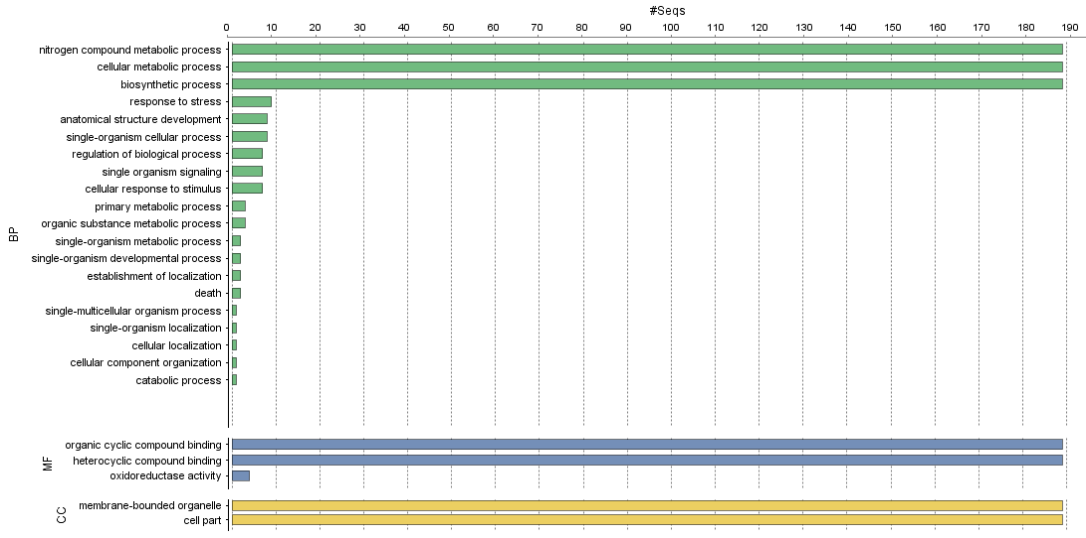
Gene ontology (GO) and functional annotation of PvAP2-ERF proteins using Blast2GO. CC (cellular compartment) was represented as yellow bars, MF (molecular function) was indicated as purple bars and BP (biological process) was given as green bars on the graph.

The GO terms of the *PvAP2-ERF* genes were annotated according to their biological processes (BP), molecular functions (MF) or involvement as cellular components (CC). The *PvAP2-ERF* genes belonged to different biological processes, cellular components and molecular functions were shown in Figure 5(Fig. 5).

The maximum numbers of *PvAP2-ERF* genes were involved in biological processes, including nitrogen compound metabolic process, cellular metabolic processes and biosynthetic process. In the context of biological process, most of the *PvAP2-ERF* genes showed both organic cyclic and heterocyclic compound binding activity. 

### AP2-ERF gene expression profiling

**Figure 6:**
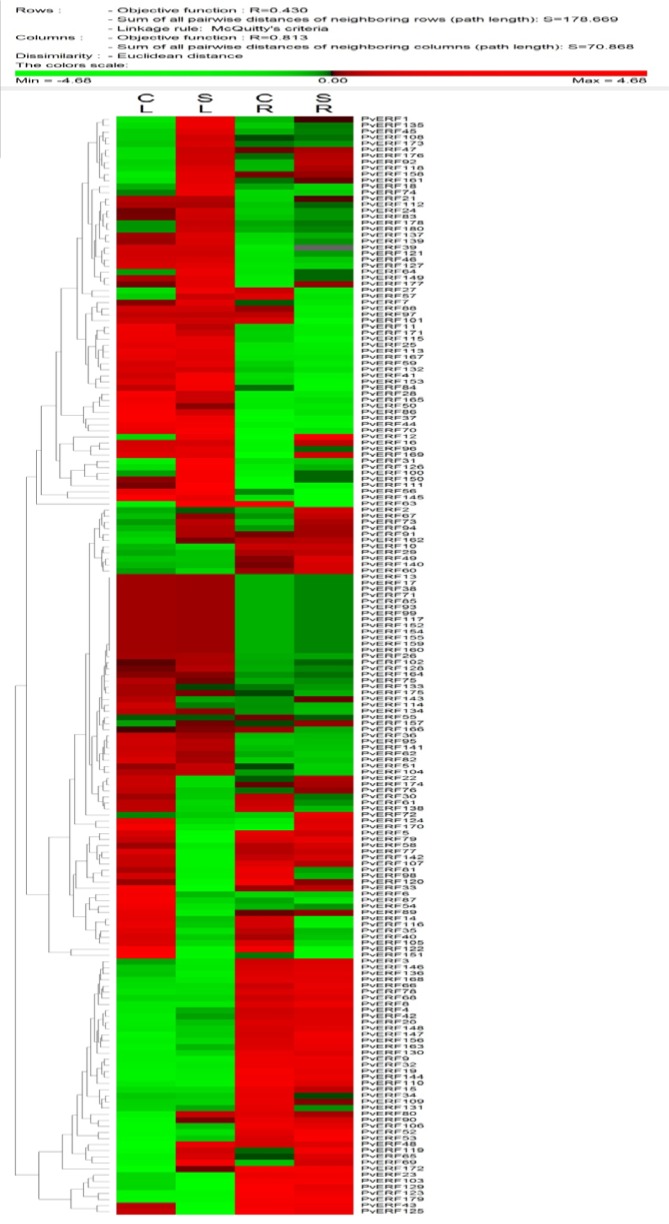
Representation of Heat map and hierarchical clustering of all *PvAP2-ERF* genes in leaves and roots upon high salinity treatment. The RPKM values retrieved from published RNA-seq data were log2 transformed and the heat map generated using PermutMatrix . CL; control leaf, SL; salt leaf, CR; control root, ST; salt root. Color scale in the dendrogram represents relative expression levels: green represents low level (down regulated of genes), red indicates high level (up-regulated ones) and black represents unchanged gene expression level. The color scales for fold-change values are shown above the dendogram.

In silico expression analysis *AP2-ERF* genes was carried out with two different tissue samples of common bean. According to RNA-seq analysis results , expression of 12 *AP2-ERF* genes (*PvAP2-ERF117, PvAP2-ERF13, PvAP2-ERF152, PvAP2-ERF154, PvAP2-ERF155, PvAP2-ERF159, PvAP2-ERF160, PvAP2-ERF17, PvAP2-ERF38, PvAP2-ERF71, PvAP2-ERF85, PvAP2-ERF99*) were not detected in reads belong to either root and leaf tissues based on normalized RPKM values. A total of 78 *PvAP2-ERF *genes were differentially expressed in at least one tissue. Among the 180 *PvAP2-ERFs*, 35 *PvAP2-ERF*s including *PvAP2-ERF27, PvAP2-ERF1, PvAP2-ERF119, PvAP2-ERF126, PvAP2-ERF69, PvAP2-ERF111 and PvAP2-ERF3 *were differentially upregulated in leaf tissue. In root tissue, a total of 10 *PvAP2-ERFs *including *PvAP2-ERF150, PvAP2-ERF72, PvAP2-ERF119, PvAP2-ERF96, PvAP2-ERF53 *showed higher expression pattern. Interestingly, six of total *PvAP2-ERF*s (*PvAP2-ERF*69, *PvAP2-ERF*119, *PvAP2-ERF*100, *PvAP2-ERF*53, *PvAP2-ERF*70 and *PvAP2-ERF*177) were highly expressed both in leaf and root tissues. Genome-wide expression analysis also showed that there were a total of 68 up-regulated *PvAP2-ERF*s and 56 down-regulated *PvAP2-ERF*s in leaf tissues after salt stress (Table S8). Likewise, there were 51 up-regulated *PvAP2-ERF*s genes and 84 down-regulated *PvAP2-ERF*s genes in root tissues. An expression profile of all identified *PvAP2-ERF*s genes was shown as heat map in Figure 6(Fig. 6).

### Gene expression levels of salt responsive AP2-ERFs transcription factor family genes

**Figure 7:**
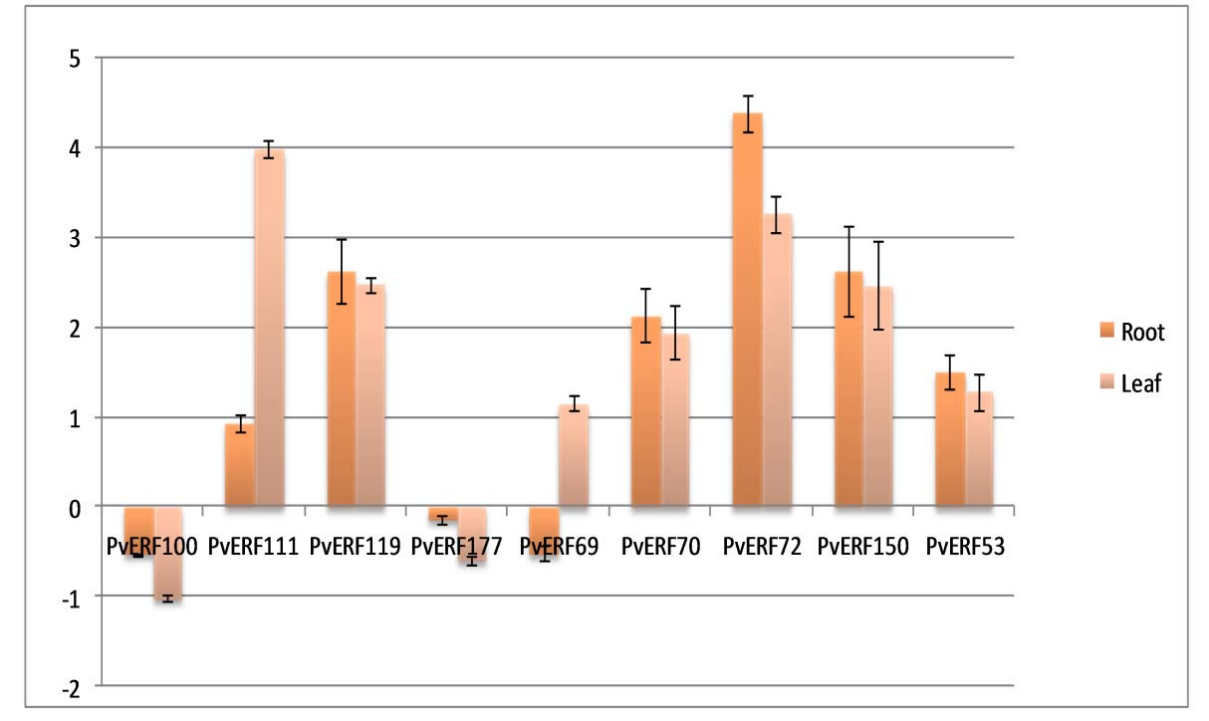
Relative expression levels of nine selected PvAP2-ERF genes in salt stressed leaf and root tissues as compared to the control by qRT-PCR . Relative expression represents log2 expression values. Standard error (SE) was determined from three independent biological replicates.

Expression levels of 9 PvAP2-ERFs family members (PvAP2-ERF100, PvAP2-ERF111, PvAP2-ERF119, PvAP2-ERF177, PvAP2-ERF69, PvAP2-ERF70, PvAP2-ERF72, PvAP2-ERF150, PvAP2-ERF53) in salt-stressed leaf and root tissues were determined. qRT-PCR results revealed that seven of the nine selected genes were up-regulated in common bean leaf tissues after salt treatment (Figure 7(Fig. 7)). Among them, PvAP2-ERF111, PvAP2-ERF119, PvAP2-ERF72 and PvAP2-ERF150 showed relatively higher expression level when compared to control sample. Therefore, we validated 7 PvAP2-ERFs in leaf tissue by using qRT-PCR. Likewise, the expression of most of genes selected after RNA-seq analysis were also validated with qRT-PCR in root tissue. According to these results, PvAP2-ERF111, PvAP2-ERF119, PvAP2-ERF72 and PvAP2-ERF150 showed two fold change in expression pattern when compared to expression level of the control tissue. Three of the selected PvAP2-ERFs (PvAP2-ERF100, PvAP2-ERF69 and PvAP2-ERF177) showed down-regulated gene expression pattern after salt treatment in root tissues. These results indicated that PvAP2-ERF genes were generally affected by salt stress, indicating, that they are involved in salt stress signal transduction by a positive regulation pathway. 

## Conclusion

The AP2-ERF TFs have crucial roles in regulation of different plant processes such as growth, development and stress responses. Due to the theirs important functions, they have been subjected to comprehensive analysis in different plants. The present study is the first report related with identification and characterization of the AP2-ERF transcription factors in common bean. A total of 180 putative common bean genes belonging to AP2-ERF superfamily were identified by using bioinformatics tools. Additionally, the characterization of genomic structures, chromosomal locations, promoter analysis, protein interaction, sequence homologies of all common bean *AP2-ERF* TF genes were also undertaken. The expression analysis of selected *AP2-ERF* genes was conducted using qRT-PCR and previously uploaded RNAseq data in root and leaf tissues exposed to salt stress. 

Results of the present extensive study would assist to understanding of roles of AP2-ERF family members in salt tolerant common bean cultivar and may also provide functional gene resources for genetic engineering approaches.

## Acknowledgements

This research was supported by a Research Fund of Ondokuz Mayıs University (PYO.ZRT.1902.13.001). The authors would also like to thank Dr. Yıldız Daşgan for providing the seed of salt resistant common bean cultivar.

## Declaration of interest

The authors report no conflicts of interest.

## Supplementary Material

Supplementary material

## Figures and Tables

**Table 1:**
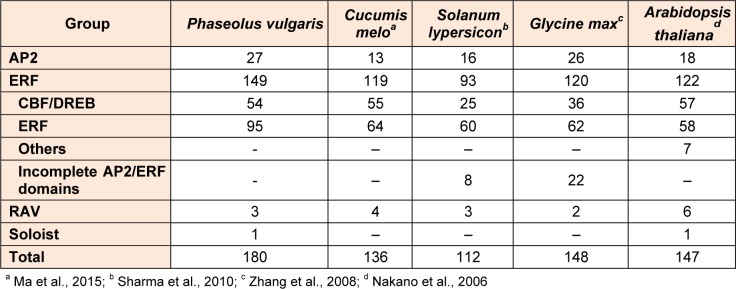
Summary of Ap2-ERF superfamily in common bean in compassion with *Cucumis melo, Solanum lypersicon, Glycine max, Arabidopsis thaliana*
