# Supplementary material for: Genome-wide investigation and expression analysis of AP2-ERF gene family in salt tolerant common bean
Source: EXCLI J. 2015 Nov 27;14:1187–206. doi: 10.17179/excli2015-600 (PMC4849109; doi:10.17179/excli2015-600)
Supplement: Supplementary material [file EXCLI-14-1187-s-001.pdf]

EXCLI Journal 2015;14:1187-1206 – ISSN 1611-2156
Received: September 26, 2015, accepted: November 01, 2015, published: November 27, 2015

Supplementary material to:

GENOME-WIDE INVESTIGATION AND EXPRESSION ANALYSIS OF *AP2-ERF* GENE FAMILY IN SALT TOLERANT COMMON BEAN

Musa Kavas[1*], Aslıhan Kurt Kızıldoğan[1], Gökhan Gökdemir[1], Mehmet Cengiz Baloğlu[2]

[1] Ondokuz Mayıs University, Faculty of Agriculture, Department of Agricultural Biotechnology, Samsun, Turkey
[2] Kastamonu University, Faculty of Engineering and Architecture, Department of Genetics and Bioengineering, Kastamonu, Turkey
* Corresponding author: Musa Kavas, Ondokuz Mayıs University, Faculty of Agriculture, Department of Agricultural Biotechnology, Samsun, Turkey; Tel: +903623121919-1158; Fax: +903624576034; E-mail: musa.kavas@omu.edu.tr

http://dx.doi.org/10.17179/excli2015-600

This is an Open Access article distributed under the terms of the Creative Commons Attribution License (http://creativecommons.org/licenses/by/4.0/).

EXCLI Journal 2015;14:1187-1206 – ISSN 1611-2156
Received: September 26, 2015, accepted: November 01, 2015, published: November 27, 2015

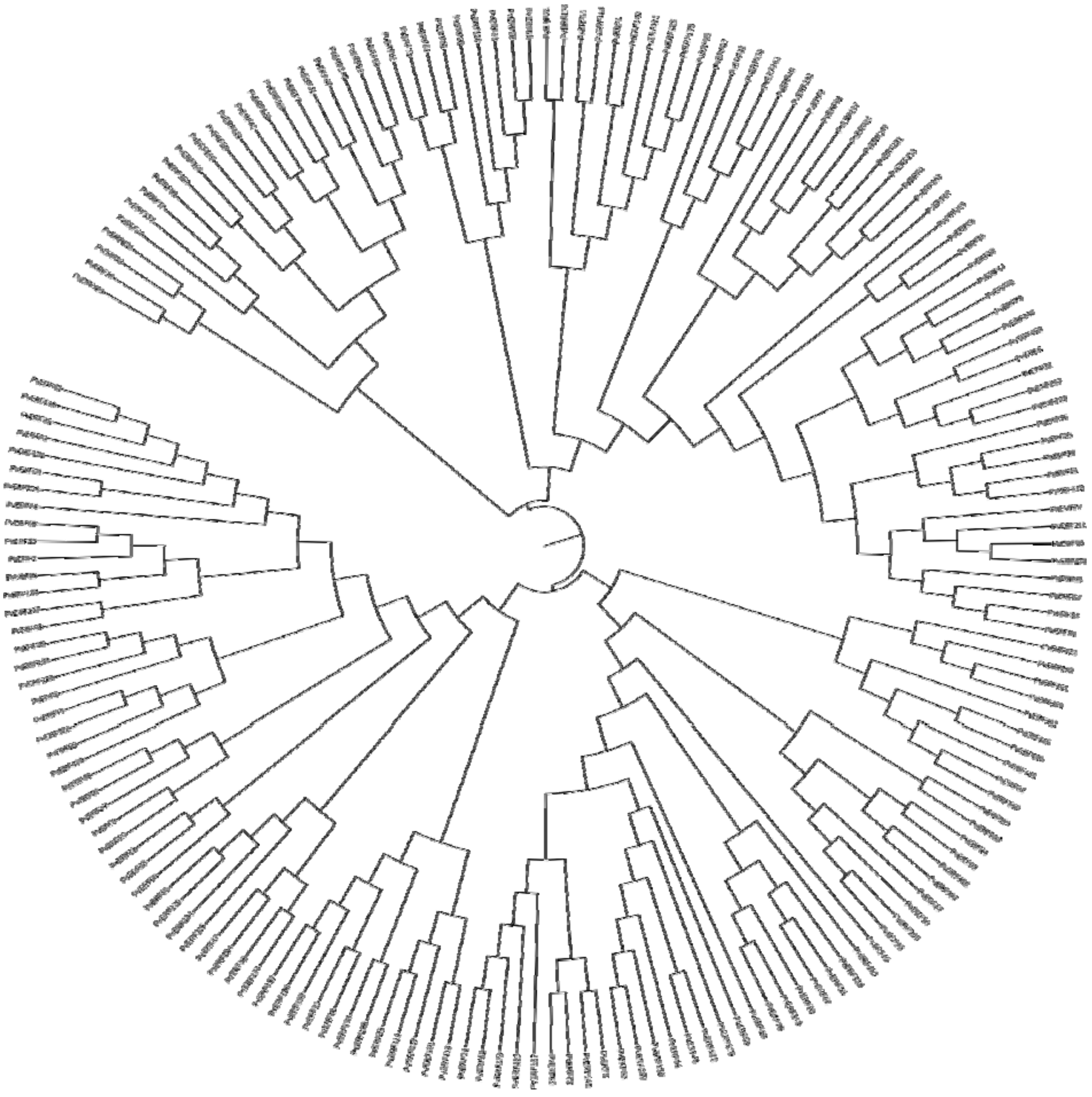

Figure S1: Phylogenetic tree constructed by the neighbor-joining method using AP2 family transcription factor domains in common bean and Arabidopsis. The numbers are bootstrap values based on 1000 iterations.

EXCLI Journal 2015;14:1187-1206 – ISSN 1611-2156
Received: September 26, 2015, accepted: November 01, 2015, published: November 27, 2015

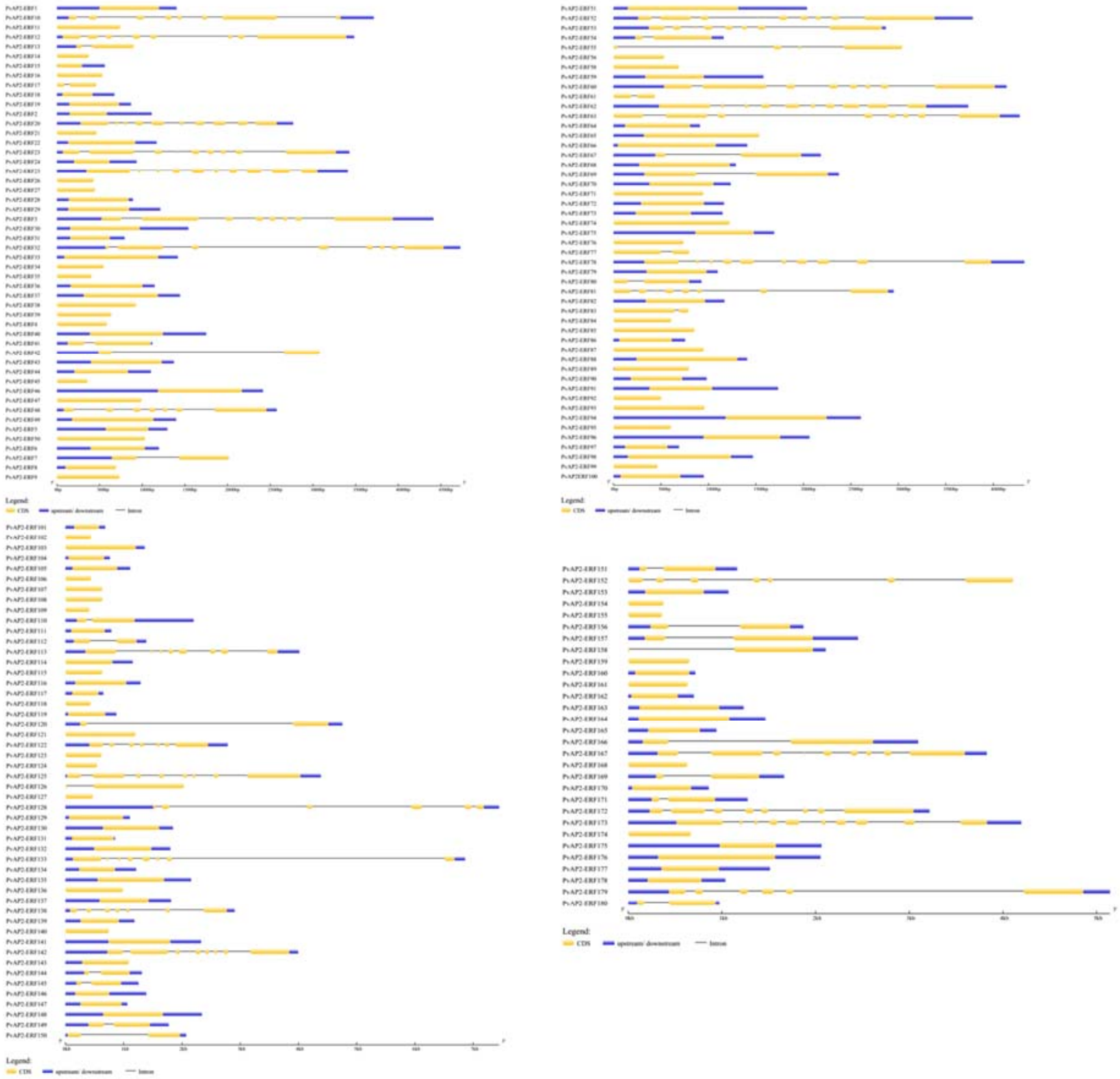

Figure S2: Structural analysis of 180 *PvAP2-ERF* genes drawn by the Gene Structure Display Server (GSDS). CDSs are represented by yellow boxes while blue ones indicate upstream and downstream region. Black lines represent introns.

EXCLI Journal 2015;14:1187-1206 – ISSN 1611-2156
Received: September 26, 2015, accepted: November 01, 2015, published: November 27, 2015

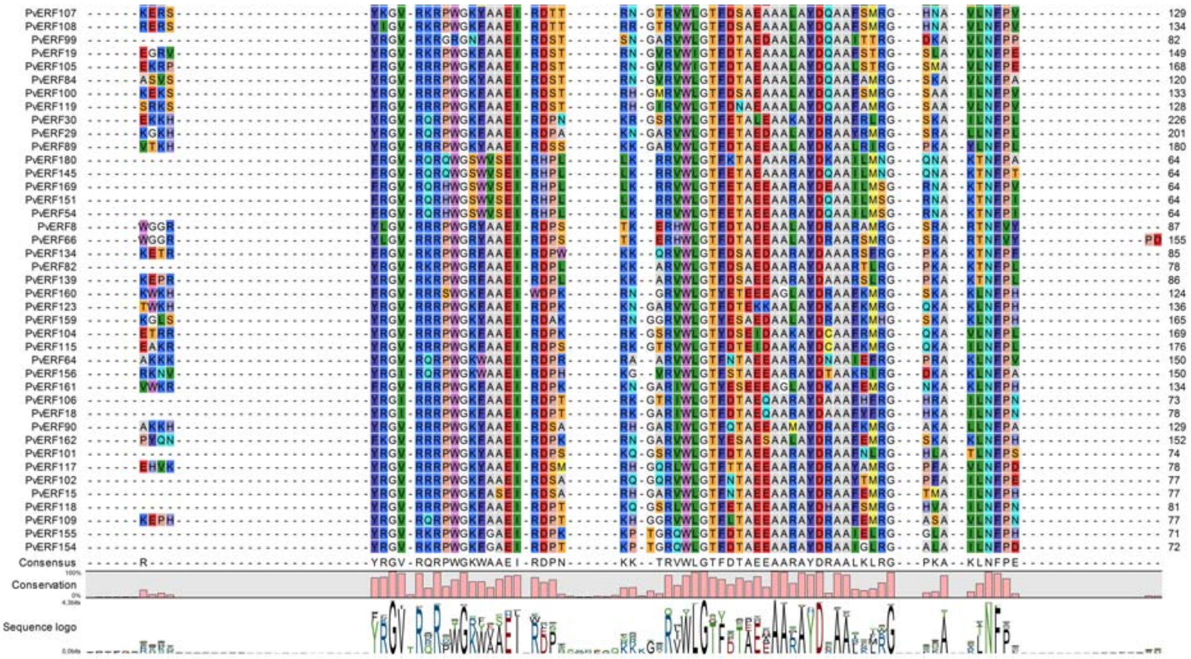

Figure S3: Multiple alignment of amino acid sequences of PvAP2-ERF proteins. It was performed with Muscle by using CLC Genomics Workbench 8.0 software. The conserved motifs belonging to AP2-ERF TFs in common bean were analysed using MEME.

Table S1: Primer sequences of *PvAP2-ERF* genes for qRT-PCR expression analysis

	Primer	Forward	Reverse	NCBI
PvERF69	PHAVU_004G092100g	CTTGGCACATTTGACACTGA	AGAGGTGGAACTGCTCTAAC	XM_007151923.1
PvERF119	PHAVU_007G273000g	ACATGGATTGCTCACTCTCT	TTCTTCGTCTTCTTTGCAGC	XM_007145782.1
PvERF100	PHAVU_007G127800g	CGCAAAAGGAGAAGTCCTAC	GTGTGGAGTCCCTAATCTCC	XM_007144028.1
PvERF53	PHAVU_003G153100g	ATCTCGGAACTTACGCTACC	TGTTGGTGTTGTTATTGGGC	XM_007154791.1
PvERF70	PHAVU_004G122000g	CAAATGGGTTTGTGAGGTGA	GCAAAGTTGAGACACGCATA	XM_007152281.1
PvERF177	PHAVU_011G125200g	CACCAATTCCCTTTTCGGTT	GAGCCAGGTTAAGATCGAGA	XM_007132732.1
PvERF111	PHAVU_007G222500g	GAGGAGGTGTTTAACATGCC	TCTACTGTGGTTTCCCCATC	XM_007145179.1
PvERF150	PHAVU_009G161000g	CACCACTAGCAATGACCAAG	CCAGCAGTACCCATGATTTG	XM_007137787.1
PvERF72	PHAVU_004G169800g	GTCCACTTCCAAGAAACGAG	GGATTTCCGACACCCATTTT	XM_007152840.1
	Skip16	CACCAGGATGCAAAAGTGG	ATCCGCTTGTCCCTTGAAC	XM_007131876.1

Table S2: The molecular and physicochemical parameters of *PvAP2-ERF* family genes in common bean

ID	Phytozome Identfier	Chromosomes	Start and end positions (bp)	Length (bp)	CDS (bp)	Protein length (aa)	pI	Molecular weight (Da)	Instability index	Stable or unstable	Solubility/Score	NCBI Accession number
PvAP2-ERF1	Phvul.001G010400	Chr01	931454-932855	1402	798	265	4,93	29057,1	45,19	unstable	insoluble; 0.574	XM_007160647
PvAP2-ERF2	Phvul.001G023700	Chr01	2101326-2102436	1111	537	178	9,61	20485	41,69	unstable	insoluble; 0.503	XM_007160808
PvAP2-ERF3	Phvul.001G031200	Chr01	2907733-2912245	4513	2064	687	6,64	75432,4	49,51	unstable	soluble; 0.608	XM_007160891
PvAP2-ERF4	Phvul.001G044500	Chr01	4680371-4681060	690	690	229	4,78	25706,2	40,08	unstable	soluble; 0.649	XM_007161059
PvAP2-ERF5	Phvul.001G046900	Chr01	5091472-5092865	1394	600	199	8,86	22372,2	63,16	unstable	insoluble; 0.484	XM_007161090
PvAP2-ERF6	Phvul.001G073800	Chr01	10050549-10051844	1296	735	244	5,77	26835	49,26	unstable	insoluble; 0.571	XM_007161432
PvAP2-ERF7	Phvul.001G084000	Chr01	14986698-14988815	2118	981	326	6,53	36576,4	62,9	unstable	insoluble; 0.329	XM_007161550
PvAP2-ERF8	Phvul.001G099700	Chr01	22388801-22389496	696	696	231	5,24	25456,7	49,83	unstable	insoluble; 0.501	XM_007161743
PvAP2-ERF9	Phvul.001G111800	Chr01	30786425-30787261	837	837	278	6,19	30646	43,73	unstable	insoluble; 0.460	XM_007161894
PvAP2-ERF10	Phvul.001G131300	Chr01	37223732-37227544	3813	1254	417	8,85	46658	54,45	unstable	insoluble; 0.546	XM_007162122
PvAP2-ERF11	Phvul.001G136100	Chr01	38195399-38196241	843	843	280	5,13	31503	55,59	unstable	soluble; 0.659	XM_007162182
PvAP2-ERF12	Phvul.001G137500	Chr01	38448464-38452047	3584	1845	614	4,98	68086,8	40,63	unstable	insoluble; 0.454	XM_007162197
PvAP2-ERF13	Phvul.001G137600	Chr01	38463292-38464293	1002	657	218	4,95	24504,2	38	stable	insoluble; 0.419	XM_007162198
PvAP2-ERF14	Phvul.001G157600	Chr01	41586154-41586630	477	477	158	9,88	17846,5	53,47	unstable	insoluble; 0.434	XM_007162443
PvAP2-ERF15	Phvul.001G160100	Chr01	41963965-41964626	662	393	130	6,83	14743,7	43,74	unstable	soluble; 0.652	XM_007162473
PvAP2-ERF16	Phvul.001G160200	Chr01	41973984-41974619	636	636	211	9,39	23643,6	47,71	unstable	insoluble; 0.516	XM_007162474
PvAP2-ERF17	Phvul.001G160300	Chr01	41988449-41989014	566	513	170	4,72	19198,6	61,82	unstable	insoluble; 0.452	XM_007162475
PvAP2-ERF18	Phvul.001G160400	Chr01	42010504-42011278	775	453	150	6,29	16476,9	43,86	unstable	soluble; 0.823	XM_007162476
PvAP2-ERF19	Phvul.001G160500	Chr01	42037682-42038650	969	684	227	4,94	25611,2	42,74	unstable	soluble; 0.706	XM_007162477
PvAP2-ERF20	Phvul.001G174400	Chr01	43723288-43726156	2869	1380	459	6,23	50414,7	49,98	unstable	insoluble; 0.550	XM_007162655
PvAP2-ERF21	Phvul.001G187100	Chr01	45289609-45290175	567	567	188	5,02	20166,4	48,7	unstable	soluble; 0.708	XM_007162797
PvAP2-ERF22	Phvul.001G251200	Chr01	50886895-50888163	1269	888	295	5,45	32793,6	45,74	unstable	insoluble; 0.538	XM_007163576
PvAP2-ERF23	Phvul.002G009100	Chr02	1058028-1061555	3528	1794	598	6,88	67205,9	58,1	unstable	insoluble; 0.580	XM_007156629
PvAP2-ERF24	Phvul.002G016700	Chr02	1837177-1838211	1035	516	171	8,6	18862,2	57,28	unstable	soluble; 0.631	XM_007156715
PvAP2-ERF25	Phvul.002G016900	Chr02	1860815-1864323	3509	1515	504	7,72	55438,1	56,76	unstable	soluble; 0.707	XM_007156717
PvAP2-ERF26	Phvul.002G035100	Chr02	3499002-3499535	534	534	177	4,97	19497,4	67,56	unstable	soluble; 0.747	XM_007156936
PvAP2-ERF27	Phvul.002G035900	Chr02	3545434-3545982	549	549	182	5,71	20227,7	53,55	unstable	insoluble; 0.487	XM_007156945
PvAP2-ERF28	Phvul.002G036000	Chr02	3561530-3562521	992	801	266	6,12	30003,8	60,57	unstable	soluble; 0.694	XM_007156946
PvAP2-ERF29	Phvul.002G055700	Chr02	5668036-5669347	1312	813	270	6,76	29405	56,74	unstable	insoluble; 0.570	XM_007157197
PvAP2-ERF30	Phvul.002G055800	Chr02	5691260-5692900	1641	915	304	8,45	33907,1	50,65	unstable	insoluble; 0.549	XM_007157198
PvAP2-ERF31	Phvul.002G056800	Chr02	5810982-5811876	895	564	187	9,28	20656,5	43,05	unstable	soluble; 0.667	XM_007157208
PvAP2-ERF32	Phvul.002G146400	Chr02	28359488-28364314	4827	1512	503	6,55	55023,5	42,06	unstable	insoluble; 0.580	XM_007158299
PvAP2-ERF33	Phvul.002G149500	Chr02	28972219-28973736	1518	1203	400	6,43	44372	54,56	unstable	insoluble; 0.578	XM_007158332
PvAP2-ERF34	Phvul.002G153900	Chr02	29528341-29528991	651	651	216	5,11	24572,3	77,64	unstable	insoluble; 0.525	XM_007158394
PvAP2-ERF35	Phvul.002G154000	Chr02	29572213-29572719	507	507	168	6,42	18511,9	55,04	unstable	insoluble; 0.549	XM_007158395
PvAP2-ERF36	Phvul.002G162500	Chr02	30433762-30435007	1246	942	313	5,44	35023,9	50,33	unstable	soluble; 0.639	XM_007158496
PvAP2-ERF37	Phvul.002G163700	Chr02	30545248-30546790	1543	969	322	8,7	35266,6	41,85	unstable	soluble; 0.600	XM_007158510
PvAP2-ERF38	Phvul.002G168900	Chr02	31177497-31178525	1029	1029	342	5,03	38663,8	65,31	unstable	soluble; 0.676	XM_007158569
PvAP2-ERF39	Phvul.002G237300	Chr02	40301361-40302101	741	741	246	8,27	27875,3	31,33	stable	insoluble; 0.327	XM_007159370
PvAP2-ERF40	Phvul.002G254500	Chr02	42080727-42082576	1850	957	318	5,41	35548,7	44,79	unstable	insoluble; 0.496	XM_007159576
PvAP2-ERF41	Phvul.002G267800	Chr02	43308758-43309976	1219	957	318	6,73	35365,6	65,52	unstable	soluble; 0.772	XM_007159732

PvAP2-ERF42	Phvul.002G281300	Chr02	44487105-44490185	3081	681	226	9,92	25017	67,25	unstable	soluble; 0.774	XM_007159890
PvAP2-ERF43	Phvul.002G295700	Chr02	45929067-45930538	1472	930	309	5	34265	53,76	unstable	soluble; 0.642	XM_007160075
PvAP2-ERF44	Phvul.002G310200	Chr02	47177131-47178333	1203	729	242	4,87	26154,9	54,14	unstable	soluble; 0.856	XM_007160240
PvAP2-ERF45	Phvul.002G315900	Chr02	47666399-47666860	462	462	153	8,78	17335,5	62,78	unstable	soluble; 0.777	XM_007160308
PvAP2-ERF46	Phvul.003G035500	Chr03	3558866-3561380	2515	1083	360	4,71	39370,5	50,93	unstable	soluble; 0.632	XM_007153379
PvAP2-ERF47	Phvul.003G064800	Chr03	9171392-9172486	1095	1095	364	6,67	40165,7	58,54	unstable	insoluble; 0.578	XM_007153724
PvAP2-ERF48	Phvul.003G069000	Chr03	10190940-10193616	2677	1206	404	9,34	45576,7	60,13	unstable	soluble; 0.604	XM_007153776
PvAP2-ERF49	Phvul.003G102500	Chr03	25181566-25183062	1497	1053	350	5,55	38898,6	55,54	unstable	insoluble; 0.382	XM_007154181
PvAP2-ERF50	Phvul.003G107900	Chr03	26813958-26815094	1137	1137	378	9,87	43617	32,92	stable	insoluble; 0.454	XM_007154248
PvAP2-ERF51	Phvul.003G111800	Chr03	28178527-28180564	2038	1164	387	8,96	42253,5	40,55	unstable	soluble; 0.654	XM_007154292
PvAP2-ERF52	Phvul.003G144500	Chr03	34150168-34153951	3784	1581	526	8,55	56410,6	42,69	unstable	soluble; 0.639	XM_007154688
PvAP2-ERF53	Phvul.003G153100	Chr03	35757558-35760426	2869	1107	368	6,68	42544,2	49,54	unstable	insoluble; 0.598	XM_007154791
PvAP2-ERF54	Phvul.003G165000	Chr03	37339186-37340346	1161	693	230	6,39	26177,1	66,1	unstable	soluble; 0.683	XM_007154944
PvAP2-ERF55	Phvul.003G180000	Chr03	39171521-39174562	3042	759	252	9,43	28553,7	42,94	unstable	insoluble; 0.445	XM_007155115
PvAP2-ERF56	Phvul.003G212700	Chr03	42784293-42784829	537	537	178	4,95	19308,6	56,04	unstable	soluble; 0.687	XM_007155504
PvAP2-ERF57	Phvul.003G212800	Chr03	42804542-42805711	1170	882	293	5,26	32295	54,7	unstable	insoluble; 0.405	XM_007155505
PvAP2-ERF58	Phvul.003G222600	Chr03	44044998-44045690	693	693	230	5,66	25399,8	47,68	unstable	soluble; 0.787	XM_007155625
PvAP2-ERF59	Phvul.003G223600	Chr03	44193652-44195231	1580	618	205	4,84	22308,8	59,27	unstable	insoluble; 0.532	XM_007155636
PvAP2-ERF60	Phvul.003G232600	Chr03	45498765-45502904	4140	1980	659	6,27	73664,4	57,59	unstable	insoluble; 0.575	XM_007155737
PvAP2-ERF61	Phvul.003G241700	Chr03	46504117-46504556	440	381	126	9,2	13914,3	43,37	unstable	insoluble; 0.327	XM_007155841
PvAP2-ERF62	Phvul.003G241900	Chr03	46544077-46547812	3736	1554	517	6,24	57051,6	61,82	unstable	soluble; 0.702	XM_007155843
PvAP2-ERF63	Phvul.003G288500	Chr03	51447092-51451369	4278	1566	521	5,71	58204,7	48,9	unstable	soluble; 0.649	XM_007156405
PvAP2-ERF64	Phvul.003G292400	Chr03	51831261-51832171	911	684	227	5,64	25001	40,59	unstable	insoluble; 0.478	XM_007156453
PvAP2-ERF65	Phvul.004G031900	Chr04	3566458-3567993	1536	1215	404	4,71	45460,3	48,01	unstable	soluble; 0.684	XM_007151203
PvAP2-ERF66	Phvul.004G068900	Chr04	10270274-10271682	1409	1032	343	5,6	37981,9	49,12	unstable	soluble; 0.641	XM_007151643
PvAP2-ERF67	Phvul.004G069900	Chr04	10524334-10526517	2184	747	248	8,63	26614,2	46,23	unstable	soluble; 0.807	XM_007151656
PvAP2-ERF68	Phvul.004G081200	Chr04	14013830-14015119	1290	954	317	5,66	36018,1	41,62	unstable	insoluble; 0.432	XM_007151794
PvAP2-ERF69	Phvul.004G092100	Chr04	24438791-24441164	2374	1308	435	6,37	46732,2	61,74	unstable	insoluble; 0.584	XM_007151923
PvAP2-ERF70	Phvul.004G122000	Chr04	39326716-39327951	1236	675	224	6,4	25625,7	68,63	unstable	insoluble; 0.407	XM_007152281
PvAP2-ERF71	Phvul.004G157600	Chr04	43964844-43965791	948	948	315	4,77	35578,4	52,8	unstable	soluble; 0.712	XM_007152702
PvAP2-ERF72	Phvul.004G169800	Chr04	45126736-45127899	1164	663	220	5,1	23836,3	51,69	unstable	soluble; 0.755	XM_007152840
PvAP2-ERF73	Phvul.005G074700	Chr05	13295710-13296859	1150	582	193	10,06	20929,5	59,57	unstable	soluble; 0.630	XM_007149427
PvAP2-ERF74	Phvul.005G105200	Chr05	31203406-31204629	1224	1224	407	5,93	45836,3	61,23	unstable	soluble; 0.650	XM_007149804
PvAP2-ERF75	Phvul.005G111200	Chr05	32566424-32568115	1692	615	204	6,45	21872,2	56,66	unstable	soluble; 0.781	XM_007149869
PvAP2-ERF76	Phvul.005G126300	Chr05	35040940-35041680	741	741	246	5,86	27360,5	54,95	unstable	soluble; 0.705	XM_007150034
PvAP2-ERF77	Phvul.005G126600	Chr05	35099239-35100039	801	684	227	9,51	25694,2	44,6	unstable	insoluble; 0.563	XM_007150037
PvAP2-ERF78	Phvul.005G138300	Chr05	36714215-36718541	4327	1383	475	7,69	52476,3	45,71	unstable	soluble; 0.780	XM_007150186
PvAP2-ERF79	Phvul.005G170600	Chr05	39420680-39421777	1098	633	210	5,67	23501,5	52,44	unstable	insoluble; 0.573	XM_007150595
PvAP2-ERF80	Phvul.005G180600	Chr05	40253016-40253942	927	627	208	5,61	23756,4	56,33	unstable	soluble; 0.622	XM_007150725
PvAP2-ERF81	Phvul.005G183700	Chr05	40443575-40446525	2951	966	321	9,36	36157,4	60,51	unstable	soluble; 0.671	XM_007150762
PvAP2-ERF82	Phvul.006G047800	Chr06	16171033-16172202	1170	624	207	5,95	22673,1	62,41	unstable	insoluble; 0.569	XM_007146458
PvAP2-ERF83	Phvul.006G080000	Chr06	19885920-19886712	793	753	250	8,94	28018,5	53,57	unstable	insoluble; 0.589	XM_007146839
PvAP2-ERF84	Phvul.006G106100	Chr06	22259920-22260531	612	612	203	5,13	22844,5	57,93	unstable	soluble; 0.670	XM_007147161
PvAP2-ERF85	Phvul.006G110200	Chr06	22619263-22620117	855	855	284	4,81	32699,4	53,31	unstable	soluble; 0.750	XM_007147212

PvAP2-ERF86	Phvul.006G114100	Chr06	22942518-22943272	755	552	183	4,92	20309,7	62,07	unstable	insoluble; 0.491	XM_007147260
PvAP2-ERF87	Phvul.006G163100	Chr06	27418948-27419898	951	951	316	6,05	34649,1	51,3	unstable	soluble; 0.612	XM_007147823
PvAP2-ERF88	Phvul.006G173500	Chr06	28425098-28426505	1408	1062	353	5,43	38026,2	39,86	stable	soluble; 0.700	XM_007147954
PvAP2-ERF89	Phvul.006G179700	Chr06	28949911-28950707	797	792	263	5,37	29608	56,25	unstable	soluble; 0.683	XM_007148025
PvAP2-ERF90	Phvul.006G179800	Chr06	28956271-28957252	982	540	179	7,88	19891,8	61,56	unstable	insoluble; 0.319	XM_007148026
PvAP2-ERF91	Phvul.006G183100	Chr06	29213340-29215073	1734	660	219	8	23556,9	46,63	unstable	soluble; 0.668	XM_007148072
PvAP2-ERF92	Phvul.006G183200	Chr06	29232219-29232722	504	504	167	9,72	18196,7	75,11	unstable	soluble; 0.691	XM_007148073
PvAP2-ERF93	Phvul.007G002900	Chr07	201437-202399	963	963	320	5,92	36796,4	37,77	stable	soluble; 0.675	XM_007142556
PvAP2-ERF94	Phvul.007G027000	Chr07	1987568-1990173	2606	1062	353	4,56	39102	60,43	unstable	insoluble; 0.449	XM_007142845
PvAP2-ERF95	Phvul.007G066500	Chr07	5928454-5929059	606	606	201	6	22418,1	55,45	unstable	insoluble; 0.552	XM_007143304
PvAP2-ERF96	Phvul.007G082000	Chr07	8065792-8067856	2065	807	268	6,98	28556,4	79,6	unstable	soluble; 0.663	XM_007143499
PvAP2-ERF97	Phvul.007G086600	Chr07	8592476-8593166	691	447	148	6,1	16520,7	78,07	unstable	insoluble; 0.489	XM_007143553
PvAP2-ERF98	Phvul.007G102800	Chr07	11606561-11608030	1470	1086	361	8,95	39472,3	38,38	stable	soluble; 0.640	XM_007143739
PvAP2-ERF99	Phvul.007G121200	Chr07	21423708-21424172	465	465	154	9,2	17838,4	55,71	unstable	soluble; 0.731	XM_007143943
PvAP2-ERF100	Phvul.007G127800	Chr07	27274067-27275018	952	630	209	5	23155,6	65,61	unstable	insoluble; 0.543	XM_007144028
PvAP2-ERF101	Phvul.007G128000	Chr07	27559597-27560279	683	429	142	9,07	15911,7	70,21	unstable	soluble; 0.794	XM_007144030
PvAP2-ERF102	Phvul.007G128100	Chr07	27720034-27720477	444	444	147	5,95	16432,9	47,32	unstable	soluble; 0.756	XM_007144031
PvAP2-ERF103	Phvul.007G135300	Chr07	33385083-33386444	1362	1209	402	8,56	44331,9	40,45	unstable	soluble; 0.616	XM_007144120
PvAP2-ERF104	Phvul.007G135900	Chr07	33583547-33584310	764	612	203	8,13	23058,9	61,59	unstable	insoluble; 0.496	XM_007144129
PvAP2-ERF105	Phvul.007G193300	Chr07	43116184-43117296	1113	768	255	5,46	28549,5	58,66	unstable	soluble; 0.685	XM_007144842
PvAP2-ERF106	Phvul.007G193400	Chr07	43136157-43136600	444	444	147	5,62	16597,4	62,85	unstable	soluble; 0.828	XM_007144843
PvAP2-ERF107	Phvul.007G193700	Chr07	43194246-43194881	636	636	211	6,08	23812,6	58,32	unstable	soluble; 0.652	XM_007144848
PvAP2-ERF108	Phvul.007G193800	Chr07	43227132-43227770	639	639	212	5,98	24026	57,25	unstable	soluble; 0.659	XM_007144849
PvAP2-ERF109	Phvul.007G193900	Chr07	43237645-43238058	414	414	137	5,73	15563,3	56,2	unstable	soluble; 0.778	XM_007144850
PvAP2-ERF110	Phvul.007G217800	Chr07	45677796-45679996	2201	900	299	5,63	33667,5	41,19	unstable	soluble; 0.643	XM_007145125
PvAP2-ERF111	Phvul.007G222500	Chr07	46214380-46215168	789	576	191	9,4	21805,8	43,4	unstable	insoluble; 0.548	XM_007145179
PvAP2-ERF112	Phvul.007G222600	Chr07	46221519-46222906	1388	633	210	5,48	22518	54,37	unstable	soluble; 0.642	XM_007145180
PvAP2-ERF113	Phvul.007G240200	Chr07	48016768-48020784	4017	1233	410	9,16	45025,4	56,46	unstable	soluble; 0.676	XM_007145387
PvAP2-ERF114	Phvul.007G241600	Chr07	48138008-48139162	1155	807	268	8,74	29731,9	59,78	unstable	insoluble; 0.522	XM_007145406
PvAP2-ERF115	Phvul.007G241800	Chr07	48154568-48155203	636	636	211	5,9	23762,6	54,74	unstable	soluble; 0.641	XM_007145408
PvAP2-ERF116	Phvul.007G255100	Chr07	49321858-49323149	1292	879	292	5,58	33036,9	57,37	unstable	insoluble; 0.504	XM_007145567
PvAP2-ERF117	Phvul.007G272800	Chr07	51105207-51105858	652	450	149	6,11	16623,1	65,78	unstable	soluble; 0.801	XM_007145780
PvAP2-ERF118	Phvul.007G272900	Chr07	51111180-51111614	435	435	144	9,38	16758,8	53,4	unstable	soluble; 0.835	XM_007145781
PvAP2-ERF119	Phvul.007G273000	Chr07	51127595-51128470	876	639	212	6,97	23321,3	72,33	unstable	soluble; 0.745	XM_007145782
PvAP2-ERF120	Phvul.008G019600	Chr08	1707540-1712293	4754	726	241	7,33	27149,7	62,89	unstable	soluble; 0.601	XM_007139261
PvAP2-ERF121	Phvul.008G039300	Chr08	3311273-3312478	1206	1206	401	4,61	45535,3	59,62	unstable	soluble; 0.689	XM_007139486
PvAP2-ERF122	Phvul.008G043500	Chr08	3742495-3745281	2787	1182	393	7,11	43915,7	51,65	unstable	insoluble; 0.506	XM_007139538
PvAP2-ERF123	Phvul.008G046400	Chr08	4004959-4005582	624	624	207	9,24	23170,9	58,67	unstable	insoluble; 0.564	XM_007139574
PvAP2-ERF124	Phvul.008G046500	Chr08	4010611-4011156	546	546	181	7,88	20177,5	55,2	unstable	insoluble; 0.584	XM_007139575
PvAP2-ERF125	Phvul.008G052000	Chr08	4601738-4606123	4386	2070	689	6,34	75694,4	49,63	unstable	insoluble; 0.415	XM_007139639
PvAP2-ERF126	Phvul.008G092800	Chr08	9500856-9502893	2038	1578	525	5,69	58645,3	48,79	unstable	insoluble; 0.494	XM_007140140
PvAP2-ERF127	Phvul.008G098900	Chr08	10466381-10466854	474	474	157	9,14	17505,3	52,31	unstable	soluble; 0.730	XM_007140217
PvAP2-ERF128	Phvul.008G131500	Chr08	20402865-20410309	7445	690	230	9	25992,3	63,76	unstable	soluble; 0.638	XM_007140602
PvAP2-ERF129	Phvul.008G141000	Chr08	23916631-23917738	1108	930	309	6,06	33899,2	48,1	unstable	soluble; 0.682	XM_007140714

PvAP2-ERF130	Phvul.008G159400	Chr08	40680584-40682430	1847	963	320	5,37	36312,7	56,11	unstable	soluble; 0.677	XM_007140945
PvAP2-ERF131	Phvul.008G165000	Chr08	42621369-42622218	850	717	238	4,94	25857,9	72,72	unstable	soluble; 0.619	XM_007141009
PvAP2-ERF132	Phvul.008G172200	Chr08	45116054-45117855	1802	984	327	5,53	36013,3	57,23	unstable	soluble; 0.656	XM_007141099
PvAP2-ERF133	Phvul.008G185400	Chr08	48884848-48891709	6862	1077	358	6,1	40305,4	46,68	unstable	soluble; 0.634	XM_007141099
PvAP2-ERF134	Phvul.008G214700	Chr08	52704530-52705744	1215	615	204	7,77	22424,1	56,13	unstable	soluble; 0.764	XM_007141600
PvAP2-ERF135	Phvul.008G220400	Chr08	53286141-53288298	2158	1143	380	4,92	42145	39,49	stable	insoluble; 0.381	XM_007141667
PvAP2-ERF136	Phvul.008G222400	Chr08	53485082-53486071	990	990	329	8,39	36041	44,13	unstable	soluble; 0.627	XM_007141687
PvAP2-ERF137	Phvul.008G246000	Chr08	56074147-56075959	1813	753	280	5,76	30843,2	52,78	unstable	insoluble; 0.589	XM_007141963
PvAP2-ERF138	Phvul.008G253600	Chr08	56759415-56762319	2905	909	302	5,83	34412,7	54,79	unstable	soluble; 0.662	XM_007142050
PvAP2-ERF139	Phvul.008G271100	Chr08	58092188-58093372	1185	660	219	8,88	23636,3	52,88	unstable	insoluble; 0.529	XM_007142267
PvAP2-ERF140	Phvul.009G013200	Chr09	2059353-2060102	750	750	249	4,71	27586,3	38,98	stable	soluble; 0.732	XM_007135981
PvAP2-ERF141	Phvul.009G029600	Chr09	6536069-6538397	2329	1062	353	6,51	38677	58,95	unstable	soluble; 0.625	XM_007136171
PvAP2-ERF142	Phvul.009G074300	Chr09	12286871-12290865	3995	1974	657	6,9	72516,7	52	unstable	soluble; 0.609	XM_007136726
PvAP2-ERF143	Phvul.009G084400	Chr09	13327339-13328429	1091	804	267	4,92	29419,5	54,99	unstable	soluble; 0.631	XM_007136851
PvAP2-ERF144	Phvul.009G089300	Chr09	13898357-13899669	1313	576	191	9,48	22239,3	59,03	unstable	insoluble; 0.365	XM_007136909
PvAP2-ERF145	Phvul.009G093600	Chr09	14411949-14413203	1255	594	197	6,23	21850,3	46,49	unstable	soluble; 0.715	XM_007136967
PvAP2-ERF146	Phvul.009G109600	Chr09	16431520-16432907	1388	585	194	5,46	21559,4	40,11	unstable	insoluble; 0.580	XM_007137158
PvAP2-ERF147	Phvul.009G123300	Chr09	18302891-18303953	1063	708	235	5,38	25428,4	63,64	unstable	insoluble; 0.467	XM_007137327
PvAP2-ERF148	Phvul.009G137000	Chr09	20103112-20105454	2343	1026	341	4,78	38280,6	55,23	unstable	insoluble; 0.560	XM_007137501
PvAP2-ERF149	Phvul.009G137900	Chr09	20264478-20266250	1773	891	296	9,41	32439,7	57,15	unstable	soluble; 0.655	XM_007137511
PvAP2-ERF150	Phvul.009G161000	Chr09	23312039-23314113	2075	777	258	6,14	28835,2	63,87	unstable	insoluble; 0.582	XM_007137787
PvAP2-ERF151	Phvul.009G196900	Chr09	29159605-29160767	1163	636	211	8,5	23974,9	63,99	unstable	soluble; 0.601	XM_007138238
PvAP2-ERF152	Phvul.009G206500	Chr09	30520376-30524486	4111	1041	346	6,61	39530,4	55,13	unstable	insoluble; 0.531	XM_007138348
PvAP2-ERF153	Phvul.009G225000	Chr09	33327983-33329052	1070	627	208	4,57	23074,4	59,22	unstable	insoluble; 0.347	XM_007138569
PvAP2-ERF154	Phvul.009G240800	Chr09	35418233-35418610	378	378	125	5,16	14314,9	53,35	unstable	soluble; 0.770	XM_007138767
PvAP2-ERF155	Phvul.009G240900	Chr09	35430515-35430877	363	363	120	5,56	13331,8	64,17	unstable	soluble; 0.793	XM_007138768
PvAP2-ERF156	Phvul.009G251600	Chr09	36449776-36451645	1870	726	241	8,64	26209,6	44,06	unstable	insoluble; 0.474	XM_007138885
PvAP2-ERF157	Phvul.009G262200	Chr09	37365318-37367770	2453	1068	355	5,13	39462,6	40,25	unstable	insoluble; 0.476	XM_007139006
PvAP2-ERF158	Phvul.010G035200	Chr10	5211022-5213130	2109	855	284	5,21	33293,3	55,24	unstable	soluble; 0.713	XM_007134232
PvAP2-ERF159	Phvul.010G050500	Chr10	8020695-8021348	654	654	217	5,97	23746,4	46,44	unstable	insoluble; 0.504	XM_007134415
PvAP2-ERF160	Phvul.010G050600	Chr10	8042080-8042795	716	579	192	9,3	21430,2	58,05	unstable	soluble; 0.635	XM_007134416
PvAP2-ERF161	Phvul.010G050700	Chr10	8078193-8078828	636	636	211	9,39	23994,2	58,04	unstable	insoluble; 0.596	XM_007134417
PvAP2-ERF162	Phvul.010G050800	Chr10	8082893-8083593	701	495	164	8,13	17655,4	40,84	unstable	insoluble; 0.493	XM_007134418
PvAP2-ERF163	Phvul.010G054000	Chr10	8503366-8504597	1232	852	283	5,07	31175,8	43,03	unstable	soluble; 0.734	XM_007134452
PvAP2-ERF164	Phvul.010G092300	Chr10	34005917-34007381	1465	969	322	8,12	34668,3	63,71	unstable	insoluble; 0.420	XM_007134925
PvAP2-ERF165	Phvul.010G114900	Chr10	38090570-38091511	942	552	183	9,27	20476,2	58,57	unstable	insoluble; 0.477	XM_007135205
PvAP2-ERF166	Phvul.010G124700	Chr10	39467544-39470639	3096	1155	384	4,87	42688,3	42	unstable	soluble; 0.621	XM_007135318
PvAP2-ERF167	Phvul.010G130200	Chr10	40027019-40030846	3828	1743	580	6,32	63706	51,61	unstable	soluble; 0.626	XM_007135383
PvAP2-ERF168	Phvul.010G146600	Chr10	41789538-41790170	633	633	210	5,3	23025,7	59,28	unstable	insoluble; 0.436	XM_007135585
PvAP2-ERF169	Phvul.010G154900	Chr10	42398336-42399999	1664	594	197	6,45	22274,9	65,96	unstable	soluble; 0.679	XM_007135682
PvAP2-ERF170	Phvul.010G158700	Chr10	42752077-42752935	859	630	209	10,04	22802,8	31,94	stable	insoluble; 0.390	XM_007135729
PvAP2-ERF171	Phvul.010G159500	Chr10	42802726-42804000	1275	588	195	6,71	22013,5	60,18	unstable	insoluble; 0.572	XM_007135737
PvAP2-ERF172	Phvul.011G058000	Chr11	5016893-5020109	3217	1635	544	6,03	60221,1	46,56	unstable	soluble; 0.624	XM_007131935
PvAP2-ERF173	Phvul.011G071100	Chr11	6338128-6342324	4197	1413	485	6,24	53472,1	47,48	unstable	soluble; 0.714	XM_007132099

PvAP2-ERF174	Phvul.011G091400	Chr11	9148323-9148991	669	669	222	5,27	24412,1	46,3	unstable	soluble; 0.673	XM_007132336
PvAP2-ERF175	Phvul.011G107800	Chr11	12892620-12894683	2064	600	199	8,73	21443,9	44,19	unstable	soluble; 0.712	XM_007132528
PvAP2-ERF176	Phvul.011G118600	Chr11	19321983-19324035	2053	1251	416	5,52	46738,7	73,62	unstable	soluble; 0.708	XM_007132652
PvAP2-ERF177	Phvul.011G125200	Chr11	26734318-26735830	1513	615	204	9,57	22862,7	62,32	unstable	insoluble; 0.569	XM_007132732
PvAP2-ERF178	Phvul.011G162900	Chr11	42720583-42721619	1037	576	191	9,39	20836,2	63,05	unstable	insoluble; 0.541	XM_007133172
PvAP2-ERF179	Phvul.011G187400	Chr11	46403162-46408304	5143	1197	398	6,09	44689,1	67,19	unstable	insoluble; 0.595	XM_007133475
PvAP2-ERF180	Phvul.L006500		scaffold_91:9073-10046	974	576	191	6,96	21264,9	49,52	unstable	insoluble; 0.460	XM_007163845

PvERF1	insoluble; 0.574
PvERF2	insoluble; 0.503
PvERF3	soluble; 0.608
PvERF4	soluble; 0.649
PvERF5	insoluble; 0.484
PvERF6	insoluble; 0.571
PvERF7	insoluble; 0.329
PvERF8	insoluble; 0.501
PvERF9	insoluble; 0.460
PvERF10	insoluble; 0.546
PvERF11	soluble; 0.659
PvERF12	insoluble; 0.454
PvERF13	insoluble; 0.419
PvERF14	insoluble; 0.434
PvERF15	soluble; 0.652
PvERF16	insoluble; 0.516
PvERF17	insoluble; 0.452
PvERF18	soluble; 0.823
PvERF19	soluble; 0.706
PvERF20	insoluble; 0.550
PvERF21	soluble; 0.708
PvERF22	insoluble; 0.538
PvERF23	insoluble; 0.580
PvERF24	soluble; 0.631
PvERF25	soluble; 0.707
PvERF26	soluble; 0.747
PvERF27	insoluble; 0.487
PvERF28	soluble; 0.694
PvERF29	insoluble; 0.570
PvERF30	insoluble; 0.549
PvERF31	soluble; 0.667
PvERF32	insoluble; 0.580
PvERF33	insoluble; 0.578
PvERF34	insoluble; 0.525
PvERF35	insoluble; 0.549
PvERF36	soluble; 0.639

PvERF37	soluble; 0.600
PvERF38	soluble; 0.676
PvERF39	insoluble; 0.327
PvERF40	insoluble; 0.496
PvERF41	soluble; 0.772
PvERF42	soluble; 0.774
PvERF43	soluble; 0.642
PvERF44	soluble; 0.856
PvERF45	soluble; 0.777
PvERF46	soluble; 0.632
PvERF47	insoluble; 0.578
PvERF48	soluble; 0.604
PvERF49	insoluble; 0.382
PvERF50	insoluble; 0.454
PvERF51	soluble; 0.654
PvERF52	soluble; 0.639
PvERF53	insoluble; 0.598
PvERF54	soluble; 0.683
PvERF55	insoluble; 0.445
PvERF56	soluble; 0.687
PvERF57	insoluble; 0.405
PvERF58	soluble; 0.787
PvERF59	insoluble; 0.532
PvERF60	insoluble; 0.575
PvERF61	insoluble; 0.327
PvERF62	soluble; 0.702
PvERF63	soluble; 0.649
PvERF64	insoluble; 0.478
PvERF65	soluble; 0.684
PvERF66	soluble; 0.641
PvERF67	soluble; 0.807
PvERF68	insoluble; 0.432
PvERF69	insoluble; 0.584
PvERF70	insoluble; 0.407
PvERF71	soluble; 0.712
PvERF72	soluble; 0.755

PvERF73	soluble; 0.630
PvERF74	soluble; 0.650
PvERF75	soluble; 0.781
PvERF76	soluble; 0.705
PvERF77	insoluble; 0.563
PvERF78	soluble; 0.780
PvERF79	insoluble; 0.573
PvERF80	soluble; 0.622
PvERF81	soluble; 0.671
PvERF82	insoluble; 0.569
PvERF83	insoluble; 0.589
PvERF84	soluble; 0.670
PvERF85	soluble; 0.750
PvERF86	insoluble; 0.491
PvERF87	soluble; 0.612
PvERF88	soluble; 0.700
PvERF89	soluble; 0.683
PvERF90	insoluble; 0.319
PvERF91	soluble; 0.668
PvERF92	soluble; 0.691
PvERF93	soluble; 0.675
PvERF94	insoluble; 0.449
PvERF95	insoluble; 0.552
PvERF96	soluble; 0.663
PvERF97	insoluble; 0.489
PvERF98	soluble; 0.640
PvERF99	soluble; 0.731
PvERF100	insoluble; 0.543
PvERF101	soluble; 0.794
PvERF102	soluble; 0.756
PvERF103	soluble; 0.616
PvERF104	insoluble; 0.496
PvERF105	soluble; 0.685
PvERF106	soluble; 0.828
PvERF107	soluble; 0.652
PvERF108	soluble; 0.659

PvERF109	soluble; 0.778
PvERF110	soluble; 0.643
PvERF111	insoluble; 0.548
PvERF112	soluble; 0.642
PvERF113	soluble; 0.676
PvERF114	insoluble; 0.522
PvERF115	soluble; 0.641
PvERF116	insoluble; 0.504
PvERF117	soluble; 0.801
PvERF118	soluble; 0.835
PvERF119	soluble; 0.745
PvERF120	soluble; 0.601
PvERF121	soluble; 0.689
PvERF122	insoluble; 0.506
PvERF123	insoluble; 0.564
PvERF124	insoluble; 0.584
PvERF125	insoluble; 0.415
PvERF126	insoluble; 0.494
PvERF127	soluble; 0.730
PvERF128	soluble; 0.638
PvERF129	soluble; 0.682
PvERF130	soluble; 0.677
PvERF131	soluble; 0.619
PvERF132	soluble; 0.656
PvERF133	soluble; 0.634
PvERF134	soluble; 0.764
PvERF135	insoluble; 0.381
PvERF136	soluble; 0.627
PvERF137	insoluble; 0.589
PvERF138	soluble; 0.662
PvERF139	insoluble; 0.529
PvERF140	soluble; 0.732
PvERF141	soluble; 0.625
PvERF142	soluble; 0.609
PvERF143	soluble; 0.631
PvERF144	insoluble; 0.365

PvERF145	soluble; 0.715
PvERF146	insoluble; 0.580
PvERF147	insoluble; 0.467
PvERF148	insoluble; 0.560
PvERF149	soluble; 0.655
PvERF150	insoluble; 0.582
PvERF151	soluble; 0.601
PvERF152	insoluble; 0.531
PvERF153	insoluble; 0.347
PvERF154	soluble; 0.770
PvERF155	soluble; 0.793
PvERF156	insoluble; 0.474
PvERF157	insoluble; 0.476
PvERF158	soluble; 0.713
PvERF159	insoluble; 0.504
PvERF160	soluble; 0.635
PvERF161	insoluble; 0.596
PvERF162	insoluble; 0.493
PvERF163	soluble; 0.734
PvERF164	insoluble; 0.420
PvERF165	insoluble; 0.477
PvERF166	soluble; 0.621
PvERF167	soluble; 0.626
PvERF168	insoluble; 0.436
PvERF169	soluble; 0.679
PvERF170	insoluble; 0.390
PvERF171	insoluble; 0.572
PvERF172	soluble; 0.624
PvERF173	soluble; 0.714
PvERF174	soluble; 0.673
PvERF175	soluble; 0.712
PvERF176	soluble; 0.708
PvERF177	insoluble; 0.569
PvERF178	insoluble; 0.541
PvERF179	insoluble; 0.595
PvERF180	insoluble; 0.460

Query	Phytozome Identfier
Phvul.001G010400	Phvul.001G010400
Phvul.001G023700	Phvul.001G023700
Phvul.001G031200	Phvul.001G031200
Phvul.001G044500	Phvul.001G044500
Phvul.001G046900	Phvul.001G046900
Phvul.001G073800	Phvul.001G073800
Phvul.001G084000	Phvul.001G084000
Phvul.001G099700	Phvul.001G099700
Phvul.001G111800	Phvul.001G111800
Phvul.001G131300	Phvul.001G131300
Phvul.001G136100	Phvul.001G136100
Phvul.001G137500	Phvul.001G137500
Phvul.001G137600	Phvul.001G137600
Phvul.001G157600	Phvul.001G157600
Phvul.001G160100	Phvul.001G160100
Phvul.001G160200	Phvul.001G160200
Phvul.001G160300	Phvul.001G160300
Phvul.001G160400	Phvul.001G160400
Phvul.001G160500	Phvul.001G160500
Phvul.001G174400	Phvul.001G174400
Phvul.001G187100	Phvul.001G187100
Phvul.001G251200	Phvul.001G251200
Phvul.002G009100	Phvul.002G009100
Phvul.002G016700	Phvul.002G016700
Phvul.002G016900	Phvul.002G016900
Phvul.002G035100	Phvul.002G035100
Phvul.002G035900	Phvul.002G035900
Phvul.002G036000	Phvul.002G036000
Phvul.002G055700	Phvul.002G055700
Phvul.002G055800	Phvul.002G055800
Phvul.002G056800	Phvul.002G056800
Phvul.002G146400	Phvul.002G146400
Phvul.002G149500	Phvul.002G149500
Phvul.002G153900	Phvul.002G153900
Phvul.002G154000	Phvul.002G154000
Phvul.002G162500	Phvul.002G162500
Phvul.002G163700	Phvul.002G163700
Phvul.002G168900	Phvul.002G168900
Phvul.002G237300	Phvul.002G237300
Phvul.002G254500	Phvul.002G254500
Phvul.002G267800	Phvul.002G267800
Phvul.002G281300	Phvul.002G281300
Phvul.002G295700	Phvul.002G295700
Phvul.002G310200	Phvul.002G310200
Phvul.002G315900	Phvul.002G315900
Phvul.003G035500	Phvul.003G035500
Phvul.003G064800	Phvul.003G064800
Phvul.003G069000	Phvul.003G069000
Phvul.003G102500	Phvul.003G102500
Phvul.003G107900	Phvul.003G107900

Phvul.003G111800	Phvul.003G111800
Phvul.003G144500	Phvul.003G144500
Phvul.003G153100	Phvul.003G153100
Phvul.003G165000	Phvul.003G165000
Phvul.003G180000	Phvul.003G180000
Phvul.003G212700	Phvul.003G212700
Phvul.003G212800	Phvul.003G212800
Phvul.003G222600	Phvul.003G222600
Phvul.003G223600	Phvul.003G223600
Phvul.003G232600	Phvul.003G232600
Phvul.003G241700	Phvul.003G241700
Phvul.003G241900	Phvul.003G241900
Phvul.003G288500	Phvul.003G288500
Phvul.003G292400	Phvul.003G292400
Phvul.004G031900	Phvul.004G031900
Phvul.004G068900	Phvul.004G068900
Phvul.004G069900	Phvul.004G069900
Phvul.004G081200	Phvul.004G081200
Phvul.004G092100	Phvul.004G092100
Phvul.004G122000	Phvul.004G122000
Phvul.004G157600	Phvul.004G157600
Phvul.004G169800	Phvul.004G169800
Phvul.005G074700	Phvul.005G074700
Phvul.005G105200	Phvul.005G105200
Phvul.005G111200	Phvul.005G111200
Phvul.005G126300	Phvul.005G126300
Phvul.005G126600	Phvul.005G126600
Phvul.005G138300	Phvul.005G138300
Phvul.005G170600	Phvul.005G138300
Phvul.005G180600	Phvul.005G170600
Phvul.005G183700	Phvul.005G180600
Phvul.006G047800	Phvul.005G183700
Phvul.006G080000	Phvul.006G047800
Phvul.006G106100	Phvul.006G080000
Phvul.006G110200	Phvul.006G106100
Phvul.006G114100	Phvul.006G110200
Phvul.006G163100	Phvul.006G114100
Phvul.006G173500	Phvul.006G163100
Phvul.006G179700	Phvul.006G173500
Phvul.006G179800	Phvul.006G179700
Phvul.006G183100	Phvul.006G179800
Phvul.006G183200	Phvul.006G183100
Phvul.007G002900	Phvul.006G183200
Phvul.007G027000	Phvul.007G002900
Phvul.007G066500	Phvul.007G027000
Phvul.007G082000	Phvul.007G066500
Phvul.007G086600	Phvul.007G082000
Phvul.007G102800	Phvul.007G086600
Phvul.007G119100	Phvul.007G102800
Phvul.007G121200	Phvul.007G121200
Phvul.007G127800	Phvul.007G127800

Phvul.007G128000	Phvul.007G128000
Phvul.007G128100	Phvul.007G128100
Phvul.007G135300	Phvul.007G135300
Phvul.007G135900	Phvul.007G135900
Phvul.007G193300	Phvul.007G193300
Phvul.007G193400	Phvul.007G193400
Phvul.007G193700	Phvul.007G193700
Phvul.007G193800	Phvul.007G193800
Phvul.007G193900	Phvul.007G193900
Phvul.007G217800	Phvul.007G217800
Phvul.007G222500	Phvul.007G222500
Phvul.007G222600	Phvul.007G222600
Phvul.007G240200	Phvul.007G240200
Phvul.007G241600	Phvul.007G241600
Phvul.007G241800	Phvul.007G241800
Phvul.007G255100	Phvul.007G255100
Phvul.007G272800	Phvul.007G272800
Phvul.007G272900	Phvul.007G272900
Phvul.007G273000	Phvul.007G273000
Phvul.008G019600	Phvul.008G019600
Phvul.008G039300	Phvul.008G039300
Phvul.008G043500	Phvul.008G043500
Phvul.008G046400	Phvul.008G046400
Phvul.008G046500	Phvul.008G046500
Phvul.008G052000	Phvul.008G052000
Phvul.008G092800	Phvul.008G092800
Phvul.008G098900	Phvul.008G098900
Phvul.008G131500	Phvul.008G131500
Phvul.008G141000	Phvul.008G141000
Phvul.008G159400	Phvul.008G159400
Phvul.008G165000	Phvul.008G165000
Phvul.008G172200	Phvul.008G172200
Phvul.008G185400	Phvul.008G185400
Phvul.008G214700	Phvul.008G214700
Phvul.008G220400	Phvul.008G220400
Phvul.008G222400	Phvul.008G246000
Phvul.008G246000	Phvul.008G253600
Phvul.008G253600	Phvul.008G271100
Phvul.008G271100	Phvul.009G013200
Phvul.009G013200	Phvul.009G029600
Phvul.009G029600	Phvul.009G074300
Phvul.009G074300	Phvul.009G084400
Phvul.009G084400	Phvul.009G089300
Phvul.009G089300	Phvul.009G093600
Phvul.009G093600	Phvul.009G109600
Phvul.009G109600	Phvul.009G123300
Phvul.009G123300	Phvul.009G137000
Phvul.009G137000	Phvul.009G137900
Phvul.009G137900	Phvul.009G161000
Phvul.009G161000	Phvul.009G196900
Phvul.009G196900	Phvul.009G206500

Phvul.009G206500	Phvul.009G225000
Phvul.009G225000	Phvul.009G240800
Phvul.009G240800	Phvul.009G240900
Phvul.009G240900	Phvul.009G251600
Phvul.009G251600	Phvul.009G262200
Phvul.009G262200	Phvul.010G035200
Phvul.010G035200	Phvul.010G050500
Phvul.010G050500	Phvul.010G050600
Phvul.010G050600	Phvul.010G050700
Phvul.010G050700	Phvul.010G050800
Phvul.010G050800	Phvul.010G054000
Phvul.010G054000	Phvul.010G092300
Phvul.010G092300	Phvul.010G114900
Phvul.010G114900	Phvul.010G124700
Phvul.010G124700	Phvul.010G130200
Phvul.010G130200	Phvul.010G146600
Phvul.010G146600	Phvul.010G154900
Phvul.010G154900	Phvul.010G158700
Phvul.010G158700	Phvul.010G159500
Phvul.010G159500	Phvul.011G058000
Phvul.011G058000	Phvul.011G071100
Phvul.011G071100	Phvul.011G091400
Phvul.011G091400	Phvul.011G107800
Phvul.011G107800	Phvul.011G118600
Phvul.011G118600	Phvul.011G125200
Phvul.011G125200	Phvul.011G162900
Phvul.011G162900	Phvul.011G187400
Phvul.011G187400	Phvul.L006500
Phvul.L006500	

Table S3: Amino acid composition of PvAP2-ERF family proteins

All frequencies are given in percent.	Ala	Cys	Asp	Glu	Phe	Gly	His	Ile	Lys	Leu	Met	Asn	Pro	Gln	Arg	Ser	Thr	Val	Trp	Tyr	Total
Domain: Data	8,658008658	0	4,761904762	6,060606061	4,761904762	6,493506494	1,731601732	2,164502165	2,597402597	5,194805195	2,597402597	6,926406926	8,225108225	3,896103896	5,627705628	13,41991342	6,926406926	3,896103896	1,731601732	4,329004329	231
	9,722222222	2,314814815	5,555555556	12,03703704	2,777777778	3,703703704	2,314814815	3,703703704	2,314814815	6,018518519	2,777777778	5,092592593	5,092592593	2,314814815	10,64814815	8,333333333	4,62962963	7,407407407	2,314814815	0,925925926	216
PvERF8	9,265175719	1,277955272	7,987220447	7,028753994	4,792332268	3,833865815	2,875399361	3,194888179	5,111821086	7,987220447	0,638977636	3,514376997	7,348242812	2,555910543	6,709265176	9,584664537	7,348242812	5,111821086	1,916932907	1,916932907	313
PvERF34	13,65638767	1,762114537	4,845814978	7,929515419	5,286343612	4,405286344	0,440528634	2,643171806	4,845814978	4,845814978	3,964757709	5,286343612	5,726872247	3,964757709	7,04845815	9,251101322	6,167400881	5,286343612	1,762114537	0,881057269	227
PvERF36	3,521126761	1,056338028	7,746478873	10,56338028	4,225352113	3,873239437	2,464788732	5,633802817	5,633802817	7,394366197	2,464788732	5,633802817	4,929577465	2,816901408	5,985915493	8,450704225	6,690140845	7,042253521	1,408450704	2,464788732	284
PvERF64	11,17318436	0,558659218	3,351955307	6,703910615	4,469273743	5,586592179	2,234636872	2,793296089	3,910614525	6,145251397	2,234636872	8,938547486	3,351955307	5,586592179	6,703910615	10,61452514	7,82122905	3,351955307	1,117318436	3,351955307	179
PvERF85	6,697108067	1,522070015	3,652968037	6,392694064	2,283105023	8,219178082	3,348554033	3,348554033	4,414003044	6,544901065	4,109589041	6,240487062	4,414003044	5,631659056	5,327245053	11,87214612	5,783866058	5,327245053	1,369863014	3,500761035	657
PvERF90	11,98156682	0	6,451612903	6,451612903	4,147465438	6,451612903	1,382488479	3,225806452	5,529953917	8,294930876	2,304147465	7,373271889	4,147465438	0,921658986	6,451612903	11,52073733	4,147465438	5,069124424	2,304147465	1,843317972	217
PvERF142	12,80487805	0	4,87804878	4,268292683	4,268292683	7,926829268	4,268292683	1,829268293	3,048780488	8,536585366	1,219512195	4,87804878	5,487804878	3,658536585	6,707317073	11,58536585	7,317073171	3,658536585	1,219512195	2,43902439	164
PvERF159	6,435643564	0,742574257	3,712871287	7,178217822	3,217821782	6,930693069	2,722772277	3,465346535	6,188118812	7,425742574	1,732673267	5,693069307	4,207920792	3,217821782	7,425742574	12,12871287	6,435643564	4,702970297	1,732673267	4,702970297	404
PvERF162	7,065217391	1,086956522	4,076086957	7,880434783	4,076086957	4,619565217	3,532608696	4,076086957	4,891304348	7,336956522	2,445652174	8,967391304	3,804347826	4,891304348	6,52173913	8,695652174	5,434782609	3,804347826	1,630434783	5,163043478	368
PvERF48.1	10,58020478	2,389078498	5,802047782	7,167235495	3,412969283	3,412969283	1,706484642	4,09556314	4,09556314	6,484641638	2,04778157	7,167235495	5,119453925	2,389078498	6,14334471	12,28668942	4,09556314	7,849829352	2,04778157	1,706484642	293
PvERF53	7,156673114	1,160541586	5,996131528	7,350096712	3,868471954	9,671179884	3,481624758	3,094777563	5,609284333	5,415860735	2,127659574	5,02901354	5,02901354	4,642166344	6,382978723	11,79883946	3,481624758	4,448742747	1,547388781	2,707930368	517
PvERF57	13,98963731	0,518134715	6,735751295	4,14507772	4,14507772	5,699481865	1,03626943	1,03626943	3,10880829	8,808290155	0,518134715	4,663212435	10,88082902	1,03626943	11,39896373	7,772020725	6,21761658	5,699481865	1,03626943	1,554404145	193
PvERF62	9,31372549	1,960784314	3,921568627	9,803921569	1,470588235	9,31372549	1,960784314	2,450980392	8,333333333	4,901960784	3,431372549	5,392156863	5,392156863	0,980392157	4,901960784	15,68627451	1,470588235	5,392156863	1,960784314	1,960784314	204
PvERF73	7,881773399	3,448275862	5,911330049	8,374384236	5,418719212	4,926108374	2,463054187	3,448275862	6,896551724	6,896551724	1,477832512	2,955665025	4,433497537	2,463054187	8,374384236	9,359605911	6,403940887	4,926108374	0,985221675	2,955665025	203
PvERF75	7,482993197	0,680272109	6,12244898	7,482993197	5,442176871	6,12244898	2,040816327	3,401360544	2,721088435	7,482993197	3,401360544	4,761904762	6,802721088	2,721088435	8,843537415	12,92517007	3,401360544	3,401360544	1,360544218	3,401360544	147
PvERF104	6,164383562	1,02739726	4,109589041	9,589041096	3,082191781	5,136986301	2,739726027	4,109589041	5,821917808	8,219178082	2,054794521	5,479452055	6,849315068	5,821917808	5,136986301	9,589041096	5,821917808	4,794520548	2,397260274	2,054794521	292
PvERF106	6,944444444	0,694444444	3,472222222	10,41666667	4,861111111	5,555555556	5,555555556	2,777777778	8,333333333	6,25	2,083333333	2,777777778	4,861111111	3,472222222	9,027777778	9,027777778	4,166666667	4,861111111	1,388888889	3,472222222	144
PvERF116	9,128630705	0,414937759	4,564315353	2,489626556	6,22406639	3,734439834	7,053941909	0,829875519	3,734439834	4,564315353	2,074688797	3,319502075	9,128630705	13,69294606	3,319502075	11,2033195	7,053941909	3,319502075	1,244813278	2,904564315	241
PvERF118	6,676342525	1,015965167	4,934687954	4,499274311	2,757619739	8,708272859	5,515239478	2,757619739	3,047895501	6,531204644	2,467343977	6,676342525	4,644412192	5,660377358	4,644412192	12,62699565	7,837445573	3,483309144	1,306240929	4,208998549	689
PvERF120	8,823529412	1,960784314	8,333333333	3,431372549	4,901960784	4,411764706	3,921568627	1,960784314	3,921568627	5,392156863	0,980392157	1,960784314	13,23529412	2,450980392	8,333333333	10,29411765	5,392156863	7,843137255	1,470588235	0,980392157	204
PvERF125	7,751937984	0	4,651162791	6,201550388	5,03875969	6,976744186	3,488372093	1,162790698	2,713178295	5,426356589	2,713178295	5,03875969	4,651162791	8,914728682	6,589147287	14,34108527	6,589147287	3,100775194	1,550387597	3,100775194	258
PvERF134	8,510638298	1,914893617	5,106382979	5,957446809	5,319148936	7,659574468	2,127659574	2,765957447	5,106382979	4,893617021	2,127659574	6,595744681	5,319148936	4,255319149	5,531914894	11,4893617	5,744680851	5,106382979	1,70212766	2,765957447	470
PvERF150	9,230769231	3,076923077	5,384615385	10,76923077	3,076923077	6,153846154	2,307692308	3,076923077	8,461538462	9,230769231	3,076923077	5,384615385	2,307692308	2,307692308	7,692307692	5,384615385	4,615384615	5,384615385	1,538461538	1,538461538	130
PvERF173.2	7,929515419	0,440528634	6,607929515	9,691629956	6,607929515	5,286343612	0,881057269	1,321585903	6,607929515	7,929515419	1,321585903	5,726872247	4,405286344	3,083700441	5,726872247	11,01321586	5,286343612	5,726872247	1,762114537	2,643171806	227
PvERF15	14,97326203	0,534759358	5,882352941	6,417112299	3,743315508	4,278074866	2,673796791	2,673796791	10,16042781	9,625668449	1,604278075	2,673796791	7,486631016	4,278074866	4,812834225	8,021390374	3,20855615	3,20855615	2,673796791	1,069518717	187
PvERF19	5,993690852	2,523659306	4,731861199	8,832807571	3,470031546	3,470031546	0,94637224	3,470031546	6,624605678	7,570977918	2,523659306	7,255520505	3,470031546	7,255520505	5,678233438	10,41009464	7,255520505	5,047318612	1,261829653	2,208201893	317
PvERF31	8,173076923	0,961538462	3,846153846	11,05769231	2,884615385	4,807692308	4,326923077	3,365384615	4,326923077	8,173076923	3,365384615	2,884615385	4,326923077	6,730769231	6,730769231	10,09615385	5,769230769	3,846153846	2,403846154	1,923076923	208
PvERF68	4,47761194	2,985074627	4,104477612	6,343283582	4,104477612	5,223880597	1,119402985	4,47761194	7,462686567	7,089552239	3,731343284	6,343283582	7,835820896	2,985074627	4,850746269	11,94029851	6,71641791	5,597014925	1,492537313	1,119402985	268
PvERF80	10	2,142857143	8,928571429	4,642857143	4,642857143	3,571428571	2,857142857	2,857142857	6,428571429	6,071428571	1,428571429	4,285714286	8,571428571	2,5	5	11,07142857	7,142857143	4,285714286	1,428571429	2,142857143	280
PvERF114	8,609271523	0,331125828	5,629139073	9,271523179	3,311258278	7,284768212	2,649006623	4,635761589	5,298013245	4,635761589	1,324503311	6,291390728	3,311258278	3,642384106	7,61589404	8,609271523	5,629139073	4,304635762	1,986754967	5,629139073	302
PvERF137.1	8,835341365	2,81124498	6,024096386	7,228915663	2,409638554	5,62248996	0,803212851	4,417670683	5,62248996	6,425702811	4,819277108	5,220883534	10,44176707	4,016064257	3,212851406	5,62248996	7,228915663	4,016064257	2,81124498	2,409638554	249
PvERF138	7,582938389	1,421800948	2,369668246	9,478672986	2,843601896	5,687203791	3,317535545	3,791469194	4,739336493	6,161137441	4,265402844	5,687203791	5,687203791	5,687203791	8,056872038	8,530805687	7,582938389	3,791469194	2,843601896	0,473933649	211
PvERF140	12,02185792	1,639344262	5,464480874	5,464480874	2,732240437	4,371584699	4,371584699	2,732240437	6,010928962	6,010928962	3,278688525	1,092896175	10,38251366	3,278688525	7,650273224	9,289617486	6,010928962	3,278688525	3,825136612	1,092896175	183
PvERF151	9,803921569	0,490196078	7,843137255	3,921568627	4,411764706	2,450980392	1,470588235	1,960784314	6,862745098	6,862745098	0,980392157	6,862745098	9,803921569	2,941176471	8,333333333	8,333333333	5,392156863	7,843137255	0,980392157	2,450980392	204
PvERF165	7,109004739	0,947867299	4,265402844	6,161137441	4,739336493	6,161137441	0,947867299	0,947867299	5,687203791	10,90047393	2,369668246	6,161137441	4,739336493	2,369668246	7,582938389	10,90047393	7,582938389	5,687203791	1,895734597	2,843601896	211
PvERF177	6,470588235	1,764705882	7,058823529	10	5,882352941	5,882352941	1,176470588	2,941176471	5,882352941	9,411764706	4,117647059	2,352941176	4,705882353	4,705882353	5,294117647	9,411764706	4,705882353	5,294117647	1,176470588	1,764705882	170
PvERF16	7,843137255	1,089324619	5,882352941	4,575163399	5,446623094	7,625272331	2,832244009	5,010893246	3,921568627	4,793028322	1,74291939	5,010893246	6,318082789	3,921568627	5,446623094	13,50762527	5,22875817	4,793028322	1,74291939	3,267973856	459
PvERF17	7,457627119	1,016949153	5,084745763	6,779661017	4,745762712	4,745762712	2,033898305	3,050847458	5,762711864	8,813559322	3,050847458	3,728813559	5,423728814	5,084745763	3,728813559	14,23728814	6,101694915	4,406779661	1,355932203	3,389830508	295
PvERF20	7,86163522	0,943396226	4,402515723	7,86163522	2,830188679	6,289308176	2,51572327	1,572327044	2,51572327	5,34591195	2,201257862	3,144654088	6,918238994	4,402515723	9,433962264	15,72327044	5,974842767	4,402515723	0,943396226	4,716981132	318
PvERF22	11,2195122	0,487804878	6,341463415	5,365853659	2,926829268	4,390243902	0,487804878	4,390243902	1,951219512	7,317073171	2,43902439	4,87804878	12,68292683	2,926829268	6,829268293	11,70731707	5,365853659	3,414634146	1,463414634	3,414634146	205
PvERF41	9,596928983	1,535508637	4,798464491	6,333973129	6,142034549	5,950095969	2,879078695	4,222648752	3,838771593	7,677543186	1,151631478	4,606525912	4,414587332	4,990403071	4,990403071	9,788867562	7,293666027	4,222648752	1,727447217	3,838771593	521
PvERF59	7,582938389	1,421800948	7,109004739	6,63507109	4,739336493	4,265402844	2,843601896	3,791469194	6,63507109	8,056872038	1,895734597	4,265402844	4,739336493	5,213270142	5,687203791	10,90047393	4,265402844	6,161137441	1,421800948	2,369668246	211
PvERF63	7,547169811	1,886792453	5,660377358	7,547169811	5,188679245	4,245283019	2,358490566	2,830188679	6,603773585	8,490566038	2,830188679	4,245283019	4,245283019	5,660377358	5,188679245	10,8490566	4,245283019	6,132075472	1,41509434	2,830188679	212
PvERF107	8,917197452	1,27388535	7,643312102	4,458598726	1,27388535	5,732484076	1,27388535	2,547770701	6,369426752	5,732484076	1,910828025	4,458598726	5,732484076	7,643312102	8,280254777	12,10191083	5,095541401	4,458598726	1,910828025	3,184713376	157
PvERF108	9,166666667	0,833333333	5,833333333	12,5	3,333333333	8,333333333	0,833333333	4,166666667	8,333333333	6,666666667	0,833333333	1,666666667	4,166666667	2,5	8,333333333	10	5	4,166666667	1,666666667	1,666666667	120
PvERF127	8,732394366	1,408450704	9,577464789	5,070422535	5,352112676	5,915492958	1,690140845	2,816901408	6,197183099	6,478873239	2,535211268	5,070422535	3,943661972	5,070422535	5,352112676	11,54929577	3,661971831	5,915492958	1,971830986	1,690140845	355
PvERF155	10	1,904761905	4,285714286	8,571428571	4,761904762	3,333333333	1,904761905	4,761904762	4,761904762	5,238095238	1,904761905	1,904761905	6,666666667	3,333333333	5,238095238	13,80952381	9,523809524	5,238095238	1,904761905	0,952380952	210
PvERF157	7,692307692	1,538461538	2,564102564	8,205128205	2,564102564	6,666666667	4,102564103	3,58974359	3,58974359	8,205128205	2,564102564	4,615384615	5,641025641	7,692307692	6,666666667	10,76923077	4,102564103	4,102564103	3,076923077	2,051282051	195
PvERF168	8,895705521	1,226993865	5,521472393	5,521472393	5,214723926	4,601226994	4,294478528	3,680981595	4,294478528	6,134969325	1,226993865	4,90797546	9,202453988	5,214723926	5,828220859	11,65644172	4,90797546	2,45398773	1,226993865	3,987730061	326
PvERF171	5,504587156	0	6,880733945	8,256880734	5,963302752	6,880733945	0,458715596	3,669724771	4,587155963	5,963302752	4,128440367	5,504587156	2,293577982	3,669724771	7,339449541	11,00917431	7,339449541	7,339449541	0,458715596	2,752293578	218
PvERF7	8,130081301	1,62601626	4,06504065	5,691056911	3,658536585	4,471544715	2,43902439	3,658536585	6,097560976	8,130081301	3,658536585	7,317073171	2,845528455	7,723577236	4,471544715	8,943089431	7,723577236	4,06504065	1,219512195	4,06504065	246
PvERF13	8,490566038	1,257861635	5,660377358	6,918238994	4,402515723	5,660377358	2,830188679	4,402515723	5,34591195	6,918238994	2,830188679	5,031446541	5,660377358	5,974842767	4,088050314	11,00628931	3,144654088	5,34591195	1,886792453	3,144654088	318
PvERF39	9,061488673	2,588996764	7,766990291	9,061488673	4,854368932	6,148867314	1,941747573	3,55987055	4,854368932	6,796116505	0,647249191	3,236245955	3,236245955	2,588996764	7,443365696	10,03236246	5,501618123	7,766990291	1,941747573	0,970873786	309
PvERF40	9,340659341	2,747252747	5,494505495	4,945054945	5,769230769	4,945054945	3,571428571	1,923076923	3,571428571	6,318681319	3,021978022	6,043956044	6,318681319	3,571428571	6,318681319	13,46153846	3,571428571	5,769230769	0,549450549	2,747252747	364
PvERF43	8	1,052631579	4,631578947	6,105263158	5,052631579	7,368421053	2,526315789	3,578947368	5,052631579	6,315789474	1,473684211	5,263157895	5,473684211	4,842105263	5,894736842	11,57894737	5,263157895	6,105263158	1,473684211	2,947368421	475
PvERF47	6,4	1,2	6,4	5,2	6,4	4	2,8	2,8	6,8	4,4	0,8	3,2	13,6	3,2	6,4	6,8	10,8	6	1,6	1,2	250
PvERF78.1	11,94029851	1,492537313	5,970149254	9,452736318	3,980099502	5,970149254	2,487562189	1,492537313	4,47761194	6,965174129	2,985074627	2,985074627	5,472636816	1,492537313	9,452736318	6,965174129	6,467661692	6,467661692	1,492537313	1,990049751	201
PvERF83	11,56716418	0	4,47761194	4,47761194	4,47761194	11,19402985	1,865671642	0,373134328	1,492537313	6,71641791	2,23880597	1,865671642	11,19402985	4,850746269	7,462686567	14,17910448	2,611940299	3,358208955	2,611940299	2,985074627	268
PvERF95	7,1875	1,875	7,5	9,0625	5,3125	4,0625	1,875	3,4375	7,1875	6,25	1,875	4,0625	6,875	3,4375	6,5625	7,5	5,9375	6,875	1,25	1,875	320
PvERF96.2	8,823529412	1,260504202	6,302521008	4,201680672	2,521008403	2,941176471	0,840336134	4,621848739	4,621848739	8,823529412	3,361344538	3,781512605	9,243697479	3,361344538	3,361344538	19,32773109	3,781512605	3,361344538	2,100840336	3,361344538	238
PvERF130	8,868501529	0,917431193	4,892966361	6,116207951	3,669724771	4,892966361	1,22324159	1,529051988	6,116207951	10,09174312	2,44648318	3,669724771	10,09174312	6,727828746	3,363914373	12,8440367	3,669724771	3,669724771	1,529051988	3,669724771	327
PvERF131	10,2484472	1,863354037	4,968944099	4,658385093	3,416149068	8,695652174	1,242236025	3,105590062	4,658385093	4,347826087	2,173913043	4,658385093	6,52173913	3,416149068	5,590062112	13,66459627	5,279503106	5,279503106	1,552795031	4,658385093	322
PvERF132	8,558558559	1,801801802	5,405405405	7,207207207	3,603603604	4,954954955	2,252252252	5,405405405	4,504504505	7,207207207	2,252252252	9,009009009	8,558558559	1,801801802	4,954954955	10,81081081	4,954954955	3,603603604	1,801801802	1,351351351	222
PvERF164	13,61256545	0	6,282722513	5,759162304	5,235602094	4,712041885	2,094240838	1,047120419	3,141361257	6,806282723	0,523560209	2,094240838	9,42408377	1,047120419	10,47120419	10,9947644	5,759162304	7,853403141	1,047120419	2,094240838	191
PvERF174	9,045226131	1,507537688	3,015075377	7,537688442	3,015075377	3,51758794	3,51758794	2,512562814	3,51758794	8,542713568	3,015075377	2,512562814	6,030150754	6,030150754	8,542713568	12,56281407	6,532663317	5,527638191	1,507537688	2,010050251	199
PvERF178	5,862646566	1,675041876	4,355108878	6,197654941	2,680067002	5,695142379	3,51758794	4,020100503	4,355108878	7,035175879	3,685092127	5,527638191	5,192629816	6,532663317	5,862646566	12,73031826	5,527638191	4,522613065	1,340033501	3,685092127	597
PvERF5	6	2	6,285714286	4	4,285714286	5,142857143	2,857142857	2,571428571	2,571428571	6,285714286	2,571428571	6,857142857	7,714285714	5,428571429	5,142857143	13,71428571	7,428571429	4	1,714285714	3,428571429	350
PvERF23.2	11,64021164	1,851851852	7,671957672	5,555555556	3,703703704	5,291005291	1,587301587	4,761904762	7,936507937	6,613756614	0,529100529	3,174603175	6,349206349	3,174603175	11,64021164	1,851851852	3,968253968	6,878306878	3,439153439	2,380952381	378
PvERF49	9,661835749	1,93236715	9,178743961	4,830917874	6,280193237	8,212560386	1,449275362	0,483091787	3,381642512	4,830917874	1,93236715	2,415458937	11,11111111	1,449275362	9,661835749	8,212560386	5,314009662	6,280193237	0,966183575	2,415458937	207
PvERF50	9,852216749	0,492610837	3,448275862	11,33004926	2,463054187	4,433497537	1,97044335	2,463054187	4,926108374	8,866995074	2,955665025	3,448275862	2,463054187	5,418719212	5,911330049	11,33004926	5,418719212	7,389162562	1,97044335	3,448275862	203
PvERF82	6,585365854	1,951219512	4,87804878	5,609756098	4,87804878	9,024390244	1,951219512	4,87804878	6,097560976	6,585365854	2,43902439	5,12195122	4,390243902	4,146341463	6,829268293	13,41463415	4,390243902	3,658536585	0,975609756	2,195121951	410
PvERF84	9,395973154	0	5,369127517	10,73825503	4,026845638	8,724832215	2,684563758	0,67114094	5,369127517	4,697986577	2,684563758	2,684563758	4,697986577	1,342281879	9,395973154	15,43624161	2,013422819	4,026845638	1,342281879	4,697986577	149
PvERF113	6,232294618	0,849858357	4,249291785	5,382436261	4,249291785	7,082152975	2,549575071	3,116147309	5,382436261	8,21529745	2,549575071	4,815864023	8,498583569	5,382436261	3,682719547	14,44759207	5,382436261	3,399433428	1,416430595	3,116147309	353
PvERF117	9,737827715	2,247191011	7,116104869	6,367041199	5,243445693	5,617977528	2,996254682	1,498127341	4,868913858	6,741573034	2,996254682	4,119850187	7,116104869	2,621722846	3,370786517	11,61048689	6,367041199	4,119850187	2,247191011	2,996254682	267
PvERF141	8	0	7,2	12	5,6	8	0,8	4	7,2	7,2	1,6	4	4	0,8	8,8	7,2	4	4	1,6	4	125
PvERF143	9,42408377	2,617801047	5,235602094	7,853403141	2,094240838	6,282722513	2,617801047	3,141361257	7,329842932	7,329842932	2,094240838	4,712041885	6,282722513	4,188481675	5,759162304	8,90052356	4,188481675	5,235602094	3,141361257	1,570680628	191
PvERF154	8,426966292	1,123595506	7,865168539	4,494382022	2,247191011	3,370786517	3,93258427	3,370786517	5,617977528	7,865168539	2,808988764	5,617977528	6,179775281	3,370786517	10,6741573	7,303370787	6,741573034	4,494382022	1,685393258	2,808988764	178
PvERF180	13,49206349	0,793650794	7,142857143	7,936507937	0,793650794	9,523809524	2,380952381	3,174603175	5,555555556	2,380952381	2,380952381	2,380952381	4,761904762	1,587301587	11,9047619	7,936507937	5,555555556	4,761904762	3,174603175	2,380952381	126
PvERF2	10	0,454545455	6,818181818	5,454545455	2,727272727	5,909090909	1,818181818	3,636363636	3,636363636	5,454545455	3,636363636	5,909090909	10	1,363636364	5,454545455	13,63636364	5	4,545454545	2,727272727	1,818181818	220
PvERF61	7,594936709	2,215189873	5,063291139	4,113924051	4,746835443	6,64556962	3,164556962	1,898734177	2,848101266	7,911392405	2,53164557	7,911392405	6,012658228	4,430379747	4,746835443	12,97468354	6,962025316	3,797468354	1,265822785	3,164556962	316
PvERF72	14,15525114	0	7,305936073	4,109589041	5,936073059	7,305936073	2,283105023	1,826484018	4,566210046	4,566210046	0,913242009	5,936073059	8,219178082	3,196347032	7,305936073	9,132420091	4,109589041	6,392694064	0,913242009	1,826484018	219
PvERF87	10,52631579	0,4784689	7,177033493	8,133971292	5,263157895	5,741626794	1,435406699	3,349282297	4,306220096	6,220095694	3,349282297	3,827751196	4,784688995	1,913875598	7,177033493	13,87559809	2,870813397	5,263157895	1,435406699	2,870813397	209
PvERF91	9,154929577	1,408450704	4,225352113	9,154929577	2,816901408	8,450704225	2,816901408	2,112676056	7,746478873	6,338028169	0,704225352	2,816901408	7,746478873	3,521126761	8,450704225	9,85915493	2,816901408	3,521126761	1,408450704	4,929577465	142
PvERF100	8,018867925	1,41509434	4,716981132	8,490566038	2,830188679	8,490566038	2,358490566	2,830188679	6,132075472	8,490566038	4,245283019	3,301886792	3,773584906	2,358490566	7,075471698	13,67924528	1,886792453	6,132075472	1,41509434	2,358490566	212
PvERF101	5,652173913	1,739130435	4,782608696	8,695652174	2,608695652	5,217391304	2,608695652	2,608695652	8,260869565	9,130434783	3,043478261	4,782608696	6,52173913	4,782608696	7,391304348	11,30434783	3,913043478	4,347826087	1,304347826	1,304347826	230
PvERF119	13,02083333	1,5625	3,645833333	8,333333333	3,125	5,208333333	2,604166667	3,125	6,770833333	7,291666667	1,5625	5,729166667	7,291666667	2,083333333	8,333333333	7,8125	3,125	4,6875	2,083333333	2,604166667	192
PvERF128.1	5,048076923	0,480769231	3,365384615	8,173076923	4,567307692	6,490384615	1,923076923	1,682692308	3,605769231	7,211538462	3,365384615	6,25	11,29807692	8,894230769	5,769230769	10,81730769	4,567307692	3,125	2,163461538	1,201923077	416
PvERF160	7,132459971	1,455604076	3,784570597	6,113537118	2,620087336	8,442503639	4,075691412	2,620087336	4,22125182	5,676855895	3,493449782	5,676855895	4,949053857	5,676855895	4,949053857	12,51819505	5,38573508	6,113537118	1,310043668	3,784570597	687
PvERF176	10,24590164	0	4,508196721	6,967213115	3,278688525	2,868852459	2,049180328	3,68852459	4,508196721	8,606557377	2,459016393	3,68852459	7,37704918	2,459016393	5,327868852	15,57377049	6,967213115	4,918032787	2,459016393	2,049180328	244
PvERF3	7,843137255	1,307189542	3,921568627	13,7254902	3,921568627	5,22875817	1,307189542	1,307189542	13,07189542	4,575163399	3,921568627	4,575163399	3,921568627	1,307189542	6,535947712	11,11111111	2,614379085	7,189542484	1,307189542	1,307189542	153
PvERF6	5,652173913	1,304347826	6,086956522	8,695652174	2,173913043	5,217391304	6,086956522	4,782608696	5,217391304	7,391304348	2,173913043	4,782608696	5,217391304	4,347826087	7,826086957	9,565217391	6,086956522	4,782608696	2,173913043	0,434782609	230
PvERF45	8,870967742	0,403225806	4,435483871	3,629032258	5,241935484	8,870967742	3,225806452	1,612903226	4,435483871	6,451612903	1,209677419	4,435483871	8,064516129	5,241935484	4,435483871	13,30645161	8,064516129	4,032258065	1,209677419	2,822580645	248
PvERF54	10,71428571	1,785714286	5,357142857	9,821428571	4,464285714	3,571428571	3,125	1,339285714	4,910714286	6,25	3,571428571	2,678571429	4,910714286	3,125	9,375	8,928571429	5,357142857	6,25	1,785714286	2,678571429	224
PvERF67	11,56716418	0	4,47761194	4,47761194	4,47761194	11,19402985	1,865671642	0,373134328	1,492537313	6,71641791	2,23880597	1,865671642	11,19402985	4,850746269	7,462686567	14,17910448	2,611940299	3,358208955	2,611940299	2,985074627	268
PvERF70	5,236907731	2,493765586	6,234413965	11,22194514	3,990024938	3,740648379	0,249376559	4,488778055	5,486284289	7,980049875	2,743142145	7,2319202	3,241895262	4,239401496	5,735660848	11,97007481	6,483790524	3,740648379	1,246882793	2,244389027	401
PvERF96.1	11,61825726	1,244813278	7,053941909	5,394190871	2,074688797	7,883817427	0,829875519	2,904564315	8,298755187	9,128630705	1,659751037	3,734439834	7,053941909	2,904564315	5,394190871	8,713692946	3,734439834	4,979253112	2,489626556	2,904564315	241
PvERF121	6,954436451	0,959232614	5,275779376	7,194244604	1,438848921	6,954436451	2,158273381	2,398081535	7,673860911	7,913669065	2,637889688	4,316546763	4,79616307	4,076738609	6,23501199	12,23021583	5,995203837	4,316546763	1,918465228	4,556354916	417
PvERF156	12,63736264	0	5,494505495	6,043956044	3,296703297	5,494505495	2,747252747	2,747252747	2,197802198	6,043956044	6,593406593	4,395604396	7,692307692	3,296703297	7,142857143	9,340659341	6,593406593	3,296703297	3,296703297	1,648351648	182
PvERF10	9,259259259	1,111111111	5,925925926	6,296296296	3,703703704	7,037037037	0,740740741	2,222222222	4,444444444	8,518518519	1,111111111	3,333333333	8,148148148	2,222222222	7,777777778	10,74074074	5,185185185	8,148148148	1,481481481	2,592592593	270
PvERF27	6,25	1,75	5	6	7	5,75	2,75	2	4,25	6,75	2,75	7,5	5,75	3	6	14,75	5,5	4,5	0,5	2,25	400
PvERF29	14,88095238	0	3,571428571	6,547619048	2,976190476	4,166666667	2,380952381	4,166666667	3,571428571	7,142857143	4,761904762	4,166666667	8,333333333	1,785714286	5,952380952	9,523809524	7,142857143	2,976190476	2,976190476	2,976190476	168
PvERF33	7,235142119	1,550387597	5,167958656	6,45994832	3,875968992	9,302325581	1,550387597	3,617571059	7,493540052	6,718346253	1,291989664	6,201550388	5,684754522	2,842377261	5,943152455	9,560723514	4,134366925	7,751937984	1,550387597	2,067183463	387

PvERF35	13,48314607	0,561797753	4,494382022	7,865168539	2,808988764	6,741573034	1,685393258	5,056179775	3,370786517	7,303370787	3,93258427	6,179775281	7,303370787	0,561797753	5,056179775	10,11235955	3,93258427	3,93258427	2,247191011	3,370786517	178
PvERF51	8,571428571	3,333333333	5,238095238	9,047619048	3,80952381	3,333333333	3,333333333	4,761904762	4,761904762	4,761904762	3,333333333	2,857142857	8,571428571	2,380952381	6,666666667	10	6,19047619	6,19047619	1,904761905	0,952380952	210
PvERF56	7,450980392	0,784313725	6,274509804	7,843137255	5,490196078	3,529411765	2,352941176	1,568627451	5,490196078	10,19607843	1,568627451	5,098039216	3,921568627	4,31372549	5,882352941	15,29411765	5,098039216	5,098039216	1,568627451	1,176470588	255
PvERF79	12,37113402	1,030927835	6,18556701	8,762886598	3,608247423	3,092783505	2,06185567	4,12371134	5,670103093	7,216494845	3,092783505	2,06185567	5,670103093	1,030927835	6,701030928	11,34020619	4,639175258	7,216494845	2,577319588	1,546391753	194
PvERF105	7,24137931	1,551724138	5	6,206896552	3,275862069	9,137931034	3,620689655	3,275862069	4,310344828	5,689655172	2,24137931	6,724137931	5,344827586	4,482758621	5,689655172	11,20689655	5,517241379	4,655172414	1,551724138	3,275862069	580
PvERF146	8,659793814	1,855670103	5,154639175	6,18556701	5,360824742	7,628865979	2,06185567	2,886597938	4,948453608	5,154639175	2,06185567	6,391752577	5,154639175	4,12371134	5,567010309	11,34020619	5,567010309	5,154639175	1,649484536	3,092783505	485
PvERF167	6,071428571	1,071428571	5,714285714	8,928571429	3,571428571	6,071428571	1,785714286	4,285714286	5,714285714	9,285714286	1,071428571	5,357142857	7,142857143	5,357142857	5,357142857	8,928571429	6,428571429	3,571428571	2,5	1,785714286	280
PvERF173.1	8,611111111	0,833333333	8,055555556	8,611111111	4,166666667	4,722222222	1,388888889	3,333333333	7,222222222	6,944444444	1,944444444	5,555555556	5,555555556	1,388888889	3,611111111	13,05555556	5,277777778	6,666666667	1,388888889	1,666666667	360
PvERF11	6,483790524	0,748129676	3,740648379	7,2319202	3,241895262	6,982543641	2,743142145	3,49127182	6,234413965	7,481296758	1,74563591	5,735660848	4,239401496	3,241895262	6,982543641	11,97007481	6,483790524	4,738154613	1,74563591	4,738154613	401
PvERF46	6,676783005	0,606980273	5,007587253	5,766312595	2,427921093	6,069802731	3,338391502	2,883156297	3,945371775	7,435508346	3,641881639	5,766312595	5,007587253	6,980273141	5,614567527	13,35356601	5,462822458	4,552352049	1,365705615	4,097116844	659
PvERF48.2	4,081632653	0,29154519	5,539358601	4,664723032	6,413994169	8,163265306	3,498542274	3,206997085	3,498542274	6,705539359	1,749271137	4,081632653	7,580174927	4,956268222	3,790087464	12,53644315	8,163265306	5,539358601	0,874635569	4,664723032	343
PvERF60	8,781869688	1,699716714	5,099150142	5,382436261	4,249291785	8,498583569	1,983002833	1,983002833	3,682719547	8,21529745	2,549575071	6,232294618	5,949008499	3,966005666	4,532577904	11,61473088	6,232294618	5,949008499	2,266288952	1,133144476	353
PvERF66	7,984790875	1,520912548	7,224334601	10,2661597	2,661596958	7,984790875	1,520912548	4,182509506	6,463878327	7,604562738	3,422053232	5,703422053	2,661596958	0,760456274	8,365019011	9,125475285	3,041825095	4,942965779	2,281368821	2,281368821	263
PvERF88	7,142857143	1,298701299	5,194805195	9,090909091	3,896103896	5,844155844	3,896103896	3,246753247	5,844155844	11,68831169	2,597402597	4,545454545	2,597402597	2,597402597	11,03896104	4,545454545	5,194805195	6,493506494	0,649350649	2,597402597	154
PvERF89	7,631578947	1,315789474	10	6,315789474	4,210526316	8,421052632	2,105263158	1,842105263	6,315789474	6,315789474	2,631578947	5,263157895	4,736842105	4,210526316	5,263157895	10,52631579	3,684210526	4,210526316	1,315789474	3,684210526	380
PvERF99	11,16751269	1,015228426	4,060913706	9,137055838	2,030456853	6,598984772	2,538071066	2,030456853	6,091370558	8,629441624	2,030456853	6,091370558	3,553299492	4,060913706	6,091370558	8,629441624	6,091370558	5,583756345	3,553299492	1,015228426	197
PvERF135	7,352941176	0,367647059	3,676470588	6,25	3,492647059	8,455882353	3,492647059	2,389705882	3,125	8,088235294	3,125	8,088235294	3,676470588	6,066176471	4,963235294	11,76470588	5,882352941	4,227941176	1,470588235	4,044117647	544
PvERF145	8,301886792	1,132075472	4,528301887	5,660377358	4,150943396	5,283018868	0,754716981	5,660377358	4,528301887	8,679245283	3,396226415	6,41509434	12,83018868	3,018867925	3,018867925	8,301886792	6,037735849	3,773584906	2,264150943	2,264150943	265
PvERF172	7,913669065	1,798561151	6,115107914	5,395683453	3,956834532	5,755395683	0,71942446	5,035971223	5,395683453	7,553956835	1,798561151	7,913669065	3,956834532	5,395683453	5,755395683	11,15107914	7,553956835	3,956834532	1,438848921	1,438848921	278
PvERF1.1	10	0	4,666666667	6,666666667	5,333333333	10	2	4	2,666666667	6,666666667	0,666666667	8	5,333333333	3,333333333	8	11,33333333	4	2	1,333333333	4	150
PvERF9	5,852842809	1,672240803	4,347826087	6,18729097	2,675585284	5,685618729	3,511705686	4,013377926	4,347826087	7,023411371	3,678929766	5,518394649	5,183946488	6,52173913	5,852842809	12,87625418	5,518394649	4,515050167	1,337792642	3,678929766	598
PvERF18	7,344632768	1,129943503	7,344632768	9,039548023	1,129943503	8,474576271	0,564971751	1,694915254	5,084745763	6,779661017	2,259887006	3,389830508	2,824858757	2,259887006	7,90960452	13,55932203	4,519774011	9,039548023	3,389830508	2,259887006	177
PvERF23.1	11,1332008	0,596421471	4,174950298	5,566600398	4,572564612	7,554671968	3,777335984	3,180914513	4,174950298	5,765407555	1,789264414	5,367793241	3,777335984	5,367793241	4,771371769	12,52485089	5,566600398	4,771371769	1,391650099	4,174950298	503
PvERF26	7,826086957	0	5,652173913	7,391304348	3,913043478	4,782608696	3,043478261	3,47826087	4,782608696	6,52173913	2,608695652	5,217391304	6,956521739	2,173913043	5,652173913	16,52173913	6,956521739	2,608695652	2,173913043	1,739130435	230
PvERF32	6,435643564	1,98019802	6,683168317	9,405940594	3,96039604	5,940594059	1,237623762	3,712871287	5,198019802	7,178217822	3,712871287	6,930693069	3,217821782	4,702970297	5,198019802	9,653465347	6,435643564	4,455445545	1,237623762	2,722772277	404
PvERF58	7,692307692	0,961538462	7,211538462	7,211538462	9,134615385	6,730769231	0	3,846153846	3,365384615	4,326923077	2,884615385	3,365384615	7,692307692	4,326923077	6,25	14,90384615	2,884615385	3,846153846	1,442307692	1,923076923	208
PvERF65	5,583756345	2,030456853	4,568527919	9,644670051	1,52284264	5,076142132	4,060913706	3,045685279	5,583756345	7,614213198	3,045685279	5,076142132	4,568527919	4,060913706	7,614213198	11,16751269	5,076142132	7,614213198	2,538071066	0,507614213	197
PvERF153	9,045226131	1,507537688	3,51758794	8,542713568	1,507537688	9,547738693	1,507537688	2,010050251	7,537688442	8,040201005	2,512562814	5,527638191	4,020100503	1,005025126	6,030150754	15,07537688	3,51758794	5,025125628	2,512562814	2,010050251	199
PvERF169	6,783919598	1,005025126	6,281407035	7,286432161	3,768844221	7,035175879	3,015075377	3,51758794	6,281407035	5,527638191	2,010050251	4,271356784	3,768844221	4,773869347	5,778894472	12,56281407	5,527638191	4,773869347	1,507537688	4,522613065	398
PvERF175.1	7,565789474	0,657894737	4,605263158	6,25	6,25	4,276315789	1,644736842	3,289473684	6,25	8,552631579	0,986842105	4,605263158	10,52631579	6,25	5,263157895	10,52631579	6,25	3,618421053	1,644736842	0,986842105	304
PvERF179	4,761904762	1,587301587	3,571428571	6,746031746	4,365079365	5,158730159	3,174603175	4,365079365	9,920634921	8,333333333	3,174603175	5,158730159	5,158730159	2,380952381	4,761904762	10,71428571	5,952380952	6,349206349	1,19047619	3,174603175	252
PvERF30	7,126436782	0	3,67816092	4,137931034	4,827586207	11,03448276	2,298850575	0,91954023	1,149425287	6,666666667	2,75862069	2,298850575	7,816091954	10,11494253	5,977011494	14,02298851	6,896551724	4,597701149	1,149425287	2,528735632	435
PvERF55	11,37724551	0,598802395	5,389221557	4,191616766	2,395209581	5,988023952	1,796407186	2,395209581	3,592814371	10,17964072	0,598802395	2,395209581	11,9760479	1,796407186	8,982035928	9,580838323	4,790419162	7,185628743	2,994011976	1,796407186	167
PvERF69	8,587257618	1,385041551	5,540166205	6,371191136	4,432132964	9,695290859	1,385041551	2,216066482	6,371191136	7,756232687	1,108033241	4,155124654	4,986149584	3,878116343	7,202216066	9,141274238	4,155124654	7,756232687	1,385041551	2,493074792	361
PvERF92	9,661835749	1,449275362	7,246376812	4,830917874	3,8647343	3,8647343	3,381642512	1,93236715	8,212560386	7,729468599	1,93236715	4,830917874	4,830917874	3,8647343	6,763285024	11,11111111	6,280193237	4,830917874	1,449275362	1,93236715	207
PvERF98	4,571428571	2,857142857	4,761904762	10,47619048	2,666666667	7,80952381	3,619047619	4	7,428571429	7,619047619	2,476190476	6,476190476	4,571428571	3,80952381	4,761904762	10,0952381	4	4,761904762	0,952380952	2,285714286	525
PvERF123	7,598784195	0,911854103	4,863221884	3,951367781	3,343465046	6,079027356	4,863221884	0,607902736	3,343465046	5,167173252	0,911854103	6,686930091	6,382978723	5,167173252	6,079027356	14,58966565	8,814589666	6,079027356	1,519756839	3,039513678	329
PvERF126	13,24200913	2,283105023	7,305936073	2,739726027	5,479452055	5,936073059	2,739726027	0,913242009	3,196347032	6,392694064	1,369863014	4,566210046	10,95890411	0,456621005	8,675799087	9,589041096	6,849315068	4,109589041	0,913242009	2,283105023	219
PvERF136	9,045226131	1,507537688	3,51758794	8,542713568	1,507537688	9,547738693	1,507537688	2,010050251	7,537688442	8,040201005	2,512562814	5,527638191	4,020100503	1,005025126	6,030150754	15,07537688	3,51758794	5,025125628	2,512562814	2,010050251	199
PvERF139	6,026058632	1,954397394	5,211726384	6,188925081	3,583061889	6,188925081	1,791530945	4,885993485	3,25732899	7,491856678	1,954397394	9,283387622	4,397394137	6,351791531	4,560260586	11,40065147	6,840390879	4,234527687	0,977198697	3,42019544	614
PvERF175.2	10,17699115	0	3,539823009	7,079646018	3,982300885	4,424778761	2,654867257	2,212389381	8,849557522	5,752212389	0,884955752	3,097345133	7,96460177	4,867256637	7,079646018	12,83185841	6,194690265	5,309734513	1,327433628	1,769911504	226
PvERF12	5,079365079	0,952380952	7,936507937	11,11111111	4,126984127	6,349206349	1,587301587	2,857142857	7,936507937	6,666666667	3,492063492	4,761904762	3,80952381	2,222222222	4,761904762	11,11111111	3,80952381	6,984126984	1,26984127	3,174603175	315
PvERF42	6,633906634	0,245700246	5,896805897	5,896805897	4,668304668	6,633906634	4,176904177	2,211302211	3,685503686	6,388206388	2,702702703	6,388206388	9,336609337	7,862407862	5,651105651	10,31941032	3,931203931	3,194103194	2,702702703	1,474201474	407
PvERF71	7,488986784	1,321585903	4,405286344	5,286343612	6,607929515	3,524229075	3,964757709	4,405286344	7,04845815	8,370044053	2,643171806	5,286343612	7,488986784	1,762114537	6,167400881	11,01321586	6,607929515	3,524229075	1,762114537	1,321585903	227
PvERF74	8,781869688	1,983002833	8,498583569	9,065155807	5,382436261	4,249291785	0,566572238	3,399433428	5,949008499	6,798866856	1,133144476	3,966005666	7,648725212	3,399433428	5,099150142	11,61473088	5,382436261	3,966005666	1,416430595	1,699716714	353
PvERF77	12,24489796	0	6,12244898	10,20408163	4,761904762	9,523809524	0,680272109	1,360544218	4,761904762	4,761904762	2,040816327	4,081632653	4,081632653	2,721088435	10,88435374	8,843537415	4,081632653	3,401360544	2,040816327	3,401360544	147
PvERF94	8,02919708	1,459854015	8,759124088	8,759124088	4,379562044	5,109489051	3,649635036	1,459854015	8,759124088	8,759124088	2,919708029	2,189781022	5,109489051	1,459854015	5,839416058	11,67883212	3,649635036	2,919708029	1,459854015	3,649635036	137
PvERF102	9,030100334	1,337792642	5,685618729	8,361204013	4,682274247	7,023411371	1,003344482	4,347826087	7,023411371	4,347826087	2,006688963	6,688963211	6,02006689	4,682274247	5,685618729	7,357859532	3,010033445	5,685618729	1,672240803	4,347826087	299
PvERF109	9,42408377	1,570680628	5,235602094	6,806282723	7,853403141	5,759162304	0,523560209	2,094240838	6,806282723	8,90052356	2,094240838	4,712041885	4,188481675	4,188481675	8,376963351	7,329842932	5,235602094	5,235602094	3,141361257	0,523560209	191
PvERF110.2	13,33333333	0,476190476	4,761904762	4,761904762	3,333333333	6,666666667	2,380952381	3,333333333	2,857142857	7,142857143	4,285714286	7,619047619	7,142857143	3,333333333	4,285714286	9,523809524	7,142857143	3,80952381	1,428571429	2,380952381	210
PvERF111	7,582938389	1,421800948	5,213270142	9,952606635	5,687203791	4,739336493	1,895734597	3,791469194	7,582938389	6,63507109	2,369668246	4,739336493	6,63507109	1,895734597	6,161137441	10,90047393	5,213270142	4,265402844	0,947867299	2,369668246	211
PvERF112	7,73480663	0,552486188	5,524861878	9,392265193	2,762430939	6,629834254	3,314917127	4,972375691	7,182320442	6,077348066	2,209944751	6,629834254	4,972375691	0,552486188	8,287292818	9,944751381	3,867403315	6,629834254	0,552486188	2,209944751	181
PvERF115	6,145251397	1,675977654	7,82122905	7,541899441	4,189944134	7,262569832	2,793296089	3,631284916	8,100558659	5,586592179	1,396648045	4,748603352	3,351955307	3,910614525	5,865921788	11,45251397	4,469273743	5,027932961	1,675977654	3,351955307	358
PvERF124	9,121621622	0,675675676	3,378378378	5,405405405	3,716216216	3,378378378	1,013513514	1,689189189	2,364864865	5,405405405	2,364864865	4,72972973	7,094594595	3,378378378	8,445945946	18,91891892	8,108108108	6,081081081	1,351351351	3,378378378	296
PvERF133	7,8125	0,260416667	8,59375	7,291666667	5,208333333	6,770833333	0,260416667	3,385416667	6,770833333	7,03125	1,822916667	4,166666667	5,989583333	4,6875	5,729166667	10,67708333	4,166666667	5,46875	1,822916667	2,083333333	384
PvERF149	11,96172249	0,956937799	3,349282297	5,741626794	2,870813397	8,133971292	2,392344498	3,349282297	7,177033493	7,177033493	0,956937799	3,827751196	4,306220096	1,913875598	8,612440191	8,133971292	7,655502392	7,177033493	1,435406699	2,870813397	209
PvERF166	8,733624454	2,183406114	7,423580786	7,860262009	6,550218341	4,366812227	3,056768559	2,620087336	5,240174672	5,240174672	2,620087336	6,113537118	4,366812227	3,056768559	3,493449782	10,91703057	6,986899563	4,80349345	2,620087336	1,746724891	229
PvERF170	14,36170213	0	5,85106383	5,319148936	4,255319149	7,446808511	1,063829787	3,191489362	2,127659574	6,382978723	4,255319149	7,978723404	6,382978723	1,063829787	6,382978723	10,63829787	3,723404255	5,319148936	1,595744681	2,659574468	188
PvERF4	11,69590643	1,169590643	5,263157895	9,356725146	1,754385965	10,52631579	2,339181287	2,339181287	4,678362573	6,432748538	2,339181287	2,339181287	4,678362573	1,754385965	11,11111111	5,847953216	4,093567251	7,602339181	2,339181287	2,339181287	171
PvERF21	8,745247148	0,570342205	4,562737643	3,6121673	5,513307985	8,935361217	2,661596958	3,422053232	3,802281369	5,703422053	1,140684411	5,703422053	6,273764259	3,6121673	4,942965779	14,82889734	8,935361217	2,661596958	1,140684411	3,231939163	526
PvERF24	8,130081301	2,032520325	6,504065041	6,097560976	3,658536585	3,25203252	1,62601626	2,032520325	4,87804878	7,723577236	2,845528455	5,691056911	6,910569106	1,62601626	6,504065041	13,41463415	6,504065041	6,097560976	1,62601626	2,845528455	246
PvERF52	6,853582555	0,62305296	4,984423676	5,607476636	3,426791277	6,853582555	3,115264798	3,115264798	6,230529595	7,476635514	2,180685358	4,672897196	8,411214953	3,426791277	7,165109034	9,657320872	6,853582555	3,115264798	1,557632399	4,672897196	321
PvERF76	7,213930348	0,746268657	4,975124378	4,726368159	3,482587065	5,223880597	2,487562189	4,47761194	6,965174129	8,208955224	3,233830846	5,721393035	5,472636816	4,975124378	3,482587065	12,43781095	7,960199005	4,47761194	1,741293532	1,990049751	402
PvERF81	6,944444444	1,19047619	6,349206349	5,753968254	4,166666667	9,722222222	3,76984127	2,579365079	5,753968254	6,349206349	1,785714286	4,563492063	5,753968254	3,968253968	6,547619048	12,6984127	3,373015873	4,365079365	1,587301587	2,777777778	504
PvERF103	10,74380165	1,652892562	4,545454545	10,33057851	2,066115702	7,438016529	2,066115702	4,132231405	3,719008264	6,198347107	2,892561983	1,652892562	6,198347107	2,479338843	5,785123967	15,70247934	3,305785124	4,545454545	2,892561983	1,652892562	242
PvERF25	7,826086957	1,086956522	4,565217391	5,869565217	5	7,391304348	2,608695652	3,47826087	5,217391304	6,086956522	1,52173913	5,434782609	5,652173913	5	5,869565217	11,73913043	5,434782609	6,086956522	1,52173913	2,608695652	460
PvERF44	6,875	2,8125	5,625	9,6875	6,25	6,875	2,8125	3,75	8,125	6,5625	2,1875	3,75	2,5	4,0625	5,3125	6,5625	4,6875	5,9375	2,1875	3,4375	320
PvERF78.2	7,888040712	0,254452926	5,597964377	7,37913486	3,816793893	8,142493639	2,03562341	4,325699746	6,361323155	7,124681934	1,781170483	4,834605598	2,290076336	4,071246819	6,615776081	11,45038168	5,34351145	4,834605598	1,526717557	4,325699746	393
PvERF93	9,061488673	1,618122977	6,796116505	5,177993528	4,207119741	3,883495146	3,236245955	3,55987055	5,501618123	6,472491909	2,265372168	5,177993528	5,501618123	3,883495146	4,854368932	16,18122977	8,090614887	1,618122977	1,618122977	1,294498382	309
PvERF122	10	1,6	9,6	4,8	4,8	4	2,8	3,2	7,2	6	1,2	4,4	8,8	2,4	5,6	7,6	8	4,4	1,6	2	250
PvERF129	7,917888563	1,759530792	7,917888563	9,384164223	4,398826979	3,812316716	2,639296188	3,225806452	4,398826979	8,797653959	1,173020528	3,519061584	7,038123167	2,639296188	6,158357771	8,504398827	6,744868035	6,451612903	1,173020528	2,346041056	341
PvERF137.2	5,281690141	2,112676056	8,450704225	8,802816901	7,394366197	2,816901408	3,169014085	4,577464789	6,690140845	5,985915493	2,112676056	3,169014085	3,873239437	4,225352113	5,985915493	7,746478873	5,281690141	7,394366197	1,76056338	3,169014085	284
PvERF148	13,92405063	0	3,797468354	6,962025316	6,962025316	5,063291139	3,164556962	1,898734177	7,594936709	7,594936709	0,632911392	1,898734177	11,39240506	1,265822785	8,227848101	2,53164557	6,329113924	5,696202532	2,53164557	2,53164557	158
PvERF158	7,518796992	1,879699248	2,631578947	7,518796992	3,007518797	5,263157895	4,135338346	3,383458647	4,135338346	6,766917293	6,390977444	3,383458647	6,390977444	5,639097744	4,135338346	11,65413534	7,142857143	3,007518797	2,255639098	3,759398496	266
PvERF14	8,385093168	2,48447205	4,658385093	5,590062112	3,416149068	5,900621118	0,931677019	4,037267081	5,900621118	9,627329193	1,863354037	5,900621118	5,279503106	2,795031056	5,900621118	14,59627329	4,658385093	3,726708075	1,552795031	2,795031056	322
PvERF28	7,894736842	2,923976608	4,678362573	11,40350877	4,385964912	7,602339181	2,046783626	3,50877193	4,970760234	6,725146199	1,461988304	4,678362573	4,678362573	2,923976608	6,432748538	8,479532164	4,385964912	4,093567251	1,169590643	5,555555556	342
PvERF37	9,836065574	3,278688525	9,836065574	6,557377049	2,18579235	7,103825137	1,092896175	3,278688525	4,918032787	6,010928962	2,732240437	1,639344262	9,289617486	2,732240437	7,650273224	7,650273224	4,371584699	4,371584699	3,825136612	1,639344262	183
PvERF38	8,108108108	2,027027027	4,054054054	6,081081081	4,054054054	4,72972973	1,351351351	6,081081081	2,702702703	7,432432432	3,378378378	5,405405405	11,48648649	2,702702703	6,756756757	10,13513514	4,72972973	3,378378378	1,351351351	4,054054054	148
PvERF86	9,030100334	1,337792642	5,685618729	8,361204013	4,682274247	7,023411371	1,003344482	4,347826087	7,023411371	4,347826087	2,006688963	6,688963211	6,02006689	4,682274247	5,685618729	7,357859532	3,010033445	5,685618729	1,672240803	4,347826087	299
PvERF97	5,240174672	1,746724891	4,80349345	8,733624454	2,620087336	5,240174672	2,620087336	2,620087336	8,296943231	9,170305677	3,056768559	4,80349345	6,550218341	4,80349345	7,423580786	11,35371179	3,930131004	4,366812227	1,310043668	1,310043668	229
PvERF110.1	6,806282723	1,570680628	1,570680628	9,42408377	2,617801047	2,617801047	3,664921466	4,188481675	5,759162304	8,90052356	3,141361257	3,664921466	5,235602094	6,282722513	8,90052356	8,90052356	7,329842932	4,712041885	2,094240838	2,617801047	191
PvERF128.2	11,91489362	1,70212766	7,234042553	3,404255319	2,553191489	2,127659574	1,276595745	3,829787234	5,106382979	7,234042553	4,255319149	2,553191489	6,382978723	2,978723404	3,829787234	20,85106383	2,978723404	4,255319149	2,127659574	3,404255319	235
PvERF144	7,514450867	1,445086705	4,046242775	8,092485549	4,046242775	5,780346821	3,179190751	2,601156069	4,624277457	6,069364162	2,601156069	8,670520231	4,046242775	4,335260116	6,936416185	9,248554913	7,225433526	3,179190751	1,734104046	4,624277457	346
PvERF147	7,109004739	0,947867299	3,791469194	7,582938389	4,739336493	4,265402844	3,317535545	2,369668246	8,056872038	8,056872038	2,843601896	4,265402844	6,161137441	3,317535545	6,63507109	10,90047393	5,687203791	5,687203791	1,421800948	2,843601896	211
PvERF152	7,4204947	1,766784452	6,007067138	7,77385159	3,180212014	2,826855124	2,473498233	3,180212014	4,946996466	6,007067138	1,766784452	4,946996466	5,300353357	3,180212014	4,593639576	18,02120141	10,9540636	1,766784452	1,766784452	2,120141343	283
PvERF161	8,258050213	1,29135953	5,472112915	6,916809843	4,060669881	6,28129099	2,486652257	3,133255741	5,226403591	6,898335458	2,385043138	5,098930333	6,000480334	4,116093037	6,151970293	11,30817122	5,564484842	4,884627464	1,677474182	2,787784736	286,3968254
PvERF163																					
Avg.																					

Data Filename:	PvAP2-ERF final aa seq V2.mas
Data Title:	fasta file
Amino acid Frequencies	
Sites Used:	All selected

Table S4: Summary of *AP2/ERF transcription factor gene* family factors of plants

Species	Number of AP2/ERF	Genome size (Mb)	AP2/ERF/Mb
Arabidopsis thaliana	122	135	0,9037
Daucus carota	267	480	0,5563
Brassica rapa	248	507	0,4892
Populus trichocarpa	200	423	0,4728
Salix arbutifolia	173	425	0,4071
Brassica oleracea	226	603	0,3748
Oryza sativa	139	372	0,3737
Cucumis melo	136	375	0,3627
Ricinus cummunis	114	320	0,3563
Setaria italica	171	515	0,3320
Phaseolus vulgaris	180	587	0,3066
Eucalyptus grandis	209	691	0,3025
Citrus sinensis	108	380	0,2842
Cucumis sativus	103	367	0,2807
Vitis vinifera	132	487	0,2710
Malus domestica	209	881	0,2372
Solanum tuberosum	155	800	0,1938
Solanum lycopersicum	112	950	0,1179
Glycine max	98	975	0,1005
Zea mays	184	2067	0,0890
Phyllostachys edulis	116	2050	0,0566
Triticum aestivum	117	17000	0,0069

Table S5: Tandem and segmental duplications of PvAP2-ERF proteins

Gen ID	Phytozome Identfier	Chromosome: Start - Finish	Gen ID 1	Phytozome Identfier	Chromosome: Start - Finish	Identity	e-value	Score
PvAP2-ERF1	Phvul.001G010400	Chr01:931454-932855	PvAP2-ERF140	Phvul.009G013200	Chr09:2059353-2060102	72%	2,00E-71	211
PvAP2-ERF2	Phvul.001G023700	Chr01:2101326-2102436	PvAP2-ERF127	Phvul.008G098900	Chr08:10466381-10466854	70%	6,00E-66	190
PvAP2-ERF3	Phvul.001G031200	Chr01:2907733-2912245	PvAP2-ERF52	Phvul.003G144500	Chr03:34150168-34153951	81%	7,00E-121	394
PvAP2-ERF3	Phvul.001G031200	Chr01:2907733-2912245	PvAP2-ERF60	Phvul.003G232600	Chr03:45498765-45502904	89%	0,00E+00	640
PvAP2-ERF3	Phvul.001G031200	Chr01:2907733-2912245	PvAP2-ERF167	Phvul.010G130200	Chr10:40027019-40030846	78%	1,00E-145	483
PvAP2-ERF4	Phvul.001G044500	Chr01:4680371-4681060	PvAP2-ERF143	Phvul.009G084400	Chr09:13327339-13328429	70%	5,00E-48	149
PvAP2-ERF6	Phvul.001G073800	Chr01:10050549-10051844	PvAP2-ERF44	Phvul.002G310200	Chr02:47177131-47178333	72%	6,00E-62	185
PvAP2-ERF6	Phvul.001G073800	Chr01:10050549-10051844	PvAP2-ERF146	Phvul.009G109600	Chr09:16431520-16432907	72%	8,00E-76	219
PvAP2-ERF6	Phvul.001G073800	Chr01:10050549-10051844	PvAP2-ERF79	Phvul.005G170600	Chr05:39420680-39421777	70%	6,00E-61	196
PvAP2-ERF10	Phvul.001G131300	Chr01:37223732-37227544	PvAP2-ERF122	Phvul.008G043500	Chr08:3742495-3745281	73%	2,00E-68	213
PvAP2-ERF11	Phvul.001G136100	Chr01:38195399-38196241	PvAP2-ERF116	Phvul.007G255100	Chr07:49321858-49323149	80%	2,00E-137	382
PvAP2-ERF19	Phvul.001G160500	Chr01:42037682-42038650	PvAP2-ERF105	Phvul.007G193300	Chr07:43116184-43117296	74%	2,00E-74	218
PvAP2-ERF22	Phvul.001G251200	Chr01:50886895-50888163	PvAP2-ERF40	Phvul.002G254500	Chr02:42080727-42082576	71%	8,00E-84	246
PvAP2-ERF22	Phvul.001G251200	Chr01:50886895-50888163	PvAP2-ERF141	Phvul.009G029600	Chr09:6536069-6538397	72%	7,00E-76	227
PvAP2-ERF23	Phvul.002G009100	Chr02:1058028-1061555	PvAP2-ERF3	Phvul.001G031200	Chr01:2907733-2912245	78%	0	524
PvAP2-ERF23	Phvul.002G009100	Chr02:1058028-1061555	PvAP2-ERF60	Phvul.003G232600	Chr03:45498765-45502904	76%	0,00E+00	777
PvAP2-ERF23	Phvul.002G009100	Chr02:1058028-1061555	PvAP2-ERF142	Phvul.009G074300	Chr09:12286871-12290865	71%	3,00E-173	500
PvAP2-ERF31	Phvul.002G056800	Chr02:5810982-5811876	PvAP2-ERF58	Phvul.003G222600	Chr03:44044998-44045690	71%	1,00E-63	187
PvAP2-ERF34	Phvul.002G153900	Chr02:29528341-29528991	PvAP2-ERF57	Phvul.003G212800	Chr03:42804542-42805711	86%	1,00E-71	229
PvAP2-ERF35	Phvul.002G154000	Chr02:29572213-29572719	PvAP2-ERF56	Phvul.003G212700	Chr03:42784293-42784829	71%	2,00E-60	176
PvAP2-ERF42	Phvul.002G281300	Chr02:44487105-44490185	PvAP2-ERF150	Phvul.009G161000	Chr09:23312039-23314113	71%	2,00E-47	147
PvAP2-ERF43	Phvul.002G295700	Chr02:45929067-45930538	PvAP2-ERF36	Phvul.002G162500	Chr02:30433762-30435007	71%	6,00E-85	219
PvAP2-ERF46	Phvul.003G035500	Chr03:3558866-3561380	PvAP2-ERF94	Phvul.007G027000	Chr07:1987568-1990173	76%	9,00E-97	283
PvAP2-ERF47	Phvul.003G064800	Chr03:9171392-9172486	PvAP2-ERF33	Phvul.002G149500	Chr02:28972219-28973736	72%	1,00E-85	285
PvAP2-ERF48	Phvul.003G069000	Chr03:10190940-10193616	PvAP2-ERF81	Phvul.005G183700	Chr05:40443575-40446525	76%	3,00E-87	275
PvAP2-ERF48	Phvul.003G069000	Chr03:10190940-10193616	PvAP2-ERF122	Phvul.008G043500	Chr08:3742495-3745281	80%	2,00E-136	388
PvAP2-ERF48	Phvul.003G069000	Chr03:10190940-10193616	PvAP2-ERF152	Phvul.009G206500	Chr09:30520376-30524486	78%	2,00E-93	306
PvAP2-ERF48	Phvul.003G069000	Chr03:10190940-10193616	PvAP2-ERF179	Phvul.011G187400	Chr11:46403162-46408304	79%	2,00E-96	331
PvAP2-ERF49	Phvul.003G102500	Chr03:25181566-25183062	PvAP2-ERF87	Phvul.006G163100	Chr06:27418948-27419898	71%	3,00E-125	354
PvAP2-ERF51	Phvul.003G111800	Chr03:28178527-28180564	PvAP2-ERF98	Phvul.007G102800	Chr07:11606561-11608030	85%	4,00E-173	479
PvAP2-ERF56	Phvul.003G212700	Chr03:42784293-42784829	PvAP2-ERF35	Phvul.002G154000	Chr02:29572213-29572719	71%	2,00E-58	171
PvAP2-ERF61	Phvul.003G241700	Chr03:46504117-46504556	PvAP2-ERF24	Phvul.002G016700	Chr02:1837177-1838211	70%	6,00E-48	153
PvAP2-ERF62	Phvul.003G241900	Chr03:46544077-46547812	PvAP2-ERF25	Phvul.002G016900	Chr02:1860815-1864323	82%	0	757
PvAP2-ERF62	Phvul.003G241900	Chr03:46544077-46547812	PvAP2-ERF133	Phvul.008G185400	Chr08:48884848-48891709	77%	2,00E-133	312
PvAP2-ERF67	Phvul.004G069900	Chr04:10524334-10526517	PvAP2-ERF7	Phvul.001G084000	Chr01:14986698-14988815	72%	1,00E-53	166
PvAP2-ERF68	Phvul.004G081200	Chr04:14013830-14015119	PvAP2-ERF9	Phvul.001G111800	Chr01:30786425-30787261	76%	1,00E-53	303
PvAP2-ERF70	Phvul.004G122000	Chr04:39326716-39327951	PvAP2-ERF95	Phvul.007G066500	Chr07:5928454-5929059	73%	1,00E-77	223

PvAP2-ERF70	Phvul.004G122000	Chr04:39326716-39327951	PvAP2-ERF34	Phvul.002G153900	Chr02:29528341-29528991	72%	1,00E-62	186
PvAP2-ERF70	Phvul.004G122000	Chr04:39326716-39327951	PvAP2-ERF57	Phvul.003G212800	Chr03:42804542-42805711	72%	4,00E-61	184
PvAP2-ERF72	Phvul.004G169800	Chr04:45126736-45127899	PvAP2-ERF44	Phvul.002G310200	Chr02:47177131-47178333	71%	8,00E-77	223
PvAP2-ERF78	Phvul.005G138300	Chr05:36714215-36718541	PvAP2-ERF20	Phvul.001G174400	Chr01:43723288-43726156	72%	2,00E-126	367
PvAP2-ERF78	Phvul.005G138300	Chr05:36714215-36718541	PvAP2-ERF173	Phvul.011G071100	Chr11:6338128-6342324	78%	0,00E+00	565
PvAP2-ERF79	Phvul.005G170600	Chr05:39420680-39421777	PvAP2-ERF168	Phvul.010G146600	Chr10:41789538-41790170	73%	6,00E-67	196
PvAP2-ERF83	Phvul.006G080000	Chr06:19885920-19886712	PvAP2-ERF137	Phvul.008G246000	Chr08:56074147-56075959	70%	6,00E-63	188
PvAP2-ERF84	Phvul.006G106100	Chr06:22259920-22260531	PvAP2-ERF99	Phvul.007G121200	Chr07:21423708-21424172	70%	9,00E-56	164
PvAP2-ERF87	Phvul.006G163100	Chr06:27418948-27419898	PvAP2-ERF49	Phvul.003G102500	Chr03:25181566-25183062	74%	1,00E-122	348
PvAP2-ERF93	Phvul.007G002900	Chr07:201437-202399	PvAP2-ERF51	Phvul.003G111800	Chr03:28178527-28180564	71%	2,00E-54	172
PvAP2-ERF94	Phvul.007G027000	Chr07:1987568-1990173	PvAP2-ERF46	Phvul.003G035500	Chr03:3558866-3561380	73%	9,00E-96	281
PvAP2-ERF96	Phvul.007G082000	Chr07:8065792-8067856	PvAP2-ERF69	Phvul.004G092100	Chr04:24438791-24441164	75%	4,00E-56	208
PvAP2-ERF104	Phvul.007G135900	Chr07:33583547-33584310	PvAP2-ERF115	Phvul.007G241800	Chr07:48154568-48155203	70%	5,00E-78	224
PvAP2-ERF109	Phvul.007G193900	Chr07:43237645-43238058	PvAP2-ERF154	Phvul.009G240800	Chr09:35418233-35418610	70%	1,00E-45	134
PvAP2-ERF110	Phvul.007G217800	Chr07:45677796-45679996	PvAP2-ERF157	Phvul.009G262200	Chr09:37365318-37367770	70%	4,00E-53	167
PvAP2-ERF111	Phvul.007G222500	Chr07:46214380-46215168	PvAP2-ERF174	Phvul.011G091400	Chr11:9148323-9148991	70%	1,00E-55	166
PvAP2-ERF112	Phvul.007G222600	Chr07:46221519-46222906	PvAP2-ERF21	Phvul.001G187100	Chr01:45289609-45290175	72%	6,00E-75	215
PvAP2-ERF113	Phvul.007G240200	Chr07:48016768-48020784	PvAP2-ERF20	Phvul.001G174400	Chr01:43723288-43726156	71%	5,00E-57	237
PvAP2-ERF116	Phvul.007G255100	Chr07:49321858-49323149	PvAP2-ERF11	Phvul.001G136100	Chr01:38195399-38196241	78%	2,00E-137	382
PvAP2-ERF119	Phvul.007G273000	Chr07:51127595-51128470	PvAP2-ERF84	Phvul.006G106100	Chr06:22259920-22260531	71%	1,00E-57	171
PvAP2-ERF121	Phvul.008G039300	Chr08:3311273-3312478	PvAP2-ERF65	Phvul.004G031900	Chr04:3566458-3567993	76%	5,00E-163	471
PvAP2-ERF121	Phvul.008G039300	Chr08:3311273-3312478	PvAP2-ERF38	Phvul.002G168900	Chr02:31177497-31178525	73%	2,00E-56	179
PvAP2-ERF125	Phvul.008G052000	Chr08:4601738-4606123	PvAP2-ERF3	Phvul.001G031200	Chr01:2907733-2912245	71%	1,00E-120	368
PvAP2-ERF125	Phvul.008G052000	Chr08:4601738-4606123	PvAP2-ERF52	Phvul.003G144500	Chr03:34150168-34153951	81%	4,00E-127	379
PvAP2-ERF125	Phvul.008G052000	Chr08:4601738-4606123	PvAP2-ERF142	Phvul.009G074300	Chr09:12286871-12290865	70%	4,00E-117	422
PvAP2-ERF125	Phvul.008G052000	Chr08:4601738-4606123	PvAP2-ERF172	Phvul.011G058000	Chr11:5016893-5020109	72%	2,00E-135	402
PvAP2-ERF129	Phvul.008G141000	Chr08:23916631-23917738	PvAP2-ERF163	Phvul.010G054000	Chr10:8503366-8504597	73%	3,00E-94	272
PvAP2-ERF129	Phvul.008G141000	Chr08:23916631-23917738	PvAP2-ERF58	Phvul.003G222600	Chr03:44044998-44045690	70%	4,00E-58	177
PvAP2-ERF131	Phvul.008G165000	Chr08:42621369-42622218	PvAP2-ERF147	Phvul.009G123300	Chr09:18302891-18303953	78%	2,00E-88	258
PvAP2-ERF132	Phvul.008G172200	Chr08:45116054-45117855	PvAP2-ERF141	Phvul.009G029600	Chr09:6536069-6538397	70%	1,00E-117	335
PvAP2-ERF132	Phvul.008G172200	Chr08:45116054-45117855	PvAP2-ERF22	Phvul.001G251200	Chr01:50886895-50888163	72%	6,00E-69	208
PvAP2-ERF138	Phvul.008G253600	Chr08:56759415-56762319	PvAP2-ERF53	Phvul.003G153100	Chr03:35757558-35760426	74%	1,00E-123	350
PvAP2-ERF138	Phvul.008G253600	Chr08:56759415-56762319	PvAP2-ERF81	Phvul.005G183700	Chr05:40443575-40446525	73%	9,00E-120	338
PvAP2-ERF138	Phvul.008G253600	Chr08:56759415-56762319	PvAP2-ERF152	Phvul.009G206500	Chr09:30520376-30524486	70%	5,00E-128	361
PvAP2-ERF138	Phvul.008G253600	Chr08:56759415-56762319	PvAP2-ERF179	Phvul.011G187400	Chr11:46403162-46408304	70%	1,00E-106	308
PvAP2-ERF141	Phvul.009G029600	Chr09:6536069-6538397	PvAP2-ERF132	Phvul.008G172200	Chr08:45116054-45117855	74%	5,00E-113	323
PvAP2-ERF141	Phvul.009G029600	Chr09:6536069-6538397	PvAP2-ERF22	Phvul.001G251200	Chr01:50886895-50888163	70%	8,00E-78	232
PvAP2-ERF142	Phvul.009G074300	Chr09:12286871-12290865	PvAP2-ERF3	Phvul.001G031200	Chr01:2907733-2912245	76%	0	898
PvAP2-ERF142	Phvul.009G074300	Chr09:12286871-12290865	PvAP2-ERF23	Phvul.002G009100	Chr02:1058028-1061555	70%	2,00E-171	495
PvAP2-ERF142	Phvul.009G074300	Chr09:12286871-12290865	PvAP2-ERF60	Phvul.003G232600	Chr03:45498765-45502904	75%	0	620

PvAP2-ERF142	Phvul.009G074300	Chr09:12286871-12290865	PvAP2-ERF167	Phvul.010G130200	Chr10:40027019-40030846	81%	2,00E-141	469
PvAP2-ERF142	Phvul.009G074300	Chr09:12286871-12290865	PvAP2-ERF172	Phvul.011G058000	Chr11:5016893-5020109	71%	4,00E-118	435
PvAP2-ERF146	Phvul.009G109600	Chr09:16431520-16432907	PvAP2-ERF6	Phvul.001G073800	Chr01:10050549-10051844	75%	2,00E-74	215
PvAP2-ERF146	Phvul.009G109600	Chr09:16431520-16432907	PvAP2-ERF79	Phvul.005G170600	Chr05:39420680-39421777	72%	1,00E-65	191
PvAP2-ERF148	Phvul.009G137000	Chr09:20103112-20105454	PvAP2-ERF130	Phvul.008G159400	Chr08:40680584-40682430	75%	1,00E-86	255
PvAP2-ERF148	Phvul.009G137000	Chr09:20103112-20105454	PvAP2-ERF43	Phvul.002G295700	Chr02:45929067-45930538	70%	5,00E-57	178
PvAP2-ERF160	Phvul.010G050600	Chr10:8042080-8042795	PvAP2-ERF125	Phvul.008G046400	Chr08:4004959-4005582	71%	2,00E-52	158
PvAP2-ERF164	Phvul.010G092300	Chr10:34005917-34007381	PvAP2-ERF76	Phvul.005G126300	Chr05:35040940-35041680	74%	2,00E-82	237
PvAP2-ERF164	Phvul.010G092300	Chr10:34005917-34007381	PvAP2-ERF77	Phvul.005G126600	Chr05:35099239-35100039	71%	9,00E-72	209
PvAP2-ERF165	Phvul.010G114900	Chr10:38090570-38091511	PvAP2-ERF86	Phvul.006G114100	Chr06:22942518-22943272	71%	2,00E-64	181
PvAP2-ERF166	Phvul.010G124700	Chr10:39467544-39470639	PvAP2-ERF157	Phvul.009G262200	Chr09:37365318-37367770	80%	5,00E-154	431
PvAP2-ERF167	Phvul.010G130200	Chr10:40027019-40030846	PvAP2-ERF23	Phvul.002G009100	Chr02:1058028-1061555	75%	2,00E-129	384
PvAP2-ERF167	Phvul.010G130200	Chr10:40027019-40030846	PvAP2-ERF51	Phvul.003G144500	Chr03:34150168-34153951	76%	3,00E-124	440
PvAP2-ERF167	Phvul.010G130200	Chr10:40027019-40030846	PvAP2-ERF60	Phvul.003G232600	Chr03:45498765-45502904	81%	1,00E-129	469
PvAP2-ERF167	Phvul.010G130200	Chr10:40027019-40030846	PvAP2-ERF125	Phvul.008G052000	Chr08:4601738-4606123	81%	1,00E-123	461
PvAP2-ERF167	Phvul.010G130200	Chr10:40027019-40030846	PvAP2-ERF172	Phvul.011G058000	Chr11:5016893-5020109	80%	7,00E-125	409
PvAP2-ERF168	Phvul.010G146600	Chr10:41789538-41790170	PvAP2-ERF79	Phvul.005G170600	Chr05:39420680-39421777	76%	5,00E-75	216
PvAP2-ERF168	Phvul.010G146600	Chr10:41789538-41790170	PvAP2-ERF6	Phvul.001G073800	Chr01:10050549-10051844	74%	2,00E-60	211
PvAP2-ERF173	Phvul.011G071100	Chr11:6338128-6342324	PvAP2-ERF20	Phvul.001G174400	Chr01:43723288-43726156	71%	4,00E-113	333
PvAP2-ERF173	Phvul.011G071100	Chr11:6338128-6342324	PvAP2-ERF25	Phvul.002G016900	Chr02:1860815-1864323	76%	8,00E-103	309
PvAP2-ERF173	Phvul.011G071100	Chr11:6338128-6342324	PvAP2-ERF62	Phvul.003G241900	Chr03:46544077-46547812	78%	3,00E-103	329
PvAP2-ERF173	Phvul.011G071100	Chr11:6338128-6342324	PvAP2-ERF78	Phvul.005G138300	Chr05:36714215-36718541	79%	0,00E+00	599
PvAP2-ERF174	Phvul.011G091400	Chr11:9148323-9148991	PvAP2-ERF76	Phvul.005G126300	Chr05:35040940-35041680	76%	2,00E-82	237
PvAP2-ERF174	Phvul.011G091400	Chr11:9148323-9148991	PvAP2-ERF77	Phvul.005G126600	Chr05:35099239-35100039	71%	9,00E-72	209
PvAP2-ERF175	Phvul.011G107800	Chr11:12892620-12894683	PvAP2-ERF75	Phvul.005G111200	Chr05:32566424-32568115	72%	1,00E-79	227
PvAP2-ERF176	Phvul.011G118600	Chr11:19321983-19324035	PvAP2-ERF74	Phvul.005G105200	Chr05:31203406-31204629	80%	3,00E-125	378

Gen ID	Phytozome Identfier	Chromosome: Start - Finish	Gen ID	Phytozome Identfier	Chromosome: Start - Finish	Identity	e-value	Score
PvAP2-ERF19	Phvul.001G160500	Chr01:42037682-42038650	PvAP2-ERF16	Phvul.001G160200	Chr01:41973984-41974619	73%	1,00E-60	180
PvAP2-ERF27	Phvul.002G035900	Chr02:3545434-3545982	PvAP2-ERF28	Phvul.002G036000	Chr02:3561530-3562521	71%	5,00E-58	174
PvAP2-ERF36	Phvul.002G162500	Chr02:30433762-30435007	PvAP2-ERF43	Phvul.002G295700	Chr02:45929067-45930538	70%	5,00E-84	247
PvAP2-ERF48	Phvul.003G069000	Chr03:10190940-10193616	PvAP2-ERF49	Phvul.003G102500	Chr03:25181566-25183062	81%	2,00E-92	347
PvAP2-ERF48	Phvul.003G069000	Chr03:10190940-10193616	PvAP2-ERF52	Phvul.003G144500	Chr03:34150168-34153951	78%	1,00E-81	351
PvAP2-ERF48	Phvul.003G069000	Chr03:10190940-10193616	PvAP2-ERF53	Phvul.003G153100	Chr03:35757558-35760426	82%	4,00E-96	318
PvAP2-ERF84	Phvul.006G106100	Chr06:22259920-22260531	PvAP2-ERF90	Phvul.006G179800	Chr06:28956271-28957252	70%	2,00E-47	159
PvAP2-ERF100	Phvul.007G127800	Chr07:27274067-27275018	PvAP2-ERF119	Phvul.007G273000	Chr07:51127595-51128470	72%	4,00E-66	194
PvAP2-ERF101	Phvul.007G128000	Chr07:27559597-27560279	PvAP2-ERF118	Phvul.007G272900	Chr07:51111180-51111614	72%	1,00E-48	143
PvAP2-ERF107	Phvul.007G193700	Chr07:43194246-43194881	PvAP2-ERF108	Phvul.007G193800	Chr07:43227132-43227770	73%	4,00E-102	285
PvAP2-ERF117	Phvul.007G272800	Chr07:51105207-51105858	PvAP2-ERF102	Phvul.007G128100	Chr07:27720034-27720477	70%	9,00E-46	136
PvAP2-ERF135	Phvul.008G220400	Chr08:53286141-53288298	PvAP2-ERF126	Phvul.008G092800	Chr08:9500856-9502893	72%	2,00E-51	218
PvAP2-ERF153	Phvul.009G225000	Chr09:33327983-33329052	PvAP2-ERF143	Phvul.009G084400	Chr09:13327339-13328429	71%	6,00E-42	150
PvAP2-ERF154	Phvul.009G240800	Chr09:35418233-35418610	PvAP2-ERF155	Phvul.009G240900	Chr09:35430515-35430877	70%	7,00E-52	154

Table S6: miRNA targets identified by psRNATarget

Acacia auriculiformis												
miRNA_Acc.	Target_Acc.	Expectation	UPE	miRNA_start	miRNA_end	Target_start	Target_end	miRNA_aligned_fragment	Target_aligned_fragment	Inhibition	Target_Desc.	Multiplicity
aau-miR172	PvAP2-ERF173	0,5	11,541	1	22	3656	3677	UGAGAAUCUUGAUGAUGCUGCA	UGCAGCAUCAUCAGGAUUCUCA	Cleavage		1
aau-miR172	PvAP2-ERF78	0,5	5,882	1	22	3797	3818	UGAGAAUCUUGAUGAUGCUGCA	UGCAGCAUCAUCAGGAUUCUCA	Cleavage		1
aau-miR172	PvAP2-ERF25	2	16,394	1	22	3064	3085	UGAGAAUCUUGAUGAUGCUGCA	UGCAGCAUCAUCAGGAUUCCCA	Cleavage		1
aau-miR172	PvAP2-ERF62	2	18,825	1	22	3200	3221	UGAGAAUCUUGAUGAUGCUGCA	UGCAGCAUCAUCAGGAUUCCCA	Cleavage		1
aau-miR172	PvAP2-ERF20	3	9,676	1	22	2530	2551	UGAGAAUCUUGAUGAUGCUGCA	UGCAGCAUCAUCAGGAUUCCCU	Cleavage		1
Arachis hypogaea												
miRNA_Acc.	Target_Acc.	Expectation	UPE	miRNA_start	miRNA_end	Target_start	Target_end	miRNA_aligned_fragment	Target_aligned_fragment	Inhibition	Target_Desc.	Multiplicity
ahy-miR3509-5p	PvAP2-ERF142	3	19,028	1	22	2269	2290	AUACUUGAGAGCCGUUAGAUGA	UGAUCUCGCGGCUCUUAAGUAC	Cleavage		1
ahy-miR3509-5p	PvAP2-ERF60	3	18,728	1	22	2310	2331	AUACUUGAGAGCCGUUAGAUGA	UGAUCUGGCCGCACUCAAGUAU	Translation		1
Arabidopsis lyrata												
miRNA_Acc.	Target_Acc.	Expectation	UPE	miRNA_start	miRNA_end	Target_start	Target_end	miRNA_aligned_fragment	Target_aligned_fragment	Inhibition	Target_Desc.	Multiplicity
aly-miR156a-3p	PvERF3	3	23,803	1	21	2744	2764	GCUCACUGCUCUUUCUGUCAG	UUUGCAGAAAGAGUAGUGGGU	Cleavage		1
aly-miR172a-3p	PvAP2-ERF78	0,5	10,114	1	20	3797	3816	AGAAUCUUGAUGAUGCUGCA	UGCAGCAUCAUCAGGAUUCU	Cleavage		1
aly-miR172a-3p	PvAP2-ERF173	0,5	11,33	1	20	3656	3675	AGAAUCUUGAUGAUGCUGCA	UGCAGCAUCAUCAGGAUUCU	Cleavage		1
aly-miR172a-3p	PvAP2-ERF62	1,5	16,463	1	20	3200	3219	AGAAUCUUGAUGAUGCUGCA	UGCAGCAUCAUCAGGAUUCC	Cleavage		1
aly-miR172a-3p	PvAP2-ERF25	1,5	16,15	1	20	3064	3083	AGAAUCUUGAUGAUGCUGCA	UGCAGCAUCAUCAGGAUUCC	Cleavage		1
aly-miR172a-3p	PvAP2-ERF20	1,5	9,626	1	20	2530	2549	AGAAUCUUGAUGAUGCUGCA	UGCAGCAUCAUCAGGAUUCC	Cleavage		1
aly-miR172a-3p	PvAP2-ERF113	2,5	22,52	1	20	3572	3591	AGAAUCUUGAUGAUGCUGCA	AGCAGCAUCAUCAGGAUUCG	Cleavage		1
aly-miR172b-3p	PvAP2-ERF173	0,5	11,33	1	20	3656	3675	AGAAUCUUGAUGAUGCUGCA	UGCAGCAUCAUCAGGAUUCU	Cleavage		1
aly-miR172b-3p	PvAP2-ERF78	0,5	10,114	1	20	3797	3816	AGAAUCUUGAUGAUGCUGCA	UGCAGCAUCAUCAGGAUUCU	Cleavage		1
aly-miR172b-3p	PvAP2-ERF20	1,5	9,626	1	20	2530	2549	AGAAUCUUGAUGAUGCUGCA	UGCAGCAUCAUCAGGAUUCC	Cleavage		1
aly-miR172b-3p	PvAP2-ERF25	1,5	16,15	1	20	3064	3083	AGAAUCUUGAUGAUGCUGCA	UGCAGCAUCAUCAGGAUUCC	Cleavage		1
aly-miR172b-3p	PvAP2-ERF62	1,5	16,463	1	20	3200	3219	AGAAUCUUGAUGAUGCUGCA	UGCAGCAUCAUCAGGAUUCC	Cleavage		1
aly-miR172b-3p	PvAP2-ERF113	2,5	22,52	1	20	3572	3591	AGAAUCUUGAUGAUGCUGCA	AGCAGCAUCAUCAGGAUUCG	Cleavage		1
aly-miR172c-3p	PvAP2-ERF173	0,5	11,33	1	21	3655	3675	AGAAUCUUGAUGAUGCUGCAG	CUGCAGCAUCAUCAGGAUUCU	Cleavage		1
aly-miR172c-3p	PvAP2-ERF78	0,5	10,114	1	21	3796	3816	AGAAUCUUGAUGAUGCUGCAG	CUGCAGCAUCAUCAGGAUUCU	Cleavage		1
aly-miR172c-3p	PvAP2-ERF20	1,5	9,626	1	21	2529	2549	AGAAUCUUGAUGAUGCUGCAG	CUGCAGCAUCAUCAGGAUUCC	Cleavage		1
aly-miR172c-3p	PvAP2-ERF25	1,5	16,15	1	21	3063	3083	AGAAUCUUGAUGAUGCUGCAG	CUGCAGCAUCAUCAGGAUUCC	Cleavage		1
aly-miR172c-3p	PvAP2-ERF62	1,5	16,463	1	21	3199	3219	AGAAUCUUGAUGAUGCUGCAG	CUGCAGCAUCAUCAGGAUUCC	Cleavage		1
aly-miR172c-3p	PvAP2-ERF113	2,5	22,52	1	21	3571	3591	AGAAUCUUGAUGAUGCUGCAG	CAGCAGCAUCAUCAGGAUUCG	Cleavage		1
aly-miR172d-3p	PvAP2-ERF173	0,5	11,33	1	21	3655	3675	AGAAUCUUGAUGAUGCUGCAG	CUGCAGCAUCAUCAGGAUUCU	Cleavage		1
aly-miR172d-3p	PvAP2-ERF78	0,5	10,114	1	21	3796	3816	AGAAUCUUGAUGAUGCUGCAG	CUGCAGCAUCAUCAGGAUUCU	Cleavage		1
aly-miR172d-3p	PvAP2-ERF20	1,5	9,626	1	21	2529	2549	AGAAUCUUGAUGAUGCUGCAG	CUGCAGCAUCAUCAGGAUUCC	Cleavage		1
aly-miR172d-3p	PvAP2-ERF25	1,5	16,15	1	21	3063	3083	AGAAUCUUGAUGAUGCUGCAG	CUGCAGCAUCAUCAGGAUUCC	Cleavage		1
aly-miR172d-3p	PvAP2-ERF62	1,5	16,463	1	21	3199	3219	AGAAUCUUGAUGAUGCUGCAG	CUGCAGCAUCAUCAGGAUUCC	Cleavage		1
aly-miR172d-3p	PvAP2-ERF113	2,5	22,52	1	21	3571	3591	AGAAUCUUGAUGAUGCUGCAG	CAGCAGCAUCAUCAGGAUUCG	Cleavage		1
aly-miR172e-3p	PvAP2-ERF20	1,5	9,823	1	20	2529	2548	GAAUCUUGAUGAUGCUGCAU	CUGCAGCAUCAUCAGGAUUC	Cleavage		1
aly-miR172e-3p	PvAP2-ERF25	1,5	15,486	1	20	3063	3082	GAAUCUUGAUGAUGCUGCAU	CUGCAGCAUCAUCAGGAUUC	Cleavage		1
aly-miR172e-3p	PvAP2-ERF62	1,5	15,901	1	20	3199	3218	GAAUCUUGAUGAUGCUGCAU	CUGCAGCAUCAUCAGGAUUC	Cleavage		1
aly-miR172e-3p	PvAP2-ERF173	1,5	11,994	1	20	3655	3674	GAAUCUUGAUGAUGCUGCAU	CUGCAGCAUCAUCAGGAUUC	Cleavage		1
aly-miR172e-3p	PvAP2-ERF78	1,5	10,7	1	20	3796	3815	GAAUCUUGAUGAUGCUGCAU	CUGCAGCAUCAUCAGGAUUC	Cleavage		1
aly-miR172e-3p	PvAP2-ERF113	2,5	20,798	1	20	3571	3590	GAAUCUUGAUGAUGCUGCAU	CAGCAGCAUCAUCAGGAUUC	Cleavage		1
aly-miR172f-3p	PvAP2-ERF173	0,5	11,33	1	21	3655	3675	AGAAUCUUGAUGAUGCUGCAG	CUGCAGCAUCAUCAGGAUUCU	Cleavage		1
aly-miR172f-3p	PvAP2-ERF78	0,5	10,114	1	21	3796	3816	AGAAUCUUGAUGAUGCUGCAG	CUGCAGCAUCAUCAGGAUUCU	Cleavage		1
aly-miR172f-3p	PvAP2-ERF20	1,5	9,626	1	21	2529	2549	AGAAUCUUGAUGAUGCUGCAG	CUGCAGCAUCAUCAGGAUUCC	Cleavage		1
aly-miR172f-3p	PvAP2-ERF25	1,5	16,15	1	21	3063	3083	AGAAUCUUGAUGAUGCUGCAG	CUGCAGCAUCAUCAGGAUUCC	Cleavage		1
aly-miR172f-3p	PvAP2-ERF62	1,5	16,463	1	21	3199	3219	AGAAUCUUGAUGAUGCUGCAG	CUGCAGCAUCAUCAGGAUUCC	Cleavage		1
aly-miR172f-3p	PvAP2-ERF113	2,5	22,52	1	21	3571	3591	AGAAUCUUGAUGAUGCUGCAG	CAGCAGCAUCAUCAGGAUUCG	Cleavage		1
aly-miR3445-5p.1	PvAP2-ERF26	3	17,388	1	21	439	459	CGAACCAUCACCUUCCAAACC	GGUUUGGAGGGUGAUGAUUCU	Cleavage		1
aly-miR4246	PvAP2-ERF30	2,5	16,965	1	21	1308	1328	AAAUCCAAUUUUCAUUGCUUA	UAAGCAAUGGAACUUGGAUUG	Translation		1

aly-miR831-5p	PvAP2-ERF139	2,5	11,62	1	21	76	96	AGAAGAGGUACAAGGAGAUGA	UCAUCAUCUUAUACCUCUUCU	Translation		1
aly-miR835-3p	PvAP2-ERF175	3	13,467	1	20	1783	1802	UGAAGAAGAUACGCAAGAAA	UUUCUUGCGUGUCUGUUUUA	Cleavage		1
aly-miR837-5p	PvAP2-ERF148	1,5	13,354	1	21	2261	2281	CAUUGUUUCUUGUUUUUUUCA	UGAAAGAAACAAAAAACAAUG	Translation		1
aly-miR838-3p	PvAP2-ERF84	1	12,97	1	21	486	506	UUUUCUUCUUCUUCUUGCACA	UGUGCAGGAAGAAGAAGAAGA	Cleavage		1
aly-miR838-3p	PvAP2-ERF156	2	21,194	1	20	1199	1218	UUUUCUUCUUCUUCUUGCAC	AUGUGAGAAGAAGAAGAAAA	Cleavage		1
aly-miR838-3p	PvAP2-ERF133	2,5	22,104	1	21	507	527	UUUUCUUCUUCUUCUUGCACA	UGUGGGAGAAGAUGAAGAAAA	Translation		1
aly-miR838-3p	PvAP2-ERF42	2,5	22,525	1	21	440	460	UUUUCUUCUUCUUCUUGCACA	UUUGGAAGAAGAAGAAGAAGA	Cleavage		1
aly-miR838-3p	PvAP2-ERF24	2	9,672	1	20	209	228	UUUUCUUCUUCUUCUUGCAC	GUGAAGGAAGAAGAAGAGAA	Cleavage		1
aly-miR838-3p	PvAP2-ERF67	2,5	7,016	1	20	267	286	UUUUCUUCUUCUUCUUGCAC	GUAGAAGAAGAAGAAGAAGA	Cleavage		1
aly-miR838-3p	PvAP2-ERF51	2,5	4,646	1	20	1498	1517	UUUUCUUCUUCUUCUUGCAC	GGGGGAGAAGAAGAAGAAAA	Cleavage		1
aly-miR838-3p	PvAP2-ERF144	3	18,622	1	20	939	958	UUUUCUUCUUCUUCUUGCAC	GAGCAAGAAGUAGAGGAGAA	Translation		1
aly-miR846-5p	PvAP2-ERF106	3	21,02	1	21	372	392	UUCAGGGACUUCAAUUCAGAA	UUUUGAGUUGGAGUUCUUGGA	Cleavage		1
aly-miR851-3p	PvAP2-ERF138	3	14,872	1	22	222	243	UGUGGGUGGCAAACAAAGACGA	UUGUUUUUGUUUGUCAUCUACU	Cleavage		1
Amphimedon queenslandica												
miRNA_Acc.	Target_Acc.	Expectation	UPE	miRNA_start	miRNA_end	Target_start	Target_end	miRNA_aligned_fragment	Target_aligned_fragment	Inhibition	Target_Desc.	Multiplicity
aqc-miR172a	PvAP2-ERF78	0,5	10,114	1	20	3797	3816	AGAAUCUUGAUGAUGCUGCA	UGCAGCAUCAUCAGGAUUCU	Cleavage		1
aqc-miR172a	PvAP2-ERF173	0,5	11,33	1	20	3656	3675	AGAAUCUUGAUGAUGCUGCA	UGCAGCAUCAUCAGGAUUCU	Cleavage		1
aqc-miR172a	PvAP2-ERF62	1,5	16,463	1	20	3200	3219	AGAAUCUUGAUGAUGCUGCA	UGCAGCAUCAUCAGGAUUCC	Cleavage		1
aqc-miR172a	PvAP2-ERF25	1,5	16,15	1	20	3064	3083	AGAAUCUUGAUGAUGCUGCA	UGCAGCAUCAUCAGGAUUCC	Cleavage		1
aqc-miR172a	PvAP2-ERF20	1,5	9,626	1	20	2530	2549	AGAAUCUUGAUGAUGCUGCA	UGCAGCAUCAUCAGGAUUCC	Cleavage		1
aqc-miR172a	PvAP2-ERF113	2,5	22,52	1	20	3572	3591	AGAAUCUUGAUGAUGCUGCA	AGCAGCAUCAUCAGGAUUCG	Cleavage		1
aqc-miR172b	PvAP2-ERF20	0,5	9,626	1	20	2530	2549	GGAAUCUUGAUGAUGCUGCA	UGCAGCAUCAUCAGGAUUCC	Cleavage		1
aqc-miR172b	PvAP2-ERF25	0,5	16,15	1	20	3064	3083	GGAAUCUUGAUGAUGCUGCA	UGCAGCAUCAUCAGGAUUCC	Cleavage		1
aqc-miR172b	PvAP2-ERF62	0,5	16,463	1	20	3200	3219	GGAAUCUUGAUGAUGCUGCA	UGCAGCAUCAUCAGGAUUCC	Cleavage		1
aqc-miR172b	PvAP2-ERF173	1	11,33	1	20	3656	3675	GGAAUCUUGAUGAUGCUGCA	UGCAGCAUCAUCAGGAUUCU	Cleavage		1
aqc-miR172b	PvAP2-ERF78	1	10,114	1	20	3797	3816	GGAAUCUUGAUGAUGCUGCA	UGCAGCAUCAUCAGGAUUCU	Cleavage		1
aqc-miR172b	PvAP2-ERF113	2,5	22,52	1	20	3572	3591	GGAAUCUUGAUGAUGCUGCA	AGCAGCAUCAUCAGGAUUCG	Cleavage		1
aqc-miR395a	PvAP2-ERF179	3	11,026	1	20	4356	4375	CUGAAGGGUUUGGAGGAACU	AGUUCCUCCUAACUCCUCAG	Translation		1
aqc-miR395b	PvAP2-ERF179	3	11,026	1	20	4356	4375	CUGAAGGGUUUGGAGGAACU	AGUUCCUCCUAACUCCUCAG	Translation		1
aqc-miR477d	PvAP2-ERF133	3	14,547	1	20	1126	1145	CUCUUCUUCAAAGGCUUCUA	UAAAAGACUAUGAAGAAGAG	Translation		1
aqc-miR529	PvAP2-ERF69	3	23,061	1	20	688	707	AGAAGAGAGAGAGCACAACC	GUUUGUCUUCUUUCUCUUCU	Cleavage		1
Aegilops tauschii												
miRNA_Acc.	Target_Acc.	Expectation	UPE	miRNA_start	miRNA_end	Target_start	Target_end	miRNA_aligned_fragment	Target_aligned_fragment	Inhibition	Target_Desc.	Multiplicity
ata-miR172	PvAP2-ERF173	0,5	11,541	1	22	3656	3677	UGAGAAUCUUGAUGAUGCUGCA	UGCAGCAUCAUCAGGAUUCUCA	Cleavage		1
ata-miR172	PvAP2-ERF78	0,5	5,882	1	22	3797	3818	UGAGAAUCUUGAUGAUGCUGCA	UGCAGCAUCAUCAGGAUUCUCA	Cleavage		1
ata-miR172	PvAP2-ERF25	2	16,394	1	22	3064	3085	UGAGAAUCUUGAUGAUGCUGCA	UGCAGCAUCAUCAGGAUUCCCA	Cleavage		1
ata-miR172	PvAP2-ERF62	2	18,825	1	22	3200	3221	UGAGAAUCUUGAUGAUGCUGCA	UGCAGCAUCAUCAGGAUUCCCA	Cleavage		1
ata-miR172	PvAP2-ERF20	3	9,676	1	22	2530	2551	UGAGAAUCUUGAUGAUGCUGCA	UGCAGCAUCAUCAGGAUUCCCU	Cleavage		1
Arabidopsis thaliana												
miRNA_Acc.	Target_Acc.	Expectation	UPE	miRNA_start	miRNA_end	Target_start	Target_end	miRNA_aligned_fragment	Target_aligned_fragment	Inhibition	Target_Desc.	Multiplicity
ath-miR172a	PvAP2-ERF173	0,5	11,33	1	20	3656	3675	AGAAUCUUGAUGAUGCUGCA	UGCAGCAUCAUCAGGAUUCU	Cleavage		1
ath-miR172a	PvAP2-ERF78	0,5	10,114	1	20	3797	3816	AGAAUCUUGAUGAUGCUGCA	UGCAGCAUCAUCAGGAUUCU	Cleavage		1
ath-miR172a	PvAP2-ERF20	1,5	9,626	1	20	2530	2549	AGAAUCUUGAUGAUGCUGCA	UGCAGCAUCAUCAGGAUUCC	Cleavage		1
ath-miR172a	PvAP2-ERF25	1,5	16,15	1	20	3064	3083	AGAAUCUUGAUGAUGCUGCA	UGCAGCAUCAUCAGGAUUCC	Cleavage		1
ath-miR172a	PvAP2-ERF62	1,5	16,463	1	20	3200	3219	AGAAUCUUGAUGAUGCUGCA	UGCAGCAUCAUCAGGAUUCC	Cleavage		1
ath-miR172a	PvAP2-ERF113	2,5	22,52	1	20	3572	3591	AGAAUCUUGAUGAUGCUGCA	AGCAGCAUCAUCAGGAUUCG	Cleavage		1
ath-miR172b-3p	PvAP2-ERF173	0,5	11,33	1	20	3656	3675	AGAAUCUUGAUGAUGCUGCA	UGCAGCAUCAUCAGGAUUCU	Cleavage		1
ath-miR172b-3p	PvAP2-ERF78	0,5	10,114	1	20	3797	3816	AGAAUCUUGAUGAUGCUGCA	UGCAGCAUCAUCAGGAUUCU	Cleavage		1
ath-miR172b-3p	PvAP2-ERF20	1,5	9,626	1	20	2530	2549	AGAAUCUUGAUGAUGCUGCA	UGCAGCAUCAUCAGGAUUCC	Cleavage		1
ath-miR172b-3p	PvAP2-ERF25	1,5	16,15	1	20	3064	3083	AGAAUCUUGAUGAUGCUGCA	UGCAGCAUCAUCAGGAUUCC	Cleavage		1
ath-miR172b-3p	PvAP2-ERF62	1,5	16,463	1	20	3200	3219	AGAAUCUUGAUGAUGCUGCA	UGCAGCAUCAUCAGGAUUCC	Cleavage		1
ath-miR172b-3p	PvAP2-ERF113	2,5	22,52	1	20	3572	3591	AGAAUCUUGAUGAUGCUGCA	AGCAGCAUCAUCAGGAUUCG	Cleavage		1
ath-miR172c	PvAP2-ERF173	0,5	11,33	1	21	3655	3675	AGAAUCUUGAUGAUGCUGCAG	CUGCAGCAUCAUCAGGAUUCU	Cleavage		1
ath-miR172c	PvAP2-ERF78	0,5	10,114	1	21	3796	3816	AGAAUCUUGAUGAUGCUGCAG	CUGCAGCAUCAUCAGGAUUCU	Cleavage		1
ath-miR172c	PvAP2-ERF20	1,5	9,626	1	21	2529	2549	AGAAUCUUGAUGAUGCUGCAG	CUGCAGCAUCAUCAGGAUUCC	Cleavage		1

ath-miR172c	PvAP2-ERF25	1,5	16,15	1	21	3063	3083	AGAAUCUUGAUGAUGCUGCAG	CUGCAGCAUCAUCAGGAUUCC	Cleavage	1
ath-miR172c	PvAP2-ERF62	1,5	16,463	1	21	3199	3219	AGAAUCUUGAUGAUGCUGCAG	CUGCAGCAUCAUCAGGAUUCC	Cleavage	1
ath-miR172c	PvAP2-ERF113	2,5	22,52	1	21	3571	3591	AGAAUCUUGAUGAUGCUGCAG	CAGCAGCAUCAUCAGGAUUCG	Cleavage	1
ath-miR172d	PvAP2-ERF78	0,5	10,114	1	21	3796	3816	AGAAUCUUGAUGAUGCUGCAG	CUGCAGCAUCAUCAGGAUUCU	Cleavage	1
ath-miR172d	PvAP2-ERF173	0,5	11,33	1	21	3655	3675	AGAAUCUUGAUGAUGCUGCAG	CUGCAGCAUCAUCAGGAUUCU	Cleavage	1
ath-miR172d	PvAP2-ERF62	1,5	16,463	1	21	3199	3219	AGAAUCUUGAUGAUGCUGCAG	CUGCAGCAUCAUCAGGAUUCC	Cleavage	1
ath-miR172d	PvAP2-ERF25	1,5	16,15	1	21	3063	3083	AGAAUCUUGAUGAUGCUGCAG	CUGCAGCAUCAUCAGGAUUCC	Cleavage	1
ath-miR172d	PvAP2-ERF20	1,5	9,626	1	21	2529	2549	AGAAUCUUGAUGAUGCUGCAG	CUGCAGCAUCAUCAGGAUUCC	Cleavage	1
ath-miR172d	PvAP2-ERF113	2,5	22,52	1	21	3571	3591	AGAAUCUUGAUGAUGCUGCAG	CAGCAGCAUCAUCAGGAUUCG	Cleavage	1
ath-miR172e	PvAP2-ERF20	0,5	9,626	1	20	2530	2549	GGAAUCUUGAUGAUGCUGCA	UGCAGCAUCAUCAGGAUUCC	Cleavage	1
ath-miR172e	PvAP2-ERF25	0,5	16,15	1	20	3064	3083	GGAAUCUUGAUGAUGCUGCA	UGCAGCAUCAUCAGGAUUCC	Cleavage	1
ath-miR172e	PvAP2-ERF62	0,5	16,463	1	20	3200	3219	GGAAUCUUGAUGAUGCUGCA	UGCAGCAUCAUCAGGAUUCC	Cleavage	1
ath-miR172e	PvAP2-ERF173	1	11,33	1	20	3656	3675	GGAAUCUUGAUGAUGCUGCA	UGCAGCAUCAUCAGGAUUCU	Cleavage	1
ath-miR172e	PvAP2-ERF78	1	10,114	1	20	3797	3816	GGAAUCUUGAUGAUGCUGCA	UGCAGCAUCAUCAGGAUUCU	Cleavage	1
ath-miR172e	PvAP2-ERF113	2,5	22,52	1	20	3572	3591	GGAAUCUUGAUGAUGCUGCA	AGCAGCAUCAUCAGGAUUCG	Cleavage	1
ath-miR407	PvAP2-ERF12	3	15,715	1	21	3528	3548	UUUAAAUCAUAUACUUUUGGU	GCUCAAAGUGUAUGGUUUGAA	Cleavage	1
ath-miR414	PvAP2-ERF166	2,5	23,018	1	20	330	349	UCAUCUUCAUCAUCAUCGUC	GAUGAUGAUGAUGAGGACGA	Cleavage	1
ath-miR414	PvAP2-ERF176	2,5	17,029	1	20	860	879	UCAUCUUCAUCAUCAUCGUC	GAGGAUGAUGAUGAUGAUGA	Cleavage	1
ath-miR414	PvAP2-ERF180	3	8,848	1	21	845	865	UCAUCUUCAUCAUCAUCGUCA	UGAUGGUGAAGAAGAAGAUGA	Translation	1
ath-miR415	PvAP2-ERF151	2,5	8,646	1	20	1014	1033	AACAGAGCAGAAACAGAACA	UCUUCUGUUUCUACUUUGUU	Cleavage	1
ath-miR5016	PvAP2-ERF17	3	16,845	1	20	137	156	UUCUUGUGGAUUCCUUGGAA	ACUCAAGGGAUCCACAAGAA	Cleavage	1
ath-miR5021	PvAP2-ERF172	1	11,663	1	20	1936	1955	UGAGAAGAAGAAGAAGAAAA	CUUUCUUCUUCUUCUUCUCA	Cleavage	1
ath-miR5021	PvAP2-ERF155	2,5	19,919	1	20	250	269	UGAGAAGAAGAAGAAGAAAA	UCUUCUUCUUCUUCUUCUUC	Cleavage	1
ath-miR5021	PvAP2-ERF117	2,5	13,45	1	20	377	396	UGAGAAGAAGAAGAAGAAAA	UCUUCUUCUUCUUCUUCUUC	Cleavage	1
ath-miR5021	PvAP2-ERF43	2	0,284	1	20	313	332	UGAGAAGAAGAAGAAGAAAA	UCUUCUUCUUCUUCUUCUCU	Cleavage	2
ath-miR5021	PvAP2-ERF43	3	14,086	1	20	1426	1445	UGAGAAGAAGAAGAAGAAAA	UUUUCUUAUUUUCCUUUUCA	Cleavage	2
ath-miR5021	PvAP2-ERF18	2,5	5,525	1	20	360	379	UGAGAAGAAGAAGAAGAAAA	UCUUCUUCUUCUUCUUCUUU	Cleavage	1
ath-miR5021	PvAP2-ERF101	3	14,685	1	20	427	446	UGAGAAGAAGAAGAAGAAAA	CCUUCUUCUUCCUCUUCUCA	Translation	1
ath-miR5021	PvAP2-ERF105	2,5	13,485	1	20	848	867	UGAGAAGAAGAAGAAGAAAA	UCUUCUUCUUCUUCUUCUUC	Cleavage	1
ath-miR5021	PvAP2-ERF53	2	11,66	1	20	333	352	UGAGAAGAAGAAGAAGAAAA	UAUUCUUCUUCUUCUUCUCU	Cleavage	1
ath-miR5021	PvAP2-ERF151	2,5	6,83	1	20	978	997	UGAGAAGAAGAAGAAGAAAA	UUCUUUUCUUCUUCUUCUUG	Cleavage	1
ath-miR5021	PvAP2-ERF25	3	20,135	1	20	365	384	UGAGAAGAAGAAGAAGAAAA	UUUUUUUUUUUUUUUUCUCU	Cleavage	1
ath-miR5021	PvAP2-ERF47	3	13,438	1	20	505	524	UGAGAAGAAGAAGAAGAAAA	CUUUCUUCUGCUCCUUCUCA	Translation	1
ath-miR5021	PvAP2-ERF125	3	8,996	1	20	1144	1163	UGAGAAGAAGAAGAAGAAAA	UUUUUUUUUUUUUUUUCUCU	Cleavage	1
ath-miR5021	PvAP2-ERF179	3	6,981	1	20	1566	1585	UGAGAAGAAGAAGAAGAAAA	UUUUUUUCUUUUUUUUUUCU	Cleavage	1
ath-miR5021	PvAP2-ERF22	3	19,08	1	20	862	881	UGAGAAGAAGAAGAAGAAAA	UCUUCUUCAUCUUCUUCUUG	Cleavage	1
ath-miR5021	PvAP2-ERF138	3	13,948	1	20	55	74	UGAGAAGAAGAAGAAGAAAA	UCUUCUUUUUUUUUUUUUCA	Cleavage	1
ath-miR5021	PvAP2-ERF32	3	7,17	1	20	320	339	UGAGAAGAAGAAGAAGAAAA	CAUUCUUCUGCUUCUUCUCA	Translation	1
ath-miR5021	PvAP2-ERF120	3	1,316	1	20	4426	4445	UGAGAAGAAGAAGAAGAAAA	UUUUCUUCAACCUCUUCUCA	Translation	1
ath-miR5655	PvAP2-ERF151	3	6,923	1	20	1010	1029	AAGUAGACACAUAAGAAGGA	GCCUUCUUCUGUUUCUACUU	Cleavage	1
ath-miR5658	PvAP2-ERF136	0,5	15,234	1	20	557	576	AUGAUGAUGAUGAUGAUGAA	UUCAUCAUCAUCAUCAUCGU	Cleavage	1
ath-miR5658	PvAP2-ERF163	1	1,942	1	20	260	279	AUGAUGAUGAUGAUGAUGAA	AUCAUCAUCAUCAUCAUCAU	Cleavage	1
ath-miR5658	PvAP2-ERF129	2	4,424	1	21	225	245	AUGAUGAUGAUGAUGAUGAAA	UCUCGUCAUCAUCGUCAUCAU	Cleavage	1
ath-miR5658	PvAP2-ERF6	2	8,284	1	20	823	842	AUGAUGAUGAUGAUGAUGAA	GUCAUUAUCAUCAUCAUCGU	Cleavage	1
ath-miR5658	PvAP2-ERF56	2,5	13,323	1	20	6	25	AUGAUGAUGAUGAUGAUGAA	UUCAUCGUCAUCUUCAUCAA	Cleavage	1
ath-miR5658	PvAP2-ERF169	2	23,281	1	20	1283	1302	AUGAUGAUGAUGAUGAUGAA	UUGAUCAUCAUGAUCAUCAU	Translation	1
ath-miR5658	PvERF3	3	9,888	1	20	633	652	AUGAUGAUGAUGAUGAUGAA	CUCAUCACCAUCAUCAUCAC	Cleavage	1
ath-miR5658	PvAP2-ERF72	3	9,608	1	20	351	370	AUGAUGAUGAUGAUGAUGAA	UUCACCAUCAUCCUCGUCGU	Cleavage	1
ath-miR773b-3p	PvAP2-ERF25	3	11,803	1	20	1403	1422	UUUGAUUCCAGCUUUUGUCU	AUAUAAAAGUUGGAAUCAAU	Cleavage	1
ath-miR837-5p	PvAP2-ERF85	3	17,282	1	21	317	337	AUCAGUUUCUUGUUCGUUUCA	UGAAGCGAACAAGAGUCUGGU	Cleavage	1
ath-miR838	PvAP2-ERF84	2	12,97	1	21	486	506	UUUUCUUCUACUUCUUGCACA	UGUGCAGGAAGAAGAAGAAGA	Translation	1
ath-miR838	PvAP2-ERF144	2	18,622	1	20	939	958	UUUUCUUCUACUUCUUGCAC	GAGCAAGAAGUAGAGGAGAA	Cleavage	1
ath-miR838	PvAP2-ERF150	3	11,983	1	21	1411	1431	UUUUCUUCUACUUCUUGCACA	UGUGAAGGGAGUGGAAGAAGA	Cleavage	1
ath-miR838	PvAP2-ERF156	3	21,194	1	20	1199	1218	UUUUCUUCUACUUCUUGCAC	AUGUGAGAAGAAGAAGAAAA	Translation	1
ath-miR838	PvAP2-ERF24	3	9,672	1	20	209	228	UUUUCUUCUACUUCUUGCAC	GUGAAGGAAGAAGAAGAGAA	Translation	1

ath-miR851-3p	PvAP2-ERF138	2,5	14,77	1	20	222	241	UGGGUGGCAAACAAAGACGA	UUGUUUUUGUUUGUCAUCUA	Cleavage		1
ath-miR854a	PvAP2-ERF6	3	16,067	1	20	863	882	GAUGAGGAUAGGGAGGAGGA	UCCUCGUCUUUAUCCUCGUU	Cleavage		1
ath-miR854a	PvAP2-ERF172	3	19,957	1	20	2795	2814	GAUGAGGAUAGGGAGGAGGA	UCCUCUUCUUCAUCUUCAUC	Translation		1
ath-miR854b	PvAP2-ERF6	3	16,067	1	20	863	882	GAUGAGGAUAGGGAGGAGGA	UCCUCGUCUUUAUCCUCGUU	Cleavage		1
ath-miR854b	PvAP2-ERF172	3	19,957	1	20	2795	2814	GAUGAGGAUAGGGAGGAGGA	UCCUCUUCUUCAUCUUCAUC	Translation		1
ath-miR854c	PvAP2-ERF6	3	16,067	1	20	863	882	GAUGAGGAUAGGGAGGAGGA	UCCUCGUCUUUAUCCUCGUU	Cleavage		1
ath-miR854c	PvAP2-ERF172	3	19,957	1	20	2795	2814	GAUGAGGAUAGGGAGGAGGA	UCCUCUUCUUCAUCUUCAUC	Translation		1
ath-miR854d	PvAP2-ERF6	3	16,067	1	20	863	882	GAUGAGGAUAGGGAGGAGGA	UCCUCGUCUUUAUCCUCGUU	Cleavage		1
ath-miR854d	PvAP2-ERF172	3	19,957	1	20	2795	2814	GAUGAGGAUAGGGAGGAGGA	UCCUCUUCUUCAUCUUCAUC	Translation		1
ath-miR854e	PvAP2-ERF6	3	16,067	1	20	863	882	GAUGAGGAUAGGGAGGAGGA	UCCUCGUCUUUAUCCUCGUU	Cleavage		1
ath-miR854e	PvAP2-ERF172	3	19,957	1	20	2795	2814	GAUGAGGAUAGGGAGGAGGA	UCCUCUUCUUCAUCUUCAUC	Translation		1
ath-miR855	PvAP2-ERF32	3	18,894	1	20	2219	2238	AGCAAAAGCUAAGGAAAAGG	CUUUAUCUUUUGCUUUUGCU	Translation		1
ath-miR859	PvAP2-ERF128	2,5	20,487	1	20	691	710	UCUCUCUGUUGUGAAGUCAA	UUGACUUCACCCUAGAGAGA	Translation		1
ath-miR863-3p	PvAP2-ERF10	3	19,124	1	20	1071	1091	UUGAGAGCAACAAGA-CAUAA	UUAUGAUCUUGCUGCUCUCAA	Translation		1
ath-miR866-5p	PvAP2-ERF120	3	15,716	1	20	1550	1569	UCAAGGAACGGAUUUUGUUA	UAACAAAACUCUUUCUUUGA	Translation		1

Brachypodium distachyon

miRNA_Acc.	Target_Acc.	Expectation	UPE	miRNA_start	miRNA_end	Target_start	Target_end	miRNA_aligned_fragment	Target_aligned_fragment	Inhibition	Target_Desc.	Multiplicity
bdi-miR172a-3p	PvAP2-ERF173	0,5	11,33	1	20	3656	3675	AGAAUCUUGAUGAUGCUGCA	UGCAGCAUCAUCAGGAUUCU	Cleavage		1
bdi-miR172a-3p	PvAP2-ERF78	0,5	10,114	1	20	3797	3816	AGAAUCUUGAUGAUGCUGCA	UGCAGCAUCAUCAGGAUUCU	Cleavage		1
bdi-miR172a-3p	PvAP2-ERF20	1,5	9,626	1	20	2530	2549	AGAAUCUUGAUGAUGCUGCA	UGCAGCAUCAUCAGGAUUCC	Cleavage		1
bdi-miR172a-3p	PvAP2-ERF25	1,5	16,15	1	20	3064	3083	AGAAUCUUGAUGAUGCUGCA	UGCAGCAUCAUCAGGAUUCC	Cleavage		1
bdi-miR172a-3p	PvAP2-ERF62	1,5	16,463	1	20	3200	3219	AGAAUCUUGAUGAUGCUGCA	UGCAGCAUCAUCAGGAUUCC	Cleavage		1
bdi-miR172a-3p	PvAP2-ERF113	2,5	22,52	1	20	3572	3591	AGAAUCUUGAUGAUGCUGCA	AGCAGCAUCAUCAGGAUUCG	Cleavage		1
bdi-miR172b	PvAP2-ERF20	0,5	9,626	1	20	2530	2549	GGAAUCUUGAUGAUGCUGCA	UGCAGCAUCAUCAGGAUUCC	Cleavage		1
bdi-miR172b	PvAP2-ERF25	0,5	16,15	1	20	3064	3083	GGAAUCUUGAUGAUGCUGCA	UGCAGCAUCAUCAGGAUUCC	Cleavage		1
bdi-miR172b	PvAP2-ERF62	0,5	16,463	1	20	3200	3219	GGAAUCUUGAUGAUGCUGCA	UGCAGCAUCAUCAGGAUUCC	Cleavage		1
bdi-miR172b	PvAP2-ERF173	1	11,33	1	20	3656	3675	GGAAUCUUGAUGAUGCUGCA	UGCAGCAUCAUCAGGAUUCU	Cleavage		1
bdi-miR172b	PvAP2-ERF78	1	10,114	1	20	3797	3816	GGAAUCUUGAUGAUGCUGCA	UGCAGCAUCAUCAGGAUUCU	Cleavage		1
bdi-miR172b	PvAP2-ERF113	2,5	22,52	1	20	3572	3591	GGAAUCUUGAUGAUGCUGCA	AGCAGCAUCAUCAGGAUUCG	Cleavage		1
bdi-miR172d	PvAP2-ERF173	0	11,33	1	21	3655	3675	AGAAUCCUGAUGAUGCUGCAG	CUGCAGCAUCAUCAGGAUUCU	Cleavage		1
bdi-miR172d	PvAP2-ERF78	0	10,114	1	21	3796	3816	AGAAUCCUGAUGAUGCUGCAG	CUGCAGCAUCAUCAGGAUUCU	Cleavage		1
bdi-miR172d	PvAP2-ERF20	1	9,626	1	21	2529	2549	AGAAUCCUGAUGAUGCUGCAG	CUGCAGCAUCAUCAGGAUUCC	Cleavage		1
bdi-miR172d	PvAP2-ERF25	1	16,15	1	21	3063	3083	AGAAUCCUGAUGAUGCUGCAG	CUGCAGCAUCAUCAGGAUUCC	Cleavage		1
bdi-miR172d	PvAP2-ERF62	1	16,463	1	21	3199	3219	AGAAUCCUGAUGAUGCUGCAG	CUGCAGCAUCAUCAGGAUUCC	Cleavage		1
bdi-miR172d	PvAP2-ERF113	2	22,52	1	21	3571	3591	AGAAUCCUGAUGAUGCUGCAG	CAGCAGCAUCAUCAGGAUUCG	Cleavage		1
bdi-miR395j-5p	PvAP2-ERF78	3	16,571	1	20	867	886	GUUUCCCGCAAGCACUUCAC	GUGUAGGGAUUGCGGGAAAC	Cleavage		1
bdi-miR395j-5p	PvAP2-ERF173	3	13,786	1	20	1187	1206	GUUUCCCGCAAGCACUUCAC	GUGUAGGGAUUGCGGGAAAC	Cleavage		1
bdi-miR5163b-3p	PvAP2-ERF126	3	10,695	1	20	1483	1502	UAGAUAUUUCAGGUUGUGUG	CACCCAAACUGAGGUAUCUA	Cleavage		1
bdi-miR5175a	PvAP2-ERF105	2,5	7,849	1	20	163	182	AAGAAUUUAGGAACGGAGGG	CACUCCCUUUCUAAAUUCUU	Cleavage		1
bdi-miR7717b-5p	PvAP2-ERF148	3	20,454	1	24	1824	1847	UCUAAGACGACUGAGAAAUAACUA	UAUUUAUUAUUCAGUUGUUUUAGG	Cleavage		1
bdi-miR7721-3p	PvAP2-ERF172	2,5	14,795	1	20	2358	2377	AAAGUUUGGCAUAGAAUUCA	UGAAUGCUGUGACAAACUUU	Translation		1
bdi-miR7725a-3p	PvAP2-ERF138	3	15,859	1	20	2068	2087	UGCAAACAAUGUGAUUUCGU	AUCAAAUCACAUUGGUUGCA	Cleavage		1
bdi-miR7747-5p	PvAP2-ERF129	3	18,292	1	22	1073	1094	AUCAGAUUGUUUUGUUGUAGGA	UACUGCAAUAGUGCAAUCUGAU	Translation		1
bdi-miR7757-3p.2	PvAP2-ERF128	2	14,57	1	20	2578	2597	AGAUAACUUGAUAUGUAAGU	UCUUACAUAUUAGGUUAUCU	Cleavage		1
bdi-miR7776-3p.1	PvAP2-ERF44	3	2,469	1	20	21	40	AAAGAUAUCAGAGGGCAACG	CGUUUCUCUUUCAUAUCUUU	Translation		1
bdi-miR7781-3p	PvAP2-ERF63	3	15,164	1	20	1083	1102	CAUGUCUGUAGUCAGAAAAA	UUUUUUUCACUGCAGACAUC	Cleavage		1

Brassica napus

miRNA_Acc.	Target_Acc.	Expectation	UPE	miRNA_start	miRNA_end	Target_start	Target_end	miRNA_aligned_fragment	Target_aligned_fragment	Inhibition	Target_Desc.	Multiplicity
bna-miR172a	PvAP2-ERF173	0,5	11,33	1	20	3656	3675	AGAAUCUUGAUGAUGCUGCA	UGCAGCAUCAUCAGGAUUCU	Cleavage		1
bna-miR172a	PvAP2-ERF78	0,5	10,114	1	20	3797	3816	AGAAUCUUGAUGAUGCUGCA	UGCAGCAUCAUCAGGAUUCU	Cleavage		1
bna-miR172a	PvAP2-ERF20	1,5	9,626	1	20	2530	2549	AGAAUCUUGAUGAUGCUGCA	UGCAGCAUCAUCAGGAUUCC	Cleavage		1
bna-miR172a	PvAP2-ERF25	1,5	16,15	1	20	3064	3083	AGAAUCUUGAUGAUGCUGCA	UGCAGCAUCAUCAGGAUUCC	Cleavage		1
bna-miR172a	PvAP2-ERF62	1,5	16,463	1	20	3200	3219	AGAAUCUUGAUGAUGCUGCA	UGCAGCAUCAUCAGGAUUCC	Cleavage		1
bna-miR172a	PvAP2-ERF113	2,5	22,52	1	20	3572	3591	AGAAUCUUGAUGAUGCUGCA	AGCAGCAUCAUCAGGAUUCG	Cleavage		1
bna-miR172b	PvAP2-ERF20	0,5	9,626	1	20	2530	2549	GGAAUCUUGAUGAUGCUGCA	UGCAGCAUCAUCAGGAUUCC	Cleavage		1

bna-miR172b	PvAP2-ERF25	0,5	16,15	1	20	3064	3083	GGAAUCUUGAUGAUGCUGCA	UGCAGCAUCAUCAGGAUUCC	Cleavage		1
bna-miR172b	PvAP2-ERF62	0,5	16,463	1	20	3200	3219	GGAAUCUUGAUGAUGCUGCA	UGCAGCAUCAUCAGGAUUCC	Cleavage		1
bna-miR172b	PvAP2-ERF173	1	11,33	1	20	3656	3675	GGAAUCUUGAUGAUGCUGCA	UGCAGCAUCAUCAGGAUUCU	Cleavage		1
bna-miR172b	PvAP2-ERF78	1	10,114	1	20	3797	3816	GGAAUCUUGAUGAUGCUGCA	UGCAGCAUCAUCAGGAUUCU	Cleavage		1
bna-miR172b	PvAP2-ERF113	2,5	22,52	1	20	3572	3591	GGAAUCUUGAUGAUGCUGCA	AGCAGCAUCAUCAGGAUUCG	Cleavage		1
bna-miR172c	PvAP2-ERF20	0,5	9,626	1	20	2530	2549	GGAAUCUUGAUGAUGCUGCA	UGCAGCAUCAUCAGGAUUCC	Cleavage		1
bna-miR172c	PvAP2-ERF25	0,5	16,15	1	20	3064	3083	GGAAUCUUGAUGAUGCUGCA	UGCAGCAUCAUCAGGAUUCC	Cleavage		1
bna-miR172c	PvAP2-ERF62	0,5	16,463	1	20	3200	3219	GGAAUCUUGAUGAUGCUGCA	UGCAGCAUCAUCAGGAUUCC	Cleavage		1
bna-miR172c	PvAP2-ERF173	1	11,33	1	20	3656	3675	GGAAUCUUGAUGAUGCUGCA	UGCAGCAUCAUCAGGAUUCU	Cleavage		1
bna-miR172c	PvAP2-ERF78	1	10,114	1	20	3797	3816	GGAAUCUUGAUGAUGCUGCA	UGCAGCAUCAUCAGGAUUCU	Cleavage		1
bna-miR172c	PvAP2-ERF113	2,5	22,52	1	20	3572	3591	GGAAUCUUGAUGAUGCUGCA	AGCAGCAUCAUCAGGAUUCG	Cleavage		1
bna-miR172d	PvAP2-ERF173	0,5	11,33	1	21	3655	3675	AGAAUCUUGAUGAUGCUGCAG	CUGCAGCAUCAUCAGGAUUCU	Cleavage		1
bna-miR172d	PvAP2-ERF78	0,5	10,114	1	21	3796	3816	AGAAUCUUGAUGAUGCUGCAG	CUGCAGCAUCAUCAGGAUUCU	Cleavage		1
bna-miR172d	PvAP2-ERF20	1,5	9,626	1	21	2529	2549	AGAAUCUUGAUGAUGCUGCAG	CUGCAGCAUCAUCAGGAUUCC	Cleavage		1
bna-miR172d	PvAP2-ERF25	1,5	16,15	1	21	3063	3083	AGAAUCUUGAUGAUGCUGCAG	CUGCAGCAUCAUCAGGAUUCC	Cleavage		1
bna-miR172d	PvAP2-ERF62	1,5	16,463	1	21	3199	3219	AGAAUCUUGAUGAUGCUGCAG	CUGCAGCAUCAUCAGGAUUCC	Cleavage		1
bna-miR172d	PvAP2-ERF113	2,5	22,52	1	21	3571	3591	AGAAUCUUGAUGAUGCUGCAG	CAGCAGCAUCAUCAGGAUUCG	Cleavage		1
Brassica oleracea												
miRNA_Acc.	Target_Acc.	Expectation	UPE	miRNA_start	miRNA_end	Target_start	Target_end	miRNA_aligned_fragment	Target_aligned_fragment	Inhibition	Target_Desc.	Multiplicity
bol-miR172a	PvAP2-ERF173	0,5	11,33	1	20	3656	3675	AGAAUCUUGAUGAUGCUGCA	UGCAGCAUCAUCAGGAUUCU	Cleavage		1
bol-miR172a	PvAP2-ERF78	0,5	10,114	1	20	3797	3816	AGAAUCUUGAUGAUGCUGCA	UGCAGCAUCAUCAGGAUUCU	Cleavage		1
bol-miR172a	PvAP2-ERF20	1,5	9,626	1	20	2530	2549	AGAAUCUUGAUGAUGCUGCA	UGCAGCAUCAUCAGGAUUCC	Cleavage		1
bol-miR172a	PvAP2-ERF25	1,5	16,15	1	20	3064	3083	AGAAUCUUGAUGAUGCUGCA	UGCAGCAUCAUCAGGAUUCC	Cleavage		1
bol-miR172a	PvAP2-ERF62	1,5	16,463	1	20	3200	3219	AGAAUCUUGAUGAUGCUGCA	UGCAGCAUCAUCAGGAUUCC	Cleavage		1
bol-miR172a	PvAP2-ERF113	2,5	22,52	1	20	3572	3591	AGAAUCUUGAUGAUGCUGCA	AGCAGCAUCAUCAGGAUUCG	Cleavage		1
bol-miR172b	PvAP2-ERF173	0,5	11,33	1	20	3656	3675	AGAAUCUUGAUGAUGCUGCA	UGCAGCAUCAUCAGGAUUCU	Cleavage		1
bol-miR172b	PvAP2-ERF78	0,5	10,114	1	20	3797	3816	AGAAUCUUGAUGAUGCUGCA	UGCAGCAUCAUCAGGAUUCU	Cleavage		1
bol-miR172b	PvAP2-ERF20	1,5	9,626	1	20	2530	2549	AGAAUCUUGAUGAUGCUGCA	UGCAGCAUCAUCAGGAUUCC	Cleavage		1
bol-miR172b	PvAP2-ERF25	1,5	16,15	1	20	3064	3083	AGAAUCUUGAUGAUGCUGCA	UGCAGCAUCAUCAGGAUUCC	Cleavage		1
bol-miR172b	PvAP2-ERF62	1,5	16,463	1	20	3200	3219	AGAAUCUUGAUGAUGCUGCA	UGCAGCAUCAUCAGGAUUCC	Cleavage		1
bol-miR172b	PvAP2-ERF113	2,5	22,52	1	20	3572	3591	AGAAUCUUGAUGAUGCUGCA	AGCAGCAUCAUCAGGAUUCG	Cleavage		1
Brassica rapa												
miRNA_Acc.	Target_Acc.	Expectation	UPE	miRNA_start	miRNA_end	Target_start	Target_end	miRNA_aligned_fragment	Target_aligned_fragment	Inhibition	Target_Desc.	Multiplicity
bra-miR172a	PvAP2-ERF173	0,5	11,33	1	20	3656	3675	AGAAUCUUGAUGAUGCUGCA	UGCAGCAUCAUCAGGAUUCU	Cleavage		1
bra-miR172a	PvAP2-ERF78	0,5	10,114	1	20	3797	3816	AGAAUCUUGAUGAUGCUGCA	UGCAGCAUCAUCAGGAUUCU	Cleavage		1
bra-miR172a	PvAP2-ERF20	1,5	9,626	1	20	2530	2549	AGAAUCUUGAUGAUGCUGCA	UGCAGCAUCAUCAGGAUUCC	Cleavage		1
bra-miR172a	PvAP2-ERF25	1,5	16,15	1	20	3064	3083	AGAAUCUUGAUGAUGCUGCA	UGCAGCAUCAUCAGGAUUCC	Cleavage		1
bra-miR172a	PvAP2-ERF62	1,5	16,463	1	20	3200	3219	AGAAUCUUGAUGAUGCUGCA	UGCAGCAUCAUCAGGAUUCC	Cleavage		1
bra-miR172a	PvAP2-ERF113	2,5	22,52	1	20	3572	3591	AGAAUCUUGAUGAUGCUGCA	AGCAGCAUCAUCAGGAUUCG	Cleavage		1
bra-miR172b-3p	PvAP2-ERF173	0,5	11,33	1	20	3656	3675	AGAAUCUUGAUGAUGCUGCA	UGCAGCAUCAUCAGGAUUCU	Cleavage		1
bra-miR172b-3p	PvAP2-ERF78	0,5	10,114	1	20	3797	3816	AGAAUCUUGAUGAUGCUGCA	UGCAGCAUCAUCAGGAUUCU	Cleavage		1
bra-miR172b-3p	PvAP2-ERF20	1,5	9,626	1	20	2530	2549	AGAAUCUUGAUGAUGCUGCA	UGCAGCAUCAUCAGGAUUCC	Cleavage		1
bra-miR172b-3p	PvAP2-ERF25	1,5	16,15	1	20	3064	3083	AGAAUCUUGAUGAUGCUGCA	UGCAGCAUCAUCAGGAUUCC	Cleavage		1
bra-miR172b-3p	PvAP2-ERF62	1,5	16,463	1	20	3200	3219	AGAAUCUUGAUGAUGCUGCA	UGCAGCAUCAUCAGGAUUCC	Cleavage		1
bra-miR172b-3p	PvAP2-ERF113	2,5	22,52	1	20	3572	3591	AGAAUCUUGAUGAUGCUGCA	AGCAGCAUCAUCAGGAUUCG	Cleavage		1
Cynara cardunculus												
miRNA_Acc.	Target_Acc.	Expectation	UPE	miRNA_start	miRNA_end	Target_start	Target_end	miRNA_aligned_fragment	Target_aligned_fragment	Inhibition	Target_Desc.	Multiplicity
cca-miR172	PvAP2-ERF173	0,5	11,33	1	20	3656	3675	AGAAUCUUGAUGAUGCUGCA	UGCAGCAUCAUCAGGAUUCU	Cleavage		1
cca-miR172	PvAP2-ERF78	0,5	10,114	1	20	3797	3816	AGAAUCUUGAUGAUGCUGCA	UGCAGCAUCAUCAGGAUUCU	Cleavage		1
cca-miR172	PvAP2-ERF20	1,5	9,626	1	20	2530	2549	AGAAUCUUGAUGAUGCUGCA	UGCAGCAUCAUCAGGAUUCC	Cleavage		1
cca-miR172	PvAP2-ERF25	1,5	16,15	1	20	3064	3083	AGAAUCUUGAUGAUGCUGCA	UGCAGCAUCAUCAGGAUUCC	Cleavage		1
cca-miR172	PvAP2-ERF62	1,5	16,463	1	20	3200	3219	AGAAUCUUGAUGAUGCUGCA	UGCAGCAUCAUCAGGAUUCC	Cleavage		1
cca-miR172	PvAP2-ERF113	2,5	22,52	1	20	3572	3591	AGAAUCUUGAUGAUGCUGCA	AGCAGCAUCAUCAGGAUUCG	Cleavage		1
cca-miR6107	PvAP2-ERF133	3	11,712	1	20	2832	2851	AAAGGGGACAAUAUCUGGUA	CACCAAAUGUUGUUCCCUUU	Cleavage		1
cca-miR6113	PvAP2-ERF141	2,5	15,62	1	22	696	717	UCUGAAACUCAAGAACACGUUG	CCACCUGUUCUUCAGUUUUAGA	Translation		1

Cucumis melo

miRNA_Acc.	Target_Acc.	Expectation	UPE	miRNA_start	miRNA_end	Target_start	Target_end	miRNA_aligned_fragment	Target_aligned_fragment	Inhibition	Target_Desc.	Multiplicity
cme-miR172a	PvAP2-ERF20	0,5	9,626	1	21	2529	2549	GGAAUCUUGAUGAUGCUGCAG	CUGCAGCAUCAUCAGGAUUCC	Cleavage		1
cme-miR172a	PvAP2-ERF25	0,5	16,15	1	21	3063	3083	GGAAUCUUGAUGAUGCUGCAG	CUGCAGCAUCAUCAGGAUUCC	Cleavage		1
cme-miR172a	PvAP2-ERF62	0,5	16,463	1	21	3199	3219	GGAAUCUUGAUGAUGCUGCAG	CUGCAGCAUCAUCAGGAUUCC	Cleavage		1
cme-miR172a	PvAP2-ERF173	1	11,33	1	21	3655	3675	GGAAUCUUGAUGAUGCUGCAG	CUGCAGCAUCAUCAGGAUUCU	Cleavage		1
cme-miR172a	PvAP2-ERF78	1	10,114	1	21	3796	3816	GGAAUCUUGAUGAUGCUGCAG	CUGCAGCAUCAUCAGGAUUCU	Cleavage		1
cme-miR172a	PvAP2-ERF113	2,5	22,52	1	21	3571	3591	GGAAUCUUGAUGAUGCUGCAG	CAGCAGCAUCAUCAGGAUUCG	Cleavage		1
cme-miR172b	PvAP2-ERF173	0,5	11,33	1	20	3656	3675	AGAAUCUUGAUGAUGCUGCA	UGCAGCAUCAUCAGGAUUCU	Cleavage		1
cme-miR172b	PvAP2-ERF78	0,5	10,114	1	20	3797	3816	AGAAUCUUGAUGAUGCUGCA	UGCAGCAUCAUCAGGAUUCU	Cleavage		1
cme-miR172b	PvAP2-ERF20	1,5	9,626	1	20	2530	2549	AGAAUCUUGAUGAUGCUGCA	UGCAGCAUCAUCAGGAUUCC	Cleavage		1
cme-miR172b	PvAP2-ERF25	1,5	16,15	1	20	3064	3083	AGAAUCUUGAUGAUGCUGCA	UGCAGCAUCAUCAGGAUUCC	Cleavage		1
cme-miR172b	PvAP2-ERF62	1,5	16,463	1	20	3200	3219	AGAAUCUUGAUGAUGCUGCA	UGCAGCAUCAUCAGGAUUCC	Cleavage		1
cme-miR172b	PvAP2-ERF113	2,5	22,52	1	20	3572	3591	AGAAUCUUGAUGAUGCUGCA	AGCAGCAUCAUCAGGAUUCG	Cleavage		1
cme-miR172c	PvAP2-ERF173	0,5	11,33	1	20	3656	3675	AGAAUCUUGAUGAUGCUGCA	UGCAGCAUCAUCAGGAUUCU	Cleavage		1
cme-miR172c	PvAP2-ERF78	0,5	10,114	1	20	3797	3816	AGAAUCUUGAUGAUGCUGCA	UGCAGCAUCAUCAGGAUUCU	Cleavage		1
cme-miR172c	PvAP2-ERF20	1,5	9,626	1	20	2530	2549	AGAAUCUUGAUGAUGCUGCA	UGCAGCAUCAUCAGGAUUCC	Cleavage		1
cme-miR172c	PvAP2-ERF25	1,5	16,15	1	20	3064	3083	AGAAUCUUGAUGAUGCUGCA	UGCAGCAUCAUCAGGAUUCC	Cleavage		1
cme-miR172c	PvAP2-ERF62	1,5	16,463	1	20	3200	3219	AGAAUCUUGAUGAUGCUGCA	UGCAGCAUCAUCAGGAUUCC	Cleavage		1
cme-miR172c	PvAP2-ERF113	2,5	22,52	1	20	3572	3591	AGAAUCUUGAUGAUGCUGCA	AGCAGCAUCAUCAGGAUUCG	Cleavage		1
cme-miR172d	PvAP2-ERF20	0,5	9,626	1	20	2530	2549	GGAAUCUUGAUGAUGCUGCA	UGCAGCAUCAUCAGGAUUCC	Cleavage		1
cme-miR172d	PvAP2-ERF25	0,5	16,15	1	20	3064	3083	GGAAUCUUGAUGAUGCUGCA	UGCAGCAUCAUCAGGAUUCC	Cleavage		1
cme-miR172d	PvAP2-ERF62	0,5	16,463	1	20	3200	3219	GGAAUCUUGAUGAUGCUGCA	UGCAGCAUCAUCAGGAUUCC	Cleavage		1
cme-miR172d	PvAP2-ERF173	1	11,33	1	20	3656	3675	GGAAUCUUGAUGAUGCUGCA	UGCAGCAUCAUCAGGAUUCU	Cleavage		1
cme-miR172d	PvAP2-ERF78	1	10,114	1	20	3797	3816	GGAAUCUUGAUGAUGCUGCA	UGCAGCAUCAUCAGGAUUCU	Cleavage		1
cme-miR172d	PvAP2-ERF113	2,5	22,52	1	20	3572	3591	GGAAUCUUGAUGAUGCUGCA	AGCAGCAUCAUCAGGAUUCG	Cleavage		1
cme-miR172e	PvAP2-ERF173	0,5	11,33	1	21	3655	3675	AGAAUCUUGAUGAUGCUGCAG	CUGCAGCAUCAUCAGGAUUCU	Cleavage		1
cme-miR172e	PvAP2-ERF78	0,5	10,114	1	21	3796	3816	AGAAUCUUGAUGAUGCUGCAG	CUGCAGCAUCAUCAGGAUUCU	Cleavage		1
cme-miR172e	PvAP2-ERF20	1,5	9,626	1	21	2529	2549	AGAAUCUUGAUGAUGCUGCAG	CUGCAGCAUCAUCAGGAUUCC	Cleavage		1
cme-miR172e	PvAP2-ERF25	1,5	16,15	1	21	3063	3083	AGAAUCUUGAUGAUGCUGCAG	CUGCAGCAUCAUCAGGAUUCC	Cleavage		1
cme-miR172e	PvAP2-ERF62	1,5	16,463	1	21	3199	3219	AGAAUCUUGAUGAUGCUGCAG	CUGCAGCAUCAUCAGGAUUCC	Cleavage		1
cme-miR172e	PvAP2-ERF113	2,5	22,52	1	21	3571	3591	AGAAUCUUGAUGAUGCUGCAG	CAGCAGCAUCAUCAGGAUUCG	Cleavage		1
cme-miR172f	PvAP2-ERF20	2,5	9,626	1	20	2530	2549	UGAAUCUUGAUGAUGCCGCA	UGCAGCAUCAUCAGGAUUCC	Cleavage		1
cme-miR172f	PvAP2-ERF25	2,5	16,15	1	20	3064	3083	UGAAUCUUGAUGAUGCCGCA	UGCAGCAUCAUCAGGAUUCC	Cleavage		1
cme-miR172f	PvAP2-ERF62	2,5	16,463	1	20	3200	3219	UGAAUCUUGAUGAUGCCGCA	UGCAGCAUCAUCAGGAUUCC	Cleavage		1
cme-miR172f	PvAP2-ERF173	2,5	11,33	1	20	3656	3675	UGAAUCUUGAUGAUGCCGCA	UGCAGCAUCAUCAGGAUUCU	Cleavage		1
cme-miR172f	PvAP2-ERF78	2,5	10,114	1	20	3797	3816	UGAAUCUUGAUGAUGCCGCA	UGCAGCAUCAUCAGGAUUCU	Cleavage		1
cme-miR172f	PvAP2-ERF113	3	22,52	1	20	3572	3591	UGAAUCUUGAUGAUGCCGCA	AGCAGCAUCAUCAGGAUUCG	Cleavage		1

Carica papaya

miRNA_Acc.	Target_Acc.	Expectation	UPE	miRNA_start	miRNA_end	Target_start	Target_end	miRNA_aligned_fragment	Target_aligned_fragment	Inhibition	Target_Desc.	Multiplicity
cpa-miR172a	PvAP2-ERF20	0,5	9,638	1	21	2530	2550	GGGAAUCUUGAUGAUGCUGCA	UGCAGCAUCAUCAGGAUUCCC	Cleavage		1
cpa-miR172a	PvAP2-ERF25	0,5	16,19	1	21	3064	3084	GGGAAUCUUGAUGAUGCUGCA	UGCAGCAUCAUCAGGAUUCCC	Cleavage		1
cpa-miR172a	PvAP2-ERF62	0,5	17,808	1	21	3200	3220	GGGAAUCUUGAUGAUGCUGCA	UGCAGCAUCAUCAGGAUUCCC	Cleavage		1
cpa-miR172a	PvAP2-ERF173	1	11,277	1	21	3656	3676	GGGAAUCUUGAUGAUGCUGCA	UGCAGCAUCAUCAGGAUUCUC	Cleavage		1
cpa-miR172a	PvAP2-ERF78	1	7,512	1	21	3797	3817	GGGAAUCUUGAUGAUGCUGCA	UGCAGCAUCAUCAGGAUUCUC	Cleavage		1
cpa-miR172a	PvAP2-ERF113	3	20,11	1	20	3573	3592	GGGAAUCUUGAUGAUGCUGC	GCAGCAUCAUCAGGAUUCGU	Cleavage		1
cpa-miR172b	PvAP2-ERF20	0,5	9,638	1	21	2530	2550	GGGAAUCUUGAUGAUGCUGCA	UGCAGCAUCAUCAGGAUUCCC	Cleavage		1
cpa-miR172b	PvAP2-ERF25	0,5	16,19	1	21	3064	3084	GGGAAUCUUGAUGAUGCUGCA	UGCAGCAUCAUCAGGAUUCCC	Cleavage		1
cpa-miR172b	PvAP2-ERF62	0,5	17,808	1	21	3200	3220	GGGAAUCUUGAUGAUGCUGCA	UGCAGCAUCAUCAGGAUUCCC	Cleavage		1
cpa-miR172b	PvAP2-ERF173	1	11,277	1	21	3656	3676	GGGAAUCUUGAUGAUGCUGCA	UGCAGCAUCAUCAGGAUUCUC	Cleavage		1
cpa-miR172b	PvAP2-ERF78	1	7,512	1	21	3797	3817	GGGAAUCUUGAUGAUGCUGCA	UGCAGCAUCAUCAGGAUUCUC	Cleavage		1
cpa-miR172b	PvAP2-ERF113	3	20,11	1	20	3573	3592	GGGAAUCUUGAUGAUGCUGC	GCAGCAUCAUCAGGAUUCGU	Cleavage		1

Citrus reticulata

miRNA_Acc.	Target_Acc.	Expectation	UPE	miRNA_start	miRNA_end	Target_start	Target_end	miRNA_aligned_fragment	Target_aligned_fragment	Inhibition	Target_Desc.	Multiplicity
cre-miR1171	PvAP2-ERF171	2	9,458	1	23	130	152	UGGAGUGGAGUGGAGUGGAGUGG	UCACUUCACUUCACUUCACUUCA	Cleavage		1

Citrus sinensis

miRNA_Acc.	Target_Acc.	Expectation	UPE	miRNA_start	miRNA_end	Target_start	Target_end	miRNA_aligned_fragment	Target_aligned_fragment	Inhibition	Target_Desc.	Multiplicity
csi-miR172a-3p	PvAP2-ERF173	0,5	11,33	1	20	3656	3675	AGAAUCUUGAUGAUGCUGCA	UGCAGCAUCAUCAGGAUUCU	Cleavage		1
csi-miR172a-3p	PvAP2-ERF78	0,5	10,114	1	20	3797	3816	AGAAUCUUGAUGAUGCUGCA	UGCAGCAUCAUCAGGAUUCU	Cleavage		1
csi-miR172a-3p	PvAP2-ERF20	1,5	9,626	1	20	2530	2549	AGAAUCUUGAUGAUGCUGCA	UGCAGCAUCAUCAGGAUUCC	Cleavage		1
csi-miR172a-3p	PvAP2-ERF25	1,5	16,15	1	20	3064	3083	AGAAUCUUGAUGAUGCUGCA	UGCAGCAUCAUCAGGAUUCC	Cleavage		1
csi-miR172a-3p	PvAP2-ERF62	1,5	16,463	1	20	3200	3219	AGAAUCUUGAUGAUGCUGCA	UGCAGCAUCAUCAGGAUUCC	Cleavage		1
csi-miR172a-3p	PvAP2-ERF113	2,5	22,52	1	20	3572	3591	AGAAUCUUGAUGAUGCUGCA	AGCAGCAUCAUCAGGAUUCG	Cleavage		1
csi-miR172b	PvAP2-ERF173	1,5	11,33	1	20	3656	3675	AGAAUCUUGAUGAUGCGGCA	UGCAGCAUCAUCAGGAUUCU	Cleavage		1
csi-miR172b	PvAP2-ERF78	1,5	10,114	1	20	3797	3816	AGAAUCUUGAUGAUGCGGCA	UGCAGCAUCAUCAGGAUUCU	Cleavage		1
csi-miR172b	PvAP2-ERF20	2,5	9,626	1	20	2530	2549	AGAAUCUUGAUGAUGCGGCA	UGCAGCAUCAUCAGGAUUCC	Cleavage		1
csi-miR172b	PvAP2-ERF25	2,5	16,15	1	20	3064	3083	AGAAUCUUGAUGAUGCGGCA	UGCAGCAUCAUCAGGAUUCC	Cleavage		1
csi-miR172b	PvAP2-ERF62	2,5	16,463	1	20	3200	3219	AGAAUCUUGAUGAUGCGGCA	UGCAGCAUCAUCAGGAUUCC	Cleavage		1
csi-miR172c	PvAP2-ERF20	1,5	9,638	1	22	2529	2550	UGGAAUCUUGAUGAUGCUGCAG	CUGCAGCAUCAUCAGGAUUCCC	Cleavage		1
csi-miR172c	PvAP2-ERF25	1,5	16,19	1	22	3063	3084	UGGAAUCUUGAUGAUGCUGCAG	CUGCAGCAUCAUCAGGAUUCCC	Cleavage		1
csi-miR172c	PvAP2-ERF62	1,5	17,808	1	22	3199	3220	UGGAAUCUUGAUGAUGCUGCAG	CUGCAGCAUCAUCAGGAUUCCC	Cleavage		1
csi-miR172c	PvAP2-ERF173	2	11,277	1	22	3655	3676	UGGAAUCUUGAUGAUGCUGCAG	CUGCAGCAUCAUCAGGAUUCUC	Cleavage		1
csi-miR172c	PvAP2-ERF78	2	7,512	1	22	3796	3817	UGGAAUCUUGAUGAUGCUGCAG	CUGCAGCAUCAUCAGGAUUCUC	Cleavage		1
csi-miR172c	PvAP2-ERF113	3	20,11	1	22	3571	3592	UGGAAUCUUGAUGAUGCUGCAG	CAGCAGCAUCAUCAGGAUUCGU	Cleavage		1
csi-miR3946	PvAP2-ERF31	3	4,15	1	21	741	761	UUGUAGAGAAAGAGAAGAGAG	CUCUUUUUUUUUUCUCCACAA	Cleavage		1
csi-miR3946	PvAP2-ERF133	3	7,44	1	23	4887	4909	UUGUAGAGAAAGAGAAGAGAGCA	UUUUCUUUUAUUUUUUUUUACAA	Cleavage		1
csi-miR3946	PvAP2-ERF128	3	3,141	1	21	30	49	UUGUAGAGAAAGAGAAGAGAG	CUCUCUU-UCUCUCUCUACAA	Translation		1
csi-miR3948	PvAP2-ERF163	2,5	0,791	1	22	182	203	UGGAGUGGGAGUGGGAGUAGGG	CUCUACUUCCACUUCCACUUCU	Cleavage		1
csi-miR3948	PvAP2-ERF10	3	1,147	1	24	18	40	UGGAGUGGGAGUGGGAGUAGGGUG	CACUC-ACUCCCACUCCCAUUUCA	Cleavage		1
csi-miR3948	PvAP2-ERF36	3	1,201	1	24	196	219	UGGAGUGGGAGUGGGAGUAGGGUG	CAAACUACUCACACUCUCACACCA	Cleavage		1
csi-miR3951	PvAP2-ERF152	3	10,261	1	20	2518	2537	UAGAUAAAGAUGAGAGAAAA	AUUUCUCUUGUUUUUAUUUA	Cleavage		1

Dictyostelium discoideum

miRNA_Acc.	Target_Acc.	Expectation	UPE	miRNA_start	miRNA_end	Target_start	Target_end	miRNA_aligned_fragment	Target_aligned_fragment	Inhibition	Target_Desc.	Multiplicity
ddi-miR-7095-3p	PvAP2-ERF32	2,5	13,389	1	20	3304	3323	GGAUCUGUUAAAAUUGGAUA	UAUUCAGUUUUGAUAGAUCU	Cleavage		1

Digitalis purpurea

miRNA_Acc.	Target_Acc.	Expectation	UPE	miRNA_start	miRNA_end	Target_start	Target_end	miRNA_aligned_fragment	Target_aligned_fragment	Inhibition	Target_Desc.	Multiplicity
dpr-miR172a	PvAP2-ERF173	0,5	11,33	1	20	3656	3675	AGAAUCUUGAUGAUGCUGCA	UGCAGCAUCAUCAGGAUUCU	Cleavage		1
dpr-miR172a	PvAP2-ERF78	0,5	10,114	1	20	3797	3816	AGAAUCUUGAUGAUGCUGCA	UGCAGCAUCAUCAGGAUUCU	Cleavage		1
dpr-miR172a	PvAP2-ERF20	1,5	9,626	1	20	2530	2549	AGAAUCUUGAUGAUGCUGCA	UGCAGCAUCAUCAGGAUUCC	Cleavage		1
dpr-miR172a	PvAP2-ERF25	1,5	16,15	1	20	3064	3083	AGAAUCUUGAUGAUGCUGCA	UGCAGCAUCAUCAGGAUUCC	Cleavage		1
dpr-miR172a	PvAP2-ERF62	1,5	16,463	1	20	3200	3219	AGAAUCUUGAUGAUGCUGCA	UGCAGCAUCAUCAGGAUUCC	Cleavage		1
dpr-miR172a	PvAP2-ERF113	2,5	22,52	1	20	3572	3591	AGAAUCUUGAUGAUGCUGCA	AGCAGCAUCAUCAGGAUUCG	Cleavage		1
dpr-miR172b	PvAP2-ERF173	0,5	11,33	1	20	3656	3675	AGAAUCUUGAUGAUGCUGCA	UGCAGCAUCAUCAGGAUUCU	Cleavage		1
dpr-miR172b	PvAP2-ERF78	0,5	10,114	1	20	3797	3816	AGAAUCUUGAUGAUGCUGCA	UGCAGCAUCAUCAGGAUUCU	Cleavage		1
dpr-miR172b	PvAP2-ERF20	1,5	9,626	1	20	2530	2549	AGAAUCUUGAUGAUGCUGCA	UGCAGCAUCAUCAGGAUUCC	Cleavage		1
dpr-miR172b	PvAP2-ERF25	1,5	16,15	1	20	3064	3083	AGAAUCUUGAUGAUGCUGCA	UGCAGCAUCAUCAGGAUUCC	Cleavage		1
dpr-miR172b	PvAP2-ERF62	1,5	16,463	1	20	3200	3219	AGAAUCUUGAUGAUGCUGCA	UGCAGCAUCAUCAGGAUUCC	Cleavage		1
dpr-miR172b	PvAP2-ERF113	2,5	22,52	1	20	3572	3591	AGAAUCUUGAUGAUGCUGCA	AGCAGCAUCAUCAGGAUUCG	Cleavage		1

Elaeis guineensis

miRNA_Acc.	Target_Acc.	Expectation	UPE	miRNA_start	miRNA_end	Target_start	Target_end	miRNA_aligned_fragment	Target_aligned_fragment	Inhibition	Target_Desc.	Multiplicity
egu-miR172a	PvAP2-ERF173	0,5	11,33	1	20	3656	3675	AGAAUCUUGAUGAUGCUGCA	UGCAGCAUCAUCAGGAUUCU	Cleavage		1
egu-miR172a	PvAP2-ERF78	0,5	10,114	1	20	3797	3816	AGAAUCUUGAUGAUGCUGCA	UGCAGCAUCAUCAGGAUUCU	Cleavage		1
egu-miR172a	PvAP2-ERF20	1,5	9,626	1	20	2530	2549	AGAAUCUUGAUGAUGCUGCA	UGCAGCAUCAUCAGGAUUCC	Cleavage		1
egu-miR172a	PvAP2-ERF25	1,5	16,15	1	20	3064	3083	AGAAUCUUGAUGAUGCUGCA	UGCAGCAUCAUCAGGAUUCC	Cleavage		1
egu-miR172a	PvAP2-ERF62	1,5	16,463	1	20	3200	3219	AGAAUCUUGAUGAUGCUGCA	UGCAGCAUCAUCAGGAUUCC	Cleavage		1
egu-miR172a	PvAP2-ERF113	2,5	22,52	1	20	3572	3591	AGAAUCUUGAUGAUGCUGCA	AGCAGCAUCAUCAGGAUUCG	Cleavage		1
egu-miR172b	PvAP2-ERF173	0,5	11,33	1	20	3656	3675	AGAAUCUUGAUGAUGCUGCA	UGCAGCAUCAUCAGGAUUCU	Cleavage		1
egu-miR172b	PvAP2-ERF78	0,5	10,114	1	20	3797	3816	AGAAUCUUGAUGAUGCUGCA	UGCAGCAUCAUCAGGAUUCU	Cleavage		1
egu-miR172b	PvAP2-ERF20	1,5	9,626	1	20	2530	2549	AGAAUCUUGAUGAUGCUGCA	UGCAGCAUCAUCAGGAUUCC	Cleavage		1
egu-miR172b	PvAP2-ERF25	1,5	16,15	1	20	3064	3083	AGAAUCUUGAUGAUGCUGCA	UGCAGCAUCAUCAGGAUUCC	Cleavage		1

egu-miR172b	PvAP2-ERF62	1,5	16,463	1	20	3200	3219	AGAAUCUUGAUGAUGCUGCA	UGCAGCAUCAUCAGGAUUCC	Cleavage		1
egu-miR172b	PvAP2-ERF113	2,5	22,52	1	20	3572	3591	AGAAUCUUGAUGAUGCUGCA	AGCAGCAUCAUCAGGAUUCG	Cleavage		1
egu-miR172c	PvAP2-ERF173	0,5	11,33	1	20	3656	3675	AGAAUCUUGAUGAUGCUGCA	UGCAGCAUCAUCAGGAUUCU	Cleavage		1
egu-miR172c	PvAP2-ERF78	0,5	10,114	1	20	3797	3816	AGAAUCUUGAUGAUGCUGCA	UGCAGCAUCAUCAGGAUUCU	Cleavage		1
egu-miR172c	PvAP2-ERF20	1,5	9,626	1	20	2530	2549	AGAAUCUUGAUGAUGCUGCA	UGCAGCAUCAUCAGGAUUCC	Cleavage		1
egu-miR172c	PvAP2-ERF25	1,5	16,15	1	20	3064	3083	AGAAUCUUGAUGAUGCUGCA	UGCAGCAUCAUCAGGAUUCC	Cleavage		1
egu-miR172c	PvAP2-ERF62	1,5	16,463	1	20	3200	3219	AGAAUCUUGAUGAUGCUGCA	UGCAGCAUCAUCAGGAUUCC	Cleavage		1
egu-miR172c	PvAP2-ERF113	2,5	22,52	1	20	3572	3591	AGAAUCUUGAUGAUGCUGCA	AGCAGCAUCAUCAGGAUUCG	Cleavage		1
egu-miR172d	PvAP2-ERF20	1,5	9,626	1	20	2530	2549	UGAAUCUUGAUGAUGCUGCA	UGCAGCAUCAUCAGGAUUCC	Cleavage		1
egu-miR172d	PvAP2-ERF25	1,5	16,15	1	20	3064	3083	UGAAUCUUGAUGAUGCUGCA	UGCAGCAUCAUCAGGAUUCC	Cleavage		1
egu-miR172d	PvAP2-ERF62	1,5	16,463	1	20	3200	3219	UGAAUCUUGAUGAUGCUGCA	UGCAGCAUCAUCAGGAUUCC	Cleavage		1
egu-miR172d	PvAP2-ERF173	1,5	11,33	1	20	3656	3675	UGAAUCUUGAUGAUGCUGCA	UGCAGCAUCAUCAGGAUUCU	Cleavage		1
egu-miR172d	PvAP2-ERF78	1,5	10,114	1	20	3797	3816	UGAAUCUUGAUGAUGCUGCA	UGCAGCAUCAUCAGGAUUCU	Cleavage		1
egu-miR172d	PvAP2-ERF113	2	22,52	1	20	3572	3591	UGAAUCUUGAUGAUGCUGCA	AGCAGCAUCAUCAGGAUUCG	Cleavage		1
egu-miR172e	PvAP2-ERF20	1,5	9,626	1	20	2530	2549	UGAAUCUUGAUGAUGCUGCA	UGCAGCAUCAUCAGGAUUCC	Cleavage		1
egu-miR172e	PvAP2-ERF25	1,5	16,15	1	20	3064	3083	UGAAUCUUGAUGAUGCUGCA	UGCAGCAUCAUCAGGAUUCC	Cleavage		1
egu-miR172e	PvAP2-ERF62	1,5	16,463	1	20	3200	3219	UGAAUCUUGAUGAUGCUGCA	UGCAGCAUCAUCAGGAUUCC	Cleavage		1
egu-miR172e	PvAP2-ERF173	1,5	11,33	1	20	3656	3675	UGAAUCUUGAUGAUGCUGCA	UGCAGCAUCAUCAGGAUUCU	Cleavage		1
egu-miR172e	PvAP2-ERF78	1,5	10,114	1	20	3797	3816	UGAAUCUUGAUGAUGCUGCA	UGCAGCAUCAUCAGGAUUCU	Cleavage		1
egu-miR172e	PvAP2-ERF113	2	22,52	1	20	3572	3591	UGAAUCUUGAUGAUGCUGCA	AGCAGCAUCAUCAGGAUUCG	Cleavage		1
egu-miR172f	PvAP2-ERF78	1,5	10,114	1	20	3797	3816	UGAAUCUUGAUGAUGCUGCA	UGCAGCAUCAUCAGGAUUCU	Cleavage		1
egu-miR172f	PvAP2-ERF173	1,5	11,33	1	20	3656	3675	UGAAUCUUGAUGAUGCUGCA	UGCAGCAUCAUCAGGAUUCU	Cleavage		1
egu-miR172f	PvAP2-ERF62	1,5	16,463	1	20	3200	3219	UGAAUCUUGAUGAUGCUGCA	UGCAGCAUCAUCAGGAUUCC	Cleavage		1
egu-miR172f	PvAP2-ERF25	1,5	16,15	1	20	3064	3083	UGAAUCUUGAUGAUGCUGCA	UGCAGCAUCAUCAGGAUUCC	Cleavage		1
egu-miR172f	PvAP2-ERF20	1,5	9,626	1	20	2530	2549	UGAAUCUUGAUGAUGCUGCA	UGCAGCAUCAUCAGGAUUCC	Cleavage		1
egu-miR172f	PvAP2-ERF113	2	22,52	1	20	3572	3591	UGAAUCUUGAUGAUGCUGCA	AGCAGCAUCAUCAGGAUUCG	Cleavage		1
Ectocarpus siliculosus												
miRNA_Acc.	Target_Acc.	Expectation	UPE	miRNA_start	miRNA_end	Target_start	Target_end	miRNA_aligned_fragment	Target_aligned_fragment	Inhibition	Target_Desc.	Multiplicity
esi-miR3463-5p	PvAP2-ERF150	3	8,131	1	20	1663	1682	UCAUUGCUGGUGCUCGUUGG	UCAACCACCACUAGCAAUGA	Cleavage		1
Festuca arundinacea												
miRNA_Acc.	Target_Acc.	Expectation	UPE	miRNA_start	miRNA_end	Target_start	Target_end	miRNA_aligned_fragment	Target_aligned_fragment	Inhibition	Target_Desc.	Multiplicity
far-miR1134	PvAP2-ERF5	3	12,992	1	20	491	510	CGACAACAACAACAAGAAGA	UUGUCUUGUUGUUGUUGUUU	Cleavage		1
far-miR529	PvAP2-ERF91	3	6,596	1	20	307	326	AGAAGAGAGAGAGCACAGCU	AUCUAUGCUCUUUCUUUUCU	Cleavage		1
Gossypium hirsutum												
miRNA_Acc.	Target_Acc.	Expectation	UPE	miRNA_start	miRNA_end	Target_start	Target_end	miRNA_aligned_fragment	Target_aligned_fragment	Inhibition	Target_Desc.	Multiplicity
ghr-miR172	PvAP2-ERF173	0	11,33	1	21	3655	3675	AGAAUCCUGAUGAUGCUGCAG	CUGCAGCAUCAUCAGGAUUCU	Cleavage		1
ghr-miR172	PvAP2-ERF78	0	10,114	1	21	3796	3816	AGAAUCCUGAUGAUGCUGCAG	CUGCAGCAUCAUCAGGAUUCU	Cleavage		1
ghr-miR172	PvAP2-ERF20	1	9,626	1	21	2529	2549	AGAAUCCUGAUGAUGCUGCAG	CUGCAGCAUCAUCAGGAUUCC	Cleavage		1
ghr-miR172	PvAP2-ERF25	1	16,15	1	21	3063	3083	AGAAUCCUGAUGAUGCUGCAG	CUGCAGCAUCAUCAGGAUUCC	Cleavage		1
ghr-miR172	PvAP2-ERF62	1	16,463	1	21	3199	3219	AGAAUCCUGAUGAUGCUGCAG	CUGCAGCAUCAUCAGGAUUCC	Cleavage		1
ghr-miR172	PvAP2-ERF113	2	22,52	1	21	3571	3591	AGAAUCCUGAUGAUGCUGCAG	CAGCAGCAUCAUCAGGAUUCG	Cleavage		1
ghr-miR7484a	PvAP2-ERF113	3	13,986	1	21	2931	2951	UUUGUAUAUUAGAUCAAAGAG	UUUUUUGAUUAAGUAUAUAAA	Translation		1
ghr-miR7484b	PvAP2-ERF113	3	13,986	1	21	2931	2951	UUUGUAUAUUAGAUCAAAGAG	UUUUUUGAUUAAGUAUAUAAA	Translation		1
ghr-miR7510a	PvAP2-ERF10	3	16,85	1	21	1060	1080	AAGGUCAUGAUCUUUAGCGGC	GCUGCUAGAGCUUAUGAUCUU	Translation		1
Glycine max												
miRNA_Acc.	Target_Acc.	Expectation	UPE	miRNA_start	miRNA_end	Target_start	Target_end	miRNA_aligned_fragment	Target_aligned_fragment	Inhibition	Target_Desc.	Multiplicity
gma-miR1507c-5p	PvAP2-ERF114	2,5	17,551	1	20	28	47	GAGGUGUUUGGGAUGAGAGA	UUUUGCAUCCCAAACACCUU	Cleavage		1
gma-miR1525	PvAP2-ERF32	3	10,22	1	21	2634	2654	UGGGUUAAUUAAGUUUUUAGU	GUUAAAAAUUUUAUUAACCUG	Translation		1
gma-miR156p	PvAP2-ERF113	3	12,404	1	20	2612	2631	UUGACAGAAGAAAGGGAGCA	UGUUUCUUUUCUAUUGUCAA	Cleavage		1
gma-miR156t	PvAP2-ERF113	3	12,404	1	20	2612	2631	UUGACAGAAGAAAGGGAGCA	UGUUUCUUUUCUAUUGUCAA	Cleavage		1
gma-miR172a	PvAP2-ERF173	0,5	11,33	1	20	3656	3675	AGAAUCUUGAUGAUGCUGCA	UGCAGCAUCAUCAGGAUUCU	Cleavage		1
gma-miR172a	PvAP2-ERF78	0,5	10,114	1	20	3797	3816	AGAAUCUUGAUGAUGCUGCA	UGCAGCAUCAUCAGGAUUCU	Cleavage		1
gma-miR172a	PvAP2-ERF20	1,5	9,626	1	20	2530	2549	AGAAUCUUGAUGAUGCUGCA	UGCAGCAUCAUCAGGAUUCC	Cleavage		1
gma-miR172a	PvAP2-ERF25	1,5	16,15	1	20	3064	3083	AGAAUCUUGAUGAUGCUGCA	UGCAGCAUCAUCAGGAUUCC	Cleavage		1
gma-miR172a	PvAP2-ERF62	1,5	16,463	1	20	3200	3219	AGAAUCUUGAUGAUGCUGCA	UGCAGCAUCAUCAGGAUUCC	Cleavage		1

gma-miR172a	PvAP2-ERF113	2,5	22,52	1	20	3572	3591	AGAAUCUUGAUGAUGCUGCA	AGCAGCAUCAUCAGGAUUCG	Cleavage	1
gma-miR172b-3p	PvAP2-ERF173	0,5	11,33	1	20	3656	3675	AGAAUCUUGAUGAUGCUGCA	UGCAGCAUCAUCAGGAUUCU	Cleavage	1
gma-miR172b-3p	PvAP2-ERF78	0,5	10,114	1	20	3797	3816	AGAAUCUUGAUGAUGCUGCA	UGCAGCAUCAUCAGGAUUCU	Cleavage	1
gma-miR172b-3p	PvAP2-ERF20	1,5	9,626	1	20	2530	2549	AGAAUCUUGAUGAUGCUGCA	UGCAGCAUCAUCAGGAUUCC	Cleavage	1
gma-miR172b-3p	PvAP2-ERF25	1,5	16,15	1	20	3064	3083	AGAAUCUUGAUGAUGCUGCA	UGCAGCAUCAUCAGGAUUCC	Cleavage	1
gma-miR172b-3p	PvAP2-ERF62	1,5	16,463	1	20	3200	3219	AGAAUCUUGAUGAUGCUGCA	UGCAGCAUCAUCAGGAUUCC	Cleavage	1
gma-miR172b-3p	PvAP2-ERF113	2,5	22,52	1	20	3572	3591	AGAAUCUUGAUGAUGCUGCA	AGCAGCAUCAUCAGGAUUCG	Cleavage	1
gma-miR172c	PvAP2-ERF20	0,5	9,626	1	21	2529	2549	GGAAUCUUGAUGAUGCUGCAG	CUGCAGCAUCAUCAGGAUUCC	Cleavage	1
gma-miR172c	PvAP2-ERF25	0,5	16,15	1	21	3063	3083	GGAAUCUUGAUGAUGCUGCAG	CUGCAGCAUCAUCAGGAUUCC	Cleavage	1
gma-miR172c	PvAP2-ERF62	0,5	16,463	1	21	3199	3219	GGAAUCUUGAUGAUGCUGCAG	CUGCAGCAUCAUCAGGAUUCC	Cleavage	1
gma-miR172c	PvAP2-ERF173	1	11,33	1	21	3655	3675	GGAAUCUUGAUGAUGCUGCAG	CUGCAGCAUCAUCAGGAUUCU	Cleavage	1
gma-miR172c	PvAP2-ERF78	1	10,114	1	21	3796	3816	GGAAUCUUGAUGAUGCUGCAG	CUGCAGCAUCAUCAGGAUUCU	Cleavage	1
gma-miR172c	PvAP2-ERF113	2,5	22,52	1	21	3571	3591	GGAAUCUUGAUGAUGCUGCAG	CAGCAGCAUCAUCAGGAUUCG	Cleavage	1
gma-miR172d	PvAP2-ERF25	0,5	16,15	1	24	3060	3083	GGAAUCUUGAUGAUGCUGCAGCAG	CUGCUGCAGCAUCAUCAGGAUUCC	Cleavage	1
gma-miR172d	PvAP2-ERF62	0,5	16,463	1	24	3196	3219	GGAAUCUUGAUGAUGCUGCAGCAG	CUGCUGCAGCAUCAUCAGGAUUCC	Cleavage	1
gma-miR172d	PvAP2-ERF173	1	11,33	1	24	3652	3675	GGAAUCUUGAUGAUGCUGCAGCAG	CUGCUGCAGCAUCAUCAGGAUUCU	Cleavage	1
gma-miR172d	PvAP2-ERF20	0,5	9,626	1	24	2526	2549	GGAAUCUUGAUGAUGCUGCAGCAG	CUACUGCAGCAUCAUCAGGAUUCC	Cleavage	1
gma-miR172d	PvAP2-ERF78	1	10,114	1	24	3793	3816	GGAAUCUUGAUGAUGCUGCAGCAG	CUACUGCAGCAUCAUCAGGAUUCU	Cleavage	1
gma-miR172d	PvAP2-ERF113	2,5	22,52	1	23	3569	3591	GGAAUCUUGAUGAUGCUGCAGCA	UGCAGCAGCAUCAUCAGGAUUCG	Cleavage	1
gma-miR172e	PvAP2-ERF25	0,5	16,15	1	24	3060	3083	GGAAUCUUGAUGAUGCUGCAGCAG	CUGCUGCAGCAUCAUCAGGAUUCC	Cleavage	1
gma-miR172e	PvAP2-ERF62	0,5	16,463	1	24	3196	3219	GGAAUCUUGAUGAUGCUGCAGCAG	CUGCUGCAGCAUCAUCAGGAUUCC	Cleavage	1
gma-miR172e	PvAP2-ERF173	1	11,33	1	24	3652	3675	GGAAUCUUGAUGAUGCUGCAGCAG	CUGCUGCAGCAUCAUCAGGAUUCU	Cleavage	1
gma-miR172e	PvAP2-ERF20	0,5	9,626	1	24	2526	2549	GGAAUCUUGAUGAUGCUGCAGCAG	CUACUGCAGCAUCAUCAGGAUUCC	Cleavage	1
gma-miR172e	PvAP2-ERF78	1	10,114	1	24	3793	3816	GGAAUCUUGAUGAUGCUGCAGCAG	CUACUGCAGCAUCAUCAGGAUUCU	Cleavage	1
gma-miR172e	PvAP2-ERF113	2,5	22,52	1	23	3569	3591	GGAAUCUUGAUGAUGCUGCAGCA	UGCAGCAGCAUCAUCAGGAUUCG	Cleavage	1
gma-miR172f	PvAP2-ERF173	0,5	11,33	1	20	3656	3675	AGAAUCUUGAUGAUGCUGCA	UGCAGCAUCAUCAGGAUUCU	Cleavage	1
gma-miR172f	PvAP2-ERF78	0,5	10,114	1	20	3797	3816	AGAAUCUUGAUGAUGCUGCA	UGCAGCAUCAUCAGGAUUCU	Cleavage	1
gma-miR172f	PvAP2-ERF20	1,5	9,626	1	20	2530	2549	AGAAUCUUGAUGAUGCUGCA	UGCAGCAUCAUCAGGAUUCC	Cleavage	1
gma-miR172f	PvAP2-ERF25	1,5	16,15	1	20	3064	3083	AGAAUCUUGAUGAUGCUGCA	UGCAGCAUCAUCAGGAUUCC	Cleavage	1
gma-miR172f	PvAP2-ERF62	1,5	16,463	1	20	3200	3219	AGAAUCUUGAUGAUGCUGCA	UGCAGCAUCAUCAGGAUUCC	Cleavage	1
gma-miR172f	PvAP2-ERF113	2,5	22,52	1	20	3572	3591	AGAAUCUUGAUGAUGCUGCA	AGCAGCAUCAUCAGGAUUCG	Cleavage	1
gma-miR172h-3p	PvAP2-ERF173	0,5	11,33	1	20	3656	3675	AGAAUCUUGAUGAUGCUGCA	UGCAGCAUCAUCAGGAUUCU	Cleavage	1
gma-miR172h-3p	PvAP2-ERF78	0,5	10,114	1	20	3797	3816	AGAAUCUUGAUGAUGCUGCA	UGCAGCAUCAUCAGGAUUCU	Cleavage	1
gma-miR172h-3p	PvAP2-ERF20	1,5	9,626	1	20	2530	2549	AGAAUCUUGAUGAUGCUGCA	UGCAGCAUCAUCAGGAUUCC	Cleavage	1
gma-miR172h-3p	PvAP2-ERF25	1,5	16,15	1	20	3064	3083	AGAAUCUUGAUGAUGCUGCA	UGCAGCAUCAUCAGGAUUCC	Cleavage	1
gma-miR172h-3p	PvAP2-ERF62	1,5	16,463	1	20	3200	3219	AGAAUCUUGAUGAUGCUGCA	UGCAGCAUCAUCAGGAUUCC	Cleavage	1
gma-miR172h-3p	PvAP2-ERF113	2,5	22,52	1	20	3572	3591	AGAAUCUUGAUGAUGCUGCA	AGCAGCAUCAUCAGGAUUCG	Cleavage	1
gma-miR172i-3p	PvAP2-ERF20	0,5	9,626	1	20	2530	2549	GGAAUCUUGAUGAUGCUGCA	UGCAGCAUCAUCAGGAUUCC	Cleavage	1
gma-miR172i-3p	PvAP2-ERF25	0,5	16,15	1	20	3064	3083	GGAAUCUUGAUGAUGCUGCA	UGCAGCAUCAUCAGGAUUCC	Cleavage	1
gma-miR172i-3p	PvAP2-ERF62	0,5	16,463	1	20	3200	3219	GGAAUCUUGAUGAUGCUGCA	UGCAGCAUCAUCAGGAUUCC	Cleavage	1
gma-miR172i-3p	PvAP2-ERF173	1	11,33	1	20	3656	3675	GGAAUCUUGAUGAUGCUGCA	UGCAGCAUCAUCAGGAUUCU	Cleavage	1
gma-miR172i-3p	PvAP2-ERF78	1	10,114	1	20	3797	3816	GGAAUCUUGAUGAUGCUGCA	UGCAGCAUCAUCAGGAUUCU	Cleavage	1
gma-miR172i-3p	PvAP2-ERF113	2,5	22,52	1	20	3572	3591	GGAAUCUUGAUGAUGCUGCA	AGCAGCAUCAUCAGGAUUCG	Cleavage	1
gma-miR172k	PvAP2-ERF20	1,5	9,626	1	20	2530	2549	UGAAUCUUGAUGAUGCUGCA	UGCAGCAUCAUCAGGAUUCC	Cleavage	1
gma-miR172k	PvAP2-ERF25	1,5	16,15	1	20	3064	3083	UGAAUCUUGAUGAUGCUGCA	UGCAGCAUCAUCAGGAUUCC	Cleavage	1
gma-miR172k	PvAP2-ERF62	1,5	16,463	1	20	3200	3219	UGAAUCUUGAUGAUGCUGCA	UGCAGCAUCAUCAGGAUUCC	Cleavage	1
gma-miR172k	PvAP2-ERF173	1,5	11,33	1	20	3656	3675	UGAAUCUUGAUGAUGCUGCA	UGCAGCAUCAUCAGGAUUCU	Cleavage	1
gma-miR172k	PvAP2-ERF78	1,5	10,114	1	20	3797	3816	UGAAUCUUGAUGAUGCUGCA	UGCAGCAUCAUCAGGAUUCU	Cleavage	1
gma-miR172k	PvAP2-ERF113	2	22,52	1	20	3572	3591	UGAAUCUUGAUGAUGCUGCA	AGCAGCAUCAUCAGGAUUCG	Cleavage	1
gma-miR172l	PvAP2-ERF20	0,5	9,626	1	20	2530	2549	GGAAUCUUGAUGAUGCUGCA	UGCAGCAUCAUCAGGAUUCC	Cleavage	1
gma-miR172l	PvAP2-ERF25	0,5	16,15	1	20	3064	3083	GGAAUCUUGAUGAUGCUGCA	UGCAGCAUCAUCAGGAUUCC	Cleavage	1
gma-miR172l	PvAP2-ERF62	0,5	16,463	1	20	3200	3219	GGAAUCUUGAUGAUGCUGCA	UGCAGCAUCAUCAGGAUUCC	Cleavage	1
gma-miR172l	PvAP2-ERF173	1	11,33	1	20	3656	3675	GGAAUCUUGAUGAUGCUGCA	UGCAGCAUCAUCAGGAUUCU	Cleavage	1
gma-miR172l	PvAP2-ERF78	1	10,114	1	20	3797	3816	GGAAUCUUGAUGAUGCUGCA	UGCAGCAUCAUCAGGAUUCU	Cleavage	1
gma-miR172l	PvAP2-ERF113	2,5	22,52	1	20	3572	3591	GGAAUCUUGAUGAUGCUGCA	AGCAGCAUCAUCAGGAUUCG	Cleavage	1

gma-miR4393b	PvAP2-ERF150	3	9,956	1	20	628	647	UUGAAAAGGGACAGCAGAGA	UUUAGGCUGUCCCUUUUUAA	Cleavage		1
gma-miR4400	PvAP2-ERF120	3	17,994	1	21	4619	4639	UUCGGAAAAAUUCUGGAAGAC	GUUGUCCACAAUUUUUCUGAA	Cleavage		1
gma-miR4407	PvAP2-ERF27	3	24,702	1	20	30	49	CAGAGGAAGCAGCACUUGUA	UGUUGGUGCUGCUUCUUCUG	Cleavage		1
gma-miR4407	PvERF3	3	19,389	1	20	3713	3732	CAGAGGAAGCAGCACUUGUA	UAGAGGUUCUGCUUUCUCUG	Cleavage		1
gma-miR4993	PvAP2-ERF73	2,5	12,57	1	20	569	588	GAGCGGCGGCGGUGGAGGAU	CUCCGCCGCCGCCGCCGCUC	Cleavage		1
gma-miR5772	PvAP2-ERF152	3	8,682	1	22	3288	3309	AGAAUGUGAGUUAGAGUGAGCA	UGUACACUCUAACACACAUUUU	Translation		1
gma-miR5779	PvAP2-ERF152	3	15,076	1	22	495	516	CAAGUCCAAAGUAGGAAUGUUG	UAACGUUUUUCCUUUGGAUUUG	Cleavage		1
Hevea brasiliensis												
miRNA_Acc.	Target_Acc.	Expectation	UPE	miRNA_start	miRNA_end	Target_start	Target_end	miRNA_aligned_fragment	Target_aligned_fragment	Inhibition	Target_Desc.	Multiplicity
hbr-miR2118	PvAP2-ERF41	3	11,857	1	20	868	887	GAAAUGGGUGGAUGGGAGUG	CACCACCAACCACCCAUUUC	Cleavage		1
hbr-miR6170	PvAP2-ERF79	2	9,297	1	20	124	143	CAAGAAACAGAAGAGAGGGA	UCUCUCUUUUCUUUUUCUUG	Cleavage		1
hbr-miR6170	PvAP2-ERF139	3	13,475	1	21	210	230	CAAGAAACAGAAGAGAGGGAU	GUCUCUGUCUUUUUUUUCUUG	Cleavage		1
hbr-miR6170	PvAP2-ERF62	3	14,847	1	20	1255	1274	CAAGAAACAGAAGAGAGGGA	UUCUUCUUUUGUGUUUUUUG	Translation		1
hbr-miR6170	PvAP2-ERF133	2,5	1,829	1	20	5667	5686	CAAGAAACAGAAGAGAGGGA	UCAUUCUAUUCUGUUUCUUG	Cleavage		1
Heliconius melpomene												
miRNA_Acc.	Target_Acc.	Expectation	UPE	miRNA_start	miRNA_end	Target_start	Target_end	miRNA_aligned_fragment	Target_aligned_fragment	Inhibition	Target_Desc.	Multiplicity
hme-miR-14	PvAP2-ERF61	3	9,927	1	20	31	50	UCAGUCUUUUUCUCUCUCCU	AGGAGAGGGAAAGAGGGUGA	Cleavage		1
hme-miR-71	PvAP2-ERF147	3	17,578	1	21	371	391	UCUCACUACCUUGUCUUUCAU	AGGAAAUACAAGGGAGUGAGA	Cleavage		1
Lotus japonicus												
miRNA_Acc.	Target_Acc.	Expectation	UPE	miRNA_start	miRNA_end	Target_start	Target_end	miRNA_aligned_fragment	Target_aligned_fragment	Inhibition	Target_Desc.	Multiplicity
lja-miR172a	PvAP2-ERF173	0,5	11,33	1	21	3655	3675	AGAAUCUUGAUGAUGCUGCAG	CUGCAGCAUCAUCAGGAUUCU	Cleavage		1
lja-miR172a	PvAP2-ERF78	0,5	10,114	1	21	3796	3816	AGAAUCUUGAUGAUGCUGCAG	CUGCAGCAUCAUCAGGAUUCU	Cleavage		1
lja-miR172a	PvAP2-ERF20	1,5	9,626	1	21	2529	2549	AGAAUCUUGAUGAUGCUGCAG	CUGCAGCAUCAUCAGGAUUCC	Cleavage		1
lja-miR172a	PvAP2-ERF25	1,5	16,15	1	21	3063	3083	AGAAUCUUGAUGAUGCUGCAG	CUGCAGCAUCAUCAGGAUUCC	Cleavage		1
lja-miR172a	PvAP2-ERF62	1,5	16,463	1	21	3199	3219	AGAAUCUUGAUGAUGCUGCAG	CUGCAGCAUCAUCAGGAUUCC	Cleavage		1
lja-miR172a	PvAP2-ERF113	2,5	22,52	1	21	3571	3591	AGAAUCUUGAUGAUGCUGCAG	CAGCAGCAUCAUCAGGAUUCG	Cleavage		1
lja-miR172b	PvAP2-ERF173	0,5	11,33	1	20	3656	3675	AGAAUCUUGAUGAUGCUGCA	UGCAGCAUCAUCAGGAUUCU	Cleavage		1
lja-miR172b	PvAP2-ERF78	0,5	10,114	1	20	3797	3816	AGAAUCUUGAUGAUGCUGCA	UGCAGCAUCAUCAGGAUUCU	Cleavage		1
lja-miR172b	PvAP2-ERF20	1,5	9,626	1	20	2530	2549	AGAAUCUUGAUGAUGCUGCA	UGCAGCAUCAUCAGGAUUCC	Cleavage		1
lja-miR172b	PvAP2-ERF25	1,5	16,15	1	20	3064	3083	AGAAUCUUGAUGAUGCUGCA	UGCAGCAUCAUCAGGAUUCC	Cleavage		1
lja-miR172b	PvAP2-ERF62	1,5	16,463	1	20	3200	3219	AGAAUCUUGAUGAUGCUGCA	UGCAGCAUCAUCAGGAUUCC	Cleavage		1
lja-miR172b	PvAP2-ERF113	2,5	22,52	1	20	3572	3591	AGAAUCUUGAUGAUGCUGCA	AGCAGCAUCAUCAGGAUUCG	Cleavage		1
lja-miR172c	PvAP2-ERF78	0,5	10,114	1	20	3797	3816	AGAAUCUUGAUGAUGCUGCA	UGCAGCAUCAUCAGGAUUCU	Cleavage		1
lja-miR172c	PvAP2-ERF173	0,5	11,33	1	20	3656	3675	AGAAUCUUGAUGAUGCUGCA	UGCAGCAUCAUCAGGAUUCU	Cleavage		1
lja-miR172c	PvAP2-ERF62	1,5	16,463	1	20	3200	3219	AGAAUCUUGAUGAUGCUGCA	UGCAGCAUCAUCAGGAUUCC	Cleavage		1
lja-miR172c	PvAP2-ERF25	1,5	16,15	1	20	3064	3083	AGAAUCUUGAUGAUGCUGCA	UGCAGCAUCAUCAGGAUUCC	Cleavage		1
lja-miR172c	PvAP2-ERF20	1,5	9,626	1	20	2530	2549	AGAAUCUUGAUGAUGCUGCA	UGCAGCAUCAUCAGGAUUCC	Cleavage		1
lja-miR172c	PvAP2-ERF113	2,5	22,52	1	20	3572	3591	AGAAUCUUGAUGAUGCUGCA	AGCAGCAUCAUCAGGAUUCG	Cleavage		1
lja-miR7530	PvAP2-ERF62	3	10,441	1	21	56	76	CCUUCCUCUCUUCACUAUCUU	GAGAGAGAGAAGAAAGGAAGG	Cleavage		1
lja-miR7533a	PvAP2-ERF77	3	5,714	1	21	361	381	GAGGGGAUGGAGAGAAGCUGG	UCAGCUUCUCUUCUUCCCAUC	Cleavage		1
lja-miR7533b	PvAP2-ERF77	3	5,714	1	21	361	381	GAGGGGAUGGAGAGAAGCUGG	UCAGCUUCUCUUCUUCCCAUC	Cleavage		1
Malus domestica												
miRNA_Acc.	Target_Acc.	Expectation	UPE	miRNA_start	miRNA_end	Target_start	Target_end	miRNA_aligned_fragment	Target_aligned_fragment	Inhibition	Target_Desc.	Multiplicity
mdm-miR172a	PvAP2-ERF173	0,5	11,33	1	20	3656	3675	AGAAUCUUGAUGAUGCUGCA	UGCAGCAUCAUCAGGAUUCU	Cleavage		1
mdm-miR172a	PvAP2-ERF78	0,5	10,114	1	20	3797	3816	AGAAUCUUGAUGAUGCUGCA	UGCAGCAUCAUCAGGAUUCU	Cleavage		1
mdm-miR172a	PvAP2-ERF20	1,5	9,626	1	20	2530	2549	AGAAUCUUGAUGAUGCUGCA	UGCAGCAUCAUCAGGAUUCC	Cleavage		1
mdm-miR172a	PvAP2-ERF25	1,5	16,15	1	20	3064	3083	AGAAUCUUGAUGAUGCUGCA	UGCAGCAUCAUCAGGAUUCC	Cleavage		1
mdm-miR172a	PvAP2-ERF62	1,5	16,463	1	20	3200	3219	AGAAUCUUGAUGAUGCUGCA	UGCAGCAUCAUCAGGAUUCC	Cleavage		1
mdm-miR172a	PvAP2-ERF113	2,5	22,52	1	20	3572	3591	AGAAUCUUGAUGAUGCUGCA	AGCAGCAUCAUCAGGAUUCG	Cleavage		1
mdm-miR172b	PvAP2-ERF173	0,5	11,33	1	20	3656	3675	AGAAUCUUGAUGAUGCUGCA	UGCAGCAUCAUCAGGAUUCU	Cleavage		1
mdm-miR172b	PvAP2-ERF78	0,5	10,114	1	20	3797	3816	AGAAUCUUGAUGAUGCUGCA	UGCAGCAUCAUCAGGAUUCU	Cleavage		1
mdm-miR172b	PvAP2-ERF20	1,5	9,626	1	20	2530	2549	AGAAUCUUGAUGAUGCUGCA	UGCAGCAUCAUCAGGAUUCC	Cleavage		1
mdm-miR172b	PvAP2-ERF25	1,5	16,15	1	20	3064	3083	AGAAUCUUGAUGAUGCUGCA	UGCAGCAUCAUCAGGAUUCC	Cleavage		1
mdm-miR172b	PvAP2-ERF62	1,5	16,463	1	20	3200	3219	AGAAUCUUGAUGAUGCUGCA	UGCAGCAUCAUCAGGAUUCC	Cleavage		1
mdm-miR172b	PvAP2-ERF113	2,5	22,52	1	20	3572	3591	AGAAUCUUGAUGAUGCUGCA	AGCAGCAUCAUCAGGAUUCG	Cleavage		1

mdm-miR172c	PvAP2-ERF173	0,5	11,33	1	20	3656	3675	AGAAUCUUGAUGAUGCUGCA	UGCAGCAUCAUCAGGAUUCU	Cleavage	1
mdm-miR172c	PvAP2-ERF78	0,5	10,114	1	20	3797	3816	AGAAUCUUGAUGAUGCUGCA	UGCAGCAUCAUCAGGAUUCU	Cleavage	1
mdm-miR172c	PvAP2-ERF20	1,5	9,626	1	20	2530	2549	AGAAUCUUGAUGAUGCUGCA	UGCAGCAUCAUCAGGAUUCC	Cleavage	1
mdm-miR172c	PvAP2-ERF25	1,5	16,15	1	20	3064	3083	AGAAUCUUGAUGAUGCUGCA	UGCAGCAUCAUCAGGAUUCC	Cleavage	1
mdm-miR172c	PvAP2-ERF62	1,5	16,463	1	20	3200	3219	AGAAUCUUGAUGAUGCUGCA	UGCAGCAUCAUCAGGAUUCC	Cleavage	1
mdm-miR172c	PvAP2-ERF113	2,5	22,52	1	20	3572	3591	AGAAUCUUGAUGAUGCUGCA	AGCAGCAUCAUCAGGAUUCG	Cleavage	1
mdm-miR172d	PvAP2-ERF173	0,5	11,33	1	20	3656	3675	AGAAUCUUGAUGAUGCUGCA	UGCAGCAUCAUCAGGAUUCU	Cleavage	1
mdm-miR172d	PvAP2-ERF78	0,5	10,114	1	20	3797	3816	AGAAUCUUGAUGAUGCUGCA	UGCAGCAUCAUCAGGAUUCU	Cleavage	1
mdm-miR172d	PvAP2-ERF20	1,5	9,626	1	20	2530	2549	AGAAUCUUGAUGAUGCUGCA	UGCAGCAUCAUCAGGAUUCC	Cleavage	1
mdm-miR172d	PvAP2-ERF25	1,5	16,15	1	20	3064	3083	AGAAUCUUGAUGAUGCUGCA	UGCAGCAUCAUCAGGAUUCC	Cleavage	1
mdm-miR172d	PvAP2-ERF62	1,5	16,463	1	20	3200	3219	AGAAUCUUGAUGAUGCUGCA	UGCAGCAUCAUCAGGAUUCC	Cleavage	1
mdm-miR172d	PvAP2-ERF113	2,5	22,52	1	20	3572	3591	AGAAUCUUGAUGAUGCUGCA	AGCAGCAUCAUCAGGAUUCG	Cleavage	1
mdm-miR172e	PvAP2-ERF173	0,5	11,33	1	20	3656	3675	AGAAUCUUGAUGAUGCUGCA	UGCAGCAUCAUCAGGAUUCU	Cleavage	1
mdm-miR172e	PvAP2-ERF78	0,5	10,114	1	20	3797	3816	AGAAUCUUGAUGAUGCUGCA	UGCAGCAUCAUCAGGAUUCU	Cleavage	1
mdm-miR172e	PvAP2-ERF20	1,5	9,626	1	20	2530	2549	AGAAUCUUGAUGAUGCUGCA	UGCAGCAUCAUCAGGAUUCC	Cleavage	1
mdm-miR172e	PvAP2-ERF25	1,5	16,15	1	20	3064	3083	AGAAUCUUGAUGAUGCUGCA	UGCAGCAUCAUCAGGAUUCC	Cleavage	1
mdm-miR172e	PvAP2-ERF62	1,5	16,463	1	20	3200	3219	AGAAUCUUGAUGAUGCUGCA	UGCAGCAUCAUCAGGAUUCC	Cleavage	1
mdm-miR172e	PvAP2-ERF113	2,5	22,52	1	20	3572	3591	AGAAUCUUGAUGAUGCUGCA	AGCAGCAUCAUCAGGAUUCG	Cleavage	1
mdm-miR172f	PvAP2-ERF173	0,5	11,33	1	20	3656	3675	AGAAUCUUGAUGAUGCUGCA	UGCAGCAUCAUCAGGAUUCU	Cleavage	1
mdm-miR172f	PvAP2-ERF78	0,5	10,114	1	20	3797	3816	AGAAUCUUGAUGAUGCUGCA	UGCAGCAUCAUCAGGAUUCU	Cleavage	1
mdm-miR172f	PvAP2-ERF20	1,5	9,626	1	20	2530	2549	AGAAUCUUGAUGAUGCUGCA	UGCAGCAUCAUCAGGAUUCC	Cleavage	1
mdm-miR172f	PvAP2-ERF25	1,5	16,15	1	20	3064	3083	AGAAUCUUGAUGAUGCUGCA	UGCAGCAUCAUCAGGAUUCC	Cleavage	1
mdm-miR172f	PvAP2-ERF62	1,5	16,463	1	20	3200	3219	AGAAUCUUGAUGAUGCUGCA	UGCAGCAUCAUCAGGAUUCC	Cleavage	1
mdm-miR172f	PvAP2-ERF113	2,5	22,52	1	20	3572	3591	AGAAUCUUGAUGAUGCUGCA	AGCAGCAUCAUCAGGAUUCG	Cleavage	1
mdm-miR172g	PvAP2-ERF173	0,5	11,33	1	20	3656	3675	AGAAUCUUGAUGAUGCUGCA	UGCAGCAUCAUCAGGAUUCU	Cleavage	1
mdm-miR172g	PvAP2-ERF78	0,5	10,114	1	20	3797	3816	AGAAUCUUGAUGAUGCUGCA	UGCAGCAUCAUCAGGAUUCU	Cleavage	1
mdm-miR172g	PvAP2-ERF20	1,5	9,626	1	20	2530	2549	AGAAUCUUGAUGAUGCUGCA	UGCAGCAUCAUCAGGAUUCC	Cleavage	1
mdm-miR172g	PvAP2-ERF25	1,5	16,15	1	20	3064	3083	AGAAUCUUGAUGAUGCUGCA	UGCAGCAUCAUCAGGAUUCC	Cleavage	1
mdm-miR172g	PvAP2-ERF62	1,5	16,463	1	20	3200	3219	AGAAUCUUGAUGAUGCUGCA	UGCAGCAUCAUCAGGAUUCC	Cleavage	1
mdm-miR172g	PvAP2-ERF113	2,5	22,52	1	20	3572	3591	AGAAUCUUGAUGAUGCUGCA	AGCAGCAUCAUCAGGAUUCG	Cleavage	1
mdm-miR172h	PvAP2-ERF173	0,5	11,33	1	20	3656	3675	AGAAUCUUGAUGAUGCUGCA	UGCAGCAUCAUCAGGAUUCU	Cleavage	1
mdm-miR172h	PvAP2-ERF78	0,5	10,114	1	20	3797	3816	AGAAUCUUGAUGAUGCUGCA	UGCAGCAUCAUCAGGAUUCU	Cleavage	1
mdm-miR172h	PvAP2-ERF20	1,5	9,626	1	20	2530	2549	AGAAUCUUGAUGAUGCUGCA	UGCAGCAUCAUCAGGAUUCC	Cleavage	1
mdm-miR172h	PvAP2-ERF25	1,5	16,15	1	20	3064	3083	AGAAUCUUGAUGAUGCUGCA	UGCAGCAUCAUCAGGAUUCC	Cleavage	1
mdm-miR172h	PvAP2-ERF62	1,5	16,463	1	20	3200	3219	AGAAUCUUGAUGAUGCUGCA	UGCAGCAUCAUCAGGAUUCC	Cleavage	1
mdm-miR172h	PvAP2-ERF113	2,5	22,52	1	20	3572	3591	AGAAUCUUGAUGAUGCUGCA	AGCAGCAUCAUCAGGAUUCG	Cleavage	1
mdm-miR172i	PvAP2-ERF20	0,5	9,626	1	20	2530	2549	GGAAUCUUGAUGAUGCUGCA	UGCAGCAUCAUCAGGAUUCC	Cleavage	1
mdm-miR172i	PvAP2-ERF25	0,5	16,15	1	20	3064	3083	GGAAUCUUGAUGAUGCUGCA	UGCAGCAUCAUCAGGAUUCC	Cleavage	1
mdm-miR172i	PvAP2-ERF62	0,5	16,463	1	20	3200	3219	GGAAUCUUGAUGAUGCUGCA	UGCAGCAUCAUCAGGAUUCC	Cleavage	1
mdm-miR172i	PvAP2-ERF173	1	11,33	1	20	3656	3675	GGAAUCUUGAUGAUGCUGCA	UGCAGCAUCAUCAGGAUUCU	Cleavage	1
mdm-miR172i	PvAP2-ERF78	1	10,114	1	20	3797	3816	GGAAUCUUGAUGAUGCUGCA	UGCAGCAUCAUCAGGAUUCU	Cleavage	1
mdm-miR172i	PvAP2-ERF113	2,5	22,52	1	20	3572	3591	GGAAUCUUGAUGAUGCUGCA	AGCAGCAUCAUCAGGAUUCG	Cleavage	1
mdm-miR172j	PvAP2-ERF20	0,5	9,626	1	20	2530	2549	GGAAUCUUGAUGAUGCUGCA	UGCAGCAUCAUCAGGAUUCC	Cleavage	1
mdm-miR172j	PvAP2-ERF25	0,5	16,15	1	20	3064	3083	GGAAUCUUGAUGAUGCUGCA	UGCAGCAUCAUCAGGAUUCC	Cleavage	1
mdm-miR172j	PvAP2-ERF62	0,5	16,463	1	20	3200	3219	GGAAUCUUGAUGAUGCUGCA	UGCAGCAUCAUCAGGAUUCC	Cleavage	1
mdm-miR172j	PvAP2-ERF173	1	11,33	1	20	3656	3675	GGAAUCUUGAUGAUGCUGCA	UGCAGCAUCAUCAGGAUUCU	Cleavage	1
mdm-miR172j	PvAP2-ERF78	1	10,114	1	20	3797	3816	GGAAUCUUGAUGAUGCUGCA	UGCAGCAUCAUCAGGAUUCU	Cleavage	1
mdm-miR172j	PvAP2-ERF113	2,5	22,52	1	20	3572	3591	GGAAUCUUGAUGAUGCUGCA	AGCAGCAUCAUCAGGAUUCG	Cleavage	1
mdm-miR172k	PvAP2-ERF20	0,5	9,626	1	20	2530	2549	GGAAUCUUGAUGAUGCUGCA	UGCAGCAUCAUCAGGAUUCC	Cleavage	1
mdm-miR172k	PvAP2-ERF25	0,5	16,15	1	20	3064	3083	GGAAUCUUGAUGAUGCUGCA	UGCAGCAUCAUCAGGAUUCC	Cleavage	1
mdm-miR172k	PvAP2-ERF62	0,5	16,463	1	20	3200	3219	GGAAUCUUGAUGAUGCUGCA	UGCAGCAUCAUCAGGAUUCC	Cleavage	1
mdm-miR172k	PvAP2-ERF173	1	11,33	1	20	3656	3675	GGAAUCUUGAUGAUGCUGCA	UGCAGCAUCAUCAGGAUUCU	Cleavage	1
mdm-miR172k	PvAP2-ERF78	1	10,114	1	20	3797	3816	GGAAUCUUGAUGAUGCUGCA	UGCAGCAUCAUCAGGAUUCU	Cleavage	1
mdm-miR172k	PvAP2-ERF113	2,5	22,52	1	20	3572	3591	GGAAUCUUGAUGAUGCUGCA	AGCAGCAUCAUCAGGAUUCG	Cleavage	1
mdm-miR172l	PvAP2-ERF20	0,5	9,626	1	21	2529	2549	GGAAUCUUGAUGAUGCUGCAG	CUGCAGCAUCAUCAGGAUUCC	Cleavage	1

mdm-miR172l	PvAP2-ERF25	0,5	16,15	1	21	3063	3083	GGAAUCUUGAUGAUGCUGCAG	CUGCAGCAUCAUCAGGAUUCC	Cleavage		1
mdm-miR172l	PvAP2-ERF62	0,5	16,463	1	21	3199	3219	GGAAUCUUGAUGAUGCUGCAG	CUGCAGCAUCAUCAGGAUUCC	Cleavage		1
mdm-miR172l	PvAP2-ERF173	1	11,33	1	21	3655	3675	GGAAUCUUGAUGAUGCUGCAG	CUGCAGCAUCAUCAGGAUUCU	Cleavage		1
mdm-miR172l	PvAP2-ERF78	1	10,114	1	21	3796	3816	GGAAUCUUGAUGAUGCUGCAG	CUGCAGCAUCAUCAGGAUUCU	Cleavage		1
mdm-miR172l	PvAP2-ERF113	2,5	22,52	1	21	3571	3591	GGAAUCUUGAUGAUGCUGCAG	CAGCAGCAUCAUCAGGAUUCG	Cleavage		1
mdm-miR172m	PvAP2-ERF173	0,5	11,33	1	21	3655	3675	AGAAUCUUGAUGAUGCUGCAG	CUGCAGCAUCAUCAGGAUUCU	Cleavage		1
mdm-miR172m	PvAP2-ERF78	0,5	10,114	1	21	3796	3816	AGAAUCUUGAUGAUGCUGCAG	CUGCAGCAUCAUCAGGAUUCU	Cleavage		1
mdm-miR172m	PvAP2-ERF20	1,5	9,626	1	21	2529	2549	AGAAUCUUGAUGAUGCUGCAG	CUGCAGCAUCAUCAGGAUUCC	Cleavage		1
mdm-miR172m	PvAP2-ERF25	1,5	16,15	1	21	3063	3083	AGAAUCUUGAUGAUGCUGCAG	CUGCAGCAUCAUCAGGAUUCC	Cleavage		1
mdm-miR172m	PvAP2-ERF62	1,5	16,463	1	21	3199	3219	AGAAUCUUGAUGAUGCUGCAG	CUGCAGCAUCAUCAGGAUUCC	Cleavage		1
mdm-miR172m	PvAP2-ERF113	2,5	22,52	1	21	3571	3591	AGAAUCUUGAUGAUGCUGCAG	CAGCAGCAUCAUCAGGAUUCG	Cleavage		1
mdm-miR172n	PvAP2-ERF173	0,5	11,33	1	21	3655	3675	AGAAUCUUGAUGAUGCUGCAG	CUGCAGCAUCAUCAGGAUUCU	Cleavage		1
mdm-miR172n	PvAP2-ERF78	0,5	10,114	1	21	3796	3816	AGAAUCUUGAUGAUGCUGCAG	CUGCAGCAUCAUCAGGAUUCU	Cleavage		1
mdm-miR172n	PvAP2-ERF20	1,5	9,626	1	21	2529	2549	AGAAUCUUGAUGAUGCUGCAG	CUGCAGCAUCAUCAGGAUUCC	Cleavage		1
mdm-miR172n	PvAP2-ERF25	1,5	16,15	1	21	3063	3083	AGAAUCUUGAUGAUGCUGCAG	CUGCAGCAUCAUCAGGAUUCC	Cleavage		1
mdm-miR172n	PvAP2-ERF62	1,5	16,463	1	21	3199	3219	AGAAUCUUGAUGAUGCUGCAG	CUGCAGCAUCAUCAGGAUUCC	Cleavage		1
mdm-miR172n	PvAP2-ERF113	2,5	22,52	1	21	3571	3591	AGAAUCUUGAUGAUGCUGCAG	CAGCAGCAUCAUCAGGAUUCG	Cleavage		1
mdm-miR172o	PvAP2-ERF173	0,5	11,33	1	21	3655	3675	AGAAUCUUGAUGAUGCUGCAG	CUGCAGCAUCAUCAGGAUUCU	Cleavage		1
mdm-miR172o	PvAP2-ERF78	0,5	10,114	1	21	3796	3816	AGAAUCUUGAUGAUGCUGCAG	CUGCAGCAUCAUCAGGAUUCU	Cleavage		1
mdm-miR172o	PvAP2-ERF20	1,5	9,626	1	21	2529	2549	AGAAUCUUGAUGAUGCUGCAG	CUGCAGCAUCAUCAGGAUUCC	Cleavage		1
mdm-miR172o	PvAP2-ERF25	1,5	16,15	1	21	3063	3083	AGAAUCUUGAUGAUGCUGCAG	CUGCAGCAUCAUCAGGAUUCC	Cleavage		1
mdm-miR172o	PvAP2-ERF62	1,5	16,463	1	21	3199	3219	AGAAUCUUGAUGAUGCUGCAG	CUGCAGCAUCAUCAGGAUUCC	Cleavage		1
mdm-miR172o	PvAP2-ERF113	2,5	22,52	1	21	3571	3591	AGAAUCUUGAUGAUGCUGCAG	CAGCAGCAUCAUCAGGAUUCG	Cleavage		1
Manihot esculenta												
miRNA_Acc.	Target_Acc.	Expectation	UPE	miRNA_start	miRNA_end	Target_start	Target_end	miRNA_aligned_fragment	Target_aligned_fragment	Inhibition	Target_Desc.	Multiplicity
mes-miR172a	PvAP2-ERF173	0,5	11,33	1	20	3656	3675	AGAAUCUUGAUGAUGCUGCA	UGCAGCAUCAUCAGGAUUCU	Cleavage		1
mes-miR172a	PvAP2-ERF78	0,5	10,114	1	20	3797	3816	AGAAUCUUGAUGAUGCUGCA	UGCAGCAUCAUCAGGAUUCU	Cleavage		1
mes-miR172a	PvAP2-ERF20	1,5	9,626	1	20	2530	2549	AGAAUCUUGAUGAUGCUGCA	UGCAGCAUCAUCAGGAUUCC	Cleavage		1
mes-miR172a	PvAP2-ERF25	1,5	16,15	1	20	3064	3083	AGAAUCUUGAUGAUGCUGCA	UGCAGCAUCAUCAGGAUUCC	Cleavage		1
mes-miR172a	PvAP2-ERF62	1,5	16,463	1	20	3200	3219	AGAAUCUUGAUGAUGCUGCA	UGCAGCAUCAUCAGGAUUCC	Cleavage		1
mes-miR172a	PvAP2-ERF113	2,5	22,52	1	20	3572	3591	AGAAUCUUGAUGAUGCUGCA	AGCAGCAUCAUCAGGAUUCG	Cleavage		1
mes-miR172b	PvAP2-ERF173	0,5	11,33	1	20	3656	3675	AGAAUCUUGAUGAUGCUGCA	UGCAGCAUCAUCAGGAUUCU	Cleavage		1
mes-miR172b	PvAP2-ERF78	0,5	10,114	1	20	3797	3816	AGAAUCUUGAUGAUGCUGCA	UGCAGCAUCAUCAGGAUUCU	Cleavage		1
mes-miR172b	PvAP2-ERF20	1,5	9,626	1	20	2530	2549	AGAAUCUUGAUGAUGCUGCA	UGCAGCAUCAUCAGGAUUCC	Cleavage		1
mes-miR172b	PvAP2-ERF25	1,5	16,15	1	20	3064	3083	AGAAUCUUGAUGAUGCUGCA	UGCAGCAUCAUCAGGAUUCC	Cleavage		1
mes-miR172b	PvAP2-ERF62	1,5	16,463	1	20	3200	3219	AGAAUCUUGAUGAUGCUGCA	UGCAGCAUCAUCAGGAUUCC	Cleavage		1
mes-miR172b	PvAP2-ERF113	2,5	22,52	1	20	3572	3591	AGAAUCUUGAUGAUGCUGCA	AGCAGCAUCAUCAGGAUUCG	Cleavage		1
mes-miR172c	PvAP2-ERF20	3	9,626	1	20	2530	2549	UGAAUCUUGAUGAUGCUACG	UGCAGCAUCAUCAGGAUUCC	Cleavage		1
mes-miR172c	PvAP2-ERF25	3	16,15	1	20	3064	3083	UGAAUCUUGAUGAUGCUACG	UGCAGCAUCAUCAGGAUUCC	Cleavage		1
mes-miR172c	PvAP2-ERF62	3	16,463	1	20	3200	3219	UGAAUCUUGAUGAUGCUACG	UGCAGCAUCAUCAGGAUUCC	Cleavage		1
mes-miR172c	PvAP2-ERF113	3	22,52	1	20	3572	3591	UGAAUCUUGAUGAUGCUACG	AGCAGCAUCAUCAGGAUUCG	Cleavage		1
mes-miR172c	PvAP2-ERF173	3	11,33	1	20	3656	3675	UGAAUCUUGAUGAUGCUACG	UGCAGCAUCAUCAGGAUUCU	Cleavage		1
mes-miR172c	PvAP2-ERF78	3	10,114	1	20	3797	3816	UGAAUCUUGAUGAUGCUACG	UGCAGCAUCAUCAGGAUUCU	Cleavage		1
mes-miR172d	PvAP2-ERF173	0,5	11,33	1	20	3656	3675	AGAAUCUUGAUGAUGCUGCA	UGCAGCAUCAUCAGGAUUCU	Cleavage		1
mes-miR172d	PvAP2-ERF78	0,5	10,114	1	20	3797	3816	AGAAUCUUGAUGAUGCUGCA	UGCAGCAUCAUCAGGAUUCU	Cleavage		1
mes-miR172d	PvAP2-ERF20	1,5	9,626	1	20	2530	2549	AGAAUCUUGAUGAUGCUGCA	UGCAGCAUCAUCAGGAUUCC	Cleavage		1
mes-miR172d	PvAP2-ERF25	1,5	16,15	1	20	3064	3083	AGAAUCUUGAUGAUGCUGCA	UGCAGCAUCAUCAGGAUUCC	Cleavage		1
mes-miR172d	PvAP2-ERF62	1,5	16,463	1	20	3200	3219	AGAAUCUUGAUGAUGCUGCA	UGCAGCAUCAUCAGGAUUCC	Cleavage		1
mes-miR172d	PvAP2-ERF113	2,5	22,52	1	20	3572	3591	AGAAUCUUGAUGAUGCUGCA	AGCAGCAUCAUCAGGAUUCG	Cleavage		1
mes-miR172e	PvAP2-ERF20	0,5	9,626	1	21	2529	2549	GGAAUCUUGAUGAUGCUGCAG	CUGCAGCAUCAUCAGGAUUCC	Cleavage		1
mes-miR172e	PvAP2-ERF25	0,5	16,15	1	21	3063	3083	GGAAUCUUGAUGAUGCUGCAG	CUGCAGCAUCAUCAGGAUUCC	Cleavage		1
mes-miR172e	PvAP2-ERF62	0,5	16,463	1	21	3199	3219	GGAAUCUUGAUGAUGCUGCAG	CUGCAGCAUCAUCAGGAUUCC	Cleavage		1
mes-miR172e	PvAP2-ERF173	1	11,33	1	21	3655	3675	GGAAUCUUGAUGAUGCUGCAG	CUGCAGCAUCAUCAGGAUUCU	Cleavage		1
mes-miR172e	PvAP2-ERF78	1	10,114	1	21	3796	3816	GGAAUCUUGAUGAUGCUGCAG	CUGCAGCAUCAUCAGGAUUCU	Cleavage		1
mes-miR172e	PvAP2-ERF113	2,5	22,52	1	21	3571	3591	GGAAUCUUGAUGAUGCUGCAG	CAGCAGCAUCAUCAGGAUUCG	Cleavage		1

mes-miR172f	PvAP2-ERF20	0,5	9,626	1	21	2529	2549	GGAAUCUUGAUGAUGCUGCAG	CUGCAGCAUCAUCAGGAUUCC	Cleavage		1
mes-miR172f	PvAP2-ERF25	0,5	16,15	1	21	3063	3083	GGAAUCUUGAUGAUGCUGCAG	CUGCAGCAUCAUCAGGAUUCC	Cleavage		1
mes-miR172f	PvAP2-ERF62	0,5	16,463	1	21	3199	3219	GGAAUCUUGAUGAUGCUGCAG	CUGCAGCAUCAUCAGGAUUCC	Cleavage		1
mes-miR172f	PvAP2-ERF173	1	11,33	1	21	3655	3675	GGAAUCUUGAUGAUGCUGCAG	CUGCAGCAUCAUCAGGAUUCU	Cleavage		1
mes-miR172f	PvAP2-ERF78	1	10,114	1	21	3796	3816	GGAAUCUUGAUGAUGCUGCAG	CUGCAGCAUCAUCAGGAUUCU	Cleavage		1
mes-miR172f	PvAP2-ERF113	2,5	22,52	1	21	3571	3591	GGAAUCUUGAUGAUGCUGCAG	CAGCAGCAUCAUCAGGAUUCG	Cleavage		1
mes-miR2275	PvAP2-ERF7	1	16,862	1	22	1455	1476	UUUGGUUUCCUCCAAUAUCUUA	UGAGAAAUUGGAGGAAACCAAA	Cleavage		1
mes-miR395e	PvAP2-ERF179	3	11,026	1	20	4356	4375	CUGAAGGGUUUGGAGGAACU	AGUUCCUCCUAACUCCUCAG	Translation		1

Medicago truncatula

miRNA_Acc.	Target_Acc.	Expectation	UPE	miRNA_start	miRNA_end	Target_start	Target_end	miRNA_aligned_fragment	Target_aligned_fragment	Inhibition	Target_Desc.	Multiplicity
mtr-miR172a	PvAP2-ERF173	0	11,33	1	21	3655	3675	AGAAUCCUGAUGAUGCUGCAG	CUGCAGCAUCAUCAGGAUUCU	Cleavage		1
mtr-miR172a	PvAP2-ERF78	0	10,114	1	21	3796	3816	AGAAUCCUGAUGAUGCUGCAG	CUGCAGCAUCAUCAGGAUUCU	Cleavage		1
mtr-miR172a	PvAP2-ERF20	1	9,626	1	21	2529	2549	AGAAUCCUGAUGAUGCUGCAG	CUGCAGCAUCAUCAGGAUUCC	Cleavage		1
mtr-miR172a	PvAP2-ERF25	1	16,15	1	21	3063	3083	AGAAUCCUGAUGAUGCUGCAG	CUGCAGCAUCAUCAGGAUUCC	Cleavage		1
mtr-miR172a	PvAP2-ERF62	1	16,463	1	21	3199	3219	AGAAUCCUGAUGAUGCUGCAG	CUGCAGCAUCAUCAGGAUUCC	Cleavage		1
mtr-miR172a	PvAP2-ERF113	2	22,52	1	21	3571	3591	AGAAUCCUGAUGAUGCUGCAG	CAGCAGCAUCAUCAGGAUUCG	Cleavage		1
mtr-miR172b	PvAP2-ERF173	0,5	11,33	1	20	3656	3675	AGAAUCUUGAUGAUGCUGCA	UGCAGCAUCAUCAGGAUUCU	Cleavage		1
mtr-miR172b	PvAP2-ERF78	0,5	10,114	1	20	3797	3816	AGAAUCUUGAUGAUGCUGCA	UGCAGCAUCAUCAGGAUUCU	Cleavage		1
mtr-miR172b	PvAP2-ERF20	1,5	9,626	1	20	2530	2549	AGAAUCUUGAUGAUGCUGCA	UGCAGCAUCAUCAGGAUUCC	Cleavage		1
mtr-miR172b	PvAP2-ERF25	1,5	16,15	1	20	3064	3083	AGAAUCUUGAUGAUGCUGCA	UGCAGCAUCAUCAGGAUUCC	Cleavage		1
mtr-miR172b	PvAP2-ERF62	1,5	16,463	1	20	3200	3219	AGAAUCUUGAUGAUGCUGCA	UGCAGCAUCAUCAGGAUUCC	Cleavage		1
mtr-miR172b	PvAP2-ERF113	2,5	22,52	1	20	3572	3591	AGAAUCUUGAUGAUGCUGCA	AGCAGCAUCAUCAGGAUUCG	Cleavage		1
mtr-miR172c-3p	PvAP2-ERF173	0,5	11,33	1	20	3656	3675	AGAAUCUUGAUGAUGCUGCA	UGCAGCAUCAUCAGGAUUCU	Cleavage		1
mtr-miR172c-3p	PvAP2-ERF78	0,5	10,114	1	20	3797	3816	AGAAUCUUGAUGAUGCUGCA	UGCAGCAUCAUCAGGAUUCU	Cleavage		1
mtr-miR172c-3p	PvAP2-ERF20	1,5	9,626	1	20	2530	2549	AGAAUCUUGAUGAUGCUGCA	UGCAGCAUCAUCAGGAUUCC	Cleavage		1
mtr-miR172c-3p	PvAP2-ERF25	1,5	16,15	1	20	3064	3083	AGAAUCUUGAUGAUGCUGCA	UGCAGCAUCAUCAGGAUUCC	Cleavage		1
mtr-miR172c-3p	PvAP2-ERF62	1,5	16,463	1	20	3200	3219	AGAAUCUUGAUGAUGCUGCA	UGCAGCAUCAUCAGGAUUCC	Cleavage		1
mtr-miR172c-3p	PvAP2-ERF113	2,5	22,52	1	20	3572	3591	AGAAUCUUGAUGAUGCUGCA	AGCAGCAUCAUCAGGAUUCG	Cleavage		1
mtr-miR172d-3p	PvAP2-ERF173	0,5	11,33	1	20	3656	3675	AGAAUCUUGAUGAUGCUGCA	UGCAGCAUCAUCAGGAUUCU	Cleavage		1
mtr-miR172d-3p	PvAP2-ERF78	0,5	10,114	1	20	3797	3816	AGAAUCUUGAUGAUGCUGCA	UGCAGCAUCAUCAGGAUUCU	Cleavage		1
mtr-miR172d-3p	PvAP2-ERF20	1,5	9,626	1	20	2530	2549	AGAAUCUUGAUGAUGCUGCA	UGCAGCAUCAUCAGGAUUCC	Cleavage		1
mtr-miR172d-3p	PvAP2-ERF25	1,5	16,15	1	20	3064	3083	AGAAUCUUGAUGAUGCUGCA	UGCAGCAUCAUCAGGAUUCC	Cleavage		1
mtr-miR172d-3p	PvAP2-ERF62	1,5	16,463	1	20	3200	3219	AGAAUCUUGAUGAUGCUGCA	UGCAGCAUCAUCAGGAUUCC	Cleavage		1
mtr-miR172d-3p	PvAP2-ERF113	2,5	22,52	1	20	3572	3591	AGAAUCUUGAUGAUGCUGCA	AGCAGCAUCAUCAGGAUUCG	Cleavage		1
mtr-miR2607	PvAP2-ERF69	2,5	9,46	1	20	1326	1345	AUGUGAUUAUGUGAUAAGUG	CAUUAGUCACAUAGUCACAU	Cleavage		1
mtr-miR2619a	PvAP2-ERF130	3	13,185	1	20	662	681	ACAUAGGAGGCUGUUUUGUA	AACAAAACAGCAUCCUAUGC	Translation		1
mtr-miR2643b-5p	PvAP2-ERF154	2	16,066	1	20	71	90	UCUAAUCUCUGUUCCCAAUU	AGUUUGGAGCAGAGAUUAGA	Cleavage		1
mtr-miR2643b-5p	PvAP2-ERF103	3	12,902	1	20	740	759	UCUAAUCUCUGUUCCCAAUU	AAUGGGUGGCAGAGAUUAGA	Cleavage		1
mtr-miR2673a	PvAP2-ERF62	2	14,892	1	22	648	669	CCUCUUCCUCUUCCUCUUCCAC	GAGGAGGAGGAGGACGAAGAGG	Cleavage		1
mtr-miR2673a	PvAP2-ERF46	3	22,007	1	21	704	724	CCUCUUCCUCUUCCUCUUCCA	UGAAGGAGGAAGAGGAGGAGA	Cleavage		1
mtr-miR2673a	PvAP2-ERF75	3	17,63	1	20	1361	1380	CCUCUUCCUCUUCCUCUUCC	AGAAGAAGAAGAAGAAGAGG	Cleavage		1
mtr-miR2673b	PvAP2-ERF62	2	14,892	1	22	648	669	CCUCUUCCUCUUCCUCUUCCAC	GAGGAGGAGGAGGACGAAGAGG	Cleavage		1
mtr-miR2673b	PvAP2-ERF46	3	22,007	1	21	704	724	CCUCUUCCUCUUCCUCUUCCA	UGAAGGAGGAAGAGGAGGAGA	Cleavage		1
mtr-miR2673b	PvAP2-ERF75	3	17,63	1	20	1361	1380	CCUCUUCCUCUUCCUCUUCC	AGAAGAAGAAGAAGAAGAGG	Cleavage		1
mtr-miR2678	PvAP2-ERF39	3	11,144	1	20	228	247	UGAAAUUGUUGCGAGUGUCU	AAACACUCGCACCAAUUUCU	Translation		1
mtr-miR396c	PvAP2-ERF155	3	20,817	1	21	314	334	AUUCAAGAAGGUCGUGGAAAA	UUUUGGAAGACCUUCUUGAAU	Cleavage		1
mtr-miR5208d	PvAP2-ERF150	3	13,045	1	24	998	1021	CAUAUUAGUCAUAUUUGUAGGCAU	AGGACUACAAAUAUUACUAGUGUU	Translation		1
mtr-miR5210	PvAP2-ERF112	3	14,819	1	20	808	827	UAAAUGUGUUGGAAUUAAGG	CCUUAAUGUCAAUGUAUUUA	Cleavage		1
mtr-miR5241a	PvAP2-ERF23	3	19,601	1	20	2573	2592	UGACUGAAUGGAAGAGUGCA	UGAACUUUUCUUUUCAGUCA	Translation		1
mtr-miR5241b	PvAP2-ERF23	3	19,601	1	20	2573	2592	UGACUGAAUGGAAGAGUGCA	UGAACUUUUCUUUUCAGUCA	Translation		1
mtr-miR5241c	PvAP2-ERF23	3	19,601	1	20	2573	2592	UGACUGAAUGGAAGAGUGCA	UGAACUUUUCUUUUCAGUCA	Translation		1
mtr-miR5298b	PvAP2-ERF56	3	13,323	1	19	5	24	UGAUGGAGAUGAU-AUGAAG	CUUCAUCGUCAUCUUCAUCA	Cleavage		1
mtr-miR5298c	PvAP2-ERF56	3	13,323	1	19	5	24	UGAUGGAGAUGAU-AUGAAG	CUUCAUCGUCAUCUUCAUCA	Cleavage		1
mtr-miR5558-5p	PvAP2-ERF126	3	9,46	1	20	1196	1215	UUUUCCAAUUCUAAGUCUAU	AUAGACAGAGAAUUGGAGAG	Cleavage		1
mtr-miR5749	PvAP2-ERF32	3	11,302	1	20	2637	2656	UUCGGGUUGAUAAUAUUUUC	AAAAAUUUUAUUAACCUGAA	Cleavage		1

mtr-miR5750	PvAP2-ERF105	3	18,55	1	22	123	144	AAGAGAGAUAGAUCAGAAUUGA	UGGAUUUUGAUUCAUCUUUCUU	Translation		1

Nicotiana tabacum

miRNA_Acc.	Target_Acc.	Expectation	UPE	miRNA_start	miRNA_end	Target_start	Target_end	miRNA_aligned_fragment	Target_aligned_fragment	Inhibition	Target_Desc.	Multiplicity
nta-miR172a	PvAP2-ERF173	0,5	11,33	1	21	3655	3675	AGAAUCUUGAUGAUGCUGCAG	CUGCAGCAUCAUCAGGAUUCU	Cleavage		1
nta-miR172a	PvAP2-ERF78	0,5	10,114	1	21	3796	3816	AGAAUCUUGAUGAUGCUGCAG	CUGCAGCAUCAUCAGGAUUCU	Cleavage		1
nta-miR172a	PvAP2-ERF20	1,5	9,626	1	21	2529	2549	AGAAUCUUGAUGAUGCUGCAG	CUGCAGCAUCAUCAGGAUUCC	Cleavage		1
nta-miR172a	PvAP2-ERF25	1,5	16,15	1	21	3063	3083	AGAAUCUUGAUGAUGCUGCAG	CUGCAGCAUCAUCAGGAUUCC	Cleavage		1
nta-miR172a	PvAP2-ERF62	1,5	16,463	1	21	3199	3219	AGAAUCUUGAUGAUGCUGCAG	CUGCAGCAUCAUCAGGAUUCC	Cleavage		1
nta-miR172a	PvAP2-ERF113	2,5	22,52	1	21	3571	3591	AGAAUCUUGAUGAUGCUGCAG	CAGCAGCAUCAUCAGGAUUCG	Cleavage		1
nta-miR172b	PvAP2-ERF173	1,5	11,33	1	20	3656	3675	AGAAUCAUGAUGAUGCUGCA	UGCAGCAUCAUCAGGAUUCU	Cleavage		1
nta-miR172b	PvAP2-ERF78	1,5	10,114	1	20	3797	3816	AGAAUCAUGAUGAUGCUGCA	UGCAGCAUCAUCAGGAUUCU	Cleavage		1
nta-miR172b	PvAP2-ERF20	2,5	9,626	1	20	2530	2549	AGAAUCAUGAUGAUGCUGCA	UGCAGCAUCAUCAGGAUUCC	Cleavage		1
nta-miR172b	PvAP2-ERF25	2,5	16,15	1	20	3064	3083	AGAAUCAUGAUGAUGCUGCA	UGCAGCAUCAUCAGGAUUCC	Cleavage		1
nta-miR172b	PvAP2-ERF62	2,5	16,463	1	20	3200	3219	AGAAUCAUGAUGAUGCUGCA	UGCAGCAUCAUCAGGAUUCC	Cleavage		1
nta-miR172c	PvAP2-ERF173	0,5	11,33	1	20	3656	3675	AGAAUCUUGAUGAUGCUGCA	UGCAGCAUCAUCAGGAUUCU	Cleavage		1
nta-miR172c	PvAP2-ERF78	0,5	10,114	1	20	3797	3816	AGAAUCUUGAUGAUGCUGCA	UGCAGCAUCAUCAGGAUUCU	Cleavage		1
nta-miR172c	PvAP2-ERF20	1,5	9,626	1	20	2530	2549	AGAAUCUUGAUGAUGCUGCA	UGCAGCAUCAUCAGGAUUCC	Cleavage		1
nta-miR172c	PvAP2-ERF25	1,5	16,15	1	20	3064	3083	AGAAUCUUGAUGAUGCUGCA	UGCAGCAUCAUCAGGAUUCC	Cleavage		1
nta-miR172c	PvAP2-ERF62	1,5	16,463	1	20	3200	3219	AGAAUCUUGAUGAUGCUGCA	UGCAGCAUCAUCAGGAUUCC	Cleavage		1
nta-miR172c	PvAP2-ERF113	2,5	22,52	1	20	3572	3591	AGAAUCUUGAUGAUGCUGCA	AGCAGCAUCAUCAGGAUUCG	Cleavage		1
nta-miR172d	PvAP2-ERF173	0,5	11,33	1	20	3656	3675	AGAAUCUUGAUGAUGCUGCA	UGCAGCAUCAUCAGGAUUCU	Cleavage		1
nta-miR172d	PvAP2-ERF78	0,5	10,114	1	20	3797	3816	AGAAUCUUGAUGAUGCUGCA	UGCAGCAUCAUCAGGAUUCU	Cleavage		1
nta-miR172d	PvAP2-ERF20	1,5	9,626	1	20	2530	2549	AGAAUCUUGAUGAUGCUGCA	UGCAGCAUCAUCAGGAUUCC	Cleavage		1
nta-miR172d	PvAP2-ERF25	1,5	16,15	1	20	3064	3083	AGAAUCUUGAUGAUGCUGCA	UGCAGCAUCAUCAGGAUUCC	Cleavage		1
nta-miR172d	PvAP2-ERF62	1,5	16,463	1	20	3200	3219	AGAAUCUUGAUGAUGCUGCA	UGCAGCAUCAUCAGGAUUCC	Cleavage		1
nta-miR172d	PvAP2-ERF113	2,5	22,52	1	20	3572	3591	AGAAUCUUGAUGAUGCUGCA	AGCAGCAUCAUCAGGAUUCG	Cleavage		1
nta-miR172e	PvAP2-ERF173	0,5	11,33	1	20	3656	3675	AGAAUCUUGAUGAUGCUGCA	UGCAGCAUCAUCAGGAUUCU	Cleavage		1
nta-miR172e	PvAP2-ERF78	0,5	10,114	1	20	3797	3816	AGAAUCUUGAUGAUGCUGCA	UGCAGCAUCAUCAGGAUUCU	Cleavage		1
nta-miR172e	PvAP2-ERF20	1,5	9,626	1	20	2530	2549	AGAAUCUUGAUGAUGCUGCA	UGCAGCAUCAUCAGGAUUCC	Cleavage		1
nta-miR172e	PvAP2-ERF25	1,5	16,15	1	20	3064	3083	AGAAUCUUGAUGAUGCUGCA	UGCAGCAUCAUCAGGAUUCC	Cleavage		1
nta-miR172e	PvAP2-ERF62	1,5	16,463	1	20	3200	3219	AGAAUCUUGAUGAUGCUGCA	UGCAGCAUCAUCAGGAUUCC	Cleavage		1
nta-miR172e	PvAP2-ERF113	2,5	22,52	1	20	3572	3591	AGAAUCUUGAUGAUGCUGCA	AGCAGCAUCAUCAGGAUUCG	Cleavage		1
nta-miR172f	PvAP2-ERF173	0,5	11,33	1	20	3656	3675	AGAAUCUUGAUGAUGCUGCA	UGCAGCAUCAUCAGGAUUCU	Cleavage		1
nta-miR172f	PvAP2-ERF78	0,5	10,114	1	20	3797	3816	AGAAUCUUGAUGAUGCUGCA	UGCAGCAUCAUCAGGAUUCU	Cleavage		1
nta-miR172f	PvAP2-ERF20	1,5	9,626	1	20	2530	2549	AGAAUCUUGAUGAUGCUGCA	UGCAGCAUCAUCAGGAUUCC	Cleavage		1
nta-miR172f	PvAP2-ERF25	1,5	16,15	1	20	3064	3083	AGAAUCUUGAUGAUGCUGCA	UGCAGCAUCAUCAGGAUUCC	Cleavage		1
nta-miR172f	PvAP2-ERF62	1,5	16,463	1	20	3200	3219	AGAAUCUUGAUGAUGCUGCA	UGCAGCAUCAUCAGGAUUCC	Cleavage		1
nta-miR172f	PvAP2-ERF113	2,5	22,52	1	20	3572	3591	AGAAUCUUGAUGAUGCUGCA	AGCAGCAUCAUCAGGAUUCG	Cleavage		1
nta-miR172g	PvAP2-ERF173	0,5	11,33	1	20	3656	3675	AGAAUCUUGAUGAUGCUGCA	UGCAGCAUCAUCAGGAUUCU	Cleavage		1
nta-miR172g	PvAP2-ERF78	0,5	10,114	1	20	3797	3816	AGAAUCUUGAUGAUGCUGCA	UGCAGCAUCAUCAGGAUUCU	Cleavage		1
nta-miR172g	PvAP2-ERF20	1,5	9,626	1	20	2530	2549	AGAAUCUUGAUGAUGCUGCA	UGCAGCAUCAUCAGGAUUCC	Cleavage		1
nta-miR172g	PvAP2-ERF25	1,5	16,15	1	20	3064	3083	AGAAUCUUGAUGAUGCUGCA	UGCAGCAUCAUCAGGAUUCC	Cleavage		1
nta-miR172g	PvAP2-ERF62	1,5	16,463	1	20	3200	3219	AGAAUCUUGAUGAUGCUGCA	UGCAGCAUCAUCAGGAUUCC	Cleavage		1
nta-miR172g	PvAP2-ERF113	2,5	22,52	1	20	3572	3591	AGAAUCUUGAUGAUGCUGCA	AGCAGCAUCAUCAGGAUUCG	Cleavage		1
nta-miR172h	PvAP2-ERF173	0,5	11,33	1	20	3656	3675	AGAAUCUUGAUGAUGCUGCA	UGCAGCAUCAUCAGGAUUCU	Cleavage		1
nta-miR172h	PvAP2-ERF78	0,5	10,114	1	20	3797	3816	AGAAUCUUGAUGAUGCUGCA	UGCAGCAUCAUCAGGAUUCU	Cleavage		1
nta-miR172h	PvAP2-ERF20	1,5	9,626	1	20	2530	2549	AGAAUCUUGAUGAUGCUGCA	UGCAGCAUCAUCAGGAUUCC	Cleavage		1
nta-miR172h	PvAP2-ERF25	1,5	16,15	1	20	3064	3083	AGAAUCUUGAUGAUGCUGCA	UGCAGCAUCAUCAGGAUUCC	Cleavage		1
nta-miR172h	PvAP2-ERF62	1,5	16,463	1	20	3200	3219	AGAAUCUUGAUGAUGCUGCA	UGCAGCAUCAUCAGGAUUCC	Cleavage		1
nta-miR172h	PvAP2-ERF113	2,5	22,52	1	20	3572	3591	AGAAUCUUGAUGAUGCUGCA	AGCAGCAUCAUCAGGAUUCG	Cleavage		1
nta-miR172i	PvAP2-ERF173	0,5	11,33	1	20	3656	3675	AGAAUCUUGAUGAUGCUGCA	UGCAGCAUCAUCAGGAUUCU	Cleavage		1
nta-miR172i	PvAP2-ERF78	0,5	10,114	1	20	3797	3816	AGAAUCUUGAUGAUGCUGCA	UGCAGCAUCAUCAGGAUUCU	Cleavage		1
nta-miR172i	PvAP2-ERF20	1,5	9,626	1	20	2530	2549	AGAAUCUUGAUGAUGCUGCA	UGCAGCAUCAUCAGGAUUCC	Cleavage		1
nta-miR172i	PvAP2-ERF25	1,5	16,15	1	20	3064	3083	AGAAUCUUGAUGAUGCUGCA	UGCAGCAUCAUCAGGAUUCC	Cleavage		1
nta-miR172i	PvAP2-ERF62	1,5	16,463	1	20	3200	3219	AGAAUCUUGAUGAUGCUGCA	UGCAGCAUCAUCAGGAUUCC	Cleavage		1

nta-miR172i	PvAP2-ERF113	2,5	22,52	1	20	3572	3591	AGAAUCUUGAUGAUGCUGCA	AGCAGCAUCAUCAGGAUUCG	Cleavage		1
nta-miR172j	PvAP2-ERF20	0,5	9,626	1	20	2530	2549	GGAAUCUUGAUGAUGCUGCA	UGCAGCAUCAUCAGGAUUCC	Cleavage		1
nta-miR172j	PvAP2-ERF25	0,5	16,15	1	20	3064	3083	GGAAUCUUGAUGAUGCUGCA	UGCAGCAUCAUCAGGAUUCC	Cleavage		1
nta-miR172j	PvAP2-ERF62	0,5	16,463	1	20	3200	3219	GGAAUCUUGAUGAUGCUGCA	UGCAGCAUCAUCAGGAUUCC	Cleavage		1
nta-miR172j	PvAP2-ERF173	1	11,33	1	20	3656	3675	GGAAUCUUGAUGAUGCUGCA	UGCAGCAUCAUCAGGAUUCU	Cleavage		1
nta-miR172j	PvAP2-ERF78	1	10,114	1	20	3797	3816	GGAAUCUUGAUGAUGCUGCA	UGCAGCAUCAUCAGGAUUCU	Cleavage		1
nta-miR172j	PvAP2-ERF113	2,5	22,52	1	20	3572	3591	GGAAUCUUGAUGAUGCUGCA	AGCAGCAUCAUCAGGAUUCG	Cleavage		1
nta-miR6025d	PvAP2-ERF149	3	7,325	1	20	1748	1767	AACAAUUGAGAUAACAUCUA	UACAUGUCAUUUUAAUUGUU	Cleavage		1
nta-miR6144	PvAP2-ERF138	3	7,664	1	20	2819	2838	UGGCAACUUCUUCAUCAUGC	ACAUGAUAAAGAAGUUGUUA	Cleavage		1
nta-miR6145d	PvAP2-ERF179	2,5	13,364	1	20	1918	1937	AUUGUUACAUGUAACACUGG	CUAGUGUCACAUGUGGCAAU	Cleavage		1
nta-miR6145e	PvAP2-ERF179	3	13,364	1	20	1918	1937	AUUGUUACAUGUAGCACUGG	CUAGUGUCACAUGUGGCAAU	Cleavage		1
nta-miR6146a	PvAP2-ERF172	3	19,825	1	20	3065	3084	UUUGUCCAAUGAAACACUUA	UAAGUGUUGUAUGGGACAAG	Cleavage		1
nta-miR6151f	PvAP2-ERF10	3	3,71	1	20	58	77	UGAGUGUGAGGCAUUGGAUU	AUUCCAAUCACUCACACUCA	Translation		1
nta-miR6156	PvAP2-ERF167	3	17,498	1	21	52	72	UUGAAGAUGUUCUAUUUCUGU	AAAGAAAUGGAAAAUUUUCAA	Translation		1
Oryza sativa												
miRNA_Acc.	Target_Acc.	Expectation	UPE	miRNA_start	miRNA_end	Target_start	Target_end	miRNA_aligned_fragment	Target_aligned_fragment	Inhibition	Target_Desc.	Multiplicity
osa-miR1426	PvAP2-ERF62	2	11,764	1	20	2195	2214	AGAAUCUUGAUGAUGAUUAA	UUAAUCAUCAUUAGGGUUUU	Cleavage		1
osa-miR172a	PvAP2-ERF173	0,5	11,33	1	20	3656	3675	AGAAUCUUGAUGAUGCUGCA	UGCAGCAUCAUCAGGAUUCU	Cleavage		1
osa-miR172a	PvAP2-ERF78	0,5	10,114	1	20	3797	3816	AGAAUCUUGAUGAUGCUGCA	UGCAGCAUCAUCAGGAUUCU	Cleavage		1
osa-miR172a	PvAP2-ERF20	1,5	9,626	1	20	2530	2549	AGAAUCUUGAUGAUGCUGCA	UGCAGCAUCAUCAGGAUUCC	Cleavage		1
osa-miR172a	PvAP2-ERF25	1,5	16,15	1	20	3064	3083	AGAAUCUUGAUGAUGCUGCA	UGCAGCAUCAUCAGGAUUCC	Cleavage		1
osa-miR172a	PvAP2-ERF62	1,5	16,463	1	20	3200	3219	AGAAUCUUGAUGAUGCUGCA	UGCAGCAUCAUCAGGAUUCC	Cleavage		1
osa-miR172a	PvAP2-ERF113	2,5	22,52	1	20	3572	3591	AGAAUCUUGAUGAUGCUGCA	AGCAGCAUCAUCAGGAUUCG	Cleavage		1
osa-miR172b	PvAP2-ERF62	0,5	16,463	1	20	3200	3219	GGAAUCUUGAUGAUGCUGCA	UGCAGCAUCAUCAGGAUUCC	Cleavage		1
osa-miR172b	PvAP2-ERF25	0,5	16,15	1	20	3064	3083	GGAAUCUUGAUGAUGCUGCA	UGCAGCAUCAUCAGGAUUCC	Cleavage		1
osa-miR172b	PvAP2-ERF20	0,5	9,626	1	20	2530	2549	GGAAUCUUGAUGAUGCUGCA	UGCAGCAUCAUCAGGAUUCC	Cleavage		1
osa-miR172b	PvAP2-ERF78	1	10,114	1	20	3797	3816	GGAAUCUUGAUGAUGCUGCA	UGCAGCAUCAUCAGGAUUCU	Cleavage		1
osa-miR172b	PvAP2-ERF173	1	11,33	1	20	3656	3675	GGAAUCUUGAUGAUGCUGCA	UGCAGCAUCAUCAGGAUUCU	Cleavage		1
osa-miR172b	PvAP2-ERF113	2,5	22,52	1	20	3572	3591	GGAAUCUUGAUGAUGCUGCA	AGCAGCAUCAUCAGGAUUCG	Cleavage		1
osa-miR172c	PvAP2-ERF20	1,5	9,626	1	20	2530	2549	UGAAUCUUGAUGAUGCUGCA	UGCAGCAUCAUCAGGAUUCC	Cleavage		1
osa-miR172c	PvAP2-ERF25	1,5	16,15	1	20	3064	3083	UGAAUCUUGAUGAUGCUGCA	UGCAGCAUCAUCAGGAUUCC	Cleavage		1
osa-miR172c	PvAP2-ERF62	1,5	16,463	1	20	3200	3219	UGAAUCUUGAUGAUGCUGCA	UGCAGCAUCAUCAGGAUUCC	Cleavage		1
osa-miR172c	PvAP2-ERF173	1,5	11,33	1	20	3656	3675	UGAAUCUUGAUGAUGCUGCA	UGCAGCAUCAUCAGGAUUCU	Cleavage		1
osa-miR172c	PvAP2-ERF78	1,5	10,114	1	20	3797	3816	UGAAUCUUGAUGAUGCUGCA	UGCAGCAUCAUCAGGAUUCU	Cleavage		1
osa-miR172c	PvAP2-ERF113	2	22,52	1	20	3572	3591	UGAAUCUUGAUGAUGCUGCA	AGCAGCAUCAUCAGGAUUCG	Cleavage		1
osa-miR172d-3p	PvAP2-ERF173	0,5	11,33	1	20	3656	3675	AGAAUCUUGAUGAUGCUGCA	UGCAGCAUCAUCAGGAUUCU	Cleavage		1
osa-miR172d-3p	PvAP2-ERF78	0,5	10,114	1	20	3797	3816	AGAAUCUUGAUGAUGCUGCA	UGCAGCAUCAUCAGGAUUCU	Cleavage		1
osa-miR172d-3p	PvAP2-ERF20	1,5	9,626	1	20	2530	2549	AGAAUCUUGAUGAUGCUGCA	UGCAGCAUCAUCAGGAUUCC	Cleavage		1
osa-miR172d-3p	PvAP2-ERF25	1,5	16,15	1	20	3064	3083	AGAAUCUUGAUGAUGCUGCA	UGCAGCAUCAUCAGGAUUCC	Cleavage		1
osa-miR172d-3p	PvAP2-ERF62	1,5	16,463	1	20	3200	3219	AGAAUCUUGAUGAUGCUGCA	UGCAGCAUCAUCAGGAUUCC	Cleavage		1
osa-miR172d-3p	PvAP2-ERF113	2,5	22,52	1	20	3572	3591	AGAAUCUUGAUGAUGCUGCA	AGCAGCAUCAUCAGGAUUCG	Cleavage		1
osa-miR1865-5p	PvAP2-ERF179	3	16,671	1	21	4489	4509	UGCUAGUGAUGGUGAUUCUUC	GAAGAAUCAUCAUCAUUGGUC	Cleavage		1
osa-miR1879	PvAP2-ERF90	3	6,041	1	24	136	159	GUGUUUGGUUUAGGGAUGAGGUGG	CCCAUUCAUUCAAAAACCAAACAC	Cleavage		1
osa-miR2055	PvAP2-ERF85	3	14,072	1	20	213	232	UUUCCUUGGGAAGGUGGUUU	GAACCAACUUUACAAGGAAA	Translation		1
osa-miR2099-5p	PvAP2-ERF71	3	13,468	1	21	342	362	UGAAUAUGUUUGUACAAGCUU	AAGCAUGUACAAAGGUGUUCA	Cleavage		1
osa-miR2275a	PvAP2-ERF7	1	16,862	1	22	1455	1476	UUUGGUUUCCUCCAAUAUCUCA	UGAGAAAUUGGAGGAAACCAAA	Cleavage		1
osa-miR2275b	PvAP2-ERF7	1	16,862	1	22	1455	1476	UUUGGUUUCCUCCAAUAUCUCA	UGAGAAAUUGGAGGAAACCAAA	Cleavage		1
osa-miR2873c	PvAP2-ERF172	3	21,923	1	20	2673	2692	CAAAUGAAGUUGAGUUUGGA	UCCAAACGCAGCUUCAGUUG	Cleavage		1
osa-miR2923	PvAP2-ERF46	2,5	17,409	1	22	2388	2409	AGACAAAAAUAUAAAUAACAAA	UUUGUUAUUUAUUUGUUUGUUU	Translation		1
osa-miR414	PvAP2-ERF166	2	23,018	1	21	329	349	UCAUCCUCAUCAUCAUCGUCC	GGAUGAUGAUGAUGAGGACGA	Cleavage		1
osa-miR414	PvAP2-ERF176	2,5	17,029	1	21	859	879	UCAUCCUCAUCAUCAUCGUCC	GGAGGAUGAUGAUGAUGAUGA	Cleavage		1
osa-miR435	PvAP2-ERF60	3	19,24	1	20	953	972	UUAUCCGGUAUUGGAGUUGA	UCAACUCCAAAACUGGAGAA	Translation		1
osa-miR5083	PvAP2-ERF78	3	17,266	1	20	3004	3023	AGACUACAAUUAUCUGAUCA	UGAAUAGGUUAUUGUAGUCU	Translation		1
osa-miR5155	PvAP2-ERF157	2,5	13,133	1	20	1103	1122	ACUUUAAUACCAUUGGAAGA	UUUUCCAAUUGUAUUAAAGG	Translation		1
osa-miR529b	PvAP2-ERF94	3	21,493	1	20	486	505	AGAAGAGAGAGAGUACAGCU	ACUUGUAAUCUCUCUCUUUU	Cleavage		1

osa-miR5339	PvAP2-ERF141	2,5	23,148	1	20	615	634	CAGAUAGAGAAUCUUCUCAG	CUGAGGAGGUUGUCUAUUUG	Translation		1
osa-miR5537	PvAP2-ERF78	2,5	7,173	1	21	2722	2742	AAUGUUUGUAUGGAUCGUUUG	UAAAUGCUUCAUAUAAACAUU	Cleavage		1
osa-miR5809	PvAP2-ERF110	3	18,828	1	20	881	900	UCGUCGCCGGCGACCACAGC	GUUGUUGUUUCCGGCGACGA	Translation		1
osa-miR5817	PvAP2-ERF152	3	11,749	1	20	2734	2753	AUCGAAUUUGAAAGAAAAAG	AUUUUUCUUUCAAAUUUGUU	Cleavage		1
osa-miR5819	PvAP2-ERF146	3	24,666	1	20	522	541	AGGACGAGGGGAACGGCGGC	GCCGUCCUCCCUCUCGUCCU	Cleavage		1
osa-miR6251	PvAP2-ERF15	3	15,935	1	20	244	263	UGUGUAGCCACAUUGUAAGG	CCUUGCAAUGUGGCUACUCU	Cleavage		1
Physcomitrella patens												
miRNA_Acc.	Target_Acc.	Expectation	UPE	miRNA_start	miRNA_end	Target_start	Target_end	miRNA_aligned_fragment	Target_aligned_fragment	Inhibition	Target_Desc.	Multiplicity
ppt-miR1023b-5p	PvAP2-ERF126	3	8,59	1	20	1201	1220	ACACUCUCUCCAUUUCUCUG	CAGAGAAUUGGAGAGGGUUU	Cleavage		1
ppt-miR1023c-5p	PvAP2-ERF25	3	17,392	1	22	90	111	CCACUCUCUCCGUUUCCCUUCC	GAGAGGGGAGAGGAGAGGGUGG	Cleavage		1
ppt-miR1023d-5p	PvAP2-ERF25	3	17,392	1	22	90	111	CCACUCUCUCCGUUUCCCUUCC	GAGAGGGGAGAGGAGAGGGUGG	Cleavage		1
ppt-miR1039-5p	PvAP2-ERF171	3	16,832	1	20	52	71	UCUUUGGGUCUUUCUCUCCU	AGGACAAAAGGACCUAAAGA	Cleavage		1
ppt-miR1054	PvAP2-ERF84	3	19,879	1	20	244	263	UAAACCCUCUCUCUAUUCCU	AGGAACGGAGUGAGGGUUUG	Translation		1
ppt-miR1069-3p	PvAP2-ERF7	3	14,795	1	21	1406	1426	UGAUAAAUCAAAGUGCUCACU	AGUGAGCAAUUGGAUUUGUUA	Translation		1
ppt-miR414	PvAP2-ERF166	3	23,018	1	21	329	349	UCAUCCUCAUCAUCCUCGUCC	GGAUGAUGAUGAUGAGGACGA	Cleavage		1
ppt-miR419	PvAP2-ERF56	3	13,323	1	20	6	24	UGAUGAAUGAUGACGAUGUA	UUCAUCGUCAUC-UUCAUCA	Cleavage		1
Pinus taeda												
miRNA_Acc.	Target_Acc.	Expectation	UPE	miRNA_start	miRNA_end	Target_start	Target_end	miRNA_aligned_fragment	Target_aligned_fragment	Inhibition	Target_Desc.	Multiplicity
pta-miR156b	PvAP2-ERF53	2,5	12,909	1	20	811	830	CAGAAGAUAGAGAGCACAAC	GUGGUGUUCUCAAUCUUCUG	Translation		1
Populus trichocarpa												
miRNA_Acc.	Target_Acc.	Expectation	UPE	miRNA_start	miRNA_end	Target_start	Target_end	miRNA_aligned_fragment	Target_aligned_fragment	Inhibition	Target_Desc.	Multiplicity
ptc-miR1450	PvAP2-ERF123	3	14,816	1	20	562	581	UUCAAUGGCUCGGUCAGGUU	AACCUCACUGAUCUAUUGAA	Translation		1
ptc-miR156l	PvAP2-ERF117	3	21,038	1	20	383	402	UUGACAGAAGAUGGAGAGCA	UCUUCUUCUUCUUCUGUCAA	Cleavage		1
ptc-miR172a	PvAP2-ERF173	0,5	11,33	1	20	3656	3675	AGAAUCUUGAUGAUGCUGCA	UGCAGCAUCAUCAGGAUUCU	Cleavage		1
ptc-miR172a	PvAP2-ERF78	0,5	10,114	1	20	3797	3816	AGAAUCUUGAUGAUGCUGCA	UGCAGCAUCAUCAGGAUUCU	Cleavage		1
ptc-miR172a	PvAP2-ERF20	1,5	9,626	1	20	2530	2549	AGAAUCUUGAUGAUGCUGCA	UGCAGCAUCAUCAGGAUUCC	Cleavage		1
ptc-miR172a	PvAP2-ERF25	1,5	16,15	1	20	3064	3083	AGAAUCUUGAUGAUGCUGCA	UGCAGCAUCAUCAGGAUUCC	Cleavage		1
ptc-miR172a	PvAP2-ERF62	1,5	16,463	1	20	3200	3219	AGAAUCUUGAUGAUGCUGCA	UGCAGCAUCAUCAGGAUUCC	Cleavage		1
ptc-miR172a	PvAP2-ERF113	2,5	22,52	1	20	3572	3591	AGAAUCUUGAUGAUGCUGCA	AGCAGCAUCAUCAGGAUUCG	Cleavage		1
ptc-miR172b-3p	PvAP2-ERF173	0,5	11,33	1	20	3656	3675	AGAAUCUUGAUGAUGCUGCA	UGCAGCAUCAUCAGGAUUCU	Cleavage		1
ptc-miR172b-3p	PvAP2-ERF78	0,5	10,114	1	20	3797	3816	AGAAUCUUGAUGAUGCUGCA	UGCAGCAUCAUCAGGAUUCU	Cleavage		1
ptc-miR172b-3p	PvAP2-ERF20	1,5	9,626	1	20	2530	2549	AGAAUCUUGAUGAUGCUGCA	UGCAGCAUCAUCAGGAUUCC	Cleavage		1
ptc-miR172b-3p	PvAP2-ERF25	1,5	16,15	1	20	3064	3083	AGAAUCUUGAUGAUGCUGCA	UGCAGCAUCAUCAGGAUUCC	Cleavage		1
ptc-miR172b-3p	PvAP2-ERF62	1,5	16,463	1	20	3200	3219	AGAAUCUUGAUGAUGCUGCA	UGCAGCAUCAUCAGGAUUCC	Cleavage		1
ptc-miR172b-3p	PvAP2-ERF113	2,5	22,52	1	20	3572	3591	AGAAUCUUGAUGAUGCUGCA	AGCAGCAUCAUCAGGAUUCG	Cleavage		1
ptc-miR172c	PvAP2-ERF173	0,5	11,33	1	20	3656	3675	AGAAUCUUGAUGAUGCUGCA	UGCAGCAUCAUCAGGAUUCU	Cleavage		1
ptc-miR172c	PvAP2-ERF78	0,5	10,114	1	20	3797	3816	AGAAUCUUGAUGAUGCUGCA	UGCAGCAUCAUCAGGAUUCU	Cleavage		1
ptc-miR172c	PvAP2-ERF20	1,5	9,626	1	20	2530	2549	AGAAUCUUGAUGAUGCUGCA	UGCAGCAUCAUCAGGAUUCC	Cleavage		1
ptc-miR172c	PvAP2-ERF25	1,5	16,15	1	20	3064	3083	AGAAUCUUGAUGAUGCUGCA	UGCAGCAUCAUCAGGAUUCC	Cleavage		1
ptc-miR172c	PvAP2-ERF62	1,5	16,463	1	20	3200	3219	AGAAUCUUGAUGAUGCUGCA	UGCAGCAUCAUCAGGAUUCC	Cleavage		1
ptc-miR172c	PvAP2-ERF113	2,5	22,52	1	20	3572	3591	AGAAUCUUGAUGAUGCUGCA	AGCAGCAUCAUCAGGAUUCG	Cleavage		1
ptc-miR172d	PvAP2-ERF20	0,5	9,626	1	20	2530	2549	GGAAUCUUGAUGAUGCUGCA	UGCAGCAUCAUCAGGAUUCC	Cleavage		1
ptc-miR172d	PvAP2-ERF25	0,5	16,15	1	20	3064	3083	GGAAUCUUGAUGAUGCUGCA	UGCAGCAUCAUCAGGAUUCC	Cleavage		1
ptc-miR172d	PvAP2-ERF62	0,5	16,463	1	20	3200	3219	GGAAUCUUGAUGAUGCUGCA	UGCAGCAUCAUCAGGAUUCC	Cleavage		1
ptc-miR172d	PvAP2-ERF173	1	11,33	1	20	3656	3675	GGAAUCUUGAUGAUGCUGCA	UGCAGCAUCAUCAGGAUUCU	Cleavage		1
ptc-miR172d	PvAP2-ERF78	1	10,114	1	20	3797	3816	GGAAUCUUGAUGAUGCUGCA	UGCAGCAUCAUCAGGAUUCU	Cleavage		1
ptc-miR172d	PvAP2-ERF113	2,5	22,52	1	20	3572	3591	GGAAUCUUGAUGAUGCUGCA	AGCAGCAUCAUCAGGAUUCG	Cleavage		1
ptc-miR172e	PvAP2-ERF20	0,5	9,626	1	20	2530	2549	GGAAUCUUGAUGAUGCUGCA	UGCAGCAUCAUCAGGAUUCC	Cleavage		1
ptc-miR172e	PvAP2-ERF25	0,5	16,15	1	20	3064	3083	GGAAUCUUGAUGAUGCUGCA	UGCAGCAUCAUCAGGAUUCC	Cleavage		1
ptc-miR172e	PvAP2-ERF62	0,5	16,463	1	20	3200	3219	GGAAUCUUGAUGAUGCUGCA	UGCAGCAUCAUCAGGAUUCC	Cleavage		1
ptc-miR172e	PvAP2-ERF173	1	11,33	1	20	3656	3675	GGAAUCUUGAUGAUGCUGCA	UGCAGCAUCAUCAGGAUUCU	Cleavage		1
ptc-miR172e	PvAP2-ERF78	1	10,114	1	20	3797	3816	GGAAUCUUGAUGAUGCUGCA	UGCAGCAUCAUCAGGAUUCU	Cleavage		1
ptc-miR172e	PvAP2-ERF113	2,5	22,52	1	20	3572	3591	GGAAUCUUGAUGAUGCUGCA	AGCAGCAUCAUCAGGAUUCG	Cleavage		1
ptc-miR172f	PvAP2-ERF173	0,5	11,33	1	20	3656	3675	AGAAUCUUGAUGAUGCUGCA	UGCAGCAUCAUCAGGAUUCU	Cleavage		1
ptc-miR172f	PvAP2-ERF78	0,5	10,114	1	20	3797	3816	AGAAUCUUGAUGAUGCUGCA	UGCAGCAUCAUCAGGAUUCU	Cleavage		1

ptc-miR172f	PvAP2-ERF20	1,5	9,626	1	20	2530	2549	AGAAUCUUGAUGAUGCUGCA	UGCAGCAUCAUCAGGAUUCC	Cleavage		1
ptc-miR172f	PvAP2-ERF25	1,5	16,15	1	20	3064	3083	AGAAUCUUGAUGAUGCUGCA	UGCAGCAUCAUCAGGAUUCC	Cleavage		1
ptc-miR172f	PvAP2-ERF62	1,5	16,463	1	20	3200	3219	AGAAUCUUGAUGAUGCUGCA	UGCAGCAUCAUCAGGAUUCC	Cleavage		1
ptc-miR172f	PvAP2-ERF113	2,5	22,52	1	20	3572	3591	AGAAUCUUGAUGAUGCUGCA	AGCAGCAUCAUCAGGAUUCG	Cleavage		1
ptc-miR172g-3p	PvAP2-ERF20	0,5	9,626	1	21	2529	2549	GGAAUCUUGAUGAUGCUGCAG	CUGCAGCAUCAUCAGGAUUCC	Cleavage		1
ptc-miR172g-3p	PvAP2-ERF25	0,5	16,15	1	21	3063	3083	GGAAUCUUGAUGAUGCUGCAG	CUGCAGCAUCAUCAGGAUUCC	Cleavage		1
ptc-miR172g-3p	PvAP2-ERF62	0,5	16,463	1	21	3199	3219	GGAAUCUUGAUGAUGCUGCAG	CUGCAGCAUCAUCAGGAUUCC	Cleavage		1
ptc-miR172g-3p	PvAP2-ERF173	1	11,33	1	21	3655	3675	GGAAUCUUGAUGAUGCUGCAG	CUGCAGCAUCAUCAGGAUUCU	Cleavage		1
ptc-miR172g-3p	PvAP2-ERF78	1	10,114	1	21	3796	3816	GGAAUCUUGAUGAUGCUGCAG	CUGCAGCAUCAUCAGGAUUCU	Cleavage		1
ptc-miR172g-3p	PvAP2-ERF113	2,5	22,52	1	21	3571	3591	GGAAUCUUGAUGAUGCUGCAG	CAGCAGCAUCAUCAGGAUUCG	Cleavage		1
ptc-miR172h-3p	PvAP2-ERF20	0,5	9,626	1	21	2529	2549	GGAAUCUUGAUGAUGCUGCAG	CUGCAGCAUCAUCAGGAUUCC	Cleavage		1
ptc-miR172h-3p	PvAP2-ERF25	0,5	16,15	1	21	3063	3083	GGAAUCUUGAUGAUGCUGCAG	CUGCAGCAUCAUCAGGAUUCC	Cleavage		1
ptc-miR172h-3p	PvAP2-ERF62	0,5	16,463	1	21	3199	3219	GGAAUCUUGAUGAUGCUGCAG	CUGCAGCAUCAUCAGGAUUCC	Cleavage		1
ptc-miR172h-3p	PvAP2-ERF173	1	11,33	1	21	3655	3675	GGAAUCUUGAUGAUGCUGCAG	CUGCAGCAUCAUCAGGAUUCU	Cleavage		1
ptc-miR172h-3p	PvAP2-ERF78	1	10,114	1	21	3796	3816	GGAAUCUUGAUGAUGCUGCAG	CUGCAGCAUCAUCAGGAUUCU	Cleavage		1
ptc-miR172h-3p	PvAP2-ERF113	2,5	22,52	1	21	3571	3591	GGAAUCUUGAUGAUGCUGCAG	CAGCAGCAUCAUCAGGAUUCG	Cleavage		1
ptc-miR172i	PvAP2-ERF173	0	11,33	1	20	3656	3675	AGAAUCCUGAUGAUGCUGCA	UGCAGCAUCAUCAGGAUUCU	Cleavage		1
ptc-miR172i	PvAP2-ERF78	0	10,114	1	20	3797	3816	AGAAUCCUGAUGAUGCUGCA	UGCAGCAUCAUCAGGAUUCU	Cleavage		1
ptc-miR172i	PvAP2-ERF20	1	9,626	1	20	2530	2549	AGAAUCCUGAUGAUGCUGCA	UGCAGCAUCAUCAGGAUUCC	Cleavage		1
ptc-miR172i	PvAP2-ERF25	1	16,15	1	20	3064	3083	AGAAUCCUGAUGAUGCUGCA	UGCAGCAUCAUCAGGAUUCC	Cleavage		1
ptc-miR172i	PvAP2-ERF62	1	16,463	1	20	3200	3219	AGAAUCCUGAUGAUGCUGCA	UGCAGCAUCAUCAGGAUUCC	Cleavage		1
ptc-miR172i	PvAP2-ERF113	2	22,52	1	20	3572	3591	AGAAUCCUGAUGAUGCUGCA	AGCAGCAUCAUCAGGAUUCG	Cleavage		1
ptc-miR395a	PvAP2-ERF179	3	11,026	1	20	4356	4375	CUGAAGGGUUUGGAGGAACU	AGUUCCUCCUAACCUCUCAG	Translation		1
ptc-miR478e	PvAP2-ERF104	3	10,814	1	23	145	167	UGACGAGUCUUCUAUUUUUAGGG	CUGUAAAAGUUGAAGACUCCUCA	Cleavage		1
ptc-miR6426a	PvAP2-ERF60	2,5	16,344	1	20	2387	2406	GUGGAGACAUGGAAGUGAAG	UUUCUCUUUCAUGUUUCCAC	Cleavage		1
ptc-miR6426a	PvAP2-ERF128	3	9,875	1	20	6669	6688	GUGGAGACAUGGAAGUGAAG	UUUUAUUUUCAUAUCUCCAC	Cleavage		1
ptc-miR6426b	PvAP2-ERF60	2,5	16,344	1	20	2387	2406	GUGGAGACAUGGAAGUGAAG	UUUCUCUUUCAUGUUUCCAC	Cleavage		1
ptc-miR6426b	PvAP2-ERF128	3	9,875	1	20	6669	6688	GUGGAGACAUGGAAGUGAAG	UUUUAUUUUCAUAUCUCCAC	Cleavage		1
ptc-miR6447	PvAP2-ERF25	2,5	21,38	1	21	797	817	UUGACGAAAUGUGACGACUAC	GUAGGCGUCAAAUUUUGUCAA	Translation		1
ptc-miR6455	PvAP2-ERF146	3	12,568	1	21	1123	1143	UCAAAUAGCAUCCUCAACAUU	GAUGAUGAUGAUGUUAUUUGG	Cleavage		1
ptc-miR7816	PvAP2-ERF165	1,5	9,181	1	20	899	918	AAUGUUGUUAUUAACACUGU	GCACUGUUAAUAACAACAUU	Cleavage		1
ptc-miR7825	PvAP2-ERF141	3	13,382	1	22	270	292	UUGAAGAAAG-GUAGACAGAUAG	UUUUUUGUCUACACUUUCUUCAG	Translation		1
ptc-miR7825	PvAP2-ERF68	3	15,666	1	20	137	156	UUGAAGAAAGGUAGACAGAU	AGCUGUGAACCUUUCUUCAA	Cleavage		1
ptc-miR7833	PvAP2-ERF12	3	9,648	1	19	2042	2061	UAAUUA-GAACUCAUACUAG	UUAGUAUGAGUUCAUAAUUA	Cleavage		1
Ricinus communis												
miRNA_Acc.	Target_Acc.	Expectation	UPE	miRNA_start	miRNA_end	Target_start	Target_end	miRNA_aligned_fragment	Target_aligned_fragment	Inhibition	Target_Desc.	Multiplicity
rco-miR172	PvAP2-ERF20	0,5	9,626	1	21	2529	2549	GGAAUCUUGAUGAUGCUGCAG	CUGCAGCAUCAUCAGGAUUCC	Cleavage		1
rco-miR172	PvAP2-ERF25	0,5	16,15	1	21	3063	3083	GGAAUCUUGAUGAUGCUGCAG	CUGCAGCAUCAUCAGGAUUCC	Cleavage		1
rco-miR172	PvAP2-ERF62	0,5	16,463	1	21	3199	3219	GGAAUCUUGAUGAUGCUGCAG	CUGCAGCAUCAUCAGGAUUCC	Cleavage		1
rco-miR172	PvAP2-ERF173	1	11,33	1	21	3655	3675	GGAAUCUUGAUGAUGCUGCAG	CUGCAGCAUCAUCAGGAUUCU	Cleavage		1
rco-miR172	PvAP2-ERF78	1	10,114	1	21	3796	3816	GGAAUCUUGAUGAUGCUGCAG	CUGCAGCAUCAUCAGGAUUCU	Cleavage		1
rco-miR172	PvAP2-ERF113	2,5	22,52	1	21	3571	3591	GGAAUCUUGAUGAUGCUGCAG	CAGCAGCAUCAUCAGGAUUCG	Cleavage		1
Sorghum bicolor												
miRNA_Acc.	Target_Acc.	Expectation	UPE	miRNA_start	miRNA_end	Target_start	Target_end	miRNA_aligned_fragment	Target_aligned_fragment	Inhibition	Target_Desc.	Multiplicity
sbi-miR172a	PvAP2-ERF173	0,5	11,33	1	20	3656	3675	AGAAUCUUGAUGAUGCUGCA	UGCAGCAUCAUCAGGAUUCU	Cleavage		1
sbi-miR172a	PvAP2-ERF78	0,5	10,114	1	20	3797	3816	AGAAUCUUGAUGAUGCUGCA	UGCAGCAUCAUCAGGAUUCU	Cleavage		1
sbi-miR172a	PvAP2-ERF20	1,5	9,626	1	20	2530	2549	AGAAUCUUGAUGAUGCUGCA	UGCAGCAUCAUCAGGAUUCC	Cleavage		1
sbi-miR172a	PvAP2-ERF25	1,5	16,15	1	20	3064	3083	AGAAUCUUGAUGAUGCUGCA	UGCAGCAUCAUCAGGAUUCC	Cleavage		1
sbi-miR172a	PvAP2-ERF62	1,5	16,463	1	20	3200	3219	AGAAUCUUGAUGAUGCUGCA	UGCAGCAUCAUCAGGAUUCC	Cleavage		1
sbi-miR172a	PvAP2-ERF113	2,5	22,52	1	20	3572	3591	AGAAUCUUGAUGAUGCUGCA	AGCAGCAUCAUCAGGAUUCG	Cleavage		1
sbi-miR172b	PvAP2-ERF20	0,5	9,626	1	20	2530	2549	GGAAUCUUGAUGAUGCUGCA	UGCAGCAUCAUCAGGAUUCC	Cleavage		1
sbi-miR172b	PvAP2-ERF25	0,5	16,15	1	20	3064	3083	GGAAUCUUGAUGAUGCUGCA	UGCAGCAUCAUCAGGAUUCC	Cleavage		1
sbi-miR172b	PvAP2-ERF62	0,5	16,463	1	20	3200	3219	GGAAUCUUGAUGAUGCUGCA	UGCAGCAUCAUCAGGAUUCC	Cleavage		1
sbi-miR172b	PvAP2-ERF173	1	11,33	1	20	3656	3675	GGAAUCUUGAUGAUGCUGCA	UGCAGCAUCAUCAGGAUUCU	Cleavage		1
sbi-miR172b	PvAP2-ERF78	1	10,114	1	20	3797	3816	GGAAUCUUGAUGAUGCUGCA	UGCAGCAUCAUCAGGAUUCU	Cleavage		1

sbi-miR172b	PvAP2-ERF113	2,5	22,52	1	20	3572	3591	GGAAUCUUGAUGAUGCUGCA	AGCAGCAUCAUCAGGAUUCG	Cleavage		1
sbi-miR172c	PvAP2-ERF173	0,5	11,33	1	20	3656	3675	AGAAUCUUGAUGAUGCUGCA	UGCAGCAUCAUCAGGAUUCU	Cleavage		1
sbi-miR172c	PvAP2-ERF78	0,5	10,114	1	20	3797	3816	AGAAUCUUGAUGAUGCUGCA	UGCAGCAUCAUCAGGAUUCU	Cleavage		1
sbi-miR172c	PvAP2-ERF20	1,5	9,626	1	20	2530	2549	AGAAUCUUGAUGAUGCUGCA	UGCAGCAUCAUCAGGAUUCC	Cleavage		1
sbi-miR172c	PvAP2-ERF25	1,5	16,15	1	20	3064	3083	AGAAUCUUGAUGAUGCUGCA	UGCAGCAUCAUCAGGAUUCC	Cleavage		1
sbi-miR172c	PvAP2-ERF62	1,5	16,463	1	20	3200	3219	AGAAUCUUGAUGAUGCUGCA	UGCAGCAUCAUCAGGAUUCC	Cleavage		1
sbi-miR172c	PvAP2-ERF113	2,5	22,52	1	20	3572	3591	AGAAUCUUGAUGAUGCUGCA	AGCAGCAUCAUCAGGAUUCG	Cleavage		1
sbi-miR172d	PvAP2-ERF173	0,5	11,33	1	20	3656	3675	AGAAUCUUGAUGAUGCUGCA	UGCAGCAUCAUCAGGAUUCU	Cleavage		1
sbi-miR172d	PvAP2-ERF78	0,5	10,114	1	20	3797	3816	AGAAUCUUGAUGAUGCUGCA	UGCAGCAUCAUCAGGAUUCU	Cleavage		1
sbi-miR172d	PvAP2-ERF20	1,5	9,626	1	20	2530	2549	AGAAUCUUGAUGAUGCUGCA	UGCAGCAUCAUCAGGAUUCC	Cleavage		1
sbi-miR172d	PvAP2-ERF25	1,5	16,15	1	20	3064	3083	AGAAUCUUGAUGAUGCUGCA	UGCAGCAUCAUCAGGAUUCC	Cleavage		1
sbi-miR172d	PvAP2-ERF62	1,5	16,463	1	20	3200	3219	AGAAUCUUGAUGAUGCUGCA	UGCAGCAUCAUCAGGAUUCC	Cleavage		1
sbi-miR172d	PvAP2-ERF113	2,5	22,52	1	20	3572	3591	AGAAUCUUGAUGAUGCUGCA	AGCAGCAUCAUCAGGAUUCG	Cleavage		1
sbi-miR172e	PvAP2-ERF20	1,5	9,626	1	20	2530	2549	UGAAUCUUGAUGAUGCUGCA	UGCAGCAUCAUCAGGAUUCC	Cleavage		1
sbi-miR172e	PvAP2-ERF25	1,5	16,15	1	20	3064	3083	UGAAUCUUGAUGAUGCUGCA	UGCAGCAUCAUCAGGAUUCC	Cleavage		1
sbi-miR172e	PvAP2-ERF62	1,5	16,463	1	20	3200	3219	UGAAUCUUGAUGAUGCUGCA	UGCAGCAUCAUCAGGAUUCC	Cleavage		1
sbi-miR172e	PvAP2-ERF173	1,5	11,33	1	20	3656	3675	UGAAUCUUGAUGAUGCUGCA	UGCAGCAUCAUCAGGAUUCU	Cleavage		1
sbi-miR172e	PvAP2-ERF78	1,5	10,114	1	20	3797	3816	UGAAUCUUGAUGAUGCUGCA	UGCAGCAUCAUCAGGAUUCU	Cleavage		1
sbi-miR172e	PvAP2-ERF113	2	22,52	1	20	3572	3591	UGAAUCUUGAUGAUGCUGCA	AGCAGCAUCAUCAGGAUUCG	Cleavage		1
sbi-miR172f	PvAP2-ERF78	0	10,114	1	20	3797	3816	AGAAUCCUGAUGAUGCUGCA	UGCAGCAUCAUCAGGAUUCU	Cleavage		1
sbi-miR172f	PvAP2-ERF173	0	11,33	1	20	3656	3675	AGAAUCCUGAUGAUGCUGCA	UGCAGCAUCAUCAGGAUUCU	Cleavage		1
sbi-miR172f	PvAP2-ERF62	1	16,463	1	20	3200	3219	AGAAUCCUGAUGAUGCUGCA	UGCAGCAUCAUCAGGAUUCC	Cleavage		1
sbi-miR172f	PvAP2-ERF25	1	16,15	1	20	3064	3083	AGAAUCCUGAUGAUGCUGCA	UGCAGCAUCAUCAGGAUUCC	Cleavage		1
sbi-miR172f	PvAP2-ERF20	1	9,626	1	20	2530	2549	AGAAUCCUGAUGAUGCUGCA	UGCAGCAUCAUCAGGAUUCC	Cleavage		1
sbi-miR172f	PvAP2-ERF113	2	22,52	1	20	3572	3591	AGAAUCCUGAUGAUGCUGCA	AGCAGCAUCAUCAGGAUUCG	Cleavage		1
sbi-miR5570	PvAP2-ERF141	3	13,82	1	20	407	426	AAAAGACAAAUCAGCAUGUC	GAUUUUCUGGUUUGUCUUUU	Cleavage		1
sbi-miR6228-3p	PvAP2-ERF60	3	14,82	1	24	1480	1503	GUGGCAGUAGAAUUAAUGAAGGGA	UGCUGUCAUUGAUUCUGUUGCCAU	Cleavage		1
sbi-miR6233-3p	PvAP2-ERF23	3	12,748	1	20	1870	1888	CAAGUUUGGUUUUGGUAAUU	AAUUAUCAAAAC-GAACUUG	Cleavage		1
sbi-miR821a	PvAP2-ERF46	3	17,925	1	21	2190	2210	AAGUCAUCAACAUAAAAGUUG	UGAUUUUGAUUUUGAUGACUU	Translation		1
sbi-miR821b	PvAP2-ERF65	3	13,105	1	21	1046	1066	AAGUUAUGAACAUAAAAGUUG	UAACUUUGAUGUCCAUGAUUU	Translation		1
sbi-miR821b	PvAP2-ERF113	3	20,581	1	20	1250	1269	AAGUUAUGAACAUAAAAGUU	AACUUUUGUGAUUAUAGUUU	Translation		1
sbi-miR821c	PvAP2-ERF46	3	17,925	1	21	2190	2210	AAGUCAUCAACAUAAAAGUUG	UGAUUUUGAUUUUGAUGACUU	Translation		1
sbi-miR821d	PvAP2-ERF46	3	17,925	1	21	2190	2210	AAGUCAUCAACAACAAAGUUG	UGAUUUUGAUUUUGAUGACUU	Translation		1
sbi-miR821e	PvAP2-ERF46	2	17,925	1	21	2190	2210	AAGUCAUCAAAAUAAAAGUUG	UGAUUUUGAUUUUGAUGACUU	Cleavage		1
Solanum lycopersicum												
miRNA_Acc.	Target_Acc.	Expectation	UPE	miRNA_start	miRNA_end	Target_start	Target_end	miRNA_aligned_fragment	Target_aligned_fragment	Inhibition	Target_Desc.	Multiplicity
sly-miR172a	PvAP2-ERF173	0,5	11,33	1	20	3656	3675	AGAAUCUUGAUGAUGCUGCA	UGCAGCAUCAUCAGGAUUCU	Cleavage		1
sly-miR172a	PvAP2-ERF78	0,5	10,114	1	20	3797	3816	AGAAUCUUGAUGAUGCUGCA	UGCAGCAUCAUCAGGAUUCU	Cleavage		1
sly-miR172a	PvAP2-ERF20	1,5	9,626	1	20	2530	2549	AGAAUCUUGAUGAUGCUGCA	UGCAGCAUCAUCAGGAUUCC	Cleavage		1
sly-miR172a	PvAP2-ERF25	1,5	16,15	1	20	3064	3083	AGAAUCUUGAUGAUGCUGCA	UGCAGCAUCAUCAGGAUUCC	Cleavage		1
sly-miR172a	PvAP2-ERF62	1,5	16,463	1	20	3200	3219	AGAAUCUUGAUGAUGCUGCA	UGCAGCAUCAUCAGGAUUCC	Cleavage		1
sly-miR172a	PvAP2-ERF113	2,5	22,52	1	20	3572	3591	AGAAUCUUGAUGAUGCUGCA	AGCAGCAUCAUCAGGAUUCG	Cleavage		1
sly-miR172b	PvAP2-ERF173	0,5	11,33	1	20	3656	3675	AGAAUCUUGAUGAUGCUGCA	UGCAGCAUCAUCAGGAUUCU	Cleavage		1
sly-miR172b	PvAP2-ERF78	0,5	10,114	1	20	3797	3816	AGAAUCUUGAUGAUGCUGCA	UGCAGCAUCAUCAGGAUUCU	Cleavage		1
sly-miR172b	PvAP2-ERF20	1,5	9,626	1	20	2530	2549	AGAAUCUUGAUGAUGCUGCA	UGCAGCAUCAUCAGGAUUCC	Cleavage		1
sly-miR172b	PvAP2-ERF25	1,5	16,15	1	20	3064	3083	AGAAUCUUGAUGAUGCUGCA	UGCAGCAUCAUCAGGAUUCC	Cleavage		1
sly-miR172b	PvAP2-ERF62	1,5	16,463	1	20	3200	3219	AGAAUCUUGAUGAUGCUGCA	UGCAGCAUCAUCAGGAUUCC	Cleavage		1
sly-miR172b	PvAP2-ERF113	2,5	22,52	1	20	3572	3591	AGAAUCUUGAUGAUGCUGCA	AGCAGCAUCAUCAGGAUUCG	Cleavage		1
sly-miR5302	PvAP2-ERF90	2,5	13,664	1	20	204	222	AAACGAGGUUUGUUACUUUG	CGAAG-AACAAACCUCGUUU	Cleavage		1
Selaginella moellendorffii												
miRNA_Acc.	Target_Acc.	Expectation	UPE	miRNA_start	miRNA_end	Target_start	Target_end	miRNA_aligned_fragment	Target_aligned_fragment	Inhibition	Target_Desc.	Multiplicity
smo-miR1097	PvAP2-ERF42	3	16,633	1	20	569	588	UAGCCAUUGUUGUUGUUGGA	UCCAACAACAACAGUGUCUC	Cleavage		1
smo-miR1106	PvAP2-ERF60	2,5	10,415	1	20	3171	3190	UUUAAAGGUGUUAAUGUGUG	UACACAAUAAUAUCUUUAAA	Cleavage		1
smo-miR156c	PvAP2-ERF117	3	21,038	1	20	383	402	UUGACAGAAGAAAGAGAGCA	UCUUCUUCUUCUUCUGUCAA	Cleavage		1
Salvia sclarea												

miRNA_Acc.	Target_Acc.	Expectation	UPE	miRNA_start	miRNA_end	Target_start	Target_end	miRNA_aligned_fragment	Target_aligned_fragment	Inhibition	Target_Desc.	Multiplicity
ssl-miR172	PvAP2-ERF173	0,5	11,33	1	20	3656	3675	AGAAUCUUGAUGAUGCUGCA	UGCAGCAUCAUCAGGAUUCU	Cleavage		1
ssl-miR172	PvAP2-ERF78	0,5	10,114	1	20	3797	3816	AGAAUCUUGAUGAUGCUGCA	UGCAGCAUCAUCAGGAUUCU	Cleavage		1
ssl-miR172	PvAP2-ERF20	1,5	9,626	1	20	2530	2549	AGAAUCUUGAUGAUGCUGCA	UGCAGCAUCAUCAGGAUUCC	Cleavage		1
ssl-miR172	PvAP2-ERF25	1,5	16,15	1	20	3064	3083	AGAAUCUUGAUGAUGCUGCA	UGCAGCAUCAUCAGGAUUCC	Cleavage		1
ssl-miR172	PvAP2-ERF62	1,5	16,463	1	20	3200	3219	AGAAUCUUGAUGAUGCUGCA	UGCAGCAUCAUCAGGAUUCC	Cleavage		1
ssl-miR172	PvAP2-ERF113	2,5	22,52	1	20	3572	3591	AGAAUCUUGAUGAUGCUGCA	AGCAGCAUCAUCAGGAUUCG	Cleavage		1

Solanum tuberosum

miRNA_Acc.	Target_Acc.	Expectation	UPE	miRNA_start	miRNA_end	Target_start	Target_end	miRNA_aligned_fragment	Target_aligned_fragment	Inhibition	Target_Desc.	Multiplicity
stu-miR172a-3p	PvAP2-ERF173	0,5	11,33	1	20	3656	3675	AGAAUCUUGAUGAUGCUGCA	UGCAGCAUCAUCAGGAUUCU	Cleavage		1
stu-miR172a-3p	PvAP2-ERF78	0,5	10,114	1	20	3797	3816	AGAAUCUUGAUGAUGCUGCA	UGCAGCAUCAUCAGGAUUCU	Cleavage		1
stu-miR172a-3p	PvAP2-ERF20	1,5	9,626	1	20	2530	2549	AGAAUCUUGAUGAUGCUGCA	UGCAGCAUCAUCAGGAUUCC	Cleavage		1
stu-miR172a-3p	PvAP2-ERF25	1,5	16,15	1	20	3064	3083	AGAAUCUUGAUGAUGCUGCA	UGCAGCAUCAUCAGGAUUCC	Cleavage		1
stu-miR172a-3p	PvAP2-ERF62	1,5	16,463	1	20	3200	3219	AGAAUCUUGAUGAUGCUGCA	UGCAGCAUCAUCAGGAUUCC	Cleavage		1
stu-miR172a-3p	PvAP2-ERF113	2,5	22,52	1	20	3572	3591	AGAAUCUUGAUGAUGCUGCA	AGCAGCAUCAUCAGGAUUCG	Cleavage		1
stu-miR172b-3p	PvAP2-ERF173	0,5	11,33	1	20	3656	3675	AGAAUCUUGAUGAUGCUGCA	UGCAGCAUCAUCAGGAUUCU	Cleavage		1
stu-miR172b-3p	PvAP2-ERF78	0,5	10,114	1	20	3797	3816	AGAAUCUUGAUGAUGCUGCA	UGCAGCAUCAUCAGGAUUCU	Cleavage		1
stu-miR172b-3p	PvAP2-ERF20	1,5	9,626	1	20	2530	2549	AGAAUCUUGAUGAUGCUGCA	UGCAGCAUCAUCAGGAUUCC	Cleavage		1
stu-miR172b-3p	PvAP2-ERF25	1,5	16,15	1	20	3064	3083	AGAAUCUUGAUGAUGCUGCA	UGCAGCAUCAUCAGGAUUCC	Cleavage		1
stu-miR172b-3p	PvAP2-ERF62	1,5	16,463	1	20	3200	3219	AGAAUCUUGAUGAUGCUGCA	UGCAGCAUCAUCAGGAUUCC	Cleavage		1
stu-miR172b-3p	PvAP2-ERF113	2,5	22,52	1	20	3572	3591	AGAAUCUUGAUGAUGCUGCA	AGCAGCAUCAUCAGGAUUCG	Cleavage		1
stu-miR172d-3p	PvAP2-ERF20	0,5	9,626	1	21	2529	2549	GGAAUCUUGAUGAUGCUGCAG	CUGCAGCAUCAUCAGGAUUCC	Cleavage		1
stu-miR172d-3p	PvAP2-ERF25	0,5	16,15	1	21	3063	3083	GGAAUCUUGAUGAUGCUGCAG	CUGCAGCAUCAUCAGGAUUCC	Cleavage		1
stu-miR172d-3p	PvAP2-ERF62	0,5	16,463	1	21	3199	3219	GGAAUCUUGAUGAUGCUGCAG	CUGCAGCAUCAUCAGGAUUCC	Cleavage		1
stu-miR172d-3p	PvAP2-ERF173	1	11,33	1	21	3655	3675	GGAAUCUUGAUGAUGCUGCAG	CUGCAGCAUCAUCAGGAUUCU	Cleavage		1
stu-miR172d-3p	PvAP2-ERF78	1	10,114	1	21	3796	3816	GGAAUCUUGAUGAUGCUGCAG	CUGCAGCAUCAUCAGGAUUCU	Cleavage		1
stu-miR172d-3p	PvAP2-ERF113	2,5	22,52	1	21	3571	3591	GGAAUCUUGAUGAUGCUGCAG	CAGCAGCAUCAUCAGGAUUCG	Cleavage		1
stu-miR172e-3p	PvAP2-ERF173	0,5	11,33	1	20	3656	3675	AGAAUCUUGAUGAUGCUGCA	UGCAGCAUCAUCAGGAUUCU	Cleavage		1
stu-miR172e-3p	PvAP2-ERF78	0,5	10,114	1	20	3797	3816	AGAAUCUUGAUGAUGCUGCA	UGCAGCAUCAUCAGGAUUCU	Cleavage		1
stu-miR172e-3p	PvAP2-ERF20	1,5	9,626	1	20	2530	2549	AGAAUCUUGAUGAUGCUGCA	UGCAGCAUCAUCAGGAUUCC	Cleavage		1
stu-miR172e-3p	PvAP2-ERF25	1,5	16,15	1	20	3064	3083	AGAAUCUUGAUGAUGCUGCA	UGCAGCAUCAUCAGGAUUCC	Cleavage		1
stu-miR172e-3p	PvAP2-ERF62	1,5	16,463	1	20	3200	3219	AGAAUCUUGAUGAUGCUGCA	UGCAGCAUCAUCAGGAUUCC	Cleavage		1
stu-miR172e-3p	PvAP2-ERF113	2,5	22,52	1	20	3572	3591	AGAAUCUUGAUGAUGCUGCA	AGCAGCAUCAUCAGGAUUCG	Cleavage		1
stu-miR172e-5p	PvAP2-ERF126	3	17,747	1	20	956	975	GCAACAUCAUCAAGAUUCAC	GAGAAUGAUGAUGAUGUUGC	Cleavage		1
stu-miR408a-3p	PvAP2-ERF25	2,5	18,4	1	21	1691	1711	UGCACAGCCUCUUCCCUGGUU	AACCAAGGAAGAGUUUGUGCA	Cleavage		1
stu-miR408a-3p	PvAP2-ERF133	2,5	17,639	1	20	1330	1349	UGCACAGCCUCUUCCCUGGU	ACCAAGGAAGAGUUUGUGCA	Cleavage		1
stu-miR8004	PvERF4	3	16,689	1	20	374	393	AGGGGUUGUGUAUGUGUUUG	CAGACACAAACACGACCUUU	Cleavage	atggtgaaaccggccat	1
stu-miR8011a-5p	PvAP2-ERF32	3	14,135	1	20	2653	2672	UUGUGUGAGGUUUCUUUUUG	UGAAAGGAAACCUUGCACAG	Cleavage		1
stu-miR8021	PvAP2-ERF37	2,5	23,596	1	24	1427	1450	AUUCAAGGCUCAAACUCGAGACCU	AUGGCUGUGGUUUGAGCCUUGAAU	Cleavage		1
stu-miR8032a-3p	PvAP2-ERF70	2,5	7,929	1	22	100	121	AGUGUGAGUCGGUGUGAUUAGG	UCUAACCUCACUGACUCACACU	Cleavage		1
stu-miR8032b-3p	PvAP2-ERF70	2,5	7,929	1	22	100	121	AGUGUGAGUCGGUGCGAUUAGG	UCUAACCUCACUGACUCACACU	Cleavage		1
stu-miR8032d-3p	PvAP2-ERF70	3	7,929	1	22	100	121	AGUGUGAGUUGGUGCGAUUAGG	UCUAACCUCACUGACUCACACU	Cleavage		1
stu-miR8032e-3p	PvAP2-ERF70	2,5	7,929	1	22	100	121	AGUGUGAGUCGGUGUGAUUAGG	UCUAACCUCACUGACUCACACU	Cleavage		1
stu-miR8032f-3p	PvAP2-ERF70	2,5	7,929	1	22	100	121	AGUGUGAGUCGGUGCGAUUAGG	UCUAACCUCACUGACUCACACU	Cleavage		1
stu-miR8032g-3p	PvAP2-ERF70	2,5	7,929	1	22	100	121	AGUGUGAGUCGGUGCGAUUAGG	UCUAACCUCACUGACUCACACU	Cleavage		1
stu-miR8047	PvAP2-ERF174	2,5	18,475	1	19	445	464	CCAUUUU-UUCGAAAUUAGA	UCUAAUUUUGAAGAAAAUGG	Cleavage		1

Triticum aestivum

miRNA_Acc.	Target_Acc.	Expectation	UPE	miRNA_start	miRNA_end	Target_start	Target_end	miRNA_aligned_fragment	Target_aligned_fragment	Inhibition	Target_Desc.	Multiplicity
tae-miR1134	PvAP2-ERF55	3	13,469	1	22	2981	3001	CAACAACAACAAGAAGAAGAAG	UUUAUUCUUCU-GUUGUUGUUG	Translation		1

Theobroma cacao

miRNA_Acc.	Target_Acc.	Expectation	UPE	miRNA_start	miRNA_end	Target_start	Target_end	miRNA_aligned_fragment	Target_aligned_fragment	Inhibition	Target_Desc.	Multiplicity
tcc-miR172a	PvAP2-ERF20	0,5	9,626	1	20	2530	2549	GGAAUCUUGAUGAUGCUGCA	UGCAGCAUCAUCAGGAUUCC	Cleavage		1
tcc-miR172a	PvAP2-ERF25	0,5	16,15	1	20	3064	3083	GGAAUCUUGAUGAUGCUGCA	UGCAGCAUCAUCAGGAUUCC	Cleavage		1
tcc-miR172a	PvAP2-ERF62	0,5	16,463	1	20	3200	3219	GGAAUCUUGAUGAUGCUGCA	UGCAGCAUCAUCAGGAUUCC	Cleavage		1
tcc-miR172a	PvAP2-ERF173	1	11,33	1	20	3656	3675	GGAAUCUUGAUGAUGCUGCA	UGCAGCAUCAUCAGGAUUCU	Cleavage		1

tcc-miR172a	PvAP2-ERF78	1	10,114	1	20	3797	3816	GGAAUCUUGAUGAUGCUGCA	UGCAGCAUCAUCAGGAUUCU	Cleavage		1
tcc-miR172a	PvAP2-ERF113	2,5	22,52	1	20	3572	3591	GGAAUCUUGAUGAUGCUGCA	AGCAGCAUCAUCAGGAUUCG	Cleavage		1
tcc-miR172b	PvAP2-ERF173	0,5	11,33	1	20	3656	3675	AGAAUCUUGAUGAUGCUGCA	UGCAGCAUCAUCAGGAUUCU	Cleavage		1
tcc-miR172b	PvAP2-ERF78	0,5	10,114	1	20	3797	3816	AGAAUCUUGAUGAUGCUGCA	UGCAGCAUCAUCAGGAUUCU	Cleavage		1
tcc-miR172b	PvAP2-ERF20	1,5	9,626	1	20	2530	2549	AGAAUCUUGAUGAUGCUGCA	UGCAGCAUCAUCAGGAUUCC	Cleavage		1
tcc-miR172b	PvAP2-ERF25	1,5	16,15	1	20	3064	3083	AGAAUCUUGAUGAUGCUGCA	UGCAGCAUCAUCAGGAUUCC	Cleavage		1
tcc-miR172b	PvAP2-ERF62	1,5	16,463	1	20	3200	3219	AGAAUCUUGAUGAUGCUGCA	UGCAGCAUCAUCAGGAUUCC	Cleavage		1
tcc-miR172b	PvAP2-ERF113	2,5	22,52	1	20	3572	3591	AGAAUCUUGAUGAUGCUGCA	AGCAGCAUCAUCAGGAUUCG	Cleavage		1
tcc-miR172c	PvAP2-ERF20	0,5	9,626	1	20	2530	2549	GGAAUCUUGAUGAUGCUGCA	UGCAGCAUCAUCAGGAUUCC	Cleavage		1
tcc-miR172c	PvAP2-ERF25	0,5	16,15	1	20	3064	3083	GGAAUCUUGAUGAUGCUGCA	UGCAGCAUCAUCAGGAUUCC	Cleavage		1
tcc-miR172c	PvAP2-ERF62	0,5	16,463	1	20	3200	3219	GGAAUCUUGAUGAUGCUGCA	UGCAGCAUCAUCAGGAUUCC	Cleavage		1
tcc-miR172c	PvAP2-ERF173	1	11,33	1	20	3656	3675	GGAAUCUUGAUGAUGCUGCA	UGCAGCAUCAUCAGGAUUCU	Cleavage		1
tcc-miR172c	PvAP2-ERF78	1	10,114	1	20	3797	3816	GGAAUCUUGAUGAUGCUGCA	UGCAGCAUCAUCAGGAUUCU	Cleavage		1
tcc-miR172c	PvAP2-ERF113	2,5	22,52	1	20	3572	3591	GGAAUCUUGAUGAUGCUGCA	AGCAGCAUCAUCAGGAUUCG	Cleavage		1
tcc-miR172d	PvAP2-ERF173	0	11,33	1	20	3656	3675	AGAAUCCUGAUGAUGCUGCA	UGCAGCAUCAUCAGGAUUCU	Cleavage		1
tcc-miR172d	PvAP2-ERF78	0	10,114	1	20	3797	3816	AGAAUCCUGAUGAUGCUGCA	UGCAGCAUCAUCAGGAUUCU	Cleavage		1
tcc-miR172d	PvAP2-ERF20	1	9,626	1	20	2530	2549	AGAAUCCUGAUGAUGCUGCA	UGCAGCAUCAUCAGGAUUCC	Cleavage		1
tcc-miR172d	PvAP2-ERF25	1	16,15	1	20	3064	3083	AGAAUCCUGAUGAUGCUGCA	UGCAGCAUCAUCAGGAUUCC	Cleavage		1
tcc-miR172d	PvAP2-ERF62	1	16,463	1	20	3200	3219	AGAAUCCUGAUGAUGCUGCA	UGCAGCAUCAUCAGGAUUCC	Cleavage		1
tcc-miR172d	PvAP2-ERF113	2	22,52	1	20	3572	3591	AGAAUCCUGAUGAUGCUGCA	AGCAGCAUCAUCAGGAUUCG	Cleavage		1
tcc-miR172e	PvAP2-ERF173	0,5	11,33	1	20	3656	3675	AGAAUCUUGAUGAUGCUGCA	UGCAGCAUCAUCAGGAUUCU	Cleavage		1
tcc-miR172e	PvAP2-ERF78	0,5	10,114	1	20	3797	3816	AGAAUCUUGAUGAUGCUGCA	UGCAGCAUCAUCAGGAUUCU	Cleavage		1
tcc-miR172e	PvAP2-ERF20	1,5	9,626	1	20	2530	2549	AGAAUCUUGAUGAUGCUGCA	UGCAGCAUCAUCAGGAUUCC	Cleavage		1
tcc-miR172e	PvAP2-ERF25	1,5	16,15	1	20	3064	3083	AGAAUCUUGAUGAUGCUGCA	UGCAGCAUCAUCAGGAUUCC	Cleavage		1
tcc-miR172e	PvAP2-ERF62	1,5	16,463	1	20	3200	3219	AGAAUCUUGAUGAUGCUGCA	UGCAGCAUCAUCAGGAUUCC	Cleavage		1
tcc-miR172e	PvAP2-ERF113	2,5	22,52	1	20	3572	3591	AGAAUCUUGAUGAUGCUGCA	AGCAGCAUCAUCAGGAUUCG	Cleavage		1
Vigna unguiculata												
miRNA_Acc.	Target_Acc.	Expectation	UPE	miRNA_start	miRNA_end	Target_start	Target_end	miRNA_aligned_fragment	Target_aligned_fragment	Inhibition	Target_Desc.	Multiplicity
vun-miR172	PvAP2-ERF173	0,5	11,33	1	20	3656	3675	AGAAUCUUGAUGAUGCUGCA	UGCAGCAUCAUCAGGAUUCU	Cleavage		1
vun-miR172	PvAP2-ERF78	0,5	10,114	1	20	3797	3816	AGAAUCUUGAUGAUGCUGCA	UGCAGCAUCAUCAGGAUUCU	Cleavage		1
vun-miR172	PvAP2-ERF20	1,5	9,626	1	20	2530	2549	AGAAUCUUGAUGAUGCUGCA	UGCAGCAUCAUCAGGAUUCC	Cleavage		1
vun-miR172	PvAP2-ERF25	1,5	16,15	1	20	3064	3083	AGAAUCUUGAUGAUGCUGCA	UGCAGCAUCAUCAGGAUUCC	Cleavage		1
vun-miR172	PvAP2-ERF62	1,5	16,463	1	20	3200	3219	AGAAUCUUGAUGAUGCUGCA	UGCAGCAUCAUCAGGAUUCC	Cleavage		1
vun-miR172	PvAP2-ERF113	2,5	22,52	1	20	3572	3591	AGAAUCUUGAUGAUGCUGCA	AGCAGCAUCAUCAGGAUUCG	Cleavage		1
Vitis vinifera												
miRNA_Acc.	Target_Acc.	Expectation	UPE	miRNA_start	miRNA_end	Target_start	Target_end	miRNA_aligned_fragment	Target_aligned_fragment	Inhibition	Target_Desc.	Multiplicity
vvi-miR156h	PvAP2-ERF131	3	18,595	1	20	595	614	UGACAGAAGAGAGAGAGCAU	AUGAUGUCUCACUUCUGUCA	Translation		1
vvi-miR156h	PvAP2-ERF128	3	18,523	1	20	2185	2204	UGACAGAAGAGAGAGAGCAU	AUUCUCUCUUUUCUCUGUCA	Cleavage		1
vvi-miR172a	PvAP2-ERF20	2,5	9,626	1	20	2530	2549	UGAAUCUUGAUGAUGCUACA	UGCAGCAUCAUCAGGAUUCC	Cleavage		1
vvi-miR172a	PvAP2-ERF25	2,5	16,15	1	20	3064	3083	UGAAUCUUGAUGAUGCUACA	UGCAGCAUCAUCAGGAUUCC	Cleavage		1
vvi-miR172a	PvAP2-ERF62	2,5	16,463	1	20	3200	3219	UGAAUCUUGAUGAUGCUACA	UGCAGCAUCAUCAGGAUUCC	Cleavage		1
vvi-miR172a	PvAP2-ERF173	2,5	11,33	1	20	3656	3675	UGAAUCUUGAUGAUGCUACA	UGCAGCAUCAUCAGGAUUCU	Cleavage		1
vvi-miR172a	PvAP2-ERF78	2,5	10,114	1	20	3797	3816	UGAAUCUUGAUGAUGCUACA	UGCAGCAUCAUCAGGAUUCU	Cleavage		1
vvi-miR172a	PvAP2-ERF113	3	22,52	1	20	3572	3591	UGAAUCUUGAUGAUGCUACA	AGCAGCAUCAUCAGGAUUCG	Cleavage		1
vvi-miR172b	PvAP2-ERF20	2,5	9,626	1	20	2530	2549	UGAAUCUUGAUGAUGCUACA	UGCAGCAUCAUCAGGAUUCC	Cleavage		1
vvi-miR172b	PvAP2-ERF25	2,5	16,15	1	20	3064	3083	UGAAUCUUGAUGAUGCUACA	UGCAGCAUCAUCAGGAUUCC	Cleavage		1
vvi-miR172b	PvAP2-ERF62	2,5	16,463	1	20	3200	3219	UGAAUCUUGAUGAUGCUACA	UGCAGCAUCAUCAGGAUUCC	Cleavage		1
vvi-miR172b	PvAP2-ERF173	2,5	11,33	1	20	3656	3675	UGAAUCUUGAUGAUGCUACA	UGCAGCAUCAUCAGGAUUCU	Cleavage		1
vvi-miR172b	PvAP2-ERF78	2,5	10,114	1	20	3797	3816	UGAAUCUUGAUGAUGCUACA	UGCAGCAUCAUCAGGAUUCU	Cleavage		1
vvi-miR172b	PvAP2-ERF113	3	22,52	1	20	3572	3591	UGAAUCUUGAUGAUGCUACA	AGCAGCAUCAUCAGGAUUCG	Cleavage		1
vvi-miR172c	PvAP2-ERF20	0,5	9,626	1	21	2529	2549	GGAAUCUUGAUGAUGCUGCAG	CUGCAGCAUCAUCAGGAUUCC	Cleavage		1
vvi-miR172c	PvAP2-ERF25	0,5	16,15	1	21	3063	3083	GGAAUCUUGAUGAUGCUGCAG	CUGCAGCAUCAUCAGGAUUCC	Cleavage		1
vvi-miR172c	PvAP2-ERF62	0,5	16,463	1	21	3199	3219	GGAAUCUUGAUGAUGCUGCAG	CUGCAGCAUCAUCAGGAUUCC	Cleavage		1
vvi-miR172c	PvAP2-ERF173	1	11,33	1	21	3655	3675	GGAAUCUUGAUGAUGCUGCAG	CUGCAGCAUCAUCAGGAUUCU	Cleavage		1
vvi-miR172c	PvAP2-ERF78	1	10,114	1	21	3796	3816	GGAAUCUUGAUGAUGCUGCAG	CUGCAGCAUCAUCAGGAUUCU	Cleavage		1

vvi-miR172c	PvAP2-ERF113	2,5	22,52	1	21	3571	3591	GGAAUCUUGAUGAUGCUGCAG	CAGCAGCAUCAUCAGGAUUCG	Cleavage		1
vvi-miR172d	PvAP2-ERF173	0,5	11,541	1	22	3656	3677	UGAGAAUCUUGAUGAUGCUGCA	UGCAGCAUCAUCAGGAUUCUCA	Cleavage		1
vvi-miR172d	PvAP2-ERF78	0,5	5,882	1	22	3797	3818	UGAGAAUCUUGAUGAUGCUGCA	UGCAGCAUCAUCAGGAUUCUCA	Cleavage		1
vvi-miR172d	PvAP2-ERF25	2	16,394	1	22	3064	3085	UGAGAAUCUUGAUGAUGCUGCA	UGCAGCAUCAUCAGGAUUCCCA	Cleavage		1
vvi-miR172d	PvAP2-ERF62	2	18,825	1	22	3200	3221	UGAGAAUCUUGAUGAUGCUGCA	UGCAGCAUCAUCAGGAUUCCCA	Cleavage		1
vvi-miR172d	PvAP2-ERF20	3	9,676	1	22	2530	2551	UGAGAAUCUUGAUGAUGCUGCA	UGCAGCAUCAUCAGGAUUCCCU	Cleavage		1
vvi-miR3633a-5p	PvAP2-ERF125	3	8,263	1	20	1066	1085	GGAAUGGAUGGUUAGGAGAG	CUCUCCAAAUCUUUCAUUCC	Translation		1
Zea mays												
miRNA_Acc.	Target_Acc.	Expectation	UPE	miRNA_start	miRNA_end	Target_start	Target_end	miRNA_aligned_fragment	Target_aligned_fragment	Inhibition	Target_Desc.	Multiplicity
zma-miR156e-3p	PvAP2-ERF46	3	15,813	1	22	2164	2185	GCUCACUGCUCUCUCUGUCAUC	GAUGACAGUGAGCCCAGUGAGC	Translation		1
zma-miR156h-3p	PvERF3	3	23,803	1	20	2745	2764	GCUCACUGCUCUUUCUGUCA	UUGCAGAAAGAGUAGUGGGU	Cleavage		1
zma-miR159e-5p	PvAP2-ERF171	3	16,656	1	21	862	882	CAGCUCCUGCAGCAUCUGUUC	GAGCAGAUGAUUCAGGAGUUG	Translation		1
zma-miR159g-3p	PvAP2-ERF135	2,5	1,098	1	20	41	60	UUUGGAGUGAAGGGAGUUCU	AAACCUCUCUUCACUCCAAA	Cleavage		1
zma-miR159h-3p	PvAP2-ERF135	2,5	1,098	1	20	41	60	UUUGGAGUGAAGGGAGCUCU	AAACCUCUCUUCACUCCAAA	Cleavage		1
zma-miR159i-3p	PvAP2-ERF135	2,5	1,098	1	20	41	60	UUUGGAGUGAAGGGAGCUCU	AAACCUCUCUUCACUCCAAA	Cleavage		1
zma-miR171g-5p	PvAP2-ERF179	3	17,265	1	20	2151	2170	UAUUGACUUGGCUCAUCUCU	AAAGAUGAGUAAAGUUAAUA	Translation		1
zma-miR172a	PvAP2-ERF173	0,5	11,33	1	20	3656	3675	AGAAUCUUGAUGAUGCUGCA	UGCAGCAUCAUCAGGAUUCU	Cleavage		1
zma-miR172a	PvAP2-ERF78	0,5	10,114	1	20	3797	3816	AGAAUCUUGAUGAUGCUGCA	UGCAGCAUCAUCAGGAUUCU	Cleavage		1
zma-miR172a	PvAP2-ERF20	1,5	9,626	1	20	2530	2549	AGAAUCUUGAUGAUGCUGCA	UGCAGCAUCAUCAGGAUUCC	Cleavage		1
zma-miR172a	PvAP2-ERF25	1,5	16,15	1	20	3064	3083	AGAAUCUUGAUGAUGCUGCA	UGCAGCAUCAUCAGGAUUCC	Cleavage		1
zma-miR172a	PvAP2-ERF62	1,5	16,463	1	20	3200	3219	AGAAUCUUGAUGAUGCUGCA	UGCAGCAUCAUCAGGAUUCC	Cleavage		1
zma-miR172a	PvAP2-ERF113	2,5	22,52	1	20	3572	3591	AGAAUCUUGAUGAUGCUGCA	AGCAGCAUCAUCAGGAUUCG	Cleavage		1
zma-miR172b-3p	PvAP2-ERF173	0,5	11,33	1	20	3656	3675	AGAAUCUUGAUGAUGCUGCA	UGCAGCAUCAUCAGGAUUCU	Cleavage		1
zma-miR172b-3p	PvAP2-ERF78	0,5	10,114	1	20	3797	3816	AGAAUCUUGAUGAUGCUGCA	UGCAGCAUCAUCAGGAUUCU	Cleavage		1
zma-miR172b-3p	PvAP2-ERF20	1,5	9,626	1	20	2530	2549	AGAAUCUUGAUGAUGCUGCA	UGCAGCAUCAUCAGGAUUCC	Cleavage		1
zma-miR172b-3p	PvAP2-ERF25	1,5	16,15	1	20	3064	3083	AGAAUCUUGAUGAUGCUGCA	UGCAGCAUCAUCAGGAUUCC	Cleavage		1
zma-miR172b-3p	PvAP2-ERF62	1,5	16,463	1	20	3200	3219	AGAAUCUUGAUGAUGCUGCA	UGCAGCAUCAUCAGGAUUCC	Cleavage		1
zma-miR172b-3p	PvAP2-ERF113	2,5	22,52	1	20	3572	3591	AGAAUCUUGAUGAUGCUGCA	AGCAGCAUCAUCAGGAUUCG	Cleavage		1
zma-miR172c-3p	PvAP2-ERF173	0,5	11,33	1	20	3656	3675	AGAAUCUUGAUGAUGCUGCA	UGCAGCAUCAUCAGGAUUCU	Cleavage		1
zma-miR172c-3p	PvAP2-ERF78	0,5	10,114	1	20	3797	3816	AGAAUCUUGAUGAUGCUGCA	UGCAGCAUCAUCAGGAUUCU	Cleavage		1
zma-miR172c-3p	PvAP2-ERF20	1,5	9,626	1	20	2530	2549	AGAAUCUUGAUGAUGCUGCA	UGCAGCAUCAUCAGGAUUCC	Cleavage		1
zma-miR172c-3p	PvAP2-ERF25	1,5	16,15	1	20	3064	3083	AGAAUCUUGAUGAUGCUGCA	UGCAGCAUCAUCAGGAUUCC	Cleavage		1
zma-miR172c-3p	PvAP2-ERF62	1,5	16,463	1	20	3200	3219	AGAAUCUUGAUGAUGCUGCA	UGCAGCAUCAUCAGGAUUCC	Cleavage		1
zma-miR172c-3p	PvAP2-ERF113	2,5	22,52	1	20	3572	3591	AGAAUCUUGAUGAUGCUGCA	AGCAGCAUCAUCAGGAUUCG	Cleavage		1
zma-miR172d-3p	PvAP2-ERF78	0,5	10,114	1	20	3797	3816	AGAAUCUUGAUGAUGCUGCA	UGCAGCAUCAUCAGGAUUCU	Cleavage		1
zma-miR172d-3p	PvAP2-ERF173	0,5	11,33	1	20	3656	3675	AGAAUCUUGAUGAUGCUGCA	UGCAGCAUCAUCAGGAUUCU	Cleavage		1
zma-miR172d-3p	PvAP2-ERF62	1,5	16,463	1	20	3200	3219	AGAAUCUUGAUGAUGCUGCA	UGCAGCAUCAUCAGGAUUCC	Cleavage		1
zma-miR172d-3p	PvAP2-ERF25	1,5	16,15	1	20	3064	3083	AGAAUCUUGAUGAUGCUGCA	UGCAGCAUCAUCAGGAUUCC	Cleavage		1
zma-miR172d-3p	PvAP2-ERF20	1,5	9,626	1	20	2530	2549	AGAAUCUUGAUGAUGCUGCA	UGCAGCAUCAUCAGGAUUCC	Cleavage		1
zma-miR172d-3p	PvAP2-ERF113	2,5	22,52	1	20	3572	3591	AGAAUCUUGAUGAUGCUGCA	AGCAGCAUCAUCAGGAUUCG	Cleavage		1
zma-miR172e	PvAP2-ERF20	0,5	9,626	1	20	2530	2549	GGAAUCUUGAUGAUGCUGCA	UGCAGCAUCAUCAGGAUUCC	Cleavage		1
zma-miR172e	PvAP2-ERF25	0,5	16,15	1	20	3064	3083	GGAAUCUUGAUGAUGCUGCA	UGCAGCAUCAUCAGGAUUCC	Cleavage		1
zma-miR172e	PvAP2-ERF62	0,5	16,463	1	20	3200	3219	GGAAUCUUGAUGAUGCUGCA	UGCAGCAUCAUCAGGAUUCC	Cleavage		1
zma-miR172e	PvAP2-ERF173	1	11,33	1	20	3656	3675	GGAAUCUUGAUGAUGCUGCA	UGCAGCAUCAUCAGGAUUCU	Cleavage		1
zma-miR172e	PvAP2-ERF78	1	10,114	1	20	3797	3816	GGAAUCUUGAUGAUGCUGCA	UGCAGCAUCAUCAGGAUUCU	Cleavage		1
zma-miR172e	PvAP2-ERF113	2,5	22,52	1	20	3572	3591	GGAAUCUUGAUGAUGCUGCA	AGCAGCAUCAUCAGGAUUCG	Cleavage		1
zma-miR2275a-3p	PvAP2-ERF7	2,5	16,862	1	22	1455	1476	UUUGUUUUCCUCCAAUAUCUCA	UGAGAAAUUGGAGGAAACCAAA	Cleavage		1
zma-miR2275d-3p	PvAP2-ERF7	3	16,862	1	22	1455	1476	UUUGUUUUCCUCUAAUAUCUCA	UGAGAAAUUGGAGGAAACCAAA	Cleavage		1
